# Transcriptome Analysis of the Chrysanthemum Foliar Nematode, *Aphelenchoides ritzemabosi* (Aphelenchida: Aphelenchoididae)

**DOI:** 10.1371/journal.pone.0166877

**Published:** 2016-11-22

**Authors:** Yu Xiang, Dong-Wei Wang, Jun-Yi Li, Hui Xie, Chun-Ling Xu, Yu Li

**Affiliations:** Laboratory of Plant Nematology and Research Center of Nematodes of Plant Quarantine, Department of Plant Pathology, College of Agriculture, South China Agricultural University, Guangzhou, People’s Republic of China; James Hutton Institute, UNITED KINGDOM

## Abstract

The chrysanthemum foliar nematode (CFN), *Aphelenchoides ritzemabosi*, is a plant parasitic nematode that attacks many plants. In this study, a transcriptomes of mixed-stage population of CFN was sequenced on the Illumina HiSeq 2000 platform. 68.10 million Illumina high quality paired end reads were obtained which generated 26,817 transcripts with a mean length of 1,032 bp and an N50 of 1,672 bp, of which 16,467 transcripts were annotated against six databases. In total, 20,311 coding region sequences (CDS), 495 simple sequence repeats (SSRs) and 8,353 single-nucleotide polymorphisms (SNPs) were predicted, respectively. The CFN with the most shared sequences was *B*. *xylophilus* with 16,846 (62.82%) common transcripts and 10,543 (39.31%) CFN transcripts matched sequences of all of four plant parasitic nematodes compared. A total of 111 CFN transcripts were predicted as homologues of 7 types of carbohydrate-active enzymes (CAZymes) with plant/fungal cell wall-degrading activities, fewer transcripts were predicted as homologues of plant cell wall-degrading enzymes than fungal cell wall-degrading enzymes. The phylogenetic analysis of GH5, GH16, GH43 and GH45 proteins between CFN and other organisms showed CFN and other nematodes have a closer phylogenetic relationship. In the CFN transcriptome, sixteen types of genes orthologues with seven classes of protein families involved in the RNAi pathway in *C*. *elegans* were predicted. This research provides comprehensive gene expression information at the transcriptional level, which will facilitate the elucidation of the molecular mechanisms of CFN and the distribution of gene functions at the macro level, potentially revealing improved methods for controlling CFN.

## Introduction

The chrysanthemum foliar nematode (CFN), *Aphelenchoides ritzemabosi* (Schwartz) Steiner and Buhrer, 1932, belongs to Nematoda, Secernentea, Aphelenchida, Aphelenchoididae and is an obligate ecto- and endoparasite of the above-ground parts of plants [1]. CFN is widely distributed globally and is economically important for chrysanthemums and many other ornamental plants. CFN can also severely damage strawberry [2–4]. There have been sporadic occurrences of CFN in China, and CFN has been listed as a quarantine plant pest in China due to potential risks [5].

Next-generation sequencing (NGS) technology has enabled impressive scientific achievements and novel biological applications. NGS has also contributed to a massive expansion of transcriptomics in many fields of biology, reducing the cost, time and performance barriers associated with conventional approaches [6,7]. NGS has been used to study several plant parasitic nematodes, including tylench nematodes, *Globodera pallida*, *Heterodera avenae*, *H*. *schachtii*, *Meloidogyne incognita*, *Nacobbus aberrans*, *Radopholus similis*, *Pratylenchus coffeae*, *P*. *thornei* [8–15], and the aphelench nematodes *Bursaphelenchus xylophilus* and *Aphelenchoides besseyi* [16–18].

Plant parasitic nematodes produce a variety of secreted proteins termed ‘‘parasitism genes” or ‘‘effectors”. These secreted proteins, which include cell-wall modifying enzymes, encompass a variety of functions and manipulate the interactions between nematodes and hosts [16]. Plant parasitic nematodes produce enzymes that break down the cell wall, the primary barrier faced by plant parasite nematodes to invade the plant cytoplasm. Since the first plant cell wall-degrading enzymes (carbohydrate-active enzymes, CAZymes) from nematodes were characterized in cyst nematodes [19], plant cell wall enzymes have been identified in many plant parasitic nematodes, including *Aphelenchoides*, *Bursaphelenchus*, *Ditylenchus*, *Globodera*, *Heterodera*, and *Meloidogyne* [16,17,20]. Some CAZymes that are secreted by parasitic nematodes of the below-ground parts of plants have been predicted to be involved in enabling nematode access to the roots and feeding on host cells [21].

In this research, the Illumina HiSeq 2000 NGS platform was used to analyze the transcriptome of a mixed-stage population of CFN. A total of 26,817 transcripts were generated and analyzed from the CFN mixed-stage library. Using bioinformatics approaches, some transcripts were predicted as orthologues of genes involved in the RNAi pathway and with plant/fungal cell wall–degrading activities from this library. We shed light on the basic biology and genetic background of CFN, and the transcriptome data generated in this study will facilitate the characterization of the mechanisms of CFN at the molecular level. In addition, this research also provides significant new information on expressed genes that may aid the elucidation of the parasitic abilities of CFN and improved strategies for its control.

## Methods

### Ethics statement

We collected the CFN in areas where chrysanthemum foliar nematodes occurred and no specific permit was required. The field for nematodes collection was neither privately owned nor protected, and did not involve endangered or protected species.

### Nematode samples, nematode extraction and RNA extraction

The CFN (NCBI BioSample accession No. SAMN03401492) used in this research were collected from the leaves of infected *Dendranthema morifolium* (Ramat.) Tzvel. in Kunming City, Yunnan Province, China. After identification by the laboratory of plant nematology of South China Agricultural University, the CFN were maintained by serial subculture on excised carrot (*Daucus carota*) disks in petri dishes at 25°C in a dark incubator [22]. Nematode extraction and total RNA extraction from a mixed-stage population of CFN (3:2.5:1 ratio of adults, juvenile stages (J2–J4) and eggs) were performed as described in Cheng et al. [23].

### cDNA library construction and Illumina sequencing

After total RNA extraction and DNase I (NEB) treatment, magnetic beads with Oligo(dT) beads (Dynabeads mRNA purification kit, Invitrogen) were used to isolate mRNA. The mRNA was then mixed with fragmentation buffer (Ambion) and fragmented into short fragments. Then, cDNA was synthesized using the mRNA fragments as templates. Short fragments were purified and dissolved in ethidium bromide (EB) buffer for end repair and single nucleotide A (adenine) addition. Then, the short fragments were connected with adapters, and suitable fragments were selected as templates for PCR amplification. During the quality control steps, an Agilent 2100 Bioanalyzer (Agilent DNA 1000 Reagents) and an ABI StepOnePlus Real-Time PCR System were used for quantification and qualification of the sample library. Finally, the libraries were sequenced using the Illumina HiSeq^™^ 2000 system (TruSeq SBS KIT-HS V3, Illumina).

### Data assembly and annotation

Raw reads were filtered by removing adaptors, reads with more than 5% unknown nucleotides, and sequences shorter than 20 nt and low quality with Q <20. *De novo* transcriptome assembly was performed with the short-read (90 bp in length) assembly software Trinity (Version: release-20130225) at the parameters of—seqType fq—min_contig_length 100,—min_glue 3—group_pairs_distance 250,—path_ reinforcement_distance 85—min_kmer_cov 3 [24, 25]. Short reads were first assembled into contigs with no gap, then connected the contigs with Trinity, and obtained sequences that no longer could be extended, such sequences are defined as transcripts. Next, all the transcripts (>200bp) annotated based on the BLASTX results (Version: v2.2.26+x64-linux) with an E-value of 1e-5 against five databases, including NCBI Nonredundant protein database (NR) (Version: release-20130408), Swiss-Prot, the Kyoto Encyclopedia of Genes and Genomes (KEGG) (Version: release 63.0), Cluster of Orthologous Groups (COG) (Version: release-20090331) databases and Gene Ontology (GO) (Blast2GO, Version: release 2012-08-01) database. In addition, all the transcripts (>200bp) annotated based on the BLASTN alignments results (Version: v2.2.26+x64-linux) with an E-value of 1e-5 against NCBI Non-redundant nucleotide database (NT) (Version: release-20130408). The best-aligned results were used to determine the sequence direction of the transcripts. If the results from the different databases conflicted, the sequence direction of the transcript was determined using the prioritization order NR, Swiss-Prot, KEGG and COG. When a transcript was not aligned to the above databases, ESTScan software (Version: v3.0.2) was used to decide the sequence direction [26]. Gene function classifications with GO annotations of the transcripts were determined by Blast2GO program with NR annotation [27]. Based on GO annotation, WEGO software [28] was used to display GO functional classification. To confirm validly assembled sequences and identify nematode orthologues with other nematodes, the annotated CFN transcripts were compared with the proteins of the completely sequenced genomes of *B*. *xylophilus* (17,704 proteins), *G*. *pallida* (16,403), *M*. *hapla* (14,420) and *M*. *incognita* (20,365) (http://parasite.wormbase.org/ftp.html), using BLASTX to assign putative orthologues.

### Transcripts analysis

KEGG homologues were identified using the KEGG database and Path_finder software (Version: release 63.0), we further characterized the biological complex behaviors of the genes via metabolic pathway annotations of the transcripts [29]. Proteins with the highest ranking in the BLAST results were used to decide the coding region sequences (CDS) of the transcriptomes with an E-value of 1e-5. The CDS were translated into amino acid sequences using the standard codon table. Transcripts that could not be aligned to any database were scanned by ESTScan to determine the nucleotide sequence (5’->3’) direction and the amino acid sequence of the predicted coding region [26]. Microsatellites (also known as simple sequence repeats, SSRs) detection was done with software MicroSAtellite (MISA) (http://pgrc.ipk-gatersleben.de/misa/misa.html) using CFN transcripts as reference, and the length of these CFN transcripts was more than 150bp. Single-nucleotide polymorphisms (SNPs) of the CFN transcripts were detected with the Short Oligonucleotide Analysis Package (SOAP) (Version: release 1.03), the program can assemble consensus sequence for the genome of a newly sequenced individual based on the alignment of the raw sequencing reads on the transcripts, the SNPs can then be predicted on the consensus sequence through the comparison with the transcripts [30].

### Annotation of the carbohydrate-active enzymes

The CAZymes Analysis Toolkit [31] was used to detect CAZymes in CFN. The CAZy database describes families of structurally related catalytic and carbohydrate-binding modules (or functional domains) of enzymes that degrade, modify, or create glycosidic bonds. The enzyme classes comprise glycoside hydrolases (GHs), glycosyltransferases (GTs), polysaccharide lyases (PLs), carbohydrate esterases (CEs) and auxiliary activities (AAs). The associated modules encompass carbohydrate-binding modules (CBMs) (http://www.cazy.org/). The annotation method “predict CAZymes for nucleotides” was used with an E-value threshold of 0.01, a bitscore threshold of 55 and rule support level of 40 (http://mothra.ornl.gov/cgi-bin/cat/cat_v2.cgi?tab=ORTHOLOGS). The annotation was confirmed manually based on BLAST search homologues and protein length matches. Expansin-like proteins were detected by BLAST search with the core modules of known expansin proteins as the query. The putative functions of the proteins were predicted based on homologues to known protein modules, and the NCBI Conserved Domain Database service was searched using BLASTP to identify catalytic sites [16]. The CFN transcripts were compared to known CAZymes based on different sequence sources of *A*. *besseyi*, *N*. *aberrans*, *P*. *coffeae* and *P*. *thornei* transcriptome, *B*. *xylophilus* whole genome and those obtained from http://mothra.ornl.gov/cgi-bin/cat/cat.cgi?tab=CAZymes [11, 12, 14, 16–18].

The amino acid sequences of GH5, GH16, GH43 and GH45 proteins predicted in the transcriptome of CFN and their homologues identified from the NCBI database were aligned using ClustalW. Based on the amino acid sequences of 30 GH5 proteins from 27 different species, 20 GH16 proteins from 19 different species, 10 GH43 proteins from 10 different species and 13 GH45 proteins from 9 different species, four phylogenetic trees were constructed using the neighbor-joining method in MEGA (Molecular Evolutionary Genetics Analysis, USA) 5.1 [23]. Bootstrap values were calculated from 1000 replicates.

### RNAi pathway genes

Thirty-seven *C*. *elegans* proteins with roles in Exo-RNAi, dicer, amplification, argonautes, RNAi suppressor, RNAi enhancer, uptake and miRNA or endo RNAi (www.wormbase.org) were obtained from NCBI and used in BLAST searches of the CFN transcripts longer than 150 bp for predicted genes. All BLAST hits with an E-value of 1e-5 were manually analyzed for accuracy of automated gene prediction, corrected if necessary and the corresponding CFN predicted proteins subjected to reciprocal BLASTP searches against the *C*. *elegans* protein database [32]. The presence of these genes in *A*. *besseyi*, *B*. *xylophilus*, *M*. *incognita* and *P*. *coffeae* were used to analyze the possible diverse characteristics of CFN and these nematodes [11,16,18,33].

## Results

### Illumina sequencing and assembly

In this study, we obtained an overview of the gene expression profile of a mixed-stage population of CFN. A total of 73,284,868 raw reads were obtained using the Illumina HiSeq 2000 platform, and a total of 68,103,194 high-quality clean reads (accumulated length of 6,129,287,460 bp) were obtained after the removal of adaptor sequences, unknown nucleotides larger than 5% and low-quality reads (the rate of reads which quality value < = 10 is more than 20%). All of the high-quality sequencing reads from the CFN were deposited in NCBI and can be accessed in the Sequence Read Archive (SRA) under the accession number SRR3999959. The average length was 90 bp, and the proportion of high-quality clean reads was 92.93%. The proportions of nucleotides with a quality value greater than 20, unknown nucleotides, and guanidine and cytosine nucleotides among the total CFN nucleotides, were 97.40%, 0.00% and 43.05%, respectively (Tables A and B in S1 File).

The clean reads were assembled to the longest assembled sequences (also called contigs) with Trinity, and resulted in 38,070 contigs with a mean length of 572 bp and an N50 of 1,278 bp, respectively. A total of 26,817 transcripts with a mean length of 1,032 bp and an N50 of 1,672 bp were obtained after further clustering and assembly. Of these transcripts, 15,659 (58.40%) had a length ≥ 500 bp, and 9,535 (35.56%) were ≥ 1,000 bp. The length distributions of the contigs and transcripts and the randomness of sample CFN reads were shown in Figs A-C in S1 File.

### Functional annotation

**Fig 1:**
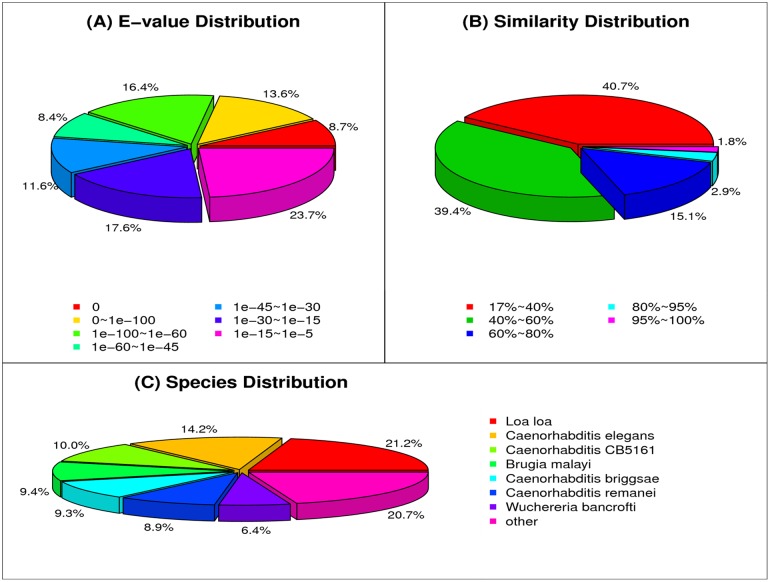
NR classification of a mixed-stage population of *Aphelenchoides ritzemabosi*. (A) The E-value distribution of the results of the NR annotation. (B) The similarity distribution of the NR annotations. (C) The species distribution of the NR annotations.

Of the CFN transcriptome, 16,467 transcripts (61.41%) were annotated against the NR, COG, GO, Swiss-Prot and KEGG databases using BLASTX and against NT using BLASTN (Table C in S1 File). 16,224, 6,410, 9,504, 3,679, 13,344 and 11,645 transcripts were annotated against the NR, COG, GO, NT, Swiss-Prot and KEGG databases, respectively (S2 File). 13,932 transcripts (85.87%) and 2,032 transcripts (55.23%) matched the NR and NT databases with an E-value of 1e-10. The CFN transcripts were annotated as the top organisms and plant parasitic nematodes based on the top BLASTX hit information in the species distribution statistics, the top 5 organisms were *Loa loa* (3,440 transcripts, 21.20%), *C*. *elegans* (2,296, 14.15%), *Caenorhabditis brenneri* CB5161 (1,630, 10.05%), *Brugia malayi* (1,518, 9.36%) and *C*. *remanei* (1,501, 9.25%); the top 5 plant parasitic nematodes were *B*. *xylophilus* (94 transcripts, 0.58%), *H*. *glycines* (38, 0.23%), *M*. *incognita* (29, 0.18%), *D*. *destructor* (14, 0.09%) and *B*. *mucronatus* (10, 0.06%) (Fig 1 and Table D in S1 File).

**Fig 2:**
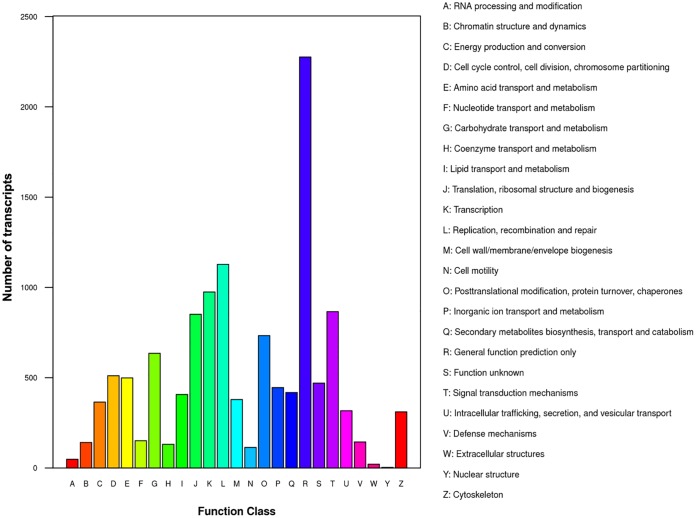
COG function classification of transcripts in a mixed-stage population of *Aphelenchoides ritzemabosi*. The x-axis shows the COG function classes, and the y-axis shows the number of transcripts in one class. The notation on the right shows the full names of the function classes.

**Fig 3:**
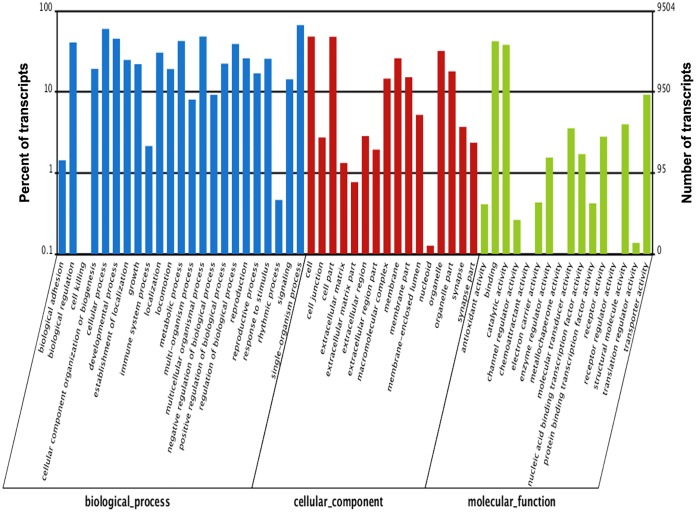
GO classification analysis of transcripts in a mixed-stage population of *Aphelenchoides ritzemabosi*. GO functions are shown on the x-axis. The right side of the y-axis shows the number of genes with the GO function, and the left side shows the percentage.

In the COG database, the largest category of annotated CFN transcripts was general function prediction only (R) (2,276 transcripts, 18.45%), followed by replication, recombination and repair (L) (1,128, 9.14%), transcription (K) (975, 7.90%), signal transduction mechanisms (T) (866, 7.02%), and translation, ribosomal structure and biogenesis (J) (851, 6.90%) (Fig 2 and S3 File). Annotation of the CFN transcripts with the GO database classified the transcripts into 55 small classes in three ontologies: molecular function, cellular component and biological process. The largest class of transcripts in the molecular function ontology was binding activity, which constituted 42.16% (4,007) of the transcripts. In the cellular component ontology, the largest class of transcripts (4,573, 48.12%) was in cell. In the biological process ontology, the largest class was single-organism processes, which constituted 66.62% (6,332) of the transcripts (Fig 3 and S4 and S5 Files).

**Fig 4:**
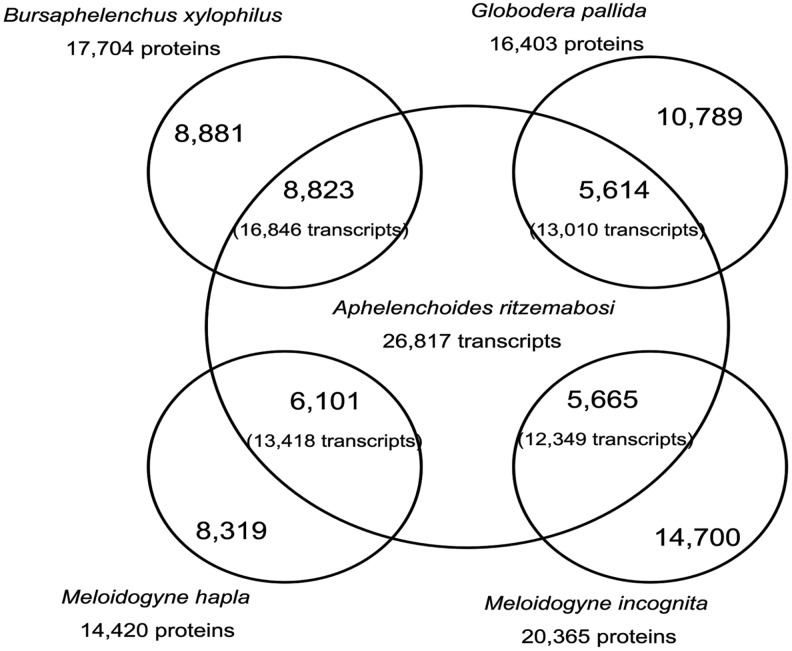
*Aphelenchoides ritzemabosi* orthologues present in four selected completely sequenced genomes of plant nematodes, *Bursaphelenchus xylophilus*, *Globodera pallida*, *Meloidogyne hapla* and *M*. *incognita*. The numbers in parentheses represent the common proteins.

The result of CFN transcripts orthologues present in four selected completely sequenced genomes of *B*. *xylophilus*, *G*. *pallida*, *M*. *hapla* and *M*. *incognita* was showed in Fig 4. In the total of 26,817 CFN transcripts, 16,846 (62.82%), 13,010 (48.51%), 13,418 (50.04%) and 12,349 (46.05%) transcripts, respectively, matched to 8,823 (49.84%) proteins of *B*. *xylophilus*, 5,614 (34.23%) proteins of *G*. *pallida*, 6,101 (42.31%) proteins of *M*. *hapla* and 5,665 (27.82%) proteins of *M*. *incognita*. In addition, 10,543 (39.31%) CFN transcripts matched sequences of all of four plant parasitic nematodes compared.

### Metabolic pathway analysis

To further survey the pathways of CFN, the CFN transcripts were annotated with the KEGG database. A total of 11,645 transcripts were mapped to 259 KEGG pathways, including metabolic pathways (1,702 transcripts, 14.62%), lysosome (477, 4.1%), focal adhesion (472, 4.05%), pathways in cancer (468, 4.02%), and regulation of actin cytoskeleton (456, 3.92%) (S6 File). In addition, 25 signaling pathways and 5 secretion pathways were predicted in the CFN transcriptome analysis. A group of important signaling pathways predicted in the CFN was orthologues with those of other nematodes; the largest group of these transcripts (345, 2.96%) was in the calcium signaling pathway (Table 1). These signaling pathways play important roles in the growth and development of nematodes [34,35].

**Table 1:** Important signaling pathways in the mixed-stage population of *Aphelenchoides ritzemabosi*.

Pathway ID	Pathway	Number of transcripts
ko04350	TGF-beta signaling pathway	99 (0.85%)
ko04020	Calcium signaling pathway	345 (2.96%)
ko04010	MAPK signaling pathway	293 (2.52%)
ko04910	Insulin signaling pathway	264 (2.27%)
ko04310	Wnt signaling pathway	245 (2.1%)

### CDS prediction, SSRs and SNP distribution

A total of 20,311 protein-coding transcripts were annotated after the CFN transcripts were aligned to protein databases by BLASTX with an E-value of 1e-5. 16,293 transcripts were mapped to the protein database, the sequence direction (5’->3’) of these transcripts was determined using the prioritization order NR, Swiss-Prot, KEGG and COG and the coding region sequences were translated into amino sequences with the standard codon table (Figs D-E in S1 File). 4,018 transcripts which cannot be aligned to any database were predicted by ESTScan to produce nucleotide sequence (5’->3’) and amino sequence of the predicted coding region (Figs F-G in S1 File). Most (> 90%) of the CDS protein sequences were less than 700 amino acids long (Figs E, G in S1 File).

**Fig 5:**
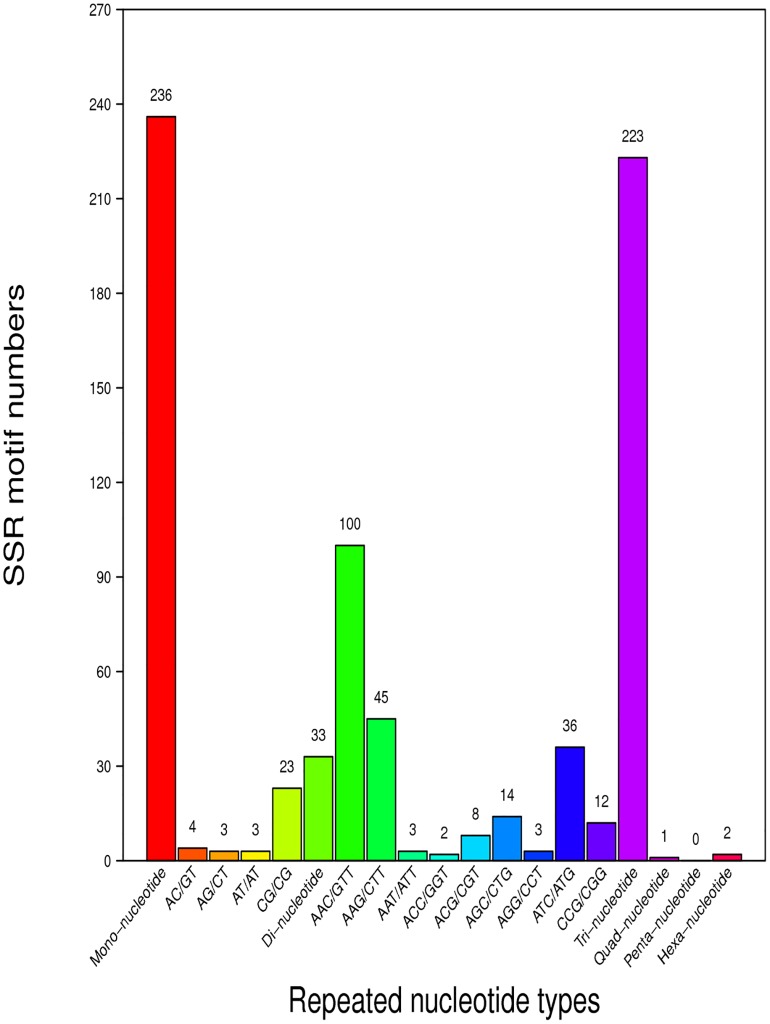
Statistics for simple sequence repeat (SSR) classification in the mixed-stage population of *Aphelenchoides ritzemabosi*. The x-axis shows the number of repeats of a repeat unit. The y-axis shows the number of SSRs.

**Fig 6:**
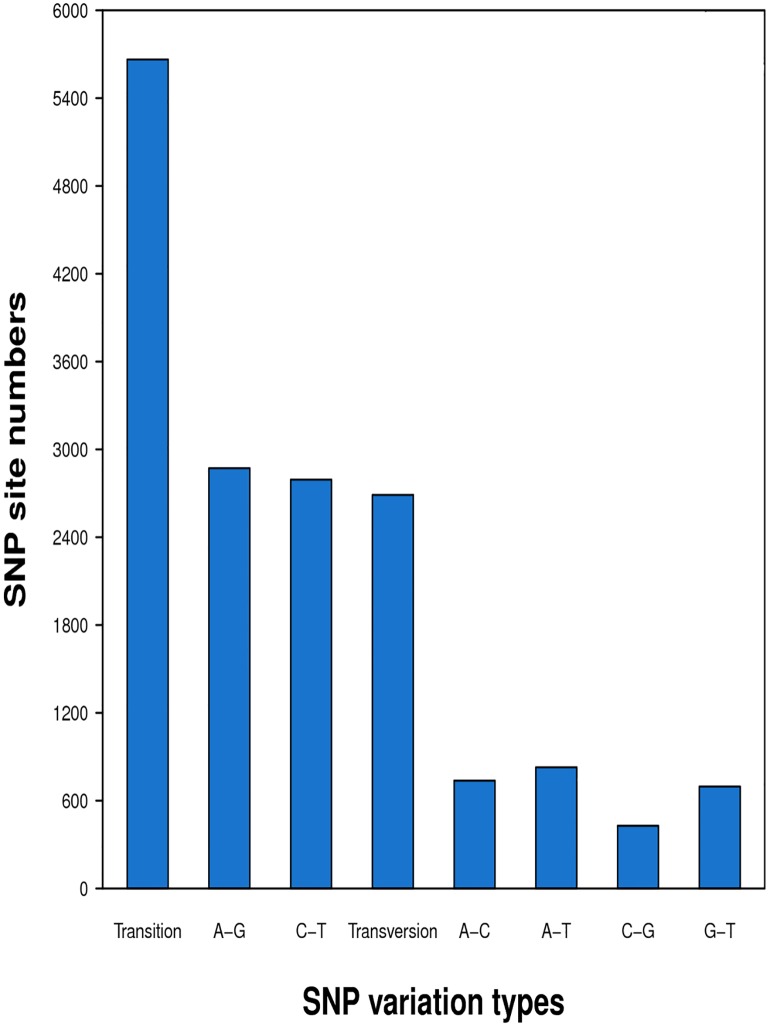
Statistics for single-nucleotide polymorphisms (SNP) in the mixed-stage population of *Aphelenchoides ritzemabosi*. The x-axis shows the SNP types. The y-axis shows the number of SNPs.

Approximately 1.85% (495) of the CFN transcripts were predicted as SSRs. The most frequent types of SSRs were mononucleotide (236 SSRs, 47.68%), followed by trinucleotide (223, 45.05%) and dinucleotide (33, 6.67%). Only a few tetranucleotide (1, 0.20%) and hexanucleotide (2, 0.40%) SSRs and no pentanucleotide SSRs were predicted in the CFN transcripts (Fig 5). A total of 8,353 SNPs were detected; the most and least common types were transition (5,664 SNPs, 67.81%) and C-G (428, 0.05%), respectively (Fig 6).

### CAZymes and cell wall degradation

A group of CAZymes were detected in the CFN transcriptome analysis. In total, 1,199 transcripts were assigned to six classes of CAZymes (Table E). Some transcripts were assigned to more than one related protein type (S7 File). A total of 111 CFN transcripts were predicted as homologues of 7 types of CAZy enzyme with plant/fungal cell wall-degrading activities. The putative plant/fungal cell wall-degrading enzymes of CFN were compared with those of other nematodes shown in Table 2. Fewer transcripts were predicted as homologues of plant cell wall-degrading enzymes than fungal cell wall-degrading enzymes. In CFN, 13 transcripts were predicted as homologues of 3 types with plant cell wall-degrading activities, including 4 transcripts predicted as homologues of GH5 proteins, 7 to GH45 proteins and 2 to GH43 proteins. GH43 proteins had been predicted only in *M*. *incognita*, *M*. *hapla* and *P*. *thornei* [12,16]. GH45 proteins had been predicted only in *B*. *xylophilus*, *A*. *besseyi* and *P*. *thornei* [12,16,18]. GH28 proteins, which are present in other nematodes, were not predicted in CFN. A total of 98 transcripts of the CFN transcriptome were predicted as homologues of 4 types with fungal cell wall-degrading activity, including 68, 11, 4 and 15 transcripts predicted as homologues of GH18, GH19 and GH20 proteins and GH16 proteins, respectively. In addition to the CFN transcriptome, GH16 1,3-glucanase had been predicted in *B*. *xylophilus*, *A*. *besseyi* and *P*. *coffeae* [11,16,18] and GH5, GH18, GH19 and GH20 proteins had been predicted in free-living, plant- and animal-parasitic nematodes; GH5 and GH18 proteins are particularly common [12,16,18,36–38].

**Table 2:** *Aphelenchoides ritzemabosi* enzymes with predicted plant/fungal cell wall-degrading activities, compared with those in other nematodes.

Substrate	Cellulose		Arabinan	Pectin	1,3-glucan	Chitin		
Family	GH5	GH45	GH43	GH28	GH16	GH18	GH19	GH20
*Aphelenchoides rizemabosi*	4	7	2	0	15	68	11	4
*Aphelenchoides fragariae*	1	0	0	0	0	0	0	0
^a^*Aphelenchoides besseyi*	-	+	-	-	+	-	-	+
*Aphelenchus avenae*	4	0	0	0	0	0	0	0
^b^*Bursaphelenchus xylophilus*	0	11	0	0	6	9	2	7
*Globodera pallida*	5	0	0	0	0	0	0	0
*Globodera rostochiensis*	4	0	0	0	0	0	0	0
*Heterodera glycines*	15	0	0	0	0	2	0	0
^b^*Meloidogyne incognita*	21	0	2	2	0	3	2	2
^b^*Meloidogyne hapla*	6	0	2	2	0	4	0	1
^c^*Nacobbus aberrans*	0	0	0	0	0	0	0	0
^d^*Pratylenchus coffeae*	+	-	-	-	+	-	-	-
^e^*Pratylenchus thornei*	2	1	1	1	0	11	2	3
*Radopholus similis*	1	0	0	0	0	0	0	0
*Rotylenchulus reniformis*	1	0	0	0	0	0	0	0
*Ascaris suum*	0	0	0	0	0	4	2	4
^b^*Pristionchus pacificus*	6	0	0	0	0	9	2	5
*Caenorhabditis elegans*	0	0	0	0	0	44	7	5

^a^ Data of *A*. *besseyi* transcriptome were obtained from Kikuchi et al. and Wang et al. [17,18],

^b^ Data of *B*. *xylophilus* whole genome were obtained from Kikuchi et al. [16],

^c–e^Data of *N*. *aberrans*, *P*. *coffeae* and *P*. *thornei* were obtained from Eves-van den Akker et al., Haegeman et al. and Nicol et al. [11,12,14]. Other data were obtained from http://mothra.ornl.gov/cgi-bin/cat/cat.cgi?tab=CAZymes.

Four phylogenetic trees were generated using the neighbor-joining method based on the amino acid sequences of GH5, GH16, GH43 and GH45 proteins (Figs H-K in S1 File). Thirty GH5 proteins from 27 different species were divided 7 groups, GH5 proteins of CFN, *A*. *avenae*, *A*. *besseyi* and *A*. *fragariae* were present in the branch of Aphelenchoidinae, suggesting a close phylogenetic relationship (Fig H in S1 File). Twenty GH16 proteins from 19 different species were divided 4 groups, GH16 proteins of CFN and *B*. *xylophilus* were present in the branch of Nematoda, suggesting a close phylogenetic relationship (Fig I in S1 File). Ten GH43 proteins from 10 different species were divided 4 groups, GH43 protein of CFN was present in the branch of Nematoda (Fig J in S1 File). Thirteen GH45 proteins from 9 different species were divided into three groups. GH45 proteins of CFN, *A*. *besseyi*, *B*. *xylophilus* and *B*. *mucronatus* were present in the branch of Nematoda, suggesting a close phylogenetic relationship (Fig K in S1 File).

### Transcripts involved in the RNAi pathway

The CFN transcripts predicted as orthologues of the genes involved in the RNAi pathway were displayed in Table 3, Table F in S1 File, along with the presence of these genes in *C*. *elegans* (www.wormbase.org), *A*. *besseyi*, *B*. *xylophilus*, *M*. *incognita* and *P*. *coffeae* [11,16,18,33]. Sixteen types of genes in the CFN transcriptome were predicted as orthologues of 7 of 8 gene families compared with *C*. *elegans* RNAi pathway. RNAi suppressor genes were predicted in *M*. *incognita* and *P*. *coffeae* but not in CFN, *A*. *besseyi* and *B*. *xylophilus*. In addition, the mut-7 gene and vig-1 gene, which are Exo-RNAi and miRNA or endo RNAi genes, were predicted in CFN, *A*. *besseyi* and *B*. *xylophilus* but not in *M*. *incognita* and *P*. *coffeae*. The CFN transcriptome encodes fewer predicted orthologues of RNAi pathway genes than *A*. *besseyi* and *M*. *incognita* [11,16,18,33].

**Table 3:** *Aphelenchoides ritzemabosi* transcripts involved in the RNAi pathway, compared with those in other nematodes.

Function in RNAi	*Caenorhabditis elegans*	*Meloidogyne incognita*	*Pratylenchus coffeae*	*Bursaphelenchus xylophilus*	*Aphelenchoides besseyi*	*Aphelenchoides rizemabosi*
Exo-RNAi	drh-1, drh-2, mut-7, mut-16, rde-2, rde-3, rde-4, smg-2, smg-5, zfp-1	drh-1,drh-2, smg-2, rde-3	drh-1, smg-2	drh-1, mut-7, rde-4, smg-2	drh-1, mut-7, mut-16, rde-3, smg-2, zfp-1	drh-1, mut-7, rde-4, smg-2,
Dicer	dcr-1	dcr-1	dcr-1	dcr-1	dcr-1	dcr-1
Amplification	ego-1, rrf-1, rrf-2, rrf-3	ego-1,rrf-1, rrf-2, rrf-3	ego-1	ego-1, rrf-1, rrf-3	ego-1, rrf-1, rrf-3	ego-1, rrf-3
Argonautes	PPW-1, PPW-2, SAGO-1, SAGO-2,rde-1	PPW-1, PPW-2, rde-1, SAGO-1, SAGO-2,	PPW-2	rde-1	PPW-1, PPW-2, SAGO-2	PPW-2, SAGO-1
RNAi suppressor	eri-1, eri-3, eri-5	eri-1	eri-1	-	-	-
RNAi enhancer	gfl-1	gfl-1	gfl-1	gfl-1	gfl-1	gfl-1
Uptake	rsd-2, rsd-3, rsd-6, sid-1, sid-2	rsd-3	-	rsd-6	rsd-3	rsd-3, rsd-6
miRNA or endo RNAi	alg-1, alg-2, drsh-1, ERGO-1, PRG-1, PRG-2, tsn-1, vig-1	alg-1, alg-2, drsh-1, tsn-1	alg-1,alg-2, drsh-1, tsn-1	alg-1, alg-2, drsh-1, tsn-1, vig-1	alg-1, alg-2, drsh-1, ERGO-1, PRG-1, PRG-2, tsn-1, vig-1	alg-1, drsh-1, tsn-1, vig-1

The proteins involved in the RNAi pathway of *C*. *elegans*, *M*. *incognita*, *P*. *coffeae*, *B*. *xylophilus* and *A*. *besseyi* were obtained from www.wormbase.org, Rosso et al., Haegeman et al., Kikuchi et al. and Wang et al., respectively [11, 16, 18, 33].

## Discussion

In this study, the transcriptome of mixed-stage population of CFN was investigated using the Illumina Hiseq 2000 system. A total of 26,817 transcripts with a mean length of 1,032 bp and an N50 of 1,672 bp were generated, and 16,467 transcripts (61.41%) were annotated against six databases. The unannotated sequences might be sequences specific to this species and require further research. Statistical analysis of CFN transcripts based on the top BLASTX hits in the NR database showed that the top organisms and plant parasitic nematodes were *L*. *loa* (3,440, 21.20%) and *B*. *xylophilus* (94, 0.58%), respectively. In the CFN transcriptome, a total of 1,199 transcripts were assigned to six classes of CAZymes containing 130 related protein types, and sixteen types of genes in the CFN transcriptome were predicted as orthologues of 7 of 8 gene families compared with *C*. *elegans* RNAi pathway.

Among foliar nematodes, only the transcriptome of *A*. *besseyi* had been studied with NGS [17,18]. Wang et al. studied a mixed-stage population of *A*. *besseyi* and obtained 51,270 transcripts with a mean length of 1,241 bp, 524 transcripts were assigned to the four classes of CAZymes: GHs, GTs, CEs and CBMs, annotating 57 RNAi pathway genes [18]. Kikuchi et al. performed a small-scale transcriptome sequencing project for *A*. *besseyi*, obtaining 5,804 transcripts with a mean length of 828 bp [17]. Among nematodes that parasitize above-ground plant parts, Kikuchi et al. conducted genome sequencing of *B*. *xylophilus*, identified a total of 58 transcripts predicted as homologues of 8 families of CAZymes and annotated potential 78 RNAi pathway genes in the *B*. *xylophilus* genome [16].

A systematic evolutionary study of plant cell wall-modifying genes in Tylenchoidea concluded that genes acquired by multiple horizontal gene transfer (HGT) events from bacteria or fungi were closely associated with the ancestors of these nematodes [39,40]. CFN can feed on a variety of fungi and is closely associated with fungi [41, 42]. GH5 protein had been predicted in many plant parasitic tylench nematodes, including *Meloidogyne*, *Globodera*, *Heterodera*, *Hirschmanniella* and *Pratylenchus* [16]. Most interestingly, GH45 protein genes were present only in CFN, *A*. *besseyi*, *B*. *xylophilus* and *P*. *thornei*; these genes are most homologues of those from fungi and are hypothesized to have been acquired by HGT from fungi [16,18,43]. The phylogenetic analyses [16,44] suggested that these proteins were not closely related to those from tylench nematodes and were likely acquired independently from different sources.

CAZymes that degrade plant cell walls and potentially degrade fungal cell walls are present in the CFN transcriptome, and more transcripts were predicted as homologues of fungal cell wall-degrading CAZymes than as homologues of plant cell wall-degrading CAZymes (Table 3). In addition, more chitin-related CAZymes were predicted in the CFN transcriptome than in other plant parasitic nematodes. Chitin is one of the main components of fungal cell walls, and the greater number of chitin-related CAZymes in CFN may be related to the ability of CFN to feed on a variety of plant tissues and fungi. GH18, GH19 and GH20 proteins had been reported in nematodes with chitin-degrading CAZymes, with GH18 protein the most abundant of these three chitin-related CAZymes (Table 3). More orthologues of GH18 protein were predicted in the CFN transcriptome compared to other nematodes, and migratory plant-parasitic nematodes encode more GH18 protein than sedentary plant-parasitic nematodes, indicating that GH18 protein may have special biological significance in the nematode life cycle and in the evolution of nematodes from a free-living to parasitic lifestyle. Fifteen GH16 proteins were predicted in the CFN transcriptome, and 1,3-glucan is another core component of the fungal cell wall. GH16 protein had also been predicted in *A*. *besseyi*, *B*. *xylophilus* and *P*. *coffeae* [11,16–18]. GH16 protein is most homologues of genes from bacteria, possibly indicating that these genes were acquired from bacteria that were closely associated with the ancestors of these nematodes [16,45].

This research confirms that aphelench nematodes, CFN, *A*. *besseyi* and *B*. *xylophilus* exhibit orthologues in the genes involved in the RNAi pathway but differ from plant parasitic tylench nematodes because they have mut-7 and vig-1 genes and no RNAi suppressor genes, whereas tylench nematodes, *M*. *incognita* and *P*. *coffeae* have RNAi suppressor genes and no mut-7 and vig-1 genes [11,16,18,31]. CFN, *A*. *besseyi* and *B*. *xylophilus* are aerial plant parasitic aphelench nematodes, have higher adaptability to low humidity and shorter life cycle than underground plant parasitic tylench nematodes, and can complete their life cycle by feeding on fungi [3]. *M*. *incognita* and *P*. *coffeae* are obligate parasitic nematodes living on the underground parts of plants and feed only on plant tissues. Therefore, there is difference in the parasitic strategies, feeding behavior and niches occupied between aphelench and tylench nematodes. But systematic and comprehensive studies are necessary to determine whether there is relationship between RNAi gene components and parasitic strategies, feeding behavior and niches occupied.

## Conclusions

In conclusion, we have performed the first *de novo* analysis of the CFN transcriptome, thus increasing the understanding of the molecular biology of foliar nematodes. These results can be used to select interesting genes of possible importance in plant parasitism for new functional studies. Additionally, this transcriptomic information should be a valuable resource for future research on novel strategies for controlling CFN.

## Supporting Information

S1 File Fig A. The length distribution of Contigs. Fig B. The length distribution transcripts. Fig C. Randomicity of sample CFN reads. Fig D. Length distributions of CDS nucleotide sequence. Fig E. Length distributions of CDS protein sequence. Fig F. Length distributions of CDS nucleotide sequence with ESTScan. Fig G. Length distributions of CDS protein sequence with ESTScan. Fig H. The polygenetic tree of GH5 protein amino acid sequence of *Aphelenchoides ritzemabosi* and other organisms. Fig I. The polygenetic tree of GH16 protein amino acid sequence of *Aphelenchoides ritzemabosi* and other organisms. Fig J. The polygenetic tree of GH43 protein amino acid sequence of *Aphelenchoides ritzemabosi* and other organisms. Fig K. The polygenetic tree of GH45 protein amino acid sequence of *Aphelenchoides ritzemabosi* and other organisms. Table A. Output statistics of sequencing. Table B. Statistics of assembly quality. Table C. Statistics of annotation results. Table D. Statistics of Nr annotation species distribution of *A*. *ritzemabosi*. Table E. Carbohydrate-active enzymes identified in the transcriptome analysis of *Aphelenchoides ritzemabosi*. Table F. The transcripts involved in the RNAi pathway, annotated in the transcriptomic analysis of *A*. *ritzemabosi*.
(DOC)

S2 File All annotation of *A*. *ritzemabosi* in five databases. (XLS)

S3 File YK-transcript.fa.cog.class.annot. (XLS)

S4 File GO annotations for each YK-transcript. (XLS)

S5 File List of YK-transcript in each GO category. (XLS)

S6 File Web page report of All-transcript Parhway annotation. (DOC)

S7 File Annotation of carbohydrate-active enzymes. (XLS)
