# Supplementary material for: Transcriptome Analysis of the Chrysanthemum Foliar Nematode, Aphelenchoides ritzemabosi (Aphelenchida: Aphelenchoididae)
Source: PLoS One. 2016 Nov 22;11(11):e0166877. doi: 10.1371/journal.pone.0166877 (PMC5119785; doi:10.1371/journal.pone.0166877)
Supplement: S1 File — Fig A. The length distribution of Contigs. Fig B. The length distribution transcripts. Fig C. Randomicity of sample CFN reads. Fig D. Length distributions of CDS nucleotide sequence. Fig E. Length distributions of CDS protein sequence. Fig F. Length distributions of CDS nucleotide sequence with ESTScan. Fig G. Length distributions of CDS protein sequence with ESTScan. Fig H. The polygenetic tree of GH5 protein amino acid sequence of Aphelenchoides ritzemabosi and other organisms. Fig I. The polygenetic tree of GH16 protein amino acid sequence of Aphelenchoides ritzemabosi and other organisms. Fig J. The polygenetic tree of GH43 protein amino acid sequence of Aphelenchoides ritzemabosi and other organisms. Fig K. The polygenetic tree of GH45 protein amino acid sequence of Aphelenchoides ritzemabosi and other organisms. Table A. Output statistics of sequencing. Table B. Statistics of assembly quality. Table C. Statistics of annotation results. Table D. Statistics of Nr annotation species distribution of A. ritzemabosi. Table E. Carbohydrate-active enzymes identified in the transcriptome analysis of Aphelenchoides ritzemabosi. Table F. The transcripts involved in the RNAi pathway, annotated in the transcriptomic analysis of A. ritzemabosi. (DOC) [file pone.0166877.s001.doc]

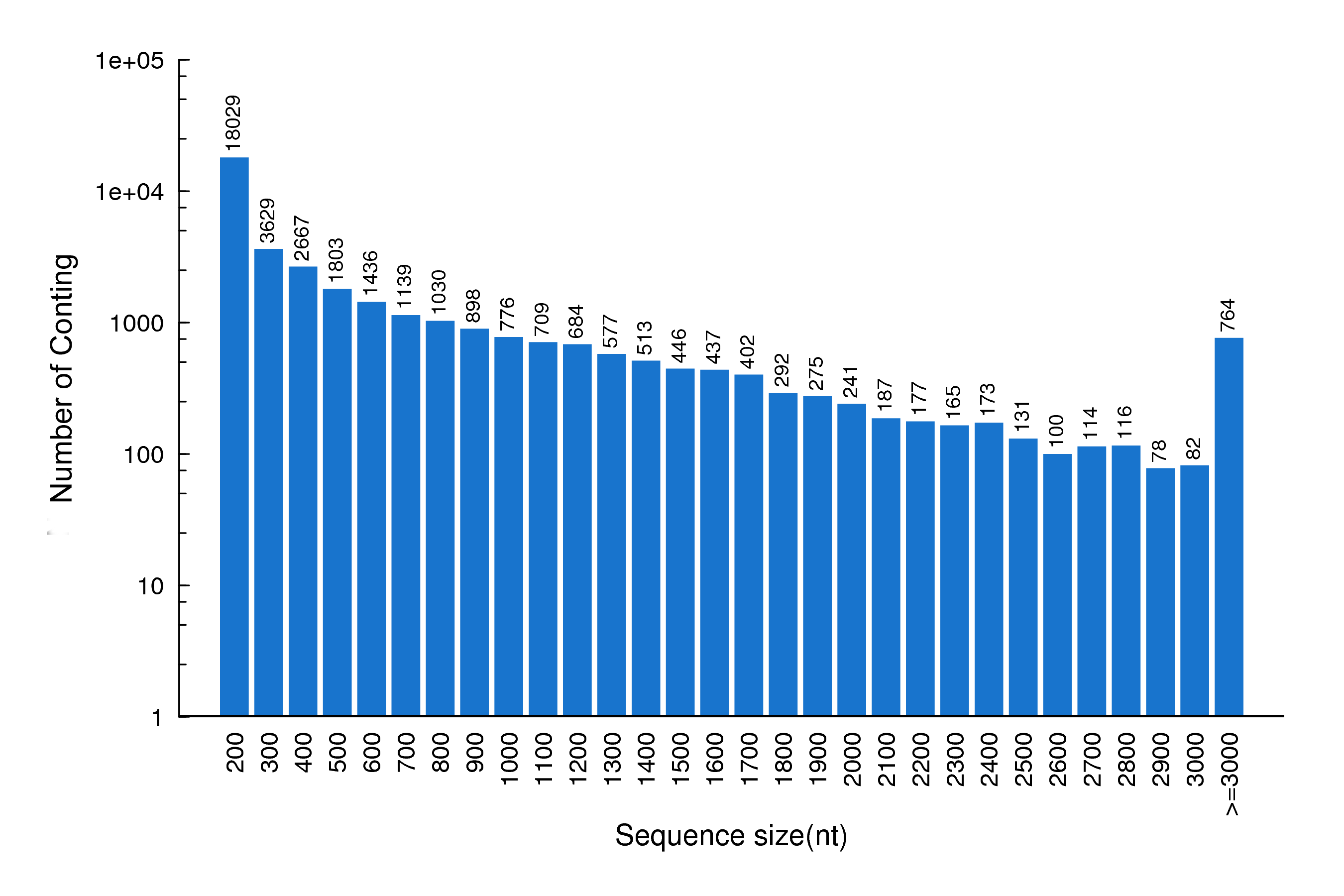


**Fig A. The length distribution of Contigs.**

The horizontal coordinates are Contig lengths and the vertical coordinates are numbers of Contigs.


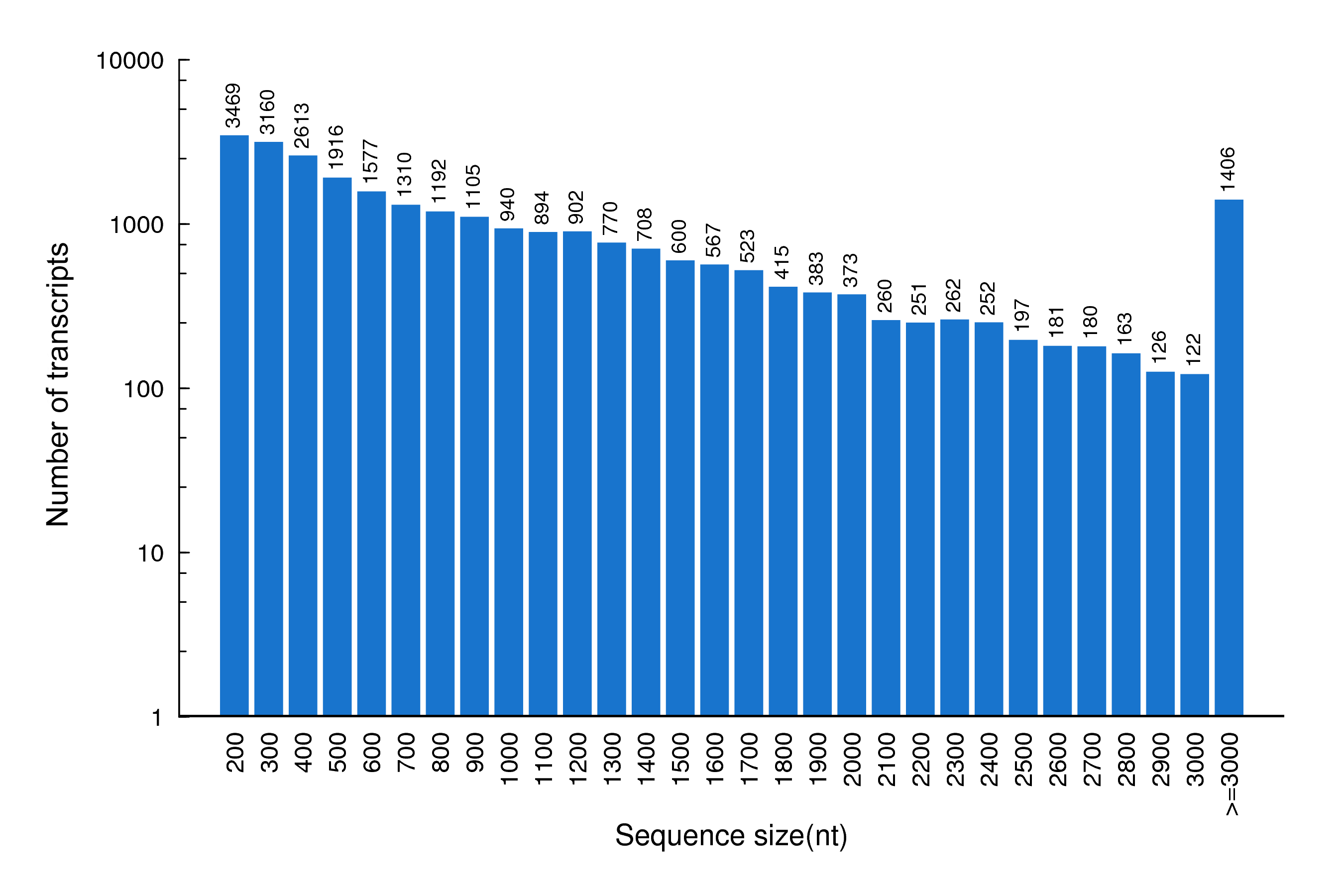


**Fig B. The length distribution transcripts.**

The horizontal coordinates are transcript lengths and the vertical coordinates are numbers of transcripts.


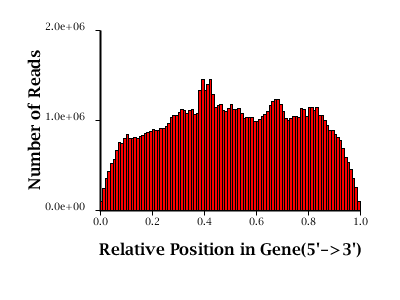


**Fig C. Randomicity of sample CFN reads.**


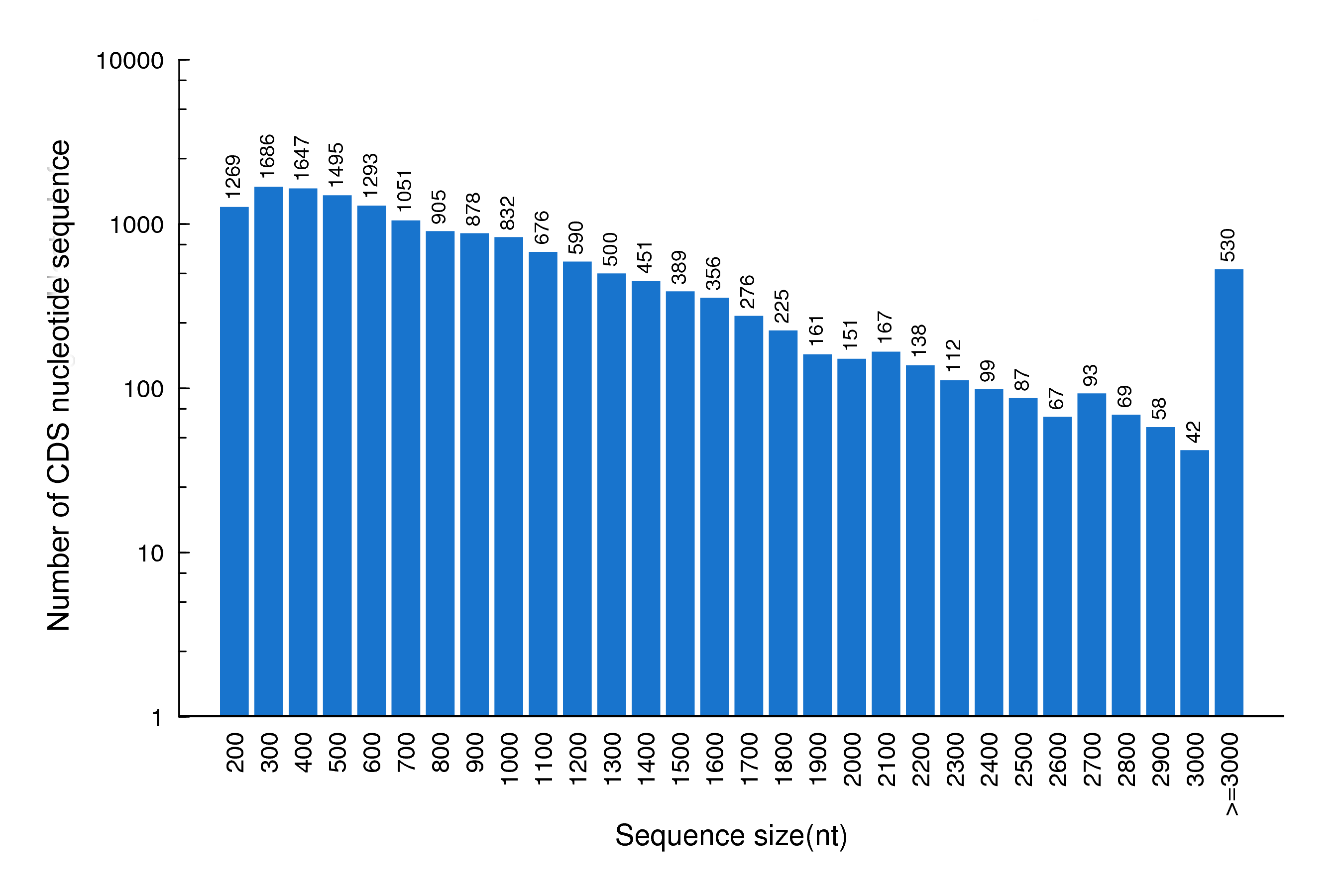


**Fig D. Length distributions of CDS nucleotide sequence.**

The horizontal coordinates are CDS lengths and the vertical coordinates are numbers of CDS.


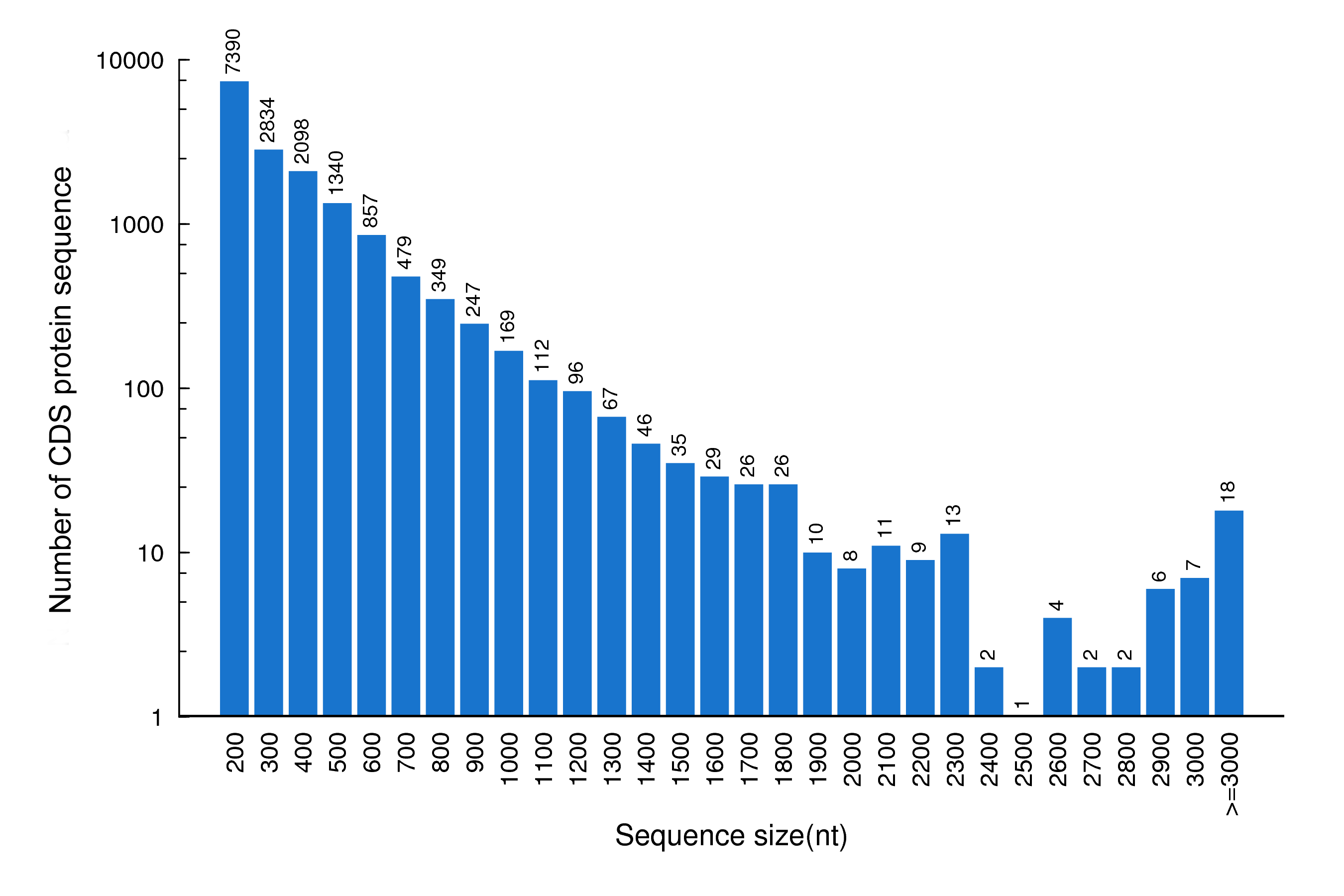


**Fig E. Length distributions of CDS protein sequence**

The horizontal coordinates are CDS lengths and the vertical coordinates are numbers of CDS.


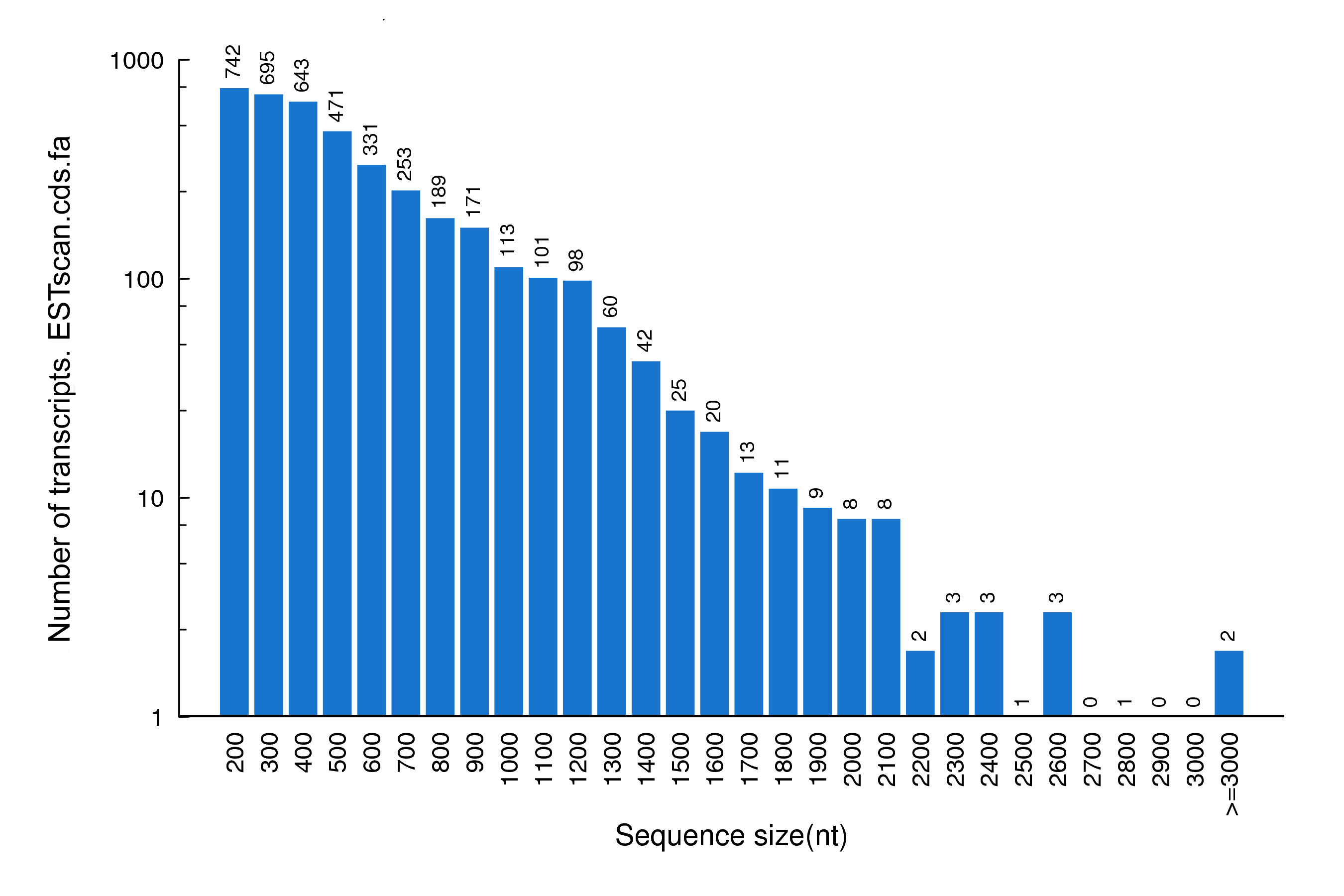


**Fig F. Length distributions of CDS nucleotide sequence with ESTScan.**

The horizontal coordinates are CDS lengths and the vertical coordinates are numbers of CDS.


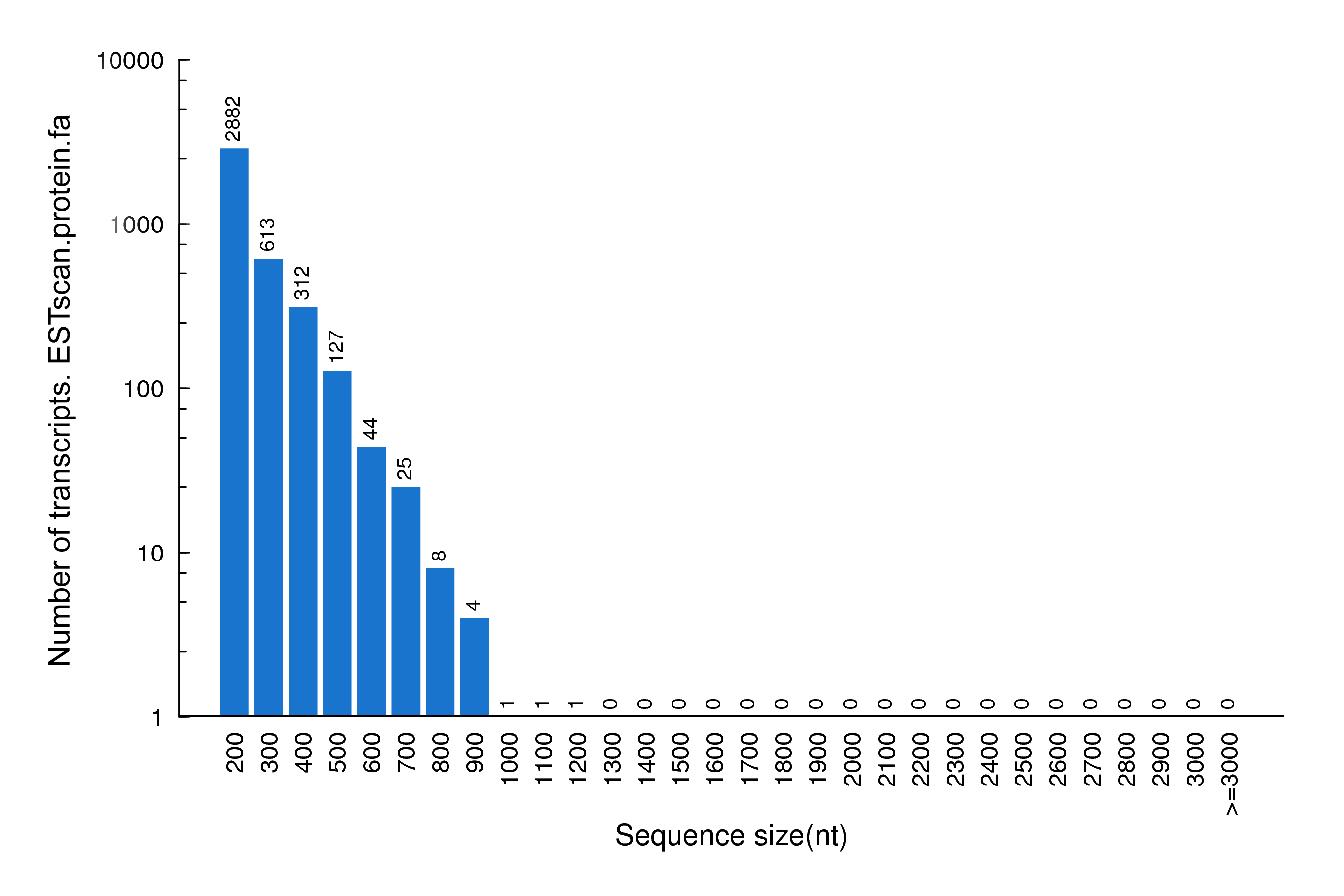


**Fig G. Length distributions of CDS protein sequence with ESTScan.**

The horizontal coordinates are CDS lengths and the vertical coordinates are numbers of CDS.


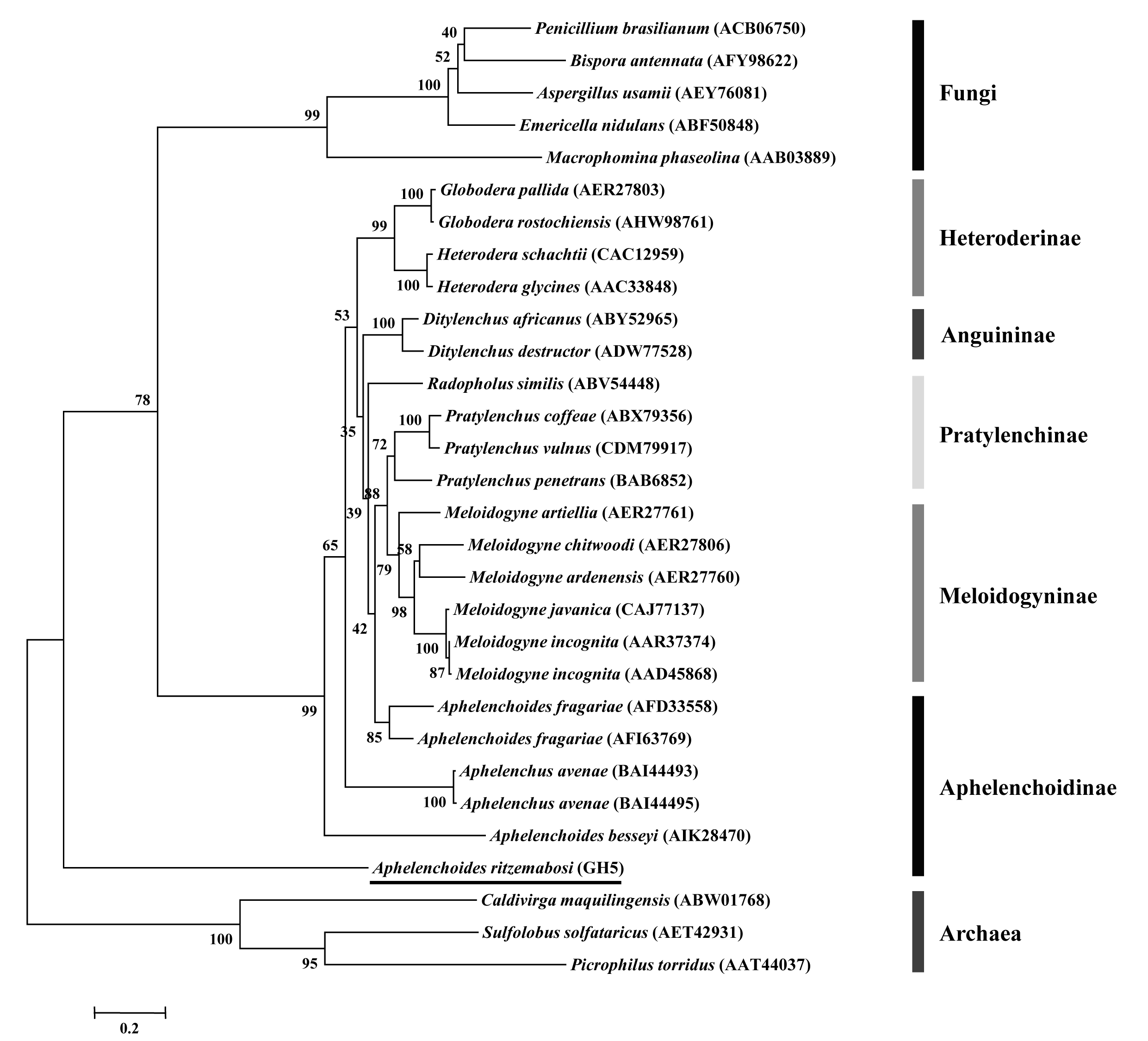


**Fig H. The polygenetic tree of GH5 protein amino acid sequence of *Aphelenchoides ritzemabosi* and other organisms.**

The phylogram was constructed based on amino acid sequences to describe the evolutionary relationships among 29 GH5 proteins from 26 different species with MEGA 5.0, distance scale = 0.2. The numbers below the branches indicate the bootstrap values, which were calculated from 1000 replicates. The *A. ritzemabosi* GH5 proteins was underlined.


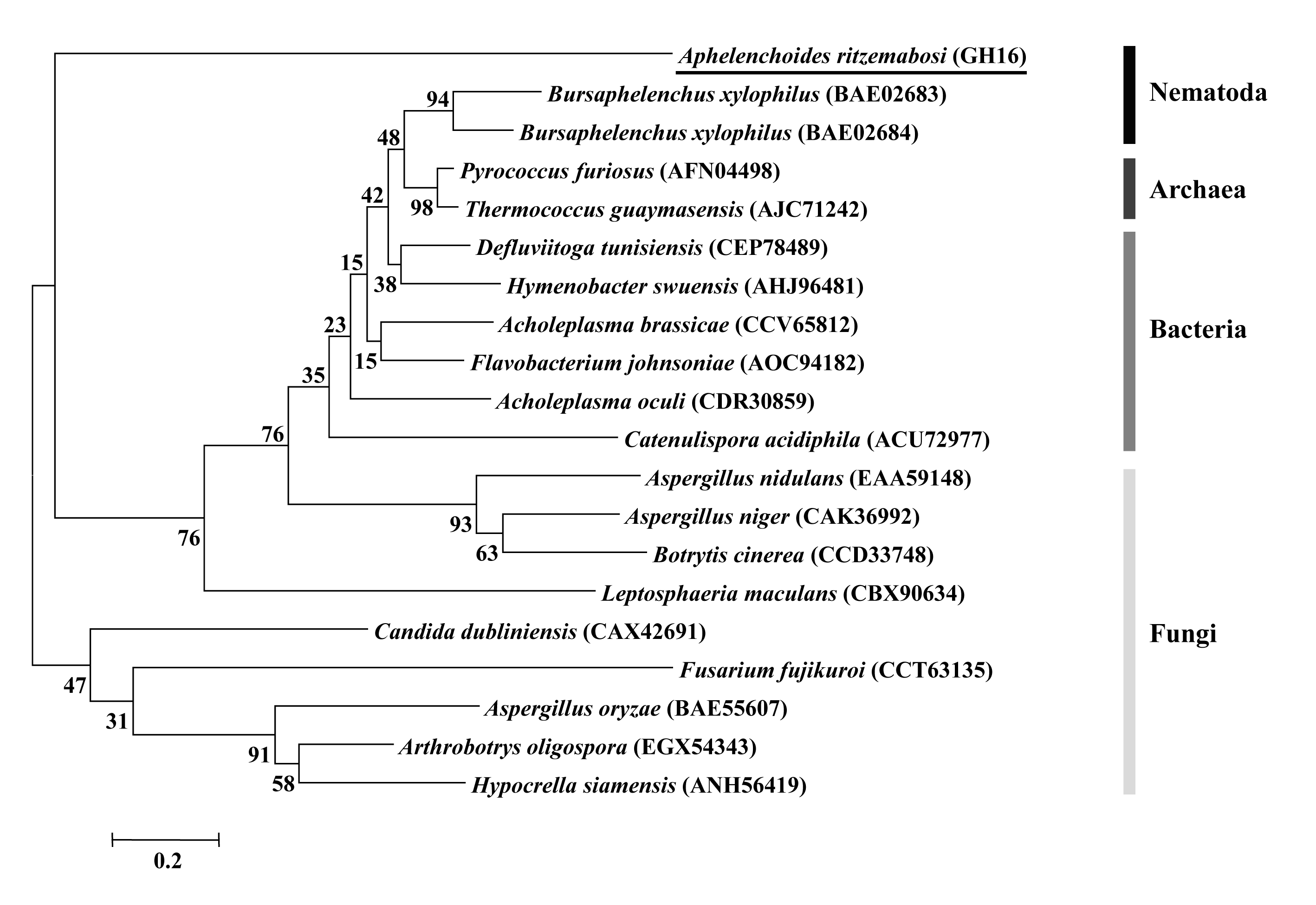


**Fig I. The polygenetic tree of GH16 protein amino acid sequence of *Aphelenchoides ritzemabosi* and other organisms.**

The phylogram was constructed based on amino acid sequences to describe the evolutionary relationships among 19 GH16 proteins from 18 different species with MEGA 5.0, distance scale = 0.2. The numbers below the branches indicate the bootstrap values, which were calculated from 1000 replicates. The *A. ritzemabosi* GH16 proteins was underlined.


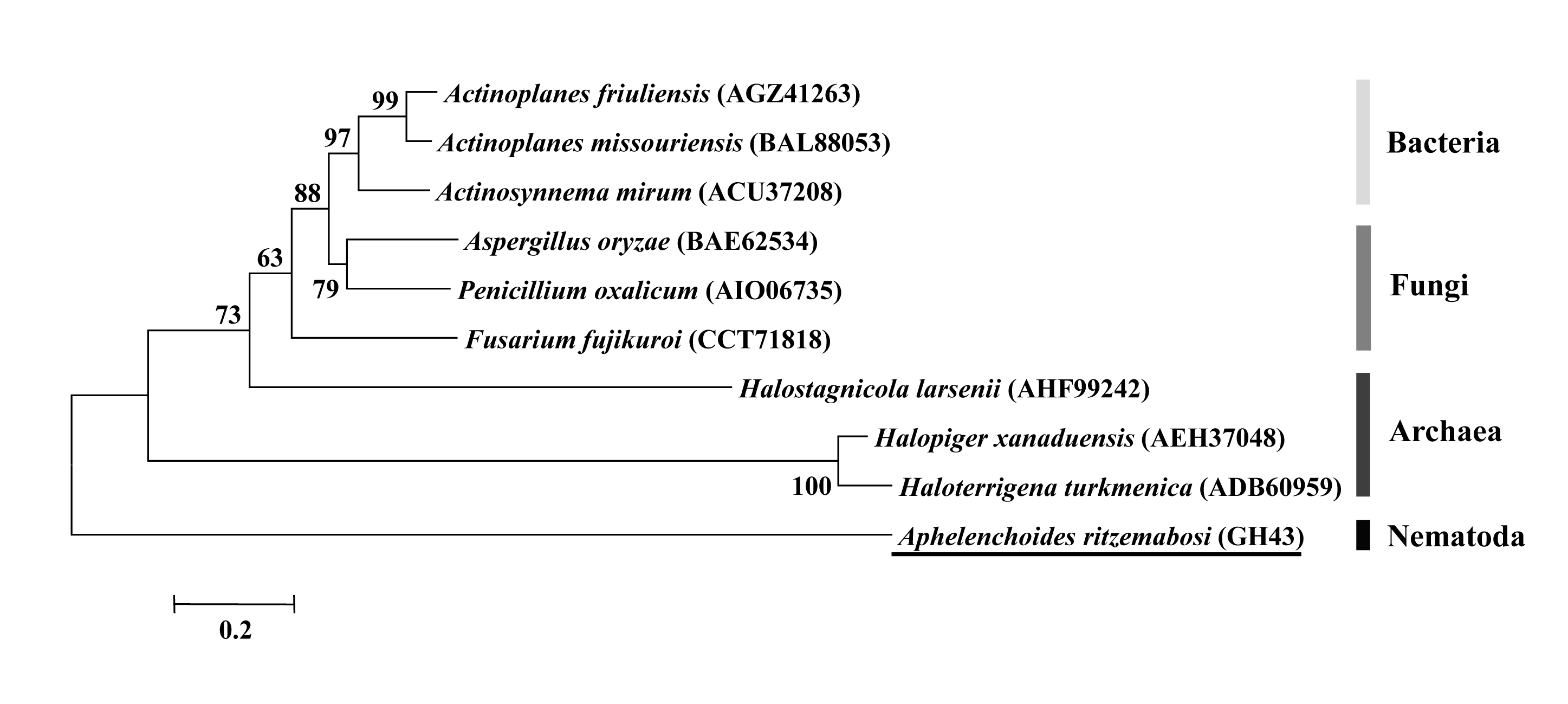


**Fig J. The polygenetic tree of GH43 protein amino acid sequence of *Aphelenchoides ritzemabosi* and other organisms.**

The phylogram was constructed based on amino acid sequences to describe the evolutionary relationships among 9 GH43 proteins from 9 different species with MEGA 5.0, distance scale = 0.2. The numbers below the branches indicate the bootstrap values, which were calculated from 1000 replicates. The *A. ritzemabosi* GH43 proteins was underlined.


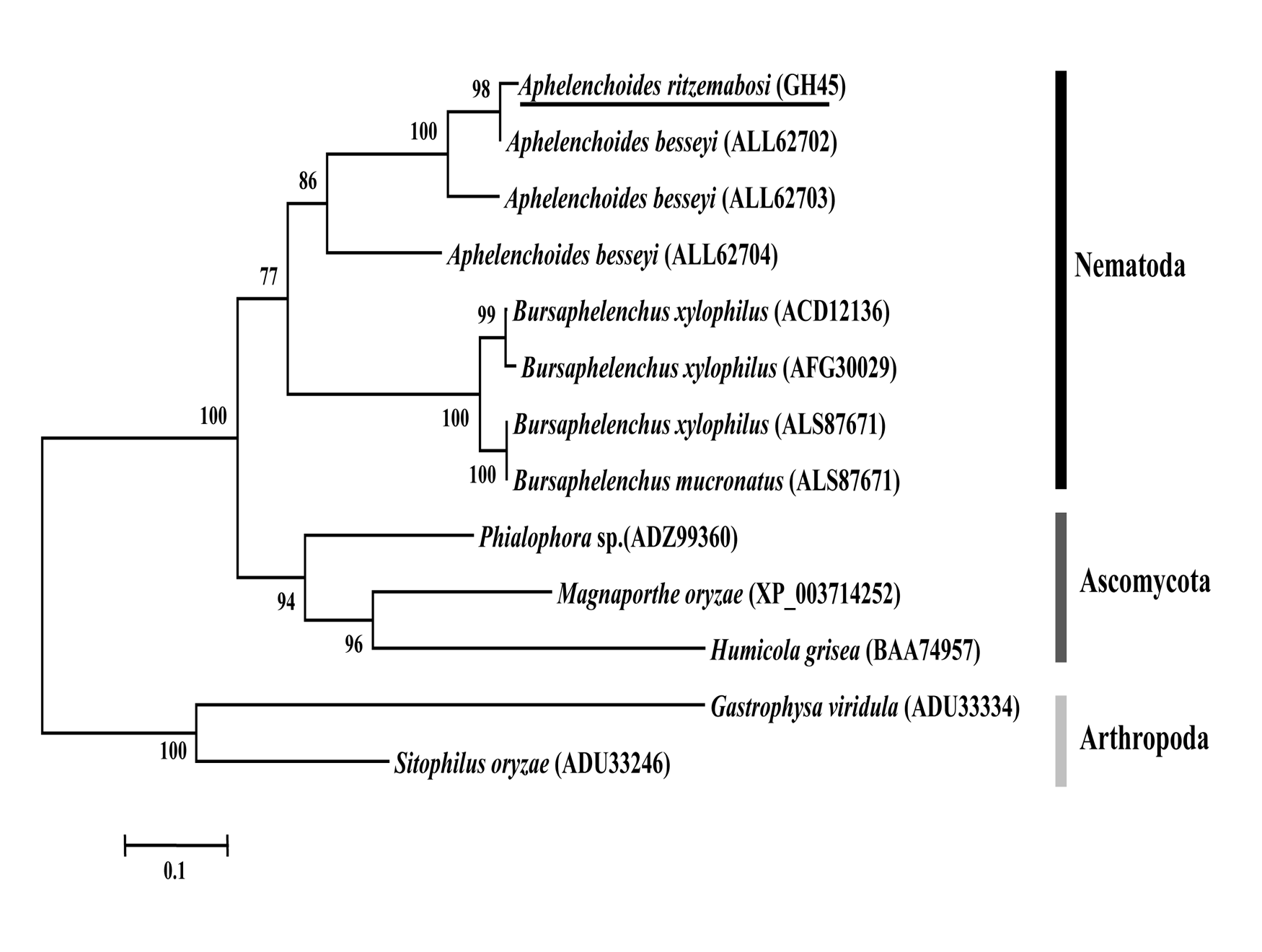


**Fig K. The polygenetic tree of GH45 protein amino acid sequence of *Aphelenchoides ritzemabosi* and other organisms.**

The phylogram was constructed based on amino acid sequences to describe the evolutionary relationships among 13 GH45 proteins from 9 different species with MEGA 5.0, distance scale = 0.1. The numbers below the branches indicate the bootstrap values, which were calculated from 1000 replicates. The *A. ritzemabosi* GH45 protein is underlined.

**Table A. Output statistics of sequencing.**

| Samples | Total Raw Reads | Total Clean Reads | Total Clean Nucleotides (nt) | Q20 percentage | N percentage | GC percentage |
| --- | --- | --- | --- | --- | --- | --- |
| YK | 73,284,868 | 68,103,194 | 6,129,287,460 | 97.40% | 0.00% | 43.05% |

Total Reads and Total Nucleotides are actually clean reads and clean nucleotides; Total Nucleotides should be more than contract provision; Q20 percentage is proportion of nucleotides with quality value larger than 20; N percentage is proportion of unknown nucleotides in clean reads; GC percentage is proportion of guanidine and cytosine nucleotides among total nucleotides. Total Clean Nucleotides = Total Clean Reads1 x Read1 size + Total Clean Reads2 x Read2 size

**Table B. Statistics of assembly quality.**

|  | Sample | Total Number | Total Length(nt) | Mean Length(nt) | N50 | Total Consensus Sequences | Distinct Clusters | Distinct Singletons |
| --- | --- | --- | --- | --- | --- | --- | --- | --- |
| Contig | YK | 38,070 | 21,770,488 | 572 | 1278 | - | - | - |
| Unigene | YK | 26,817 | 27,666,837 | 1032 | 1672 | 26,817 | 7,650 | 19,167 |

Total Consensus Sequences represents the all assembled unigenes; Distinct Clusters represents the cluster unigenes; The same cluster contains some high similar (more than 70%) unigenes, and these unigenes may come from same gene or homologous gene; Distinct Singletons represents this unigene come from a single gene. The length of sequences assmblied is a criterion for assembly success. We calculate the distribution of length of Contigsand Unigenes.

**Table C. Statistics of annotation results.**

| Sequence File | NR | NT | Swiss-Prot | KEGG | COG | GO | ALL |
| --- | --- | --- | --- | --- | --- | --- | --- |
| YK-Unigene.fa | 16,224 | 3,679 | 13,344 | 11,645 | 6,410 | 9,504 | 16,467 |

**Table D. Statistics of Nr annotation species distribution of *A. ritzemabosi***.

| species | gene numbers | percentage | species | gene numbers | percentage |
| --- | --- | --- | --- | --- | --- |
| Loa loa | 3440 | 21.20% | Strongyloides papillosus | 4 | 0.02% |
| Caenorhabditis elegans | 2296 | 14.15% | Neospora caninum Liverpool | 4 | 0.02% |
| Caenorhabditis CB5161 | 1630 | 10.05% | Trypanosoma cruzi | 4 | 0.02% |
| Brugia malayi | 1518 | 9.36% | Scylla paramamosain | 4 | 0.02% |
| Caenorhabditis briggsae | 1501 | 9.25% | Hydra attenuata | 4 | 0.02% |
| Caenorhabditis remanei | 1448 | 8.93% | Leishmania braziliensis MHOM/BR/75/M2904 | 4 | 0.02% |
| Wuchereria bancrofti | 1038 | 6.40% | Cooperia oncophora | 4 | 0.02% |
| Homo sapiens | 111 | 0.68% | Anubis baboon | 4 | 0.02% |
| Trichinella spiralis | 107 | 0.66% | Onchocerca gutturosa | 3 | 0.02% |
| Galendromus occidentalis | 103 | 0.63% | Panagrellus redivivus | 3 | 0.02% |
| Aphelenchoides xylophilus | 94 | 0.58% | Oscheius sp. CEW1 | 3 | 0.02% |
| Capitella sp. I | 82 | 0.51% | Australian ghost shark | 3 | 0.02% |
| Trichomonas vaginalis G3 | 75 | 0.46% | Entamoeba histolytica HM-1:IMSS-B | 3 | 0.02% |
| Daphnia pulex | 74 | 0.46% | Boophilus microplus | 3 | 0.02% |
| Crassostrea gigas | 64 | 0.39% | Dicentrarchus labrax | 3 | 0.02% |
| Haemonchus contortus | 60 | 0.37% | Ictalurus punctatus | 3 | 0.02% |
| Strongylocentrotus purpuratus | 59 | 0.36% | Aquarana catesbeiana | 3 | 0.02% |
| Ascaris suum | 55 | 0.34% | Maconellicoccus hirsutus | 3 | 0.02% |
| Strongyloides ratti | 52 | 0.32% | Tenebrio molitor | 2 | 0.01% |
| Nasonia vitripennis | 51 | 0.31% | Macaca cynomolgus | 2 | 0.01% |
| Saccoglossus kowalevskii | 48 | 0.30% | Plasmodium vivax (STRAIN SAL-I) | 2 | 0.01% |
| Florida manatee | 48 | 0.30% | Oigolaimella attenuata | 2 | 0.01% |
| Tribolium castaneum | 47 | 0.29% | Oesophagostomum dentatum | 2 | 0.01% |
| Amphioxus floridae | 45 | 0.28% | Caligus clemensi | 2 | 0.01% |
| Brachydanio rerio | 45 | 0.28% | Pristionchus aerivora | 2 | 0.01% |
| Nematostella vectensis | 43 | 0.27% | Heterocephalus glaber | 2 | 0.01% |
| Acyrthosiphon pisum | 40 | 0.25% | Entamoeba histolytica KU27 | 2 | 0.01% |
| Megachile rotundata | 38 | 0.23% | Koerneria sp. RS1982 | 2 | 0.01% |
| Heterodera glycines | 38 | 0.23% | Oncorhynchus mykiss | 2 | 0.01% |
| Anopheles gambiae PEST | 36 | 0.22% | Bursaphelenchus doui | 2 | 0.01% |
| Amphimedon queenslandica | 35 | 0.22% | Pantholops hodgsoni | 2 | 0.01% |
| Ixodes dammini | 34 | 0.21% | Plasmodium yoelii yoelii 17XNL | 2 | 0.01% |
| Hydra magnipapillata | 34 | 0.21% | Cyathostomum catinatum | 2 | 0.01% |
| Proterospongia sp. ATCC 50818 | 33 | 0.20% | Haliotis diversicolor | 2 | 0.01% |
| Steinernema carpocapsae | 32 | 0.20% | Drosophila simulans | 2 | 0.01% |
| Gorilla gorilla gorilla | 29 | 0.18% | Pagothenia bernacchii | 2 | 0.01% |
| Bombyx mori | 29 | 0.18% | Trichostrongylus vitrinus | 2 | 0.01% |
| African clawed frog | 29 | 0.18% | Ichthyophthirius multifiliis | 2 | 0.01% |
| Meloidogyne incognita | 29 | 0.18% | Acanthocheilonema viteae | 2 | 0.01% |
| Atlantic bottle-nosed dolphin | 29 | 0.18% | Atlantic cod | 2 | 0.01% |
| American monarch | 28 | 0.17% | Clytia hemisphaerica | 2 | 0.01% |
| Angiostrongylus cantonensis | 27 | 0.17% | Trichosurus vulpecula | 2 | 0.01% |
| Pediculus humanus corporis | 27 | 0.17% | Dumeril's clam worm | 2 | 0.01% |
| Nile tilapia | 27 | 0.17% | Squalus acanthias | 2 | 0.01% |
| Anolis carolinensis | 27 | 0.17% | Dugesia japonica | 2 | 0.01% |
| Culex pipiens quinquefasciatus | 26 | 0.16% | Leptopilina boulardi | 2 | 0.01% |
| Drosophila mohavensis | 26 | 0.16% | Carassius auratus | 2 | 0.01% |
| Japanese medaka | 25 | 0.15% | Epinephelus coioides | 2 | 0.01% |
| Mus musculus | 24 | 0.15% | Astyanax mexicanus | 1 | 0.01% |
| Camponotus floridana | 23 | 0.14% | Syngamus trachea | 1 | 0.01% |
| Chelonia agassizi | 23 | 0.14% | Haliotis discus discus | 1 | 0.01% |
| Ascidia intestinalis | 23 | 0.14% | Oscheius brevesophaga | 1 | 0.01% |
| Silurana tropicalis | 22 | 0.14% | Diplodus sargus | 1 | 0.01% |
| Drosophila grimshawi | 22 | 0.14% | Crassostrea ariakensis | 1 | 0.01% |
| Trichoplax adhaerens | 22 | 0.14% | Diploscapter coronatus | 1 | 0.01% |
| Drosophila pseudoobscura pseudoobscura | 21 | 0.13% | Latrodectus hesperus | 1 | 0.01% |
| Harpegnathos saltator | 21 | 0.13% | Galleria mellonella | 1 | 0.01% |
| Hylobates concolor leucogenys | 21 | 0.13% | Colchester native oyster | 1 | 0.01% |
| Aedes aegypti | 21 | 0.13% | Pristionchus sp. 10 RS5133 | 1 | 0.01% |
| Monosiga brevicollis MX1 | 21 | 0.13% | Mytilus edulis | 1 | 0.01% |
| Dictyostelium discoideum AX4 | 21 | 0.13% | Aplysia californica | 1 | 0.01% |
| Oikopleura dioica | 20 | 0.12% | Myocastor coypus | 1 | 0.01% |
| Ailuropoda melanoleuca | 19 | 0.12% | Entamoeba nuttalli / P19 | 1 | 0.01% |
| Chinese tree shrew | 18 | 0.11% | Australian lungfish | 1 | 0.01% |
| Clonorchis sinensis | 18 | 0.11% | African green monkey | 1 | 0.01% |
| Sarcophilus harrisii | 18 | 0.11% | Pinctada margaritifera | 1 | 0.01% |
| Caenorhabditis angaria | 17 | 0.10% | Spadella cephaloptera | 1 | 0.01% |
| Bos grunniens mutus | 16 | 0.10% | Belcher's lancelet | 1 | 0.01% |
| Killer whale | 16 | 0.10% | Setaria digitata | 1 | 0.01% |
| Drosophila ananassae | 15 | 0.09% | Chironomus thummi | 1 | 0.01% |
| Acromyrmex echinatior | 15 | 0.09% | Schistosoma haematobium | 1 | 0.01% |
| Tetrahymena thermophila | 15 | 0.09% | Ceratitis capitata | 1 | 0.01% |
| Cavia aperea porcellus | 15 | 0.09% | Globodera rostochiensis | 1 | 0.01% |
| Leishmania major MHOM/IL/81/Friedlin | 15 | 0.09% | Pristionchus sp. 6 RS5101 | 1 | 0.01% |
| Fugu rubripes | 14 | 0.09% | Pinctada maxima | 1 | 0.01% |
| Tetraodon nigroviridis | 14 | 0.09% | Theileria annulata Ankara clone C9 | 1 | 0.01% |
| Ditylenchus destructor | 14 | 0.09% | Neodiplogaster sp. WEM-2009 | 1 | 0.01% |
| Bolivian squirrel monkey | 13 | 0.08% | Amblyomma americanum | 1 | 0.01% |
| Dictyostelium purpureum | 12 | 0.07% | Trypanosoma cruzi marinkellei | 1 | 0.01% |
| Polysphondylium pallidum PN500 | 12 | 0.07% | Helobdella sp. IQ-2007 | 1 | 0.01% |
| Drosophila virilis | 12 | 0.07% | Ornithodoros parkeri | 1 | 0.01% |
| Toxoplasma gondii ME49 | 12 | 0.07% | Mecistocirrus digitatus | 1 | 0.01% |
| Onchocerca volvulus | 12 | 0.07% | Pristionchus uniformis | 1 | 0.01% |
| Apis mellifera | 11 | 0.07% | Polysphondylium violaceum | 1 | 0.01% |
| Schistosoma mansoni | 11 | 0.07% | Branchiostoma lanceolatum | 1 | 0.01% |
| Drosophila willistoni | 11 | 0.07% | Esox lucius | 1 | 0.01% |
| Orang-utan | 11 | 0.07% | Locusta gregaria | 1 | 0.01% |
| Apis florea | 11 | 0.07% | Chautsi bass | 1 | 0.01% |
| Ornithorhynchus anatinus | 11 | 0.07% | Caligus rogercresseyi | 1 | 0.01% |
| CHO cell lines | 11 | 0.07% | Onchocerca ochengi | 1 | 0.01% |
| Dictyostelium discoideum | 10 | 0.06% | Golden hamsters | 1 | 0.01% |
| Dendroctonus ponderosae | 10 | 0.06% | Triatoma infestans | 1 | 0.01% |
| Dictyocaulus viviparus | 10 | 0.06% | Monosiga ovata | 1 | 0.01% |
| Bombus impatiens | 10 | 0.06% | Urechis unicinctus | 1 | 0.01% |
| Bursaphelenchus mucronatus | 10 | 0.06% | Ciona savignyi | 1 | 0.01% |
| Drosophila melanogaster | 10 | 0.06% | North Pacific bluefin tuna | 1 | 0.01% |
| Ascaris lumbricoides | 10 | 0.06% | Theileria parva strain Muguga | 1 | 0.01% |
| Ostertagia circumcincta | 9 | 0.06% | Toxoplasma gondii VEG | 1 | 0.01% |
| Necator americanus | 9 | 0.06% | Plasmodium chabaudi chabaudi | 1 | 0.01% |
| Callithrix jacchus | 9 | 0.06% | Cyprinus carpio | 1 | 0.01% |
| Perkinsus marinus ATCC 50983 | 9 | 0.06% | American cockroach | 1 | 0.01% |
| Macaca mulatta | 9 | 0.06% | Clarias batrachus | 1 | 0.01% |
| Buffalo rat | 9 | 0.06% | Aphelenchus avenae | 1 | 0.01% |
| Drosophila yakuba | 9 | 0.06% | Meloidogyne arenaria | 1 | 0.01% |
| Monodelphis domestica | 9 | 0.06% | Trichostrongylus colubriformis | 1 | 0.01% |
| Drosophila persimilis | 8 | 0.05% | Heterodera schachtii | 1 | 0.01% |
| Toxocara canis | 8 | 0.05% | Hemicentrotus pulcherrimus | 1 | 0.01% |
| Leishmania mexicana MHOM/GT/2001/U1103 | 8 | 0.05% | Cryptosporidium parvum Iowa II | 1 | 0.01% |
| Paramecium tetraurelia d4-2 | 8 | 0.05% | Hydractinia carnea | 1 | 0.01% |
| Entamoeba histolytica HM-1:IMSS | 8 | 0.05% | Giardia intestinalis ATCC 50581 | 1 | 0.01% |
| Sus scrofa | 8 | 0.05% | Episyrphus (Episyrphus) balteatus | 1 | 0.01% |
| Schistosoma japonicum | 7 | 0.04% | Onchocerca dukei | 1 | 0.01% |
| African bush elephant | 7 | 0.04% | Babesia bovis T2Bo | 1 | 0.01% |
| Lepeophtheirus salmonis | 7 | 0.04% | Glossina palpalis | 1 | 0.01% |
| Bombus terrestris | 7 | 0.04% | Babesia microti strain RI | 1 | 0.01% |
| Galago garnetti | 7 | 0.04% | Cryptosporidium hominis TU502 | 1 | 0.01% |
| Solenopsis invicta | 7 | 0.04% | Ixodes ricinus | 1 | 0.01% |
| Gallus domesticus | 7 | 0.04% | African dwarf goat | 1 | 0.01% |
| Acanthamoeba castellanii Neff strain | 7 | 0.04% | Trypanosoma brucei gambiense DAL972 | 1 | 0.01% |
| Canis canis | 7 | 0.04% | Globodera pallida | 1 | 0.01% |
| American malaria mosquito | 7 | 0.04% | Papilio polytes | 1 | 0.01% |
| Oxytricha trifallax | 7 | 0.04% | Caenorhabditis sp. 23 AD-2012 | 1 | 0.01% |
| European rabbit | 7 | 0.04% | Plasmodium yoelii | 1 | 0.01% |
| Pan troglodytes | 7 | 0.04% | Papilio xuthus | 1 | 0.01% |
| Dictyostelium fasciculatum | 6 | 0.04% | Artemia franciscana | 1 | 0.01% |
| Ovis ammon aries | 6 | 0.04% | Chironomus yoshimatsui | 1 | 0.01% |
| Toxoplasma gondii GT1 | 6 | 0.04% | Drosophila buzzati | 1 | 0.01% |
| Pristionchus pacificus | 6 | 0.04% | Streblomastix strix | 1 | 0.01% |
| Entamoeba invadens IP1 | 6 | 0.04% | Thermobia domestica | 1 | 0.01% |
| Dirofilaria immitis | 6 | 0.04% | Ctenopharyngodon idella | 1 | 0.01% |
| Phragmatopoma californica | 6 | 0.04% | Halocynthia roretzi | 1 | 0.01% |
| Atlantic salmon | 6 | 0.04% | Aphonopelma chalcodes | 1 | 0.01% |
| Radopholus similis | 6 | 0.04% | Paracentrotus lividus | 1 | 0.01% |
| Pteropus alecto | 6 | 0.04% | Mytilus chilensis | 1 | 0.01% |
| Myotis davidii | 6 | 0.04% | Osphronemus goramy | 1 | 0.01% |
| Columba livia | 6 | 0.04% | Gryllus bimaculatus | 1 | 0.01% |
| Ips paraconfusus | 6 | 0.04% | Callinectes sapidus | 1 | 0.01% |
| Ancylostoma caninum | 6 | 0.04% | Cyathostominae sp. JM-2007a | 1 | 0.01% |
| Steinernema feltiae | 5 | 0.03% | Mamestra configurata | 1 | 0.01% |
| Felis catus | 5 | 0.03% | Latrunculia (Biannulata) oparinae | 1 | 0.01% |
| Babesia equi | 5 | 0.03% | Dreissena polymorpha | 1 | 0.01% |
| Adineta vaga | 5 | 0.03% | Argas monolakensis | 1 | 0.01% |
| Equus caballus | 5 | 0.03% | Marsupenaeus japonicus | 1 | 0.01% |
| Cryptosporidium muris RN66 | 5 | 0.03% | Cylicocyclus nassatus | 1 | 0.01% |
| Bos bovis | 5 | 0.03% | Aelurostrongylus abstrusus | 1 | 0.01% |
| Leishmania infantum JPCM5 | 5 | 0.03% | Phalansterium solitarium | 1 | 0.01% |
| Drosophila erecta | 5 | 0.03% | Caenorhabditis japonica | 1 | 0.01% |
| Strongyloides stercoralis | 5 | 0.03% | Blattella germanica | 1 | 0.01% |
| Drosophila sechellia | 5 | 0.03% | Psammechinus miliaris | 1 | 0.01% |
| Anisakis simplex | 5 | 0.03% | Molgula tectiformis | 1 | 0.01% |
| Parascaris equorum | 4 | 0.02% | Baylisascaris schroederi | 1 | 0.01% |
| Naegleria gruberi strain NEG-M | 4 | 0.02% | Chrysomela tremula | 1 | 0.01% |
| Plasmodium falciparum (isolate 3D7) | 4 | 0.02% | Euplotes crassus | 1 | 0.01% |
| Meleagris gallopavo | 4 | 0.02% | Fasciola hepatica | 1 | 0.01% |
| Entamoeba dispar SAW760 | 4 | 0.02% | Parnell's mustached bat | 1 | 0.01% |
| Pan paniscus | 4 | 0.02% | Mediterranean mussel | 1 | 0.01% |
| Heterorhabditis bacteriophora | 4 | 0.02% | Derocheilocaris typicus | 1 | 0.01% |
| Ancylostoma ceylanicum | 4 | 0.02% | Cyathostomum tetracanthum | 1 | 0.01% |
| Lysiphlebus testaceipes | 4 | 0.02% | Asian seabass | 1 | 0.01% |
| Trypanosoma cruzi strain CL Brener | 4 | 0.02% | Trichinella pseudospiralis | 1 | 0.01% |
| Capsaspora owczarzaki ATCC 30864 | 4 | 0.02% | Meloidogyne artiellia | 1 | 0.01% |
| Poephila guttata | 4 | 0.02% | Meloidogyne javanica | 1 | 0.01% |
| Aphelenchoides fragariae | 4 | 0.02% | Atlantic horseshoe crab | 1 | 0.01% |
| Pinctada fucata | 4 | 0.02% |  |  |  |

**Table E. Carbohydrate-active enzymes identified in the transcriptome analysis of *Aphelenchoides ritzemabosi*.**

| CAZy enzyme classes Number of related families Unigenes number | | |
| --- | --- | --- |
| Glycoside hydrolases (GHs) | 46 | 266 |
| Glycosyltransferases (GTs) | 48 | 449 |
| Carbohydrate esterases (CEs) | 6 | 95 |
| Carbohydrate-binding modules (CBMs) | 22 | 513 |

**Table F. The transcripts involved in the RNAi pathway, annotated in the transcriptomic analysis of *A. ritzemabosi*.**

| geneID | Subject | Identities | Evalue | Score |
| --- | --- | --- | --- | --- |
| CL1448.Contig1_YK | drh-1 | 28.698 | 1.57E-22 | 94.7 |
| CL1448.Contig2_YK | drh-1 | 28.994 | 5.10E-23 | 96.3 |
| CL1693.Contig1_YK | drh-1 | 29.221 | 1.72E-06 | 43.9 |
| CL1693.Contig2_YK | drh-1 | 29.221 | 1.81E-06 | 43.9 |
| CL1693.Contig3_YK | drh-1 | 30.065 | 3.52E-08 | 49.7 |
| CL1693.Contig4_YK | drh-1 | 30.065 | 2.53E-08 | 49.7 |
| CL1693.Contig5_YK | drh-1 | 30.065 | 3.25E-08 | 49.3 |
| CL815.Contig1_YK | drh-1 | 34.831 | 6.17E-48 | 175 |
| CL815.Contig2_YK | drh-1 | 32.615 | 1.98E-77 | 156 |
| CL815.Contig3_YK | drh-1 | 29.191 | 2.34E-89 | 306 |
| CL815.Contig4_YK | drh-1 | 37.868 | 1.43E-41 | 155 |
| CL815.Contig5_YK | drh-1 | 31.209 | 2.27E-83 | 176 |
| CL815.Contig6_YK | drh-1 | 29.396 | 1.26E-82 | 286 |
| Unigene11972_YK | drh-1 | 29.851 | 2.12E-07 | 46.6 |
| Unigene14339_YK | drh-1 | 28.504 | 3.73E-90 | 308 |
| Unigene14472_YK | drh-1 | 27.66 | 1.43E-06 | 44.3 |
| Unigene5558_YK | drh-1 | 30.855 | 2.49E-27 | 109 |
| Unigene6436_YK | drh-1 | 27.473 | 1.29E-06 | 45.8 |
|  |  |  |  |  |
| CL2691.Contig1_YK | mut-7 | 22.954 | 1.84E-19 | 86.7 |
| Unigene14412_YK | mut-7 | 21.832 | 7.67E-19 | 84.7 |
| Unigene3013_YK | mut-7 | 42.623 | 4.23E-09 | 52.8 |
|  |  |  |  |  |
| CL2098.Contig1_YK | rde-4 | 27.485 | 5.37E-09 | 50.1 |
| CL2098.Contig2_YK | rde-4 | 27.485 | 5.25E-09 | 50.1 |
| Unigene6646_YK | rde-4 | 21.457 | 2.87E-07 | 44.3 |
|  |  |  |  |  |
| CL1134.Contig1_YK | smg-2 | 55.215 | 0 | 1016 |
| CL1134.Contig2_YK | smg-2 | 39.785 | 3.75E-32 | 119 |
| CL2554.Contig1_YK | smg-2 | 26.452 | 9.70E-11 | 53.9 |
| CL2554.Contig2_YK | smg-2 | 26.452 | 1.34E-10 | 53.9 |
| CL524.Contig1_YK | smg-2 | 28.778 | 1.11E-32 | 130 |
| Unigene11545_YK | smg-2 | 28.696 | 1.57E-10 | 55.1 |
| Unigene14366_YK | smg-2 | 27.458 | 6.45E-25 | 105 |
| Unigene14535_YK | smg-2 | 29.343 | 5.99E-36 | 139 |
| Unigene14620_YK | smg-2 | 27.912 | 7.45E-31 | 122 |
| Unigene3398_YK | smg-2 | 27.729 | 1.01E-33 | 132 |
| Unigene9258_YK | smg-2 | 54.59 | 0 | 690 |
| Unigene9259_YK | smg-2 | 61.538 | 8.98E-42 | 141 |
| CL1374.Contig4_YK | dcr-1 | 30.097 | 9.15E-06 | 41.2 |
| CL2699.Contig1_YK | dcr-1 | 40.385 | 7.75E-06 | 42.7 |
| CL2699.Contig2_YK | dcr-1 | 40.385 | 7.87E-06 | 42.7 |
| Unigene12498_YK | dcr-1 | 25.85 | 2.70E-07 | 47 |
| Unigene13141_YK | dcr-1 | 34.483 | 6.93E-07 | 44.7 |
| Unigene13193_YK | dcr-1 | 30.275 | 1.60E-07 | 46.6 |
| Unigene14066_YK | dcr-1 | 34.286 | 3.69E-07 | 45.8 |
| Unigene14603_YK | dcr-1 | 25 | 1.23E-06 | 43.9 |
| Unigene14846_YK | dcr-1 | 34.936 | 0 | 685 |
| Unigene4289_YK | dcr-1 | 32.075 | 2.71E-06 | 42.7 |
| Unigene6435_YK | dcr-1 | 34.483 | 6.66E-07 | 45.8 |
| Unigene14108_YK | ego-1 | 36.12 | 0 | 745 |
| Unigene6374_YK | ego-1 | 30 | 5.55E-60 | 213 |
| Unigene6375_YK | ego-1 | 36.863 | 7.54E-178 | 561 |
| Unigene2477_YK | rrf-3 | 38.105 | 0 | 815 |
| CL1706.Contig2_YK | ppw-2 | 24.859 | 3.10E-18 | 80.1 |
| CL950.Contig1_YK | ppw-2 | 26.415 | 2.06E-14 | 68.2 |
| CL950.Contig2_YK | ppw-2 | 28.972 | 4.80E-12 | 58.9 |
| Unigene12345_YK | ppw-2 | 27.441 | 2.79E-34 | 128 |
| Unigene12681_YK | ppw-2 | 25.477 | 3.40E-37 | 140 |
| CL948.Contig1_YK | sago-1 | 18.806 | 5.63E-10 | 55.5 |
| CL1859.Contig1_YK | gfl-1 | 45.109 | 9.03E-52 | 164 |
| CL1859.Contig2_YK | gfl-1 | 46.734 | 1.97E-57 | 179 |
| Unigene3385_YK | gfl-1 | 40 | 4.84E-14 | 63.2 |
| Unigene13774_YK | rsd-3 | 35.446 | 2.48E-57 | 196 |
| Unigene4330_YK | rsd-3 | 40.299 | 6.56E-30 | 116 |
| Unigene4423_YK | rsd-3 | 30 | 1.02E-09 | 52.8 |
| Unigene2880_YK | rsd-6 | 26 | 1.07E-06 | 43.5 |
| Unigene13298_YK | alg-1 | 79.367 | 0 | 1510 |
| Unigene11256_YK | drsh-1 | 37.315 | 0 | 558 |
| Unigene13566_YK | tsn-1 | 50.498 | 0 | 935 |
| Unigene14207_YK | tsn-1 | 32.813 | 8.92E-07 | 42.4 |
| CL1633.Contig1_YK | vig-1 | 36.25 | 2.01E-12 | 61.6 |
