# Supplementary material for: Transcriptome Analysis of the Chrysanthemum Foliar Nematode, Aphelenchoides ritzemabosi (Aphelenchida: Aphelenchoididae)
Source: PLoS One. 2016 Nov 22;11(11):e0166877. doi: 10.1371/journal.pone.0166877 (PMC5119785; doi:10.1371/journal.pone.0166877)
Supplement: S6 File — (DOC) [file pone.0166877.s006.doc]

**1. YK-Unigene.fa**

| **#** | **Pathway** | **All genes with pathway annotation (11645)** | **Pathway ID** | **Level 1** | **Level 2** |
| --- | --- | --- | --- | --- | --- |
| 1 | [Metabolic pathways](../../../../D:%5C%E9%AB%98%E9%80%9A%E9%87%8F%E6%B5%8B%E5%BA%8F%E7%BB%93%E6%9E%9C%5CF14FTSSCKF1242_NEMpnqE%5Cannotation%5CKEGG%5CYK-Unigene.fa.htm#gene1) | 1702 (14.62%) | ko01100 | Metabolism | Global map |
| 2 | [Lysosome](../../../../D:%5C%E9%AB%98%E9%80%9A%E9%87%8F%E6%B5%8B%E5%BA%8F%E7%BB%93%E6%9E%9C%5CF14FTSSCKF1242_NEMpnqE%5Cannotation%5CKEGG%5CYK-Unigene.fa.htm#gene2) | 477 (4.1%) | ko04142 | Cellular Processes | Transport and catabolism |
| 3 | [Focal adhesion](../../../../D:%5C%E9%AB%98%E9%80%9A%E9%87%8F%E6%B5%8B%E5%BA%8F%E7%BB%93%E6%9E%9C%5CF14FTSSCKF1242_NEMpnqE%5Cannotation%5CKEGG%5CYK-Unigene.fa.htm#gene3) | 472 (4.05%) | ko04510 | Cellular Processes | Cell communication |
| 4 | [Pathways in cancer](../../../../D:%5C%E9%AB%98%E9%80%9A%E9%87%8F%E6%B5%8B%E5%BA%8F%E7%BB%93%E6%9E%9C%5CF14FTSSCKF1242_NEMpnqE%5Cannotation%5CKEGG%5CYK-Unigene.fa.htm#gene4) | 468 (4.02%) | ko05200 | Human Diseases | Cancers: Overview |
| 5 | [Regulation of actin cytoskeleton](../../../../D:%5C%E9%AB%98%E9%80%9A%E9%87%8F%E6%B5%8B%E5%BA%8F%E7%BB%93%E6%9E%9C%5CF14FTSSCKF1242_NEMpnqE%5Cannotation%5CKEGG%5CYK-Unigene.fa.htm#gene5) | 456 (3.92%) | ko04810 | Cellular Processes | Cell motility |
| 6 | [Vascular smooth muscle contraction](../../../../D:%5C%E9%AB%98%E9%80%9A%E9%87%8F%E6%B5%8B%E5%BA%8F%E7%BB%93%E6%9E%9C%5CF14FTSSCKF1242_NEMpnqE%5Cannotation%5CKEGG%5CYK-Unigene.fa.htm#gene6) | 434 (3.73%) | ko04270 | Organismal Systems | Circulatory system |
| 7 | [Calcium signaling pathway](../../../../D:%5C%E9%AB%98%E9%80%9A%E9%87%8F%E6%B5%8B%E5%BA%8F%E7%BB%93%E6%9E%9C%5CF14FTSSCKF1242_NEMpnqE%5Cannotation%5CKEGG%5CYK-Unigene.fa.htm#gene7) | 345 (2.96%) | ko04020 | Environmental Information Processing | Signal transduction |
| 8 | [Protein digestion and absorption](../../../../D:%5C%E9%AB%98%E9%80%9A%E9%87%8F%E6%B5%8B%E5%BA%8F%E7%BB%93%E6%9E%9C%5CF14FTSSCKF1242_NEMpnqE%5Cannotation%5CKEGG%5CYK-Unigene.fa.htm#gene8) | 338 (2.9%) | ko04974 | Organismal Systems | Digestive system |
| 9 | [Amoebiasis](../../../../D:%5C%E9%AB%98%E9%80%9A%E9%87%8F%E6%B5%8B%E5%BA%8F%E7%BB%93%E6%9E%9C%5CF14FTSSCKF1242_NEMpnqE%5Cannotation%5CKEGG%5CYK-Unigene.fa.htm#gene9) | 334 (2.87%) | ko05146 | Human Diseases | Infectious diseases: Parasitic |
| 10 | [Spliceosome](../../../../D:%5C%E9%AB%98%E9%80%9A%E9%87%8F%E6%B5%8B%E5%BA%8F%E7%BB%93%E6%9E%9C%5CF14FTSSCKF1242_NEMpnqE%5Cannotation%5CKEGG%5CYK-Unigene.fa.htm#gene10) | 299 (2.57%) | ko03040 | Genetic Information Processing | Transcription |
| 11 | [Alzheimer's disease](../../../../D:%5C%E9%AB%98%E9%80%9A%E9%87%8F%E6%B5%8B%E5%BA%8F%E7%BB%93%E6%9E%9C%5CF14FTSSCKF1242_NEMpnqE%5Cannotation%5CKEGG%5CYK-Unigene.fa.htm#gene11) | 299 (2.57%) | ko05010 | Human Diseases | Neurodegenerative diseases |
| 12 | [MAPK signaling pathway](../../../../D:%5C%E9%AB%98%E9%80%9A%E9%87%8F%E6%B5%8B%E5%BA%8F%E7%BB%93%E6%9E%9C%5CF14FTSSCKF1242_NEMpnqE%5Cannotation%5CKEGG%5CYK-Unigene.fa.htm#gene12) | 293 (2.52%) | ko04010 | Environmental Information Processing | Signal transduction |
| 13 | [HTLV-I infection](../../../../D:%5C%E9%AB%98%E9%80%9A%E9%87%8F%E6%B5%8B%E5%BA%8F%E7%BB%93%E6%9E%9C%5CF14FTSSCKF1242_NEMpnqE%5Cannotation%5CKEGG%5CYK-Unigene.fa.htm#gene13) | 291 (2.5%) | ko05166 | Human Diseases | Infectious diseases: Viral |
| 14 | [Tight junction](../../../../D:%5C%E9%AB%98%E9%80%9A%E9%87%8F%E6%B5%8B%E5%BA%8F%E7%BB%93%E6%9E%9C%5CF14FTSSCKF1242_NEMpnqE%5Cannotation%5CKEGG%5CYK-Unigene.fa.htm#gene14) | 288 (2.47%) | ko04530 | Cellular Processes | Cell communication |
| 15 | [Neuroactive ligand-receptor interaction](../../../../D:%5C%E9%AB%98%E9%80%9A%E9%87%8F%E6%B5%8B%E5%BA%8F%E7%BB%93%E6%9E%9C%5CF14FTSSCKF1242_NEMpnqE%5Cannotation%5CKEGG%5CYK-Unigene.fa.htm#gene15) | 279 (2.4%) | ko04080 | Environmental Information Processing | Signaling molecules and interaction |
| 16 | [Dilated cardiomyopathy](../../../../D:%5C%E9%AB%98%E9%80%9A%E9%87%8F%E6%B5%8B%E5%BA%8F%E7%BB%93%E6%9E%9C%5CF14FTSSCKF1242_NEMpnqE%5Cannotation%5CKEGG%5CYK-Unigene.fa.htm#gene16) | 277 (2.38%) | ko05414 | Human Diseases | Cardiovascular diseases |
| 17 | [Purine metabolism](../../../../D:%5C%E9%AB%98%E9%80%9A%E9%87%8F%E6%B5%8B%E5%BA%8F%E7%BB%93%E6%9E%9C%5CF14FTSSCKF1242_NEMpnqE%5Cannotation%5CKEGG%5CYK-Unigene.fa.htm#gene17) | 276 (2.37%) | ko00230 | Metabolism | Nucleotide metabolism |
| 18 | [Tuberculosis](../../../../D:%5C%E9%AB%98%E9%80%9A%E9%87%8F%E6%B5%8B%E5%BA%8F%E7%BB%93%E6%9E%9C%5CF14FTSSCKF1242_NEMpnqE%5Cannotation%5CKEGG%5CYK-Unigene.fa.htm#gene18) | 276 (2.37%) | ko05152 | Human Diseases | Infectious diseases: Bacterial |
| 19 | [Huntington's disease](../../../../D:%5C%E9%AB%98%E9%80%9A%E9%87%8F%E6%B5%8B%E5%BA%8F%E7%BB%93%E6%9E%9C%5CF14FTSSCKF1242_NEMpnqE%5Cannotation%5CKEGG%5CYK-Unigene.fa.htm#gene19) | 273 (2.34%) | ko05016 | Human Diseases | Neurodegenerative diseases |
| 20 | [Bile secretion](../../../../D:%5C%E9%AB%98%E9%80%9A%E9%87%8F%E6%B5%8B%E5%BA%8F%E7%BB%93%E6%9E%9C%5CF14FTSSCKF1242_NEMpnqE%5Cannotation%5CKEGG%5CYK-Unigene.fa.htm#gene20) | 273 (2.34%) | ko04976 | Organismal Systems | Digestive system |
| 21 | [Epstein-Barr virus infection](../../../../D:%5C%E9%AB%98%E9%80%9A%E9%87%8F%E6%B5%8B%E5%BA%8F%E7%BB%93%E6%9E%9C%5CF14FTSSCKF1242_NEMpnqE%5Cannotation%5CKEGG%5CYK-Unigene.fa.htm#gene21) | 269 (2.31%) | ko05169 | Human Diseases | Infectious diseases: Viral |
| 22 | [Gastric acid secretion](../../../../D:%5C%E9%AB%98%E9%80%9A%E9%87%8F%E6%B5%8B%E5%BA%8F%E7%BB%93%E6%9E%9C%5CF14FTSSCKF1242_NEMpnqE%5Cannotation%5CKEGG%5CYK-Unigene.fa.htm#gene22) | 267 (2.29%) | ko04971 | Organismal Systems | Digestive system |
| 23 | [Hypertrophic cardiomyopathy (HCM)](../../../../D:%5C%E9%AB%98%E9%80%9A%E9%87%8F%E6%B5%8B%E5%BA%8F%E7%BB%93%E6%9E%9C%5CF14FTSSCKF1242_NEMpnqE%5Cannotation%5CKEGG%5CYK-Unigene.fa.htm#gene23) | 267 (2.29%) | ko05410 | Human Diseases | Cardiovascular diseases |
| 24 | [Insulin signaling pathway](../../../../D:%5C%E9%AB%98%E9%80%9A%E9%87%8F%E6%B5%8B%E5%BA%8F%E7%BB%93%E6%9E%9C%5CF14FTSSCKF1242_NEMpnqE%5Cannotation%5CKEGG%5CYK-Unigene.fa.htm#gene24) | 264 (2.27%) | ko04910 | Organismal Systems | Endocrine system |
| 25 | [Dopaminergic synapse](../../../../D:%5C%E9%AB%98%E9%80%9A%E9%87%8F%E6%B5%8B%E5%BA%8F%E7%BB%93%E6%9E%9C%5CF14FTSSCKF1242_NEMpnqE%5Cannotation%5CKEGG%5CYK-Unigene.fa.htm#gene25) | 263 (2.26%) | ko04728 | Organismal Systems | Nervous system |
| 26 | [RNA transport](../../../../D:%5C%E9%AB%98%E9%80%9A%E9%87%8F%E6%B5%8B%E5%BA%8F%E7%BB%93%E6%9E%9C%5CF14FTSSCKF1242_NEMpnqE%5Cannotation%5CKEGG%5CYK-Unigene.fa.htm#gene26) | 261 (2.24%) | ko03013 | Genetic Information Processing | Translation |
| 27 | [Protein processing in endoplasmic reticulum](../../../../D:%5C%E9%AB%98%E9%80%9A%E9%87%8F%E6%B5%8B%E5%BA%8F%E7%BB%93%E6%9E%9C%5CF14FTSSCKF1242_NEMpnqE%5Cannotation%5CKEGG%5CYK-Unigene.fa.htm#gene27) | 247 (2.12%) | ko04141 | Genetic Information Processing | Folding, sorting and degradation |
| 28 | [Wnt signaling pathway](../../../../D:%5C%E9%AB%98%E9%80%9A%E9%87%8F%E6%B5%8B%E5%BA%8F%E7%BB%93%E6%9E%9C%5CF14FTSSCKF1242_NEMpnqE%5Cannotation%5CKEGG%5CYK-Unigene.fa.htm#gene28) | 245 (2.1%) | ko04310 | Environmental Information Processing | Signal transduction |
| 29 | [Salivary secretion](../../../../D:%5C%E9%AB%98%E9%80%9A%E9%87%8F%E6%B5%8B%E5%BA%8F%E7%BB%93%E6%9E%9C%5CF14FTSSCKF1242_NEMpnqE%5Cannotation%5CKEGG%5CYK-Unigene.fa.htm#gene29) | 243 (2.09%) | ko04970 | Organismal Systems | Digestive system |
| 30 | [Transcriptional misregulation in cancer](../../../../D:%5C%E9%AB%98%E9%80%9A%E9%87%8F%E6%B5%8B%E5%BA%8F%E7%BB%93%E6%9E%9C%5CF14FTSSCKF1242_NEMpnqE%5Cannotation%5CKEGG%5CYK-Unigene.fa.htm#gene30) | 241 (2.07%) | ko05202 | Human Diseases | Cancers: Overview |
| 31 | [Endocytosis](../../../../D:%5C%E9%AB%98%E9%80%9A%E9%87%8F%E6%B5%8B%E5%BA%8F%E7%BB%93%E6%9E%9C%5CF14FTSSCKF1242_NEMpnqE%5Cannotation%5CKEGG%5CYK-Unigene.fa.htm#gene31) | 240 (2.06%) | ko04144 | Cellular Processes | Transport and catabolism |
| 32 | [Chemokine signaling pathway](../../../../D:%5C%E9%AB%98%E9%80%9A%E9%87%8F%E6%B5%8B%E5%BA%8F%E7%BB%93%E6%9E%9C%5CF14FTSSCKF1242_NEMpnqE%5Cannotation%5CKEGG%5CYK-Unigene.fa.htm#gene32) | 234 (2.01%) | ko04062 | Organismal Systems | Immune system |
| 33 | [Glutamatergic synapse](../../../../D:%5C%E9%AB%98%E9%80%9A%E9%87%8F%E6%B5%8B%E5%BA%8F%E7%BB%93%E6%9E%9C%5CF14FTSSCKF1242_NEMpnqE%5Cannotation%5CKEGG%5CYK-Unigene.fa.htm#gene33) | 232 (1.99%) | ko04724 | Organismal Systems | Nervous system |
| 34 | [Salmonella infection](../../../../D:%5C%E9%AB%98%E9%80%9A%E9%87%8F%E6%B5%8B%E5%BA%8F%E7%BB%93%E6%9E%9C%5CF14FTSSCKF1242_NEMpnqE%5Cannotation%5CKEGG%5CYK-Unigene.fa.htm#gene34) | 232 (1.99%) | ko05132 | Human Diseases | Infectious diseases: Bacterial |
| 35 | [Serotonergic synapse](../../../../D:%5C%E9%AB%98%E9%80%9A%E9%87%8F%E6%B5%8B%E5%BA%8F%E7%BB%93%E6%9E%9C%5CF14FTSSCKF1242_NEMpnqE%5Cannotation%5CKEGG%5CYK-Unigene.fa.htm#gene35) | 230 (1.98%) | ko04726 | Organismal Systems | Nervous system |
| 36 | [Vibrio cholerae infection](../../../../D:%5C%E9%AB%98%E9%80%9A%E9%87%8F%E6%B5%8B%E5%BA%8F%E7%BB%93%E6%9E%9C%5CF14FTSSCKF1242_NEMpnqE%5Cannotation%5CKEGG%5CYK-Unigene.fa.htm#gene36) | 227 (1.95%) | ko05110 | Human Diseases | Infectious diseases: Bacterial |
| 37 | [Pancreatic secretion](../../../../D:%5C%E9%AB%98%E9%80%9A%E9%87%8F%E6%B5%8B%E5%BA%8F%E7%BB%93%E6%9E%9C%5CF14FTSSCKF1242_NEMpnqE%5Cannotation%5CKEGG%5CYK-Unigene.fa.htm#gene37) | 223 (1.91%) | ko04972 | Organismal Systems | Digestive system |
| 38 | [Herpes simplex infection](../../../../D:%5C%E9%AB%98%E9%80%9A%E9%87%8F%E6%B5%8B%E5%BA%8F%E7%BB%93%E6%9E%9C%5CF14FTSSCKF1242_NEMpnqE%5Cannotation%5CKEGG%5CYK-Unigene.fa.htm#gene38) | 219 (1.88%) | ko05168 | Human Diseases | Infectious diseases: Viral |
| 39 | [ECM-receptor interaction](../../../../D:%5C%E9%AB%98%E9%80%9A%E9%87%8F%E6%B5%8B%E5%BA%8F%E7%BB%93%E6%9E%9C%5CF14FTSSCKF1242_NEMpnqE%5Cannotation%5CKEGG%5CYK-Unigene.fa.htm#gene39) | 217 (1.86%) | ko04512 | Environmental Information Processing | Signaling molecules and interaction |
| 40 | [Melanogenesis](../../../../D:%5C%E9%AB%98%E9%80%9A%E9%87%8F%E6%B5%8B%E5%BA%8F%E7%BB%93%E6%9E%9C%5CF14FTSSCKF1242_NEMpnqE%5Cannotation%5CKEGG%5CYK-Unigene.fa.htm#gene40) | 211 (1.81%) | ko04916 | Organismal Systems | Endocrine system |
| 41 | [Cardiac muscle contraction](../../../../D:%5C%E9%AB%98%E9%80%9A%E9%87%8F%E6%B5%8B%E5%BA%8F%E7%BB%93%E6%9E%9C%5CF14FTSSCKF1242_NEMpnqE%5Cannotation%5CKEGG%5CYK-Unigene.fa.htm#gene41) | 211 (1.81%) | ko04260 | Organismal Systems | Circulatory system |
| 42 | [Ubiquitin mediated proteolysis](../../../../D:%5C%E9%AB%98%E9%80%9A%E9%87%8F%E6%B5%8B%E5%BA%8F%E7%BB%93%E6%9E%9C%5CF14FTSSCKF1242_NEMpnqE%5Cannotation%5CKEGG%5CYK-Unigene.fa.htm#gene42) | 209 (1.79%) | ko04120 | Genetic Information Processing | Folding, sorting and degradation |
| 43 | [GnRH signaling pathway](../../../../D:%5C%E9%AB%98%E9%80%9A%E9%87%8F%E6%B5%8B%E5%BA%8F%E7%BB%93%E6%9E%9C%5CF14FTSSCKF1242_NEMpnqE%5Cannotation%5CKEGG%5CYK-Unigene.fa.htm#gene43) | 208 (1.79%) | ko04912 | Organismal Systems | Endocrine system |
| 44 | [Long-term potentiation](../../../../D:%5C%E9%AB%98%E9%80%9A%E9%87%8F%E6%B5%8B%E5%BA%8F%E7%BB%93%E6%9E%9C%5CF14FTSSCKF1242_NEMpnqE%5Cannotation%5CKEGG%5CYK-Unigene.fa.htm#gene44) | 208 (1.79%) | ko04720 | Organismal Systems | Nervous system |
| 45 | [Alcoholism](../../../../D:%5C%E9%AB%98%E9%80%9A%E9%87%8F%E6%B5%8B%E5%BA%8F%E7%BB%93%E6%9E%9C%5CF14FTSSCKF1242_NEMpnqE%5Cannotation%5CKEGG%5CYK-Unigene.fa.htm#gene45) | 205 (1.76%) | ko05034 | Human Diseases | Substance dependence |
| 46 | [Cholinergic synapse](../../../../D:%5C%E9%AB%98%E9%80%9A%E9%87%8F%E6%B5%8B%E5%BA%8F%E7%BB%93%E6%9E%9C%5CF14FTSSCKF1242_NEMpnqE%5Cannotation%5CKEGG%5CYK-Unigene.fa.htm#gene46) | 204 (1.75%) | ko04725 | Organismal Systems | Nervous system |
| 47 | [Oocyte meiosis](../../../../D:%5C%E9%AB%98%E9%80%9A%E9%87%8F%E6%B5%8B%E5%BA%8F%E7%BB%93%E6%9E%9C%5CF14FTSSCKF1242_NEMpnqE%5Cannotation%5CKEGG%5CYK-Unigene.fa.htm#gene47) | 204 (1.75%) | ko04114 | Cellular Processes | Cell growth and death |
| 48 | [Adherens junction](../../../../D:%5C%E9%AB%98%E9%80%9A%E9%87%8F%E6%B5%8B%E5%BA%8F%E7%BB%93%E6%9E%9C%5CF14FTSSCKF1242_NEMpnqE%5Cannotation%5CKEGG%5CYK-Unigene.fa.htm#gene48) | 203 (1.74%) | ko04520 | Cellular Processes | Cell communication |
| 49 | [Cell cycle](../../../../D:%5C%E9%AB%98%E9%80%9A%E9%87%8F%E6%B5%8B%E5%BA%8F%E7%BB%93%E6%9E%9C%5CF14FTSSCKF1242_NEMpnqE%5Cannotation%5CKEGG%5CYK-Unigene.fa.htm#gene49) | 200 (1.72%) | ko04110 | Cellular Processes | Cell growth and death |
| 50 | [Retrograde endocannabinoid signaling](../../../../D:%5C%E9%AB%98%E9%80%9A%E9%87%8F%E6%B5%8B%E5%BA%8F%E7%BB%93%E6%9E%9C%5CF14FTSSCKF1242_NEMpnqE%5Cannotation%5CKEGG%5CYK-Unigene.fa.htm#gene50) | 196 (1.68%) | ko04723 | Organismal Systems | Nervous system |
| 51 | [mRNA surveillance pathway](../../../../D:%5C%E9%AB%98%E9%80%9A%E9%87%8F%E6%B5%8B%E5%BA%8F%E7%BB%93%E6%9E%9C%5CF14FTSSCKF1242_NEMpnqE%5Cannotation%5CKEGG%5CYK-Unigene.fa.htm#gene51) | 195 (1.67%) | ko03015 | Genetic Information Processing | Translation |
| 52 | [Phagosome](../../../../D:%5C%E9%AB%98%E9%80%9A%E9%87%8F%E6%B5%8B%E5%BA%8F%E7%BB%93%E6%9E%9C%5CF14FTSSCKF1242_NEMpnqE%5Cannotation%5CKEGG%5CYK-Unigene.fa.htm#gene52) | 194 (1.67%) | ko04145 | Cellular Processes | Transport and catabolism |
| 53 | [Gap junction](../../../../D:%5C%E9%AB%98%E9%80%9A%E9%87%8F%E6%B5%8B%E5%BA%8F%E7%BB%93%E6%9E%9C%5CF14FTSSCKF1242_NEMpnqE%5Cannotation%5CKEGG%5CYK-Unigene.fa.htm#gene53) | 192 (1.65%) | ko04540 | Cellular Processes | Cell communication |
| 54 | [Ribosome](../../../../D:%5C%E9%AB%98%E9%80%9A%E9%87%8F%E6%B5%8B%E5%BA%8F%E7%BB%93%E6%9E%9C%5CF14FTSSCKF1242_NEMpnqE%5Cannotation%5CKEGG%5CYK-Unigene.fa.htm#gene54) | 191 (1.64%) | ko03010 | Genetic Information Processing | Translation |
| 55 | [Phosphatidylinositol signaling system](../../../../D:%5C%E9%AB%98%E9%80%9A%E9%87%8F%E6%B5%8B%E5%BA%8F%E7%BB%93%E6%9E%9C%5CF14FTSSCKF1242_NEMpnqE%5Cannotation%5CKEGG%5CYK-Unigene.fa.htm#gene55) | 190 (1.63%) | ko04070 | Environmental Information Processing | Signal transduction |
| 56 | [Viral myocarditis](../../../../D:%5C%E9%AB%98%E9%80%9A%E9%87%8F%E6%B5%8B%E5%BA%8F%E7%BB%93%E6%9E%9C%5CF14FTSSCKF1242_NEMpnqE%5Cannotation%5CKEGG%5CYK-Unigene.fa.htm#gene56) | 188 (1.61%) | ko05416 | Human Diseases | Cardiovascular diseases |
| 57 | [Progesterone-mediated oocyte maturation](../../../../D:%5C%E9%AB%98%E9%80%9A%E9%87%8F%E6%B5%8B%E5%BA%8F%E7%BB%93%E6%9E%9C%5CF14FTSSCKF1242_NEMpnqE%5Cannotation%5CKEGG%5CYK-Unigene.fa.htm#gene57) | 181 (1.55%) | ko04914 | Organismal Systems | Endocrine system |
| 58 | [Influenza A](../../../../D:%5C%E9%AB%98%E9%80%9A%E9%87%8F%E6%B5%8B%E5%BA%8F%E7%BB%93%E6%9E%9C%5CF14FTSSCKF1242_NEMpnqE%5Cannotation%5CKEGG%5CYK-Unigene.fa.htm#gene58) | 178 (1.53%) | ko05164 | Human Diseases | Infectious diseases: Viral |
| 59 | [Amphetamine addiction](../../../../D:%5C%E9%AB%98%E9%80%9A%E9%87%8F%E6%B5%8B%E5%BA%8F%E7%BB%93%E6%9E%9C%5CF14FTSSCKF1242_NEMpnqE%5Cannotation%5CKEGG%5CYK-Unigene.fa.htm#gene59) | 174 (1.49%) | ko05031 | Human Diseases | Substance dependence |
| 60 | [GABAergic synapse](../../../../D:%5C%E9%AB%98%E9%80%9A%E9%87%8F%E6%B5%8B%E5%BA%8F%E7%BB%93%E6%9E%9C%5CF14FTSSCKF1242_NEMpnqE%5Cannotation%5CKEGG%5CYK-Unigene.fa.htm#gene60) | 168 (1.44%) | ko04727 | Organismal Systems | Nervous system |
| 61 | [Axon guidance](../../../../D:%5C%E9%AB%98%E9%80%9A%E9%87%8F%E6%B5%8B%E5%BA%8F%E7%BB%93%E6%9E%9C%5CF14FTSSCKF1242_NEMpnqE%5Cannotation%5CKEGG%5CYK-Unigene.fa.htm#gene61) | 165 (1.42%) | ko04360 | Organismal Systems | Development |
| 62 | [Neurotrophin signaling pathway](../../../../D:%5C%E9%AB%98%E9%80%9A%E9%87%8F%E6%B5%8B%E5%BA%8F%E7%BB%93%E6%9E%9C%5CF14FTSSCKF1242_NEMpnqE%5Cannotation%5CKEGG%5CYK-Unigene.fa.htm#gene62) | 163 (1.4%) | ko04722 | Organismal Systems | Nervous system |
| 63 | [Oxidative phosphorylation](../../../../D:%5C%E9%AB%98%E9%80%9A%E9%87%8F%E6%B5%8B%E5%BA%8F%E7%BB%93%E6%9E%9C%5CF14FTSSCKF1242_NEMpnqE%5Cannotation%5CKEGG%5CYK-Unigene.fa.htm#gene63) | 162 (1.39%) | ko00190 | Metabolism | Energy metabolism |
| 64 | [Pyrimidine metabolism](../../../../D:%5C%E9%AB%98%E9%80%9A%E9%87%8F%E6%B5%8B%E5%BA%8F%E7%BB%93%E6%9E%9C%5CF14FTSSCKF1242_NEMpnqE%5Cannotation%5CKEGG%5CYK-Unigene.fa.htm#gene64) | 160 (1.37%) | ko00240 | Metabolism | Nucleotide metabolism |
| 65 | [Fc gamma R-mediated phagocytosis](../../../../D:%5C%E9%AB%98%E9%80%9A%E9%87%8F%E6%B5%8B%E5%BA%8F%E7%BB%93%E6%9E%9C%5CF14FTSSCKF1242_NEMpnqE%5Cannotation%5CKEGG%5CYK-Unigene.fa.htm#gene65) | 157 (1.35%) | ko04666 | Organismal Systems | Immune system |
| 66 | [Endocrine and other factor-regulated calcium reabsorption](../../../../D:%5C%E9%AB%98%E9%80%9A%E9%87%8F%E6%B5%8B%E5%BA%8F%E7%BB%93%E6%9E%9C%5CF14FTSSCKF1242_NEMpnqE%5Cannotation%5CKEGG%5CYK-Unigene.fa.htm#gene66) | 156 (1.34%) | ko04961 | Organismal Systems | Excretory system |
| 67 | [Peroxisome](../../../../D:%5C%E9%AB%98%E9%80%9A%E9%87%8F%E6%B5%8B%E5%BA%8F%E7%BB%93%E6%9E%9C%5CF14FTSSCKF1242_NEMpnqE%5Cannotation%5CKEGG%5CYK-Unigene.fa.htm#gene67) | 152 (1.31%) | ko04146 | Cellular Processes | Transport and catabolism |
| 68 | [Leukocyte transendothelial migration](../../../../D:%5C%E9%AB%98%E9%80%9A%E9%87%8F%E6%B5%8B%E5%BA%8F%E7%BB%93%E6%9E%9C%5CF14FTSSCKF1242_NEMpnqE%5Cannotation%5CKEGG%5CYK-Unigene.fa.htm#gene68) | 152 (1.31%) | ko04670 | Organismal Systems | Immune system |
| 69 | [Parkinson's disease](../../../../D:%5C%E9%AB%98%E9%80%9A%E9%87%8F%E6%B5%8B%E5%BA%8F%E7%BB%93%E6%9E%9C%5CF14FTSSCKF1242_NEMpnqE%5Cannotation%5CKEGG%5CYK-Unigene.fa.htm#gene69) | 150 (1.29%) | ko05012 | Human Diseases | Neurodegenerative diseases |
| 70 | [Retinol metabolism](../../../../D:%5C%E9%AB%98%E9%80%9A%E9%87%8F%E6%B5%8B%E5%BA%8F%E7%BB%93%E6%9E%9C%5CF14FTSSCKF1242_NEMpnqE%5Cannotation%5CKEGG%5CYK-Unigene.fa.htm#gene70) | 148 (1.27%) | ko00830 | Metabolism | Metabolism of cofactors and vitamins |
| 71 | [Prostate cancer](../../../../D:%5C%E9%AB%98%E9%80%9A%E9%87%8F%E6%B5%8B%E5%BA%8F%E7%BB%93%E6%9E%9C%5CF14FTSSCKF1242_NEMpnqE%5Cannotation%5CKEGG%5CYK-Unigene.fa.htm#gene71) | 148 (1.27%) | ko05215 | Human Diseases | Cancers: Specific types |
| 72 | [Starch and sucrose metabolism](../../../../D:%5C%E9%AB%98%E9%80%9A%E9%87%8F%E6%B5%8B%E5%BA%8F%E7%BB%93%E6%9E%9C%5CF14FTSSCKF1242_NEMpnqE%5Cannotation%5CKEGG%5CYK-Unigene.fa.htm#gene72) | 143 (1.23%) | ko00500 | Metabolism | Carbohydrate metabolism |
| 73 | [Phototransduction - fly](../../../../D:%5C%E9%AB%98%E9%80%9A%E9%87%8F%E6%B5%8B%E5%BA%8F%E7%BB%93%E6%9E%9C%5CF14FTSSCKF1242_NEMpnqE%5Cannotation%5CKEGG%5CYK-Unigene.fa.htm#gene73) | 141 (1.21%) | ko04745 | Organismal Systems | Sensory system |
| 74 | [Morphine addiction](../../../../D:%5C%E9%AB%98%E9%80%9A%E9%87%8F%E6%B5%8B%E5%BA%8F%E7%BB%93%E6%9E%9C%5CF14FTSSCKF1242_NEMpnqE%5Cannotation%5CKEGG%5CYK-Unigene.fa.htm#gene74) | 141 (1.21%) | ko05032 | Human Diseases | Substance dependence |
| 75 | [Carbohydrate digestion and absorption](../../../../D:%5C%E9%AB%98%E9%80%9A%E9%87%8F%E6%B5%8B%E5%BA%8F%E7%BB%93%E6%9E%9C%5CF14FTSSCKF1242_NEMpnqE%5Cannotation%5CKEGG%5CYK-Unigene.fa.htm#gene75) | 139 (1.19%) | ko04973 | Organismal Systems | Digestive system |
| 76 | [Drug metabolism - cytochrome P450](../../../../D:%5C%E9%AB%98%E9%80%9A%E9%87%8F%E6%B5%8B%E5%BA%8F%E7%BB%93%E6%9E%9C%5CF14FTSSCKF1242_NEMpnqE%5Cannotation%5CKEGG%5CYK-Unigene.fa.htm#gene76) | 139 (1.19%) | ko00982 | Metabolism | Xenobiotics biodegradation and metabolism |
| 77 | [Metabolism of xenobiotics by cytochrome P450](../../../../D:%5C%E9%AB%98%E9%80%9A%E9%87%8F%E6%B5%8B%E5%BA%8F%E7%BB%93%E6%9E%9C%5CF14FTSSCKF1242_NEMpnqE%5Cannotation%5CKEGG%5CYK-Unigene.fa.htm#gene77) | 137 (1.18%) | ko00980 | Metabolism | Xenobiotics biodegradation and metabolism |
| 78 | [Pathogenic Escherichia coli infection](../../../../D:%5C%E9%AB%98%E9%80%9A%E9%87%8F%E6%B5%8B%E5%BA%8F%E7%BB%93%E6%9E%9C%5CF14FTSSCKF1242_NEMpnqE%5Cannotation%5CKEGG%5CYK-Unigene.fa.htm#gene78) | 137 (1.18%) | ko05130 | Human Diseases | Infectious diseases: Bacterial |
| 79 | [Synaptic vesicle cycle](../../../../D:%5C%E9%AB%98%E9%80%9A%E9%87%8F%E6%B5%8B%E5%BA%8F%E7%BB%93%E6%9E%9C%5CF14FTSSCKF1242_NEMpnqE%5Cannotation%5CKEGG%5CYK-Unigene.fa.htm#gene79) | 135 (1.16%) | ko04721 | Organismal Systems | Nervous system |
| 80 | [Small cell lung cancer](../../../../D:%5C%E9%AB%98%E9%80%9A%E9%87%8F%E6%B5%8B%E5%BA%8F%E7%BB%93%E6%9E%9C%5CF14FTSSCKF1242_NEMpnqE%5Cannotation%5CKEGG%5CYK-Unigene.fa.htm#gene80) | 134 (1.15%) | ko05222 | Human Diseases | Cancers: Specific types |
| 81 | [Mineral absorption](../../../../D:%5C%E9%AB%98%E9%80%9A%E9%87%8F%E6%B5%8B%E5%BA%8F%E7%BB%93%E6%9E%9C%5CF14FTSSCKF1242_NEMpnqE%5Cannotation%5CKEGG%5CYK-Unigene.fa.htm#gene81) | 132 (1.13%) | ko04978 | Organismal Systems | Digestive system |
| 82 | [ABC transporters](../../../../D:%5C%E9%AB%98%E9%80%9A%E9%87%8F%E6%B5%8B%E5%BA%8F%E7%BB%93%E6%9E%9C%5CF14FTSSCKF1242_NEMpnqE%5Cannotation%5CKEGG%5CYK-Unigene.fa.htm#gene82) | 129 (1.11%) | ko02010 | Environmental Information Processing | Membrane transport |
| 83 | [Inositol phosphate metabolism](../../../../D:%5C%E9%AB%98%E9%80%9A%E9%87%8F%E6%B5%8B%E5%BA%8F%E7%BB%93%E6%9E%9C%5CF14FTSSCKF1242_NEMpnqE%5Cannotation%5CKEGG%5CYK-Unigene.fa.htm#gene83) | 127 (1.09%) | ko00562 | Metabolism | Carbohydrate metabolism |
| 84 | [Long-term depression](../../../../D:%5C%E9%AB%98%E9%80%9A%E9%87%8F%E6%B5%8B%E5%BA%8F%E7%BB%93%E6%9E%9C%5CF14FTSSCKF1242_NEMpnqE%5Cannotation%5CKEGG%5CYK-Unigene.fa.htm#gene84) | 126 (1.08%) | ko04730 | Organismal Systems | Nervous system |
| 85 | [Bacterial invasion of epithelial cells](../../../../D:%5C%E9%AB%98%E9%80%9A%E9%87%8F%E6%B5%8B%E5%BA%8F%E7%BB%93%E6%9E%9C%5CF14FTSSCKF1242_NEMpnqE%5Cannotation%5CKEGG%5CYK-Unigene.fa.htm#gene85) | 124 (1.06%) | ko05100 | Human Diseases | Infectious diseases: Bacterial |
| 86 | [Dorso-ventral axis formation](../../../../D:%5C%E9%AB%98%E9%80%9A%E9%87%8F%E6%B5%8B%E5%BA%8F%E7%BB%93%E6%9E%9C%5CF14FTSSCKF1242_NEMpnqE%5Cannotation%5CKEGG%5CYK-Unigene.fa.htm#gene86) | 124 (1.06%) | ko04320 | Organismal Systems | Development |
| 87 | [Vasopressin-regulated water reabsorption](../../../../D:%5C%E9%AB%98%E9%80%9A%E9%87%8F%E6%B5%8B%E5%BA%8F%E7%BB%93%E6%9E%9C%5CF14FTSSCKF1242_NEMpnqE%5Cannotation%5CKEGG%5CYK-Unigene.fa.htm#gene87) | 124 (1.06%) | ko04962 | Organismal Systems | Excretory system |
| 88 | [Fatty acid metabolism](../../../../D:%5C%E9%AB%98%E9%80%9A%E9%87%8F%E6%B5%8B%E5%BA%8F%E7%BB%93%E6%9E%9C%5CF14FTSSCKF1242_NEMpnqE%5Cannotation%5CKEGG%5CYK-Unigene.fa.htm#gene88) | 123 (1.06%) | ko00071 | Metabolism | Lipid metabolism |
| 89 | [Lysine degradation](../../../../D:%5C%E9%AB%98%E9%80%9A%E9%87%8F%E6%B5%8B%E5%BA%8F%E7%BB%93%E6%9E%9C%5CF14FTSSCKF1242_NEMpnqE%5Cannotation%5CKEGG%5CYK-Unigene.fa.htm#gene89) | 122 (1.05%) | ko00310 | Metabolism | Amino acid metabolism |
| 90 | [Amyotrophic lateral sclerosis (ALS)](../../../../D:%5C%E9%AB%98%E9%80%9A%E9%87%8F%E6%B5%8B%E5%BA%8F%E7%BB%93%E6%9E%9C%5CF14FTSSCKF1242_NEMpnqE%5Cannotation%5CKEGG%5CYK-Unigene.fa.htm#gene90) | 122 (1.05%) | ko05014 | Human Diseases | Neurodegenerative diseases |
| 91 | [Chagas disease (American trypanosomiasis)](../../../../D:%5C%E9%AB%98%E9%80%9A%E9%87%8F%E6%B5%8B%E5%BA%8F%E7%BB%93%E6%9E%9C%5CF14FTSSCKF1242_NEMpnqE%5Cannotation%5CKEGG%5CYK-Unigene.fa.htm#gene91) | 119 (1.02%) | ko05142 | Human Diseases | Infectious diseases: Parasitic |
| 92 | [Taste transduction](../../../../D:%5C%E9%AB%98%E9%80%9A%E9%87%8F%E6%B5%8B%E5%BA%8F%E7%BB%93%E6%9E%9C%5CF14FTSSCKF1242_NEMpnqE%5Cannotation%5CKEGG%5CYK-Unigene.fa.htm#gene92) | 119 (1.02%) | ko04742 | Organismal Systems | Sensory system |
| 93 | [T cell receptor signaling pathway](../../../../D:%5C%E9%AB%98%E9%80%9A%E9%87%8F%E6%B5%8B%E5%BA%8F%E7%BB%93%E6%9E%9C%5CF14FTSSCKF1242_NEMpnqE%5Cannotation%5CKEGG%5CYK-Unigene.fa.htm#gene93) | 117 (1%) | ko04660 | Organismal Systems | Immune system |
| 94 | [Glycerophospholipid metabolism](../../../../D:%5C%E9%AB%98%E9%80%9A%E9%87%8F%E6%B5%8B%E5%BA%8F%E7%BB%93%E6%9E%9C%5CF14FTSSCKF1242_NEMpnqE%5Cannotation%5CKEGG%5CYK-Unigene.fa.htm#gene94) | 116 (1%) | ko00564 | Metabolism | Lipid metabolism |
| 95 | [RNA degradation](../../../../D:%5C%E9%AB%98%E9%80%9A%E9%87%8F%E6%B5%8B%E5%BA%8F%E7%BB%93%E6%9E%9C%5CF14FTSSCKF1242_NEMpnqE%5Cannotation%5CKEGG%5CYK-Unigene.fa.htm#gene95) | 115 (0.99%) | ko03018 | Genetic Information Processing | Folding, sorting and degradation |
| 96 | [ErbB signaling pathway](../../../../D:%5C%E9%AB%98%E9%80%9A%E9%87%8F%E6%B5%8B%E5%BA%8F%E7%BB%93%E6%9E%9C%5CF14FTSSCKF1242_NEMpnqE%5Cannotation%5CKEGG%5CYK-Unigene.fa.htm#gene96) | 112 (0.96%) | ko04012 | Environmental Information Processing | Signal transduction |
| 97 | [Shigellosis](../../../../D:%5C%E9%AB%98%E9%80%9A%E9%87%8F%E6%B5%8B%E5%BA%8F%E7%BB%93%E6%9E%9C%5CF14FTSSCKF1242_NEMpnqE%5Cannotation%5CKEGG%5CYK-Unigene.fa.htm#gene97) | 109 (0.94%) | ko05131 | Human Diseases | Infectious diseases: Bacterial |
| 98 | [Drug metabolism - other enzymes](../../../../D:%5C%E9%AB%98%E9%80%9A%E9%87%8F%E6%B5%8B%E5%BA%8F%E7%BB%93%E6%9E%9C%5CF14FTSSCKF1242_NEMpnqE%5Cannotation%5CKEGG%5CYK-Unigene.fa.htm#gene98) | 109 (0.94%) | ko00983 | Metabolism | Xenobiotics biodegradation and metabolism |
| 99 | [Tryptophan metabolism](../../../../D:%5C%E9%AB%98%E9%80%9A%E9%87%8F%E6%B5%8B%E5%BA%8F%E7%BB%93%E6%9E%9C%5CF14FTSSCKF1242_NEMpnqE%5Cannotation%5CKEGG%5CYK-Unigene.fa.htm#gene99) | 106 (0.91%) | ko00380 | Metabolism | Amino acid metabolism |
| 100 | [Glycerolipid metabolism](../../../../D:%5C%E9%AB%98%E9%80%9A%E9%87%8F%E6%B5%8B%E5%BA%8F%E7%BB%93%E6%9E%9C%5CF14FTSSCKF1242_NEMpnqE%5Cannotation%5CKEGG%5CYK-Unigene.fa.htm#gene100) | 106 (0.91%) | ko00561 | Metabolism | Lipid metabolism |
| 101 | [Adipocytokine signaling pathway](../../../../D:%5C%E9%AB%98%E9%80%9A%E9%87%8F%E6%B5%8B%E5%BA%8F%E7%BB%93%E6%9E%9C%5CF14FTSSCKF1242_NEMpnqE%5Cannotation%5CKEGG%5CYK-Unigene.fa.htm#gene101) | 105 (0.9%) | ko04920 | Organismal Systems | Endocrine system |
| 102 | [Toxoplasmosis](../../../../D:%5C%E9%AB%98%E9%80%9A%E9%87%8F%E6%B5%8B%E5%BA%8F%E7%BB%93%E6%9E%9C%5CF14FTSSCKF1242_NEMpnqE%5Cannotation%5CKEGG%5CYK-Unigene.fa.htm#gene102) | 104 (0.89%) | ko05145 | Human Diseases | Infectious diseases: Parasitic |
| 103 | [PPAR signaling pathway](../../../../D:%5C%E9%AB%98%E9%80%9A%E9%87%8F%E6%B5%8B%E5%BA%8F%E7%BB%93%E6%9E%9C%5CF14FTSSCKF1242_NEMpnqE%5Cannotation%5CKEGG%5CYK-Unigene.fa.htm#gene103) | 102 (0.88%) | ko03320 | Organismal Systems | Endocrine system |
| 104 | [Antigen processing and presentation](../../../../D:%5C%E9%AB%98%E9%80%9A%E9%87%8F%E6%B5%8B%E5%BA%8F%E7%BB%93%E6%9E%9C%5CF14FTSSCKF1242_NEMpnqE%5Cannotation%5CKEGG%5CYK-Unigene.fa.htm#gene104) | 100 (0.86%) | ko04612 | Organismal Systems | Immune system |
| 105 | [Cocaine addiction](../../../../D:%5C%E9%AB%98%E9%80%9A%E9%87%8F%E6%B5%8B%E5%BA%8F%E7%BB%93%E6%9E%9C%5CF14FTSSCKF1242_NEMpnqE%5Cannotation%5CKEGG%5CYK-Unigene.fa.htm#gene105) | 99 (0.85%) | ko05030 | Human Diseases | Substance dependence |
| 106 | [TGF-beta signaling pathway](../../../../D:%5C%E9%AB%98%E9%80%9A%E9%87%8F%E6%B5%8B%E5%BA%8F%E7%BB%93%E6%9E%9C%5CF14FTSSCKF1242_NEMpnqE%5Cannotation%5CKEGG%5CYK-Unigene.fa.htm#gene106) | 99 (0.85%) | ko04350 | Environmental Information Processing | Signal transduction |
| 107 | [Measles](../../../../D:%5C%E9%AB%98%E9%80%9A%E9%87%8F%E6%B5%8B%E5%BA%8F%E7%BB%93%E6%9E%9C%5CF14FTSSCKF1242_NEMpnqE%5Cannotation%5CKEGG%5CYK-Unigene.fa.htm#gene107) | 98 (0.84%) | ko05162 | Human Diseases | Infectious diseases: Viral |
| 108 | [VEGF signaling pathway](../../../../D:%5C%E9%AB%98%E9%80%9A%E9%87%8F%E6%B5%8B%E5%BA%8F%E7%BB%93%E6%9E%9C%5CF14FTSSCKF1242_NEMpnqE%5Cannotation%5CKEGG%5CYK-Unigene.fa.htm#gene108) | 98 (0.84%) | ko04370 | Environmental Information Processing | Signal transduction |
| 109 | [Pentose and glucuronate interconversions](../../../../D:%5C%E9%AB%98%E9%80%9A%E9%87%8F%E6%B5%8B%E5%BA%8F%E7%BB%93%E6%9E%9C%5CF14FTSSCKF1242_NEMpnqE%5Cannotation%5CKEGG%5CYK-Unigene.fa.htm#gene109) | 98 (0.84%) | ko00040 | Metabolism | Carbohydrate metabolism |
| 110 | [Hepatitis C](../../../../D:%5C%E9%AB%98%E9%80%9A%E9%87%8F%E6%B5%8B%E5%BA%8F%E7%BB%93%E6%9E%9C%5CF14FTSSCKF1242_NEMpnqE%5Cannotation%5CKEGG%5CYK-Unigene.fa.htm#gene110) | 97 (0.83%) | ko05160 | Human Diseases | Infectious diseases: Viral |
| 111 | [Renal cell carcinoma](../../../../D:%5C%E9%AB%98%E9%80%9A%E9%87%8F%E6%B5%8B%E5%BA%8F%E7%BB%93%E6%9E%9C%5CF14FTSSCKF1242_NEMpnqE%5Cannotation%5CKEGG%5CYK-Unigene.fa.htm#gene111) | 97 (0.83%) | ko05211 | Human Diseases | Cancers: Specific types |
| 112 | [Glycolysis / Gluconeogenesis](../../../../D:%5C%E9%AB%98%E9%80%9A%E9%87%8F%E6%B5%8B%E5%BA%8F%E7%BB%93%E6%9E%9C%5CF14FTSSCKF1242_NEMpnqE%5Cannotation%5CKEGG%5CYK-Unigene.fa.htm#gene112) | 97 (0.83%) | ko00010 | Metabolism | Carbohydrate metabolism |
| 113 | [Epithelial cell signaling in Helicobacter pylori infection](../../../../D:%5C%E9%AB%98%E9%80%9A%E9%87%8F%E6%B5%8B%E5%BA%8F%E7%BB%93%E6%9E%9C%5CF14FTSSCKF1242_NEMpnqE%5Cannotation%5CKEGG%5CYK-Unigene.fa.htm#gene113) | 97 (0.83%) | ko05120 | Human Diseases | Infectious diseases: Bacterial |
| 114 | [Prion diseases](../../../../D:%5C%E9%AB%98%E9%80%9A%E9%87%8F%E6%B5%8B%E5%BA%8F%E7%BB%93%E6%9E%9C%5CF14FTSSCKF1242_NEMpnqE%5Cannotation%5CKEGG%5CYK-Unigene.fa.htm#gene114) | 97 (0.83%) | ko05020 | Human Diseases | Neurodegenerative diseases |
| 115 | [Glioma](../../../../D:%5C%E9%AB%98%E9%80%9A%E9%87%8F%E6%B5%8B%E5%BA%8F%E7%BB%93%E6%9E%9C%5CF14FTSSCKF1242_NEMpnqE%5Cannotation%5CKEGG%5CYK-Unigene.fa.htm#gene115) | 95 (0.82%) | ko05214 | Human Diseases | Cancers: Specific types |
| 116 | [B cell receptor signaling pathway](../../../../D:%5C%E9%AB%98%E9%80%9A%E9%87%8F%E6%B5%8B%E5%BA%8F%E7%BB%93%E6%9E%9C%5CF14FTSSCKF1242_NEMpnqE%5Cannotation%5CKEGG%5CYK-Unigene.fa.htm#gene116) | 94 (0.81%) | ko04662 | Organismal Systems | Immune system |
| 117 | [Tyrosine metabolism](../../../../D:%5C%E9%AB%98%E9%80%9A%E9%87%8F%E6%B5%8B%E5%BA%8F%E7%BB%93%E6%9E%9C%5CF14FTSSCKF1242_NEMpnqE%5Cannotation%5CKEGG%5CYK-Unigene.fa.htm#gene117) | 94 (0.81%) | ko00350 | Metabolism | Amino acid metabolism |
| 118 | [Ribosome biogenesis in eukaryotes](../../../../D:%5C%E9%AB%98%E9%80%9A%E9%87%8F%E6%B5%8B%E5%BA%8F%E7%BB%93%E6%9E%9C%5CF14FTSSCKF1242_NEMpnqE%5Cannotation%5CKEGG%5CYK-Unigene.fa.htm#gene118) | 92 (0.79%) | ko03008 | Genetic Information Processing | Translation |
| 119 | [Olfactory transduction](../../../../D:%5C%E9%AB%98%E9%80%9A%E9%87%8F%E6%B5%8B%E5%BA%8F%E7%BB%93%E6%9E%9C%5CF14FTSSCKF1242_NEMpnqE%5Cannotation%5CKEGG%5CYK-Unigene.fa.htm#gene119) | 91 (0.78%) | ko04740 | Organismal Systems | Sensory system |
| 120 | [Glutathione metabolism](../../../../D:%5C%E9%AB%98%E9%80%9A%E9%87%8F%E6%B5%8B%E5%BA%8F%E7%BB%93%E6%9E%9C%5CF14FTSSCKF1242_NEMpnqE%5Cannotation%5CKEGG%5CYK-Unigene.fa.htm#gene120) | 91 (0.78%) | ko00480 | Metabolism | Metabolism of other amino acids |
| 121 | [Notch signaling pathway](../../../../D:%5C%E9%AB%98%E9%80%9A%E9%87%8F%E6%B5%8B%E5%BA%8F%E7%BB%93%E6%9E%9C%5CF14FTSSCKF1242_NEMpnqE%5Cannotation%5CKEGG%5CYK-Unigene.fa.htm#gene121) | 90 (0.77%) | ko04330 | Environmental Information Processing | Signal transduction |
| 122 | [Renin-angiotensin system](../../../../D:%5C%E9%AB%98%E9%80%9A%E9%87%8F%E6%B5%8B%E5%BA%8F%E7%BB%93%E6%9E%9C%5CF14FTSSCKF1242_NEMpnqE%5Cannotation%5CKEGG%5CYK-Unigene.fa.htm#gene122) | 90 (0.77%) | ko04614 | Organismal Systems | Endocrine system |
| 123 | [Aldosterone-regulated sodium reabsorption](../../../../D:%5C%E9%AB%98%E9%80%9A%E9%87%8F%E6%B5%8B%E5%BA%8F%E7%BB%93%E6%9E%9C%5CF14FTSSCKF1242_NEMpnqE%5Cannotation%5CKEGG%5CYK-Unigene.fa.htm#gene123) | 90 (0.77%) | ko04960 | Organismal Systems | Excretory system |
| 124 | [Osteoclast differentiation](../../../../D:%5C%E9%AB%98%E9%80%9A%E9%87%8F%E6%B5%8B%E5%BA%8F%E7%BB%93%E6%9E%9C%5CF14FTSSCKF1242_NEMpnqE%5Cannotation%5CKEGG%5CYK-Unigene.fa.htm#gene124) | 89 (0.76%) | ko04380 | Organismal Systems | Development |
| 125 | [Natural killer cell mediated cytotoxicity](../../../../D:%5C%E9%AB%98%E9%80%9A%E9%87%8F%E6%B5%8B%E5%BA%8F%E7%BB%93%E6%9E%9C%5CF14FTSSCKF1242_NEMpnqE%5Cannotation%5CKEGG%5CYK-Unigene.fa.htm#gene125) | 88 (0.76%) | ko04650 | Organismal Systems | Immune system |
| 126 | [Arrhythmogenic right ventricular cardiomyopathy (ARVC)](../../../../D:%5C%E9%AB%98%E9%80%9A%E9%87%8F%E6%B5%8B%E5%BA%8F%E7%BB%93%E6%9E%9C%5CF14FTSSCKF1242_NEMpnqE%5Cannotation%5CKEGG%5CYK-Unigene.fa.htm#gene126) | 88 (0.76%) | ko05412 | Human Diseases | Cardiovascular diseases |
| 127 | [Arginine and proline metabolism](../../../../D:%5C%E9%AB%98%E9%80%9A%E9%87%8F%E6%B5%8B%E5%BA%8F%E7%BB%93%E6%9E%9C%5CF14FTSSCKF1242_NEMpnqE%5Cannotation%5CKEGG%5CYK-Unigene.fa.htm#gene127) | 87 (0.75%) | ko00330 | Metabolism | Amino acid metabolism |
| 128 | [Hedgehog signaling pathway](../../../../D:%5C%E9%AB%98%E9%80%9A%E9%87%8F%E6%B5%8B%E5%BA%8F%E7%BB%93%E6%9E%9C%5CF14FTSSCKF1242_NEMpnqE%5Cannotation%5CKEGG%5CYK-Unigene.fa.htm#gene128) | 87 (0.75%) | ko04340 | Environmental Information Processing | Signal transduction |
| 129 | [Pyruvate metabolism](../../../../D:%5C%E9%AB%98%E9%80%9A%E9%87%8F%E6%B5%8B%E5%BA%8F%E7%BB%93%E6%9E%9C%5CF14FTSSCKF1242_NEMpnqE%5Cannotation%5CKEGG%5CYK-Unigene.fa.htm#gene129) | 87 (0.75%) | ko00620 | Metabolism | Carbohydrate metabolism |
| 130 | [Hematopoietic cell lineage](../../../../D:%5C%E9%AB%98%E9%80%9A%E9%87%8F%E6%B5%8B%E5%BA%8F%E7%BB%93%E6%9E%9C%5CF14FTSSCKF1242_NEMpnqE%5Cannotation%5CKEGG%5CYK-Unigene.fa.htm#gene130) | 86 (0.74%) | ko04640 | Organismal Systems | Immune system |
| 131 | [Arachidonic acid metabolism](../../../../D:%5C%E9%AB%98%E9%80%9A%E9%87%8F%E6%B5%8B%E5%BA%8F%E7%BB%93%E6%9E%9C%5CF14FTSSCKF1242_NEMpnqE%5Cannotation%5CKEGG%5CYK-Unigene.fa.htm#gene131) | 86 (0.74%) | ko00590 | Metabolism | Lipid metabolism |
| 132 | [Non-small cell lung cancer](../../../../D:%5C%E9%AB%98%E9%80%9A%E9%87%8F%E6%B5%8B%E5%BA%8F%E7%BB%93%E6%9E%9C%5CF14FTSSCKF1242_NEMpnqE%5Cannotation%5CKEGG%5CYK-Unigene.fa.htm#gene132) | 85 (0.73%) | ko05223 | Human Diseases | Cancers: Specific types |
| 133 | [Colorectal cancer](../../../../D:%5C%E9%AB%98%E9%80%9A%E9%87%8F%E6%B5%8B%E5%BA%8F%E7%BB%93%E6%9E%9C%5CF14FTSSCKF1242_NEMpnqE%5Cannotation%5CKEGG%5CYK-Unigene.fa.htm#gene133) | 85 (0.73%) | ko05210 | Human Diseases | Cancers: Specific types |
| 134 | [Steroid hormone biosynthesis](../../../../D:%5C%E9%AB%98%E9%80%9A%E9%87%8F%E6%B5%8B%E5%BA%8F%E7%BB%93%E6%9E%9C%5CF14FTSSCKF1242_NEMpnqE%5Cannotation%5CKEGG%5CYK-Unigene.fa.htm#gene134) | 84 (0.72%) | ko00140 | Metabolism | Lipid metabolism |
| 135 | [Basal transcription factors](../../../../D:%5C%E9%AB%98%E9%80%9A%E9%87%8F%E6%B5%8B%E5%BA%8F%E7%BB%93%E6%9E%9C%5CF14FTSSCKF1242_NEMpnqE%5Cannotation%5CKEGG%5CYK-Unigene.fa.htm#gene135) | 83 (0.71%) | ko03022 | Genetic Information Processing | Transcription |
| 136 | [Fc epsilon RI signaling pathway](../../../../D:%5C%E9%AB%98%E9%80%9A%E9%87%8F%E6%B5%8B%E5%BA%8F%E7%BB%93%E6%9E%9C%5CF14FTSSCKF1242_NEMpnqE%5Cannotation%5CKEGG%5CYK-Unigene.fa.htm#gene136) | 81 (0.7%) | ko04664 | Organismal Systems | Immune system |
| 137 | [Galactose metabolism](../../../../D:%5C%E9%AB%98%E9%80%9A%E9%87%8F%E6%B5%8B%E5%BA%8F%E7%BB%93%E6%9E%9C%5CF14FTSSCKF1242_NEMpnqE%5Cannotation%5CKEGG%5CYK-Unigene.fa.htm#gene137) | 80 (0.69%) | ko00052 | Metabolism | Carbohydrate metabolism |
| 138 | [mTOR signaling pathway](../../../../D:%5C%E9%AB%98%E9%80%9A%E9%87%8F%E6%B5%8B%E5%BA%8F%E7%BB%93%E6%9E%9C%5CF14FTSSCKF1242_NEMpnqE%5Cannotation%5CKEGG%5CYK-Unigene.fa.htm#gene138) | 80 (0.69%) | ko04150 | Environmental Information Processing | Signal transduction |
| 139 | [NF-kappa B signaling pathway](../../../../D:%5C%E9%AB%98%E9%80%9A%E9%87%8F%E6%B5%8B%E5%BA%8F%E7%BB%93%E6%9E%9C%5CF14FTSSCKF1242_NEMpnqE%5Cannotation%5CKEGG%5CYK-Unigene.fa.htm#gene139) | 79 (0.68%) | ko04064 | Environmental Information Processing | Signal transduction |
| 140 | [Vitamin digestion and absorption](../../../../D:%5C%E9%AB%98%E9%80%9A%E9%87%8F%E6%B5%8B%E5%BA%8F%E7%BB%93%E6%9E%9C%5CF14FTSSCKF1242_NEMpnqE%5Cannotation%5CKEGG%5CYK-Unigene.fa.htm#gene140) | 78 (0.67%) | ko04977 | Organismal Systems | Digestive system |
| 141 | [Endometrial cancer](../../../../D:%5C%E9%AB%98%E9%80%9A%E9%87%8F%E6%B5%8B%E5%BA%8F%E7%BB%93%E6%9E%9C%5CF14FTSSCKF1242_NEMpnqE%5Cannotation%5CKEGG%5CYK-Unigene.fa.htm#gene141) | 77 (0.66%) | ko05213 | Human Diseases | Cancers: Specific types |
| 142 | [RNA polymerase](../../../../D:%5C%E9%AB%98%E9%80%9A%E9%87%8F%E6%B5%8B%E5%BA%8F%E7%BB%93%E6%9E%9C%5CF14FTSSCKF1242_NEMpnqE%5Cannotation%5CKEGG%5CYK-Unigene.fa.htm#gene142) | 76 (0.65%) | ko03020 | Genetic Information Processing | Transcription |
| 143 | [Proximal tubule bicarbonate reclamation](../../../../D:%5C%E9%AB%98%E9%80%9A%E9%87%8F%E6%B5%8B%E5%BA%8F%E7%BB%93%E6%9E%9C%5CF14FTSSCKF1242_NEMpnqE%5Cannotation%5CKEGG%5CYK-Unigene.fa.htm#gene143) | 76 (0.65%) | ko04964 | Organismal Systems | Excretory system |
| 144 | [Amino sugar and nucleotide sugar metabolism](../../../../D:%5C%E9%AB%98%E9%80%9A%E9%87%8F%E6%B5%8B%E5%BA%8F%E7%BB%93%E6%9E%9C%5CF14FTSSCKF1242_NEMpnqE%5Cannotation%5CKEGG%5CYK-Unigene.fa.htm#gene144) | 75 (0.64%) | ko00520 | Metabolism | Carbohydrate metabolism |
| 145 | [Basal cell carcinoma](../../../../D:%5C%E9%AB%98%E9%80%9A%E9%87%8F%E6%B5%8B%E5%BA%8F%E7%BB%93%E6%9E%9C%5CF14FTSSCKF1242_NEMpnqE%5Cannotation%5CKEGG%5CYK-Unigene.fa.htm#gene145) | 74 (0.64%) | ko05217 | Human Diseases | Cancers: Specific types |
| 146 | [Sphingolipid metabolism](../../../../D:%5C%E9%AB%98%E9%80%9A%E9%87%8F%E6%B5%8B%E5%BA%8F%E7%BB%93%E6%9E%9C%5CF14FTSSCKF1242_NEMpnqE%5Cannotation%5CKEGG%5CYK-Unigene.fa.htm#gene146) | 74 (0.64%) | ko00600 | Metabolism | Lipid metabolism |
| 147 | [Rheumatoid arthritis](../../../../D:%5C%E9%AB%98%E9%80%9A%E9%87%8F%E6%B5%8B%E5%BA%8F%E7%BB%93%E6%9E%9C%5CF14FTSSCKF1242_NEMpnqE%5Cannotation%5CKEGG%5CYK-Unigene.fa.htm#gene147) | 73 (0.63%) | ko05323 | Human Diseases | Immune diseases |
| 148 | [Legionellosis](../../../../D:%5C%E9%AB%98%E9%80%9A%E9%87%8F%E6%B5%8B%E5%BA%8F%E7%BB%93%E6%9E%9C%5CF14FTSSCKF1242_NEMpnqE%5Cannotation%5CKEGG%5CYK-Unigene.fa.htm#gene148) | 72 (0.62%) | ko05134 | Human Diseases | Infectious diseases: Bacterial |
| 149 | [Valine, leucine and isoleucine degradation](../../../../D:%5C%E9%AB%98%E9%80%9A%E9%87%8F%E6%B5%8B%E5%BA%8F%E7%BB%93%E6%9E%9C%5CF14FTSSCKF1242_NEMpnqE%5Cannotation%5CKEGG%5CYK-Unigene.fa.htm#gene149) | 72 (0.62%) | ko00280 | Metabolism | Amino acid metabolism |
| 150 | [Fat digestion and absorption](../../../../D:%5C%E9%AB%98%E9%80%9A%E9%87%8F%E6%B5%8B%E5%BA%8F%E7%BB%93%E6%9E%9C%5CF14FTSSCKF1242_NEMpnqE%5Cannotation%5CKEGG%5CYK-Unigene.fa.htm#gene150) | 71 (0.61%) | ko04975 | Organismal Systems | Digestive system |
| 151 | [Other types of O-glycan biosynthesis](../../../../D:%5C%E9%AB%98%E9%80%9A%E9%87%8F%E6%B5%8B%E5%BA%8F%E7%BB%93%E6%9E%9C%5CF14FTSSCKF1242_NEMpnqE%5Cannotation%5CKEGG%5CYK-Unigene.fa.htm#gene151) | 70 (0.6%) | ko00514 | Metabolism | Glycan biosynthesis and metabolism |
| 152 | [Cell adhesion molecules (CAMs)](../../../../D:%5C%E9%AB%98%E9%80%9A%E9%87%8F%E6%B5%8B%E5%BA%8F%E7%BB%93%E6%9E%9C%5CF14FTSSCKF1242_NEMpnqE%5Cannotation%5CKEGG%5CYK-Unigene.fa.htm#gene152) | 69 (0.59%) | ko04514 | Environmental Information Processing | Signaling molecules and interaction |
| 153 | [Chronic myeloid leukemia](../../../../D:%5C%E9%AB%98%E9%80%9A%E9%87%8F%E6%B5%8B%E5%BA%8F%E7%BB%93%E6%9E%9C%5CF14FTSSCKF1242_NEMpnqE%5Cannotation%5CKEGG%5CYK-Unigene.fa.htm#gene153) | 69 (0.59%) | ko05220 | Human Diseases | Cancers: Specific types |
| 154 | [Acute myeloid leukemia](../../../../D:%5C%E9%AB%98%E9%80%9A%E9%87%8F%E6%B5%8B%E5%BA%8F%E7%BB%93%E6%9E%9C%5CF14FTSSCKF1242_NEMpnqE%5Cannotation%5CKEGG%5CYK-Unigene.fa.htm#gene154) | 68 (0.58%) | ko05221 | Human Diseases | Cancers: Specific types |
| 155 | [Jak-STAT signaling pathway](../../../../D:%5C%E9%AB%98%E9%80%9A%E9%87%8F%E6%B5%8B%E5%BA%8F%E7%BB%93%E6%9E%9C%5CF14FTSSCKF1242_NEMpnqE%5Cannotation%5CKEGG%5CYK-Unigene.fa.htm#gene155) | 68 (0.58%) | ko04630 | Environmental Information Processing | Signal transduction |
| 156 | [p53 signaling pathway](../../../../D:%5C%E9%AB%98%E9%80%9A%E9%87%8F%E6%B5%8B%E5%BA%8F%E7%BB%93%E6%9E%9C%5CF14FTSSCKF1242_NEMpnqE%5Cannotation%5CKEGG%5CYK-Unigene.fa.htm#gene156) | 66 (0.57%) | ko04115 | Cellular Processes | Cell growth and death |
| 157 | [Porphyrin and chlorophyll metabolism](../../../../D:%5C%E9%AB%98%E9%80%9A%E9%87%8F%E6%B5%8B%E5%BA%8F%E7%BB%93%E6%9E%9C%5CF14FTSSCKF1242_NEMpnqE%5Cannotation%5CKEGG%5CYK-Unigene.fa.htm#gene157) | 66 (0.57%) | ko00860 | Metabolism | Metabolism of cofactors and vitamins |
| 158 | [Nicotine addiction](../../../../D:%5C%E9%AB%98%E9%80%9A%E9%87%8F%E6%B5%8B%E5%BA%8F%E7%BB%93%E6%9E%9C%5CF14FTSSCKF1242_NEMpnqE%5Cannotation%5CKEGG%5CYK-Unigene.fa.htm#gene158) | 65 (0.56%) | ko05033 | Human Diseases | Substance dependence |
| 159 | [African trypanosomiasis](../../../../D:%5C%E9%AB%98%E9%80%9A%E9%87%8F%E6%B5%8B%E5%BA%8F%E7%BB%93%E6%9E%9C%5CF14FTSSCKF1242_NEMpnqE%5Cannotation%5CKEGG%5CYK-Unigene.fa.htm#gene159) | 65 (0.56%) | ko05143 | Human Diseases | Infectious diseases: Parasitic |
| 160 | [Ascorbate and aldarate metabolism](../../../../D:%5C%E9%AB%98%E9%80%9A%E9%87%8F%E6%B5%8B%E5%BA%8F%E7%BB%93%E6%9E%9C%5CF14FTSSCKF1242_NEMpnqE%5Cannotation%5CKEGG%5CYK-Unigene.fa.htm#gene160) | 65 (0.56%) | ko00053 | Metabolism | Carbohydrate metabolism |
| 161 | [Pancreatic cancer](../../../../D:%5C%E9%AB%98%E9%80%9A%E9%87%8F%E6%B5%8B%E5%BA%8F%E7%BB%93%E6%9E%9C%5CF14FTSSCKF1242_NEMpnqE%5Cannotation%5CKEGG%5CYK-Unigene.fa.htm#gene161) | 62 (0.53%) | ko05212 | Human Diseases | Cancers: Specific types |
| 162 | [Cytokine-cytokine receptor interaction](../../../../D:%5C%E9%AB%98%E9%80%9A%E9%87%8F%E6%B5%8B%E5%BA%8F%E7%BB%93%E6%9E%9C%5CF14FTSSCKF1242_NEMpnqE%5Cannotation%5CKEGG%5CYK-Unigene.fa.htm#gene162) | 62 (0.53%) | ko04060 | Environmental Information Processing | Signaling molecules and interaction |
| 163 | [Phototransduction](../../../../D:%5C%E9%AB%98%E9%80%9A%E9%87%8F%E6%B5%8B%E5%BA%8F%E7%BB%93%E6%9E%9C%5CF14FTSSCKF1242_NEMpnqE%5Cannotation%5CKEGG%5CYK-Unigene.fa.htm#gene163) | 61 (0.52%) | ko04744 | Organismal Systems | Sensory system |
| 164 | [Fructose and mannose metabolism](../../../../D:%5C%E9%AB%98%E9%80%9A%E9%87%8F%E6%B5%8B%E5%BA%8F%E7%BB%93%E6%9E%9C%5CF14FTSSCKF1242_NEMpnqE%5Cannotation%5CKEGG%5CYK-Unigene.fa.htm#gene164) | 61 (0.52%) | ko00051 | Metabolism | Carbohydrate metabolism |
| 165 | [N-Glycan biosynthesis](../../../../D:%5C%E9%AB%98%E9%80%9A%E9%87%8F%E6%B5%8B%E5%BA%8F%E7%BB%93%E6%9E%9C%5CF14FTSSCKF1242_NEMpnqE%5Cannotation%5CKEGG%5CYK-Unigene.fa.htm#gene165) | 60 (0.52%) | ko00510 | Metabolism | Glycan biosynthesis and metabolism |
| 166 | [Pertussis](../../../../D:%5C%E9%AB%98%E9%80%9A%E9%87%8F%E6%B5%8B%E5%BA%8F%E7%BB%93%E6%9E%9C%5CF14FTSSCKF1242_NEMpnqE%5Cannotation%5CKEGG%5CYK-Unigene.fa.htm#gene166) | 58 (0.5%) | ko05133 | Human Diseases | Infectious diseases: Bacterial |
| 167 | [Cytosolic DNA-sensing pathway](../../../../D:%5C%E9%AB%98%E9%80%9A%E9%87%8F%E6%B5%8B%E5%BA%8F%E7%BB%93%E6%9E%9C%5CF14FTSSCKF1242_NEMpnqE%5Cannotation%5CKEGG%5CYK-Unigene.fa.htm#gene167) | 58 (0.5%) | ko04623 | Organismal Systems | Immune system |
| 168 | [Apoptosis](../../../../D:%5C%E9%AB%98%E9%80%9A%E9%87%8F%E6%B5%8B%E5%BA%8F%E7%BB%93%E6%9E%9C%5CF14FTSSCKF1242_NEMpnqE%5Cannotation%5CKEGG%5CYK-Unigene.fa.htm#gene168) | 58 (0.5%) | ko04210 | Cellular Processes | Cell growth and death |
| 169 | [Cysteine and methionine metabolism](../../../../D:%5C%E9%AB%98%E9%80%9A%E9%87%8F%E6%B5%8B%E5%BA%8F%E7%BB%93%E6%9E%9C%5CF14FTSSCKF1242_NEMpnqE%5Cannotation%5CKEGG%5CYK-Unigene.fa.htm#gene169) | 57 (0.49%) | ko00270 | Metabolism | Amino acid metabolism |
| 170 | [Phenylalanine metabolism](../../../../D:%5C%E9%AB%98%E9%80%9A%E9%87%8F%E6%B5%8B%E5%BA%8F%E7%BB%93%E6%9E%9C%5CF14FTSSCKF1242_NEMpnqE%5Cannotation%5CKEGG%5CYK-Unigene.fa.htm#gene170) | 56 (0.48%) | ko00360 | Metabolism | Amino acid metabolism |
| 171 | [Riboflavin metabolism](../../../../D:%5C%E9%AB%98%E9%80%9A%E9%87%8F%E6%B5%8B%E5%BA%8F%E7%BB%93%E6%9E%9C%5CF14FTSSCKF1242_NEMpnqE%5Cannotation%5CKEGG%5CYK-Unigene.fa.htm#gene171) | 55 (0.47%) | ko00740 | Metabolism | Metabolism of cofactors and vitamins |
| 172 | [Alanine, aspartate and glutamate metabolism](../../../../D:%5C%E9%AB%98%E9%80%9A%E9%87%8F%E6%B5%8B%E5%BA%8F%E7%BB%93%E6%9E%9C%5CF14FTSSCKF1242_NEMpnqE%5Cannotation%5CKEGG%5CYK-Unigene.fa.htm#gene172) | 55 (0.47%) | ko00250 | Metabolism | Amino acid metabolism |
| 173 | [Toll-like receptor signaling pathway](../../../../D:%5C%E9%AB%98%E9%80%9A%E9%87%8F%E6%B5%8B%E5%BA%8F%E7%BB%93%E6%9E%9C%5CF14FTSSCKF1242_NEMpnqE%5Cannotation%5CKEGG%5CYK-Unigene.fa.htm#gene173) | 54 (0.46%) | ko04620 | Organismal Systems | Immune system |
| 174 | [Citrate cycle (TCA cycle)](../../../../D:%5C%E9%AB%98%E9%80%9A%E9%87%8F%E6%B5%8B%E5%BA%8F%E7%BB%93%E6%9E%9C%5CF14FTSSCKF1242_NEMpnqE%5Cannotation%5CKEGG%5CYK-Unigene.fa.htm#gene174) | 54 (0.46%) | ko00020 | Metabolism | Carbohydrate metabolism |
| 175 | [Nucleotide excision repair](../../../../D:%5C%E9%AB%98%E9%80%9A%E9%87%8F%E6%B5%8B%E5%BA%8F%E7%BB%93%E6%9E%9C%5CF14FTSSCKF1242_NEMpnqE%5Cannotation%5CKEGG%5CYK-Unigene.fa.htm#gene175) | 53 (0.46%) | ko03420 | Genetic Information Processing | Replication and repair |
| 176 | [Aminoacyl-tRNA biosynthesis](../../../../D:%5C%E9%AB%98%E9%80%9A%E9%87%8F%E6%B5%8B%E5%BA%8F%E7%BB%93%E6%9E%9C%5CF14FTSSCKF1242_NEMpnqE%5Cannotation%5CKEGG%5CYK-Unigene.fa.htm#gene176) | 53 (0.46%) | ko00970 | Genetic Information Processing | Translation |
| 177 | [Melanoma](../../../../D:%5C%E9%AB%98%E9%80%9A%E9%87%8F%E6%B5%8B%E5%BA%8F%E7%BB%93%E6%9E%9C%5CF14FTSSCKF1242_NEMpnqE%5Cannotation%5CKEGG%5CYK-Unigene.fa.htm#gene177) | 52 (0.45%) | ko05218 | Human Diseases | Cancers: Specific types |
| 178 | [Propanoate metabolism](../../../../D:%5C%E9%AB%98%E9%80%9A%E9%87%8F%E6%B5%8B%E5%BA%8F%E7%BB%93%E6%9E%9C%5CF14FTSSCKF1242_NEMpnqE%5Cannotation%5CKEGG%5CYK-Unigene.fa.htm#gene178) | 51 (0.44%) | ko00640 | Metabolism | Carbohydrate metabolism |
| 179 | [Mucin type O-Glycan biosynthesis](../../../../D:%5C%E9%AB%98%E9%80%9A%E9%87%8F%E6%B5%8B%E5%BA%8F%E7%BB%93%E6%9E%9C%5CF14FTSSCKF1242_NEMpnqE%5Cannotation%5CKEGG%5CYK-Unigene.fa.htm#gene179) | 51 (0.44%) | ko00512 | Metabolism | Glycan biosynthesis and metabolism |
| 180 | [Systemic lupus erythematosus](../../../../D:%5C%E9%AB%98%E9%80%9A%E9%87%8F%E6%B5%8B%E5%BA%8F%E7%BB%93%E6%9E%9C%5CF14FTSSCKF1242_NEMpnqE%5Cannotation%5CKEGG%5CYK-Unigene.fa.htm#gene180) | 50 (0.43%) | ko05322 | Human Diseases | Immune diseases |
| 181 | [Fanconi anemia pathway](../../../../D:%5C%E9%AB%98%E9%80%9A%E9%87%8F%E6%B5%8B%E5%BA%8F%E7%BB%93%E6%9E%9C%5CF14FTSSCKF1242_NEMpnqE%5Cannotation%5CKEGG%5CYK-Unigene.fa.htm#gene181) | 50 (0.43%) | ko03460 | Genetic Information Processing | Replication and repair |
| 182 | [NOD-like receptor signaling pathway](../../../../D:%5C%E9%AB%98%E9%80%9A%E9%87%8F%E6%B5%8B%E5%BA%8F%E7%BB%93%E6%9E%9C%5CF14FTSSCKF1242_NEMpnqE%5Cannotation%5CKEGG%5CYK-Unigene.fa.htm#gene182) | 48 (0.41%) | ko04621 | Organismal Systems | Immune system |
| 183 | [Thyroid cancer](../../../../D:%5C%E9%AB%98%E9%80%9A%E9%87%8F%E6%B5%8B%E5%BA%8F%E7%BB%93%E6%9E%9C%5CF14FTSSCKF1242_NEMpnqE%5Cannotation%5CKEGG%5CYK-Unigene.fa.htm#gene183) | 47 (0.4%) | ko05216 | Human Diseases | Cancers: Specific types |
| 184 | [Glycine, serine and threonine metabolism](../../../../D:%5C%E9%AB%98%E9%80%9A%E9%87%8F%E6%B5%8B%E5%BA%8F%E7%BB%93%E6%9E%9C%5CF14FTSSCKF1242_NEMpnqE%5Cannotation%5CKEGG%5CYK-Unigene.fa.htm#gene184) | 46 (0.4%) | ko00260 | Metabolism | Amino acid metabolism |
| 185 | [DNA replication](../../../../D:%5C%E9%AB%98%E9%80%9A%E9%87%8F%E6%B5%8B%E5%BA%8F%E7%BB%93%E6%9E%9C%5CF14FTSSCKF1242_NEMpnqE%5Cannotation%5CKEGG%5CYK-Unigene.fa.htm#gene185) | 46 (0.4%) | ko03030 | Genetic Information Processing | Replication and repair |
| 186 | [Collecting duct acid secretion](../../../../D:%5C%E9%AB%98%E9%80%9A%E9%87%8F%E6%B5%8B%E5%BA%8F%E7%BB%93%E6%9E%9C%5CF14FTSSCKF1242_NEMpnqE%5Cannotation%5CKEGG%5CYK-Unigene.fa.htm#gene186) | 45 (0.39%) | ko04966 | Organismal Systems | Excretory system |
| 187 | [Butanoate metabolism](../../../../D:%5C%E9%AB%98%E9%80%9A%E9%87%8F%E6%B5%8B%E5%BA%8F%E7%BB%93%E6%9E%9C%5CF14FTSSCKF1242_NEMpnqE%5Cannotation%5CKEGG%5CYK-Unigene.fa.htm#gene187) | 45 (0.39%) | ko00650 | Metabolism | Carbohydrate metabolism |
| 188 | [Biosynthesis of unsaturated fatty acids](../../../../D:%5C%E9%AB%98%E9%80%9A%E9%87%8F%E6%B5%8B%E5%BA%8F%E7%BB%93%E6%9E%9C%5CF14FTSSCKF1242_NEMpnqE%5Cannotation%5CKEGG%5CYK-Unigene.fa.htm#gene188) | 44 (0.38%) | ko01040 | Metabolism | Lipid metabolism |
| 189 | [Maturity onset diabetes of the young](../../../../D:%5C%E9%AB%98%E9%80%9A%E9%87%8F%E6%B5%8B%E5%BA%8F%E7%BB%93%E6%9E%9C%5CF14FTSSCKF1242_NEMpnqE%5Cannotation%5CKEGG%5CYK-Unigene.fa.htm#gene189) | 44 (0.38%) | ko04950 | Human Diseases | Endocrine and metabolic diseases |
| 190 | [Linoleic acid metabolism](../../../../D:%5C%E9%AB%98%E9%80%9A%E9%87%8F%E6%B5%8B%E5%BA%8F%E7%BB%93%E6%9E%9C%5CF14FTSSCKF1242_NEMpnqE%5Cannotation%5CKEGG%5CYK-Unigene.fa.htm#gene190) | 43 (0.37%) | ko00591 | Metabolism | Lipid metabolism |
| 191 | [Proteasome](../../../../D:%5C%E9%AB%98%E9%80%9A%E9%87%8F%E6%B5%8B%E5%BA%8F%E7%BB%93%E6%9E%9C%5CF14FTSSCKF1242_NEMpnqE%5Cannotation%5CKEGG%5CYK-Unigene.fa.htm#gene191) | 43 (0.37%) | ko03050 | Genetic Information Processing | Folding, sorting and degradation |
| 192 | [Type II diabetes mellitus](../../../../D:%5C%E9%AB%98%E9%80%9A%E9%87%8F%E6%B5%8B%E5%BA%8F%E7%BB%93%E6%9E%9C%5CF14FTSSCKF1242_NEMpnqE%5Cannotation%5CKEGG%5CYK-Unigene.fa.htm#gene192) | 42 (0.36%) | ko04930 | Human Diseases | Endocrine and metabolic diseases |
| 193 | [Complement and coagulation cascades](../../../../D:%5C%E9%AB%98%E9%80%9A%E9%87%8F%E6%B5%8B%E5%BA%8F%E7%BB%93%E6%9E%9C%5CF14FTSSCKF1242_NEMpnqE%5Cannotation%5CKEGG%5CYK-Unigene.fa.htm#gene193) | 42 (0.36%) | ko04610 | Organismal Systems | Immune system |
| 194 | [MAPK signaling pathway - fly](../../../../D:%5C%E9%AB%98%E9%80%9A%E9%87%8F%E6%B5%8B%E5%BA%8F%E7%BB%93%E6%9E%9C%5CF14FTSSCKF1242_NEMpnqE%5Cannotation%5CKEGG%5CYK-Unigene.fa.htm#gene194) | 42 (0.36%) | ko04013 | Environmental Information Processing | Signal transduction |
| 195 | [beta-Alanine metabolism](../../../../D:%5C%E9%AB%98%E9%80%9A%E9%87%8F%E6%B5%8B%E5%BA%8F%E7%BB%93%E6%9E%9C%5CF14FTSSCKF1242_NEMpnqE%5Cannotation%5CKEGG%5CYK-Unigene.fa.htm#gene195) | 40 (0.34%) | ko00410 | Metabolism | Metabolism of other amino acids |
| 196 | [Glyoxylate and dicarboxylate metabolism](../../../../D:%5C%E9%AB%98%E9%80%9A%E9%87%8F%E6%B5%8B%E5%BA%8F%E7%BB%93%E6%9E%9C%5CF14FTSSCKF1242_NEMpnqE%5Cannotation%5CKEGG%5CYK-Unigene.fa.htm#gene196) | 40 (0.34%) | ko00630 | Metabolism | Carbohydrate metabolism |
| 197 | [Glycosphingolipid biosynthesis - lacto and neolacto series](../../../../D:%5C%E9%AB%98%E9%80%9A%E9%87%8F%E6%B5%8B%E5%BA%8F%E7%BB%93%E6%9E%9C%5CF14FTSSCKF1242_NEMpnqE%5Cannotation%5CKEGG%5CYK-Unigene.fa.htm#gene197) | 38 (0.33%) | ko00601 | Metabolism | Glycan biosynthesis and metabolism |
| 198 | [Fatty acid elongation](../../../../D:%5C%E9%AB%98%E9%80%9A%E9%87%8F%E6%B5%8B%E5%BA%8F%E7%BB%93%E6%9E%9C%5CF14FTSSCKF1242_NEMpnqE%5Cannotation%5CKEGG%5CYK-Unigene.fa.htm#gene198) | 38 (0.33%) | ko00062 | Metabolism | Lipid metabolism |
| 199 | [Bladder cancer](../../../../D:%5C%E9%AB%98%E9%80%9A%E9%87%8F%E6%B5%8B%E5%BA%8F%E7%BB%93%E6%9E%9C%5CF14FTSSCKF1242_NEMpnqE%5Cannotation%5CKEGG%5CYK-Unigene.fa.htm#gene199) | 38 (0.33%) | ko05219 | Human Diseases | Cancers: Specific types |
| 200 | [Ether lipid metabolism](../../../../D:%5C%E9%AB%98%E9%80%9A%E9%87%8F%E6%B5%8B%E5%BA%8F%E7%BB%93%E6%9E%9C%5CF14FTSSCKF1242_NEMpnqE%5Cannotation%5CKEGG%5CYK-Unigene.fa.htm#gene200) | 37 (0.32%) | ko00565 | Metabolism | Lipid metabolism |
| 201 | [Leishmaniasis](../../../../D:%5C%E9%AB%98%E9%80%9A%E9%87%8F%E6%B5%8B%E5%BA%8F%E7%BB%93%E6%9E%9C%5CF14FTSSCKF1242_NEMpnqE%5Cannotation%5CKEGG%5CYK-Unigene.fa.htm#gene201) | 37 (0.32%) | ko05140 | Human Diseases | Infectious diseases: Parasitic |
| 202 | [Glycosaminoglycan biosynthesis - heparan sulfate](../../../../D:%5C%E9%AB%98%E9%80%9A%E9%87%8F%E6%B5%8B%E5%BA%8F%E7%BB%93%E6%9E%9C%5CF14FTSSCKF1242_NEMpnqE%5Cannotation%5CKEGG%5CYK-Unigene.fa.htm#gene202) | 36 (0.31%) | ko00534 | Metabolism | Glycan biosynthesis and metabolism |
| 203 | [Regulation of autophagy](../../../../D:%5C%E9%AB%98%E9%80%9A%E9%87%8F%E6%B5%8B%E5%BA%8F%E7%BB%93%E6%9E%9C%5CF14FTSSCKF1242_NEMpnqE%5Cannotation%5CKEGG%5CYK-Unigene.fa.htm#gene203) | 36 (0.31%) | ko04140 | Cellular Processes | Transport and catabolism |
| 204 | [Nicotinate and nicotinamide metabolism](../../../../D:%5C%E9%AB%98%E9%80%9A%E9%87%8F%E6%B5%8B%E5%BA%8F%E7%BB%93%E6%9E%9C%5CF14FTSSCKF1242_NEMpnqE%5Cannotation%5CKEGG%5CYK-Unigene.fa.htm#gene204) | 36 (0.31%) | ko00760 | Metabolism | Metabolism of cofactors and vitamins |
| 205 | [Base excision repair](../../../../D:%5C%E9%AB%98%E9%80%9A%E9%87%8F%E6%B5%8B%E5%BA%8F%E7%BB%93%E6%9E%9C%5CF14FTSSCKF1242_NEMpnqE%5Cannotation%5CKEGG%5CYK-Unigene.fa.htm#gene205) | 35 (0.3%) | ko03410 | Genetic Information Processing | Replication and repair |
| 206 | [Homologous recombination](../../../../D:%5C%E9%AB%98%E9%80%9A%E9%87%8F%E6%B5%8B%E5%BA%8F%E7%BB%93%E6%9E%9C%5CF14FTSSCKF1242_NEMpnqE%5Cannotation%5CKEGG%5CYK-Unigene.fa.htm#gene206) | 35 (0.3%) | ko03440 | Genetic Information Processing | Replication and repair |
| 207 | [Glycosylphosphatidylinositol(GPI)-anchor biosynthesis](../../../../D:%5C%E9%AB%98%E9%80%9A%E9%87%8F%E6%B5%8B%E5%BA%8F%E7%BB%93%E6%9E%9C%5CF14FTSSCKF1242_NEMpnqE%5Cannotation%5CKEGG%5CYK-Unigene.fa.htm#gene207) | 34 (0.29%) | ko00563 | Metabolism | Glycan biosynthesis and metabolism |
| 208 | [Pentose phosphate pathway](../../../../D:%5C%E9%AB%98%E9%80%9A%E9%87%8F%E6%B5%8B%E5%BA%8F%E7%BB%93%E6%9E%9C%5CF14FTSSCKF1242_NEMpnqE%5Cannotation%5CKEGG%5CYK-Unigene.fa.htm#gene208) | 30 (0.26%) | ko00030 | Metabolism | Carbohydrate metabolism |
| 209 | [Malaria](../../../../D:%5C%E9%AB%98%E9%80%9A%E9%87%8F%E6%B5%8B%E5%BA%8F%E7%BB%93%E6%9E%9C%5CF14FTSSCKF1242_NEMpnqE%5Cannotation%5CKEGG%5CYK-Unigene.fa.htm#gene209) | 28 (0.24%) | ko05144 | Human Diseases | Infectious diseases: Parasitic |
| 210 | [Protein export](../../../../D:%5C%E9%AB%98%E9%80%9A%E9%87%8F%E6%B5%8B%E5%BA%8F%E7%BB%93%E6%9E%9C%5CF14FTSSCKF1242_NEMpnqE%5Cannotation%5CKEGG%5CYK-Unigene.fa.htm#gene210) | 27 (0.23%) | ko03060 | Genetic Information Processing | Folding, sorting and degradation |
| 211 | [Primary immunodeficiency](../../../../D:%5C%E9%AB%98%E9%80%9A%E9%87%8F%E6%B5%8B%E5%BA%8F%E7%BB%93%E6%9E%9C%5CF14FTSSCKF1242_NEMpnqE%5Cannotation%5CKEGG%5CYK-Unigene.fa.htm#gene211) | 27 (0.23%) | ko05340 | Human Diseases | Immune diseases |
| 212 | [alpha-Linolenic acid metabolism](../../../../D:%5C%E9%AB%98%E9%80%9A%E9%87%8F%E6%B5%8B%E5%BA%8F%E7%BB%93%E6%9E%9C%5CF14FTSSCKF1242_NEMpnqE%5Cannotation%5CKEGG%5CYK-Unigene.fa.htm#gene212) | 27 (0.23%) | ko00592 | Metabolism | Lipid metabolism |
| 213 | [Histidine metabolism](../../../../D:%5C%E9%AB%98%E9%80%9A%E9%87%8F%E6%B5%8B%E5%BA%8F%E7%BB%93%E6%9E%9C%5CF14FTSSCKF1242_NEMpnqE%5Cannotation%5CKEGG%5CYK-Unigene.fa.htm#gene213) | 27 (0.23%) | ko00340 | Metabolism | Amino acid metabolism |
| 214 | [RIG-I-like receptor signaling pathway](../../../../D:%5C%E9%AB%98%E9%80%9A%E9%87%8F%E6%B5%8B%E5%BA%8F%E7%BB%93%E6%9E%9C%5CF14FTSSCKF1242_NEMpnqE%5Cannotation%5CKEGG%5CYK-Unigene.fa.htm#gene214) | 27 (0.23%) | ko04622 | Organismal Systems | Immune system |
| 215 | [Mismatch repair](../../../../D:%5C%E9%AB%98%E9%80%9A%E9%87%8F%E6%B5%8B%E5%BA%8F%E7%BB%93%E6%9E%9C%5CF14FTSSCKF1242_NEMpnqE%5Cannotation%5CKEGG%5CYK-Unigene.fa.htm#gene215) | 26 (0.22%) | ko03430 | Genetic Information Processing | Replication and repair |
| 216 | [Terpenoid backbone biosynthesis](../../../../D:%5C%E9%AB%98%E9%80%9A%E9%87%8F%E6%B5%8B%E5%BA%8F%E7%BB%93%E6%9E%9C%5CF14FTSSCKF1242_NEMpnqE%5Cannotation%5CKEGG%5CYK-Unigene.fa.htm#gene216) | 25 (0.21%) | ko00900 | Metabolism | Metabolism of terpenoids and polyketides |
| 217 | [SNARE interactions in vesicular transport](../../../../D:%5C%E9%AB%98%E9%80%9A%E9%87%8F%E6%B5%8B%E5%BA%8F%E7%BB%93%E6%9E%9C%5CF14FTSSCKF1242_NEMpnqE%5Cannotation%5CKEGG%5CYK-Unigene.fa.htm#gene217) | 25 (0.21%) | ko04130 | Genetic Information Processing | Folding, sorting and degradation |
| 218 | [Autoimmune thyroid disease](../../../../D:%5C%E9%AB%98%E9%80%9A%E9%87%8F%E6%B5%8B%E5%BA%8F%E7%BB%93%E6%9E%9C%5CF14FTSSCKF1242_NEMpnqE%5Cannotation%5CKEGG%5CYK-Unigene.fa.htm#gene218) | 24 (0.21%) | ko05320 | Human Diseases | Immune diseases |
| 219 | [Staphylococcus aureus infection](../../../../D:%5C%E9%AB%98%E9%80%9A%E9%87%8F%E6%B5%8B%E5%BA%8F%E7%BB%93%E6%9E%9C%5CF14FTSSCKF1242_NEMpnqE%5Cannotation%5CKEGG%5CYK-Unigene.fa.htm#gene219) | 23 (0.2%) | ko05150 | Human Diseases | Infectious diseases: Bacterial |
| 220 | [Primary bile acid biosynthesis](../../../../D:%5C%E9%AB%98%E9%80%9A%E9%87%8F%E6%B5%8B%E5%BA%8F%E7%BB%93%E6%9E%9C%5CF14FTSSCKF1242_NEMpnqE%5Cannotation%5CKEGG%5CYK-Unigene.fa.htm#gene220) | 22 (0.19%) | ko00120 | Metabolism | Lipid metabolism |
| 221 | [Glycosphingolipid biosynthesis - globo series](../../../../D:%5C%E9%AB%98%E9%80%9A%E9%87%8F%E6%B5%8B%E5%BA%8F%E7%BB%93%E6%9E%9C%5CF14FTSSCKF1242_NEMpnqE%5Cannotation%5CKEGG%5CYK-Unigene.fa.htm#gene221) | 22 (0.19%) | ko00603 | Metabolism | Glycan biosynthesis and metabolism |
| 222 | [Ubiquinone and other terpenoid-quinone biosynthesis](../../../../D:%5C%E9%AB%98%E9%80%9A%E9%87%8F%E6%B5%8B%E5%BA%8F%E7%BB%93%E6%9E%9C%5CF14FTSSCKF1242_NEMpnqE%5Cannotation%5CKEGG%5CYK-Unigene.fa.htm#gene222) | 22 (0.19%) | ko00130 | Metabolism | Metabolism of cofactors and vitamins |
| 223 | [Circadian rhythm - fly](../../../../D:%5C%E9%AB%98%E9%80%9A%E9%87%8F%E6%B5%8B%E5%BA%8F%E7%BB%93%E6%9E%9C%5CF14FTSSCKF1242_NEMpnqE%5Cannotation%5CKEGG%5CYK-Unigene.fa.htm#gene223) | 21 (0.18%) | ko04711 | Organismal Systems | Environmental adaptation |
| 224 | [Other glycan degradation](../../../../D:%5C%E9%AB%98%E9%80%9A%E9%87%8F%E6%B5%8B%E5%BA%8F%E7%BB%93%E6%9E%9C%5CF14FTSSCKF1242_NEMpnqE%5Cannotation%5CKEGG%5CYK-Unigene.fa.htm#gene224) | 21 (0.18%) | ko00511 | Metabolism | Glycan biosynthesis and metabolism |
| 225 | [Glycosaminoglycan biosynthesis - chondroitin sulfate](../../../../D:%5C%E9%AB%98%E9%80%9A%E9%87%8F%E6%B5%8B%E5%BA%8F%E7%BB%93%E6%9E%9C%5CF14FTSSCKF1242_NEMpnqE%5Cannotation%5CKEGG%5CYK-Unigene.fa.htm#gene225) | 21 (0.18%) | ko00532 | Metabolism | Glycan biosynthesis and metabolism |
| 226 | [Pantothenate and CoA biosynthesis](../../../../D:%5C%E9%AB%98%E9%80%9A%E9%87%8F%E6%B5%8B%E5%BA%8F%E7%BB%93%E6%9E%9C%5CF14FTSSCKF1242_NEMpnqE%5Cannotation%5CKEGG%5CYK-Unigene.fa.htm#gene226) | 19 (0.16%) | ko00770 | Metabolism | Metabolism of cofactors and vitamins |
| 227 | [Folate biosynthesis](../../../../D:%5C%E9%AB%98%E9%80%9A%E9%87%8F%E6%B5%8B%E5%BA%8F%E7%BB%93%E6%9E%9C%5CF14FTSSCKF1242_NEMpnqE%5Cannotation%5CKEGG%5CYK-Unigene.fa.htm#gene227) | 19 (0.16%) | ko00790 | Metabolism | Metabolism of cofactors and vitamins |
| 228 | [D-Glutamine and D-glutamate metabolism](../../../../D:%5C%E9%AB%98%E9%80%9A%E9%87%8F%E6%B5%8B%E5%BA%8F%E7%BB%93%E6%9E%9C%5CF14FTSSCKF1242_NEMpnqE%5Cannotation%5CKEGG%5CYK-Unigene.fa.htm#gene228) | 18 (0.15%) | ko00471 | Metabolism | Metabolism of other amino acids |
| 229 | [Steroid biosynthesis](../../../../D:%5C%E9%AB%98%E9%80%9A%E9%87%8F%E6%B5%8B%E5%BA%8F%E7%BB%93%E6%9E%9C%5CF14FTSSCKF1242_NEMpnqE%5Cannotation%5CKEGG%5CYK-Unigene.fa.htm#gene229) | 18 (0.15%) | ko00100 | Metabolism | Lipid metabolism |
| 230 | [Cyanoamino acid metabolism](../../../../D:%5C%E9%AB%98%E9%80%9A%E9%87%8F%E6%B5%8B%E5%BA%8F%E7%BB%93%E6%9E%9C%5CF14FTSSCKF1242_NEMpnqE%5Cannotation%5CKEGG%5CYK-Unigene.fa.htm#gene230) | 15 (0.13%) | ko00460 | Metabolism | Metabolism of other amino acids |
| 231 | [Circadian rhythm - mammal](../../../../D:%5C%E9%AB%98%E9%80%9A%E9%87%8F%E6%B5%8B%E5%BA%8F%E7%BB%93%E6%9E%9C%5CF14FTSSCKF1242_NEMpnqE%5Cannotation%5CKEGG%5CYK-Unigene.fa.htm#gene231) | 15 (0.13%) | ko04710 | Organismal Systems | Environmental adaptation |
| 232 | [Sulfur relay system](../../../../D:%5C%E9%AB%98%E9%80%9A%E9%87%8F%E6%B5%8B%E5%BA%8F%E7%BB%93%E6%9E%9C%5CF14FTSSCKF1242_NEMpnqE%5Cannotation%5CKEGG%5CYK-Unigene.fa.htm#gene232) | 15 (0.13%) | ko04122 | Genetic Information Processing | Folding, sorting and degradation |
| 233 | [Sulfur metabolism](../../../../D:%5C%E9%AB%98%E9%80%9A%E9%87%8F%E6%B5%8B%E5%BA%8F%E7%BB%93%E6%9E%9C%5CF14FTSSCKF1242_NEMpnqE%5Cannotation%5CKEGG%5CYK-Unigene.fa.htm#gene233) | 15 (0.13%) | ko00920 | Metabolism | Energy metabolism |
| 234 | [Non-homologous end-joining](../../../../D:%5C%E9%AB%98%E9%80%9A%E9%87%8F%E6%B5%8B%E5%BA%8F%E7%BB%93%E6%9E%9C%5CF14FTSSCKF1242_NEMpnqE%5Cannotation%5CKEGG%5CYK-Unigene.fa.htm#gene234) | 15 (0.13%) | ko03450 | Genetic Information Processing | Replication and repair |
| 235 | [One carbon pool by folate](../../../../D:%5C%E9%AB%98%E9%80%9A%E9%87%8F%E6%B5%8B%E5%BA%8F%E7%BB%93%E6%9E%9C%5CF14FTSSCKF1242_NEMpnqE%5Cannotation%5CKEGG%5CYK-Unigene.fa.htm#gene235) | 15 (0.13%) | ko00670 | Metabolism | Metabolism of cofactors and vitamins |
| 236 | [Selenocompound metabolism](../../../../D:%5C%E9%AB%98%E9%80%9A%E9%87%8F%E6%B5%8B%E5%BA%8F%E7%BB%93%E6%9E%9C%5CF14FTSSCKF1242_NEMpnqE%5Cannotation%5CKEGG%5CYK-Unigene.fa.htm#gene236) | 14 (0.12%) | ko00450 | Metabolism | Metabolism of other amino acids |
| 237 | [Valine, leucine and isoleucine biosynthesis](../../../../D:%5C%E9%AB%98%E9%80%9A%E9%87%8F%E6%B5%8B%E5%BA%8F%E7%BB%93%E6%9E%9C%5CF14FTSSCKF1242_NEMpnqE%5Cannotation%5CKEGG%5CYK-Unigene.fa.htm#gene237) | 13 (0.11%) | ko00290 | Metabolism | Amino acid metabolism |
| 238 | [Glycosaminoglycan degradation](../../../../D:%5C%E9%AB%98%E9%80%9A%E9%87%8F%E6%B5%8B%E5%BA%8F%E7%BB%93%E6%9E%9C%5CF14FTSSCKF1242_NEMpnqE%5Cannotation%5CKEGG%5CYK-Unigene.fa.htm#gene238) | 12 (0.1%) | ko00531 | Metabolism | Glycan biosynthesis and metabolism |
| 239 | [Fatty acid biosynthesis](../../../../D:%5C%E9%AB%98%E9%80%9A%E9%87%8F%E6%B5%8B%E5%BA%8F%E7%BB%93%E6%9E%9C%5CF14FTSSCKF1242_NEMpnqE%5Cannotation%5CKEGG%5CYK-Unigene.fa.htm#gene239) | 12 (0.1%) | ko00061 | Metabolism | Lipid metabolism |
| 240 | [Glycosaminoglycan biosynthesis - keratan sulfate](../../../../D:%5C%E9%AB%98%E9%80%9A%E9%87%8F%E6%B5%8B%E5%BA%8F%E7%BB%93%E6%9E%9C%5CF14FTSSCKF1242_NEMpnqE%5Cannotation%5CKEGG%5CYK-Unigene.fa.htm#gene240) | 11 (0.09%) | ko00533 | Metabolism | Glycan biosynthesis and metabolism |
| 241 | [Caffeine metabolism](../../../../D:%5C%E9%AB%98%E9%80%9A%E9%87%8F%E6%B5%8B%E5%BA%8F%E7%BB%93%E6%9E%9C%5CF14FTSSCKF1242_NEMpnqE%5Cannotation%5CKEGG%5CYK-Unigene.fa.htm#gene241) | 10 (0.09%) | ko00232 | Metabolism | Biosynthesis of other secondary metabolites |
| 242 | [Asthma](../../../../D:%5C%E9%AB%98%E9%80%9A%E9%87%8F%E6%B5%8B%E5%BA%8F%E7%BB%93%E6%9E%9C%5CF14FTSSCKF1242_NEMpnqE%5Cannotation%5CKEGG%5CYK-Unigene.fa.htm#gene242) | 10 (0.09%) | ko05310 | Human Diseases | Immune diseases |
| 243 | [Taurine and hypotaurine metabolism](../../../../D:%5C%E9%AB%98%E9%80%9A%E9%87%8F%E6%B5%8B%E5%BA%8F%E7%BB%93%E6%9E%9C%5CF14FTSSCKF1242_NEMpnqE%5Cannotation%5CKEGG%5CYK-Unigene.fa.htm#gene243) | 9 (0.08%) | ko00430 | Metabolism | Metabolism of other amino acids |
| 244 | [Lipoic acid metabolism](../../../../D:%5C%E9%AB%98%E9%80%9A%E9%87%8F%E6%B5%8B%E5%BA%8F%E7%BB%93%E6%9E%9C%5CF14FTSSCKF1242_NEMpnqE%5Cannotation%5CKEGG%5CYK-Unigene.fa.htm#gene244) | 8 (0.07%) | ko00785 | Metabolism | Metabolism of cofactors and vitamins |
| 245 | [Phenylalanine, tyrosine and tryptophan biosynthesis](../../../../D:%5C%E9%AB%98%E9%80%9A%E9%87%8F%E6%B5%8B%E5%BA%8F%E7%BB%93%E6%9E%9C%5CF14FTSSCKF1242_NEMpnqE%5Cannotation%5CKEGG%5CYK-Unigene.fa.htm#gene245) | 8 (0.07%) | ko00400 | Metabolism | Amino acid metabolism |
| 246 | [D-Arginine and D-ornithine metabolism](../../../../D:%5C%E9%AB%98%E9%80%9A%E9%87%8F%E6%B5%8B%E5%BA%8F%E7%BB%93%E6%9E%9C%5CF14FTSSCKF1242_NEMpnqE%5Cannotation%5CKEGG%5CYK-Unigene.fa.htm#gene246) | 7 (0.06%) | ko00472 | Metabolism | Metabolism of other amino acids |
| 247 | [Type I diabetes mellitus](../../../../D:%5C%E9%AB%98%E9%80%9A%E9%87%8F%E6%B5%8B%E5%BA%8F%E7%BB%93%E6%9E%9C%5CF14FTSSCKF1242_NEMpnqE%5Cannotation%5CKEGG%5CYK-Unigene.fa.htm#gene247) | 7 (0.06%) | ko04940 | Human Diseases | Endocrine and metabolic diseases |
| 248 | [Allograft rejection](../../../../D:%5C%E9%AB%98%E9%80%9A%E9%87%8F%E6%B5%8B%E5%BA%8F%E7%BB%93%E6%9E%9C%5CF14FTSSCKF1242_NEMpnqE%5Cannotation%5CKEGG%5CYK-Unigene.fa.htm#gene248) | 6 (0.05%) | ko05330 | Human Diseases | Immune diseases |
| 249 | [Synthesis and degradation of ketone bodies](../../../../D:%5C%E9%AB%98%E9%80%9A%E9%87%8F%E6%B5%8B%E5%BA%8F%E7%BB%93%E6%9E%9C%5CF14FTSSCKF1242_NEMpnqE%5Cannotation%5CKEGG%5CYK-Unigene.fa.htm#gene249) | 6 (0.05%) | ko00072 | Metabolism | Lipid metabolism |
| 250 | [Lysine biosynthesis](../../../../D:%5C%E9%AB%98%E9%80%9A%E9%87%8F%E6%B5%8B%E5%BA%8F%E7%BB%93%E6%9E%9C%5CF14FTSSCKF1242_NEMpnqE%5Cannotation%5CKEGG%5CYK-Unigene.fa.htm#gene250) | 5 (0.04%) | ko00300 | Metabolism | Amino acid metabolism |
| 251 | [Butirosin and neomycin biosynthesis](../../../../D:%5C%E9%AB%98%E9%80%9A%E9%87%8F%E6%B5%8B%E5%BA%8F%E7%BB%93%E6%9E%9C%5CF14FTSSCKF1242_NEMpnqE%5Cannotation%5CKEGG%5CYK-Unigene.fa.htm#gene251) | 4 (0.03%) | ko00524 | Metabolism | Biosynthesis of other secondary metabolites |
| 252 | [Intestinal immune network for IgA production](../../../../D:%5C%E9%AB%98%E9%80%9A%E9%87%8F%E6%B5%8B%E5%BA%8F%E7%BB%93%E6%9E%9C%5CF14FTSSCKF1242_NEMpnqE%5Cannotation%5CKEGG%5CYK-Unigene.fa.htm#gene252) | 4 (0.03%) | ko04672 | Organismal Systems | Immune system |
| 253 | [Vitamin B6 metabolism](../../../../D:%5C%E9%AB%98%E9%80%9A%E9%87%8F%E6%B5%8B%E5%BA%8F%E7%BB%93%E6%9E%9C%5CF14FTSSCKF1242_NEMpnqE%5Cannotation%5CKEGG%5CYK-Unigene.fa.htm#gene253) | 4 (0.03%) | ko00750 | Metabolism | Metabolism of cofactors and vitamins |
| 254 | [Biotin metabolism](../../../../D:%5C%E9%AB%98%E9%80%9A%E9%87%8F%E6%B5%8B%E5%BA%8F%E7%BB%93%E6%9E%9C%5CF14FTSSCKF1242_NEMpnqE%5Cannotation%5CKEGG%5CYK-Unigene.fa.htm#gene254) | 3 (0.03%) | ko00780 | Metabolism | Metabolism of cofactors and vitamins |
| 255 | [Glycosphingolipid biosynthesis - ganglio series](../../../../D:%5C%E9%AB%98%E9%80%9A%E9%87%8F%E6%B5%8B%E5%BA%8F%E7%BB%93%E6%9E%9C%5CF14FTSSCKF1242_NEMpnqE%5Cannotation%5CKEGG%5CYK-Unigene.fa.htm#gene255) | 3 (0.03%) | ko00604 | Metabolism | Glycan biosynthesis and metabolism |
| 256 | [Polyketide sugar unit biosynthesis](../../../../D:%5C%E9%AB%98%E9%80%9A%E9%87%8F%E6%B5%8B%E5%BA%8F%E7%BB%93%E6%9E%9C%5CF14FTSSCKF1242_NEMpnqE%5Cannotation%5CKEGG%5CYK-Unigene.fa.htm#gene256) | 3 (0.03%) | ko00523 | Metabolism | Metabolism of terpenoids and polyketides |
| 257 | [Graft-versus-host disease](../../../../D:%5C%E9%AB%98%E9%80%9A%E9%87%8F%E6%B5%8B%E5%BA%8F%E7%BB%93%E6%9E%9C%5CF14FTSSCKF1242_NEMpnqE%5Cannotation%5CKEGG%5CYK-Unigene.fa.htm#gene257) | 2 (0.02%) | ko05332 | Human Diseases | Immune diseases |
| 258 | [Thiamine metabolism](../../../../D:%5C%E9%AB%98%E9%80%9A%E9%87%8F%E6%B5%8B%E5%BA%8F%E7%BB%93%E6%9E%9C%5CF14FTSSCKF1242_NEMpnqE%5Cannotation%5CKEGG%5CYK-Unigene.fa.htm#gene258) | 2 (0.02%) | ko00730 | Metabolism | Metabolism of cofactors and vitamins |
| 259 | [Insect hormone biosynthesis](../../../../D:%5C%E9%AB%98%E9%80%9A%E9%87%8F%E6%B5%8B%E5%BA%8F%E7%BB%93%E6%9E%9C%5CF14FTSSCKF1242_NEMpnqE%5Cannotation%5CKEGG%5CYK-Unigene.fa.htm#gene259) | 1 (0.01%) | ko00981 | Metabolism | Metabolism of terpenoids and polyketides |

| **#** | **Pathway** | **Differentially expressed genes** |
| --- | --- | --- |
| 1 | Metabolic pathways (no map in kegg database) | CL100.Contig1_YK, CL1002.Contig1_YK, CL1002.Contig2_YK, CL1023.Contig1_YK, CL1023.Contig2_YK, CL1027.Contig1_YK, CL103.Contig1_YK, CL103.Contig2_YK, CL103.Contig3_YK, CL103.Contig4_YK, CL103.Contig5_YK, CL103.Contig6_YK, CL103.Contig7_YK, CL103.Contig8_YK, CL1040.Contig2_YK, CL1058.Contig4_YK, CL1059.Contig1_YK, CL1059.Contig2_YK, CL1062.Contig10_YK, CL1062.Contig11_YK, CL1062.Contig1_YK, CL1062.Contig2_YK, CL1062.Contig3_YK, CL1062.Contig4_YK, CL1062.Contig5_YK, CL1062.Contig6_YK, CL1062.Contig7_YK, CL1062.Contig8_YK, CL1062.Contig9_YK, CL1083.Contig1_YK, CL1083.Contig2_YK, CL1083.Contig3_YK, CL1083.Contig4_YK, CL1101.Contig1_YK, CL1101.Contig2_YK, CL1101.Contig3_YK, CL1101.Contig4_YK, CL1102.Contig1_YK, CL1102.Contig2_YK, CL1102.Contig3_YK, CL1102.Contig4_YK, CL1103.Contig1_YK, CL1121.Contig1_YK, CL1121.Contig2_YK, CL1137.Contig1_YK, CL1137.Contig2_YK, CL1159.Contig1_YK, CL1159.Contig2_YK, CL1175.Contig1_YK, CL1175.Contig2_YK, CL1177.Contig1_YK, CL1177.Contig2_YK, CL1178.Contig1_YK, CL1178.Contig2_YK, CL1179.Contig1_YK, CL1179.Contig2_YK, CL1179.Contig3_YK, CL1179.Contig4_YK, CL118.Contig1_YK, CL118.Contig2_YK, CL118.Contig3_YK, CL1199.Contig1_YK, CL1203.Contig1_YK, CL1203.Contig2_YK, CL1206.Contig1_YK, CL1206.Contig2_YK, CL121.Contig2_YK, CL1233.Contig1_YK, CL1233.Contig2_YK, CL1234.Contig4_YK, CL1236.Contig1_YK, CL1236.Contig2_YK, CL1236.Contig3_YK, CL1236.Contig4_YK, CL1247.Contig1_YK, CL1247.Contig2_YK, CL1248.Contig1_YK, CL1248.Contig2_YK, CL1261.Contig1_YK, CL1261.Contig2_YK, CL1285.Contig1_YK, CL1285.Contig2_YK, CL1286.Contig1_YK, CL1286.Contig2_YK, CL1286.Contig3_YK, CL1286.Contig4_YK, CL1286.Contig5_YK, CL1286.Contig6_YK, CL1286.Contig7_YK, CL1286.Contig8_YK, CL1288.Contig1_YK, CL129.Contig1_YK, CL129.Contig2_YK, CL1293.Contig1_YK, CL1293.Contig2_YK, CL1302.Contig3_YK, CL1302.Contig4_YK, CL1308.Contig2_YK, CL1351.Contig1_YK, CL1351.Contig2_YK, CL1353.Contig1_YK, CL1353.Contig2_YK, CL1356.Contig1_YK, CL1356.Contig2_YK, CL1363.Contig1_YK, CL1363.Contig2_YK, CL1379.Contig1_YK, CL1379.Contig2_YK, CL1382.Contig1_YK, CL1382.Contig2_YK, CL1389.Contig1_YK, CL1389.Contig2_YK, CL139.Contig1_YK, CL139.Contig2_YK, CL139.Contig3_YK, CL139.Contig4_YK, CL139.Contig5_YK, CL1391.Contig1_YK, CL1391.Contig2_YK, CL1401.Contig1_YK, CL1401.Contig2_YK, CL141.Contig1_YK, CL141.Contig2_YK, CL1414.Contig1_YK, CL1414.Contig2_YK, CL1414.Contig3_YK, CL1414.Contig4_YK, CL1414.Contig5_YK, CL1414.Contig6_YK, CL1414.Contig7_YK, CL1414.Contig8_YK, CL1419.Contig1_YK, CL1419.Contig2_YK, CL1429.Contig1_YK, CL1429.Contig2_YK, CL1429.Contig3_YK, CL1429.Contig4_YK, CL1441.Contig1_YK, CL1441.Contig2_YK, CL1441.Contig3_YK, CL1441.Contig4_YK, CL1456.Contig3_YK, CL1465.Contig1_YK, CL1465.Contig2_YK, CL1466.Contig1_YK, CL1466.Contig2_YK, CL1469.Contig1_YK, CL1471.Contig1_YK, CL1473.Contig1_YK, CL1473.Contig2_YK, CL15.Contig10_YK, CL15.Contig11_YK, CL15.Contig12_YK, CL15.Contig13_YK, CL15.Contig14_YK, CL15.Contig15_YK, CL15.Contig16_YK, CL15.Contig17_YK, CL15.Contig18_YK, CL15.Contig19_YK, CL15.Contig1_YK, CL15.Contig20_YK, CL15.Contig21_YK, CL15.Contig22_YK, CL15.Contig23_YK, CL15.Contig24_YK, CL15.Contig2_YK, CL15.Contig3_YK, CL15.Contig4_YK, CL15.Contig5_YK, CL15.Contig6_YK, CL15.Contig7_YK, CL15.Contig8_YK, CL15.Contig9_YK, CL1500.Contig1_YK, CL1500.Contig2_YK, CL1500.Contig3_YK, CL1530.Contig1_YK, CL1530.Contig2_YK, CL1530.Contig3_YK, CL1530.Contig4_YK, CL1560.Contig1_YK, CL1560.Contig2_YK, CL1560.Contig3_YK, CL1569.Contig1_YK, CL1569.Contig2_YK, CL1573.Contig1_YK, CL1573.Contig2_YK, CL1585.Contig1_YK, CL1585.Contig2_YK, CL1594.Contig1_YK, CL1594.Contig2_YK, CL1611.Contig1_YK, CL1611.Contig2_YK, CL1643.Contig1_YK, CL1643.Contig2_YK, CL1643.Contig3_YK, CL1651.Contig1_YK, CL1652.Contig1_YK, CL1652.Contig2_YK, CL1654.Contig1_YK, CL1662.Contig1_YK, CL1662.Contig2_YK, CL1677.Contig1_YK, CL1677.Contig2_YK, CL1681.Contig1_YK, CL1681.Contig2_YK, CL1681.Contig3_YK, CL1682.Contig1_YK, CL1723.Contig1_YK, CL1723.Contig2_YK, CL1745.Contig1_YK, CL1745.Contig2_YK, CL1759.Contig1_YK, CL1759.Contig2_YK, CL1759.Contig3_YK, CL1763.Contig1_YK, CL1763.Contig2_YK, CL1766.Contig1_YK, CL1766.Contig2_YK, CL1766.Contig3_YK, CL1785.Contig1_YK, CL1785.Contig2_YK, CL1785.Contig3_YK, CL1791.Contig1_YK, CL1791.Contig2_YK, CL1791.Contig3_YK, CL1791.Contig4_YK, CL1798.Contig1_YK, CL1798.Contig2_YK, CL180.Contig2_YK, CL180.Contig3_YK, CL180.Contig4_YK, CL1802.Contig1_YK, CL1802.Contig2_YK, CL1807.Contig2_YK, CL1812.Contig1_YK, CL1812.Contig2_YK, CL1826.Contig1_YK, CL1826.Contig2_YK, CL1835.Contig1_YK, CL1835.Contig2_YK, CL1837.Contig1_YK, CL1837.Contig2_YK, CL1841.Contig1_YK, CL1841.Contig2_YK, CL1849.Contig1_YK, CL1849.Contig2_YK, CL1868.Contig1_YK, CL1871.Contig1_YK, CL1871.Contig2_YK, CL1888.Contig1_YK, CL1892.Contig1_YK, CL1892.Contig2_YK, CL1892.Contig3_YK, CL1892.Contig4_YK, CL1896.Contig1_YK, CL1896.Contig2_YK, CL1896.Contig3_YK, CL1901.Contig1_YK, CL1901.Contig2_YK, CL1903.Contig1_YK, CL1903.Contig2_YK, CL1933.Contig1_YK, CL1933.Contig2_YK, CL1935.Contig1_YK, CL1935.Contig2_YK, CL1941.Contig1_YK, CL1941.Contig2_YK, CL1943.Contig1_YK, CL1943.Contig2_YK, CL1952.Contig1_YK, CL1952.Contig2_YK, CL1961.Contig1_YK, CL1961.Contig2_YK, CL1969.Contig1_YK, CL1969.Contig2_YK, CL1973.Contig1_YK, CL1973.Contig2_YK, CL1974.Contig1_YK, CL1974.Contig2_YK, CL1978.Contig1_YK, CL1978.Contig2_YK, CL1994.Contig1_YK, CL1994.Contig2_YK, CL2004.Contig1_YK, CL2004.Contig2_YK, CL2010.Contig1_YK, CL2010.Contig2_YK, CL2042.Contig1_YK, CL2050.Contig1_YK, CL2050.Contig2_YK, CL2074.Contig1_YK, CL2074.Contig2_YK, CL2092.Contig1_YK, CL2092.Contig2_YK, CL2094.Contig1_YK, CL2094.Contig2_YK, CL2119.Contig1_YK, CL2119.Contig2_YK, CL2166.Contig1_YK, CL2166.Contig2_YK, CL2176.Contig1_YK, CL2176.Contig2_YK, CL2177.Contig1_YK, CL2177.Contig2_YK, CL2183.Contig1_YK, CL2183.Contig2_YK, CL2188.Contig1_YK, CL2188.Contig2_YK, CL2192.Contig1_YK, CL2192.Contig2_YK, CL2194.Contig1_YK, CL2194.Contig2_YK, CL2222.Contig1_YK, CL2222.Contig2_YK, CL2259.Contig1_YK, CL2259.Contig2_YK, CL2275.Contig1_YK, CL2275.Contig2_YK, CL2278.Contig1_YK, CL2278.Contig2_YK, CL2289.Contig2_YK, CL2293.Contig1_YK, CL2293.Contig2_YK, CL2316.Contig1_YK, CL2316.Contig2_YK, CL2322.Contig1_YK, CL2322.Contig2_YK, CL2325.Contig1_YK, CL2325.Contig2_YK, CL2334.Contig1_YK, CL2334.Contig2_YK, CL2391.Contig1_YK, CL2391.Contig2_YK, CL2391.Contig3_YK, CL2406.Contig1_YK, CL2406.Contig2_YK, CL2410.Contig1_YK, CL2410.Contig2_YK, CL2410.Contig3_YK, CL2416.Contig1_YK, CL2416.Contig2_YK, CL2419.Contig1_YK, CL243.Contig1_YK, CL243.Contig2_YK, CL2444.Contig1_YK, CL2444.Contig2_YK, CL2455.Contig1_YK, CL2455.Contig2_YK, CL2459.Contig1_YK, CL2459.Contig2_YK, CL2476.Contig1_YK, CL249.Contig1_YK, CL2492.Contig2_YK, CL2493.Contig1_YK, CL2502.Contig1_YK, CL2502.Contig2_YK, CL2503.Contig1_YK, CL2503.Contig2_YK, CL2504.Contig1_YK, CL2504.Contig2_YK, CL2531.Contig1_YK, CL2531.Contig2_YK, CL257.Contig1_YK, CL257.Contig2_YK, CL2580.Contig1_YK, CL2580.Contig2_YK, CL2594.Contig1_YK, CL2594.Contig2_YK, CL2594.Contig3_YK, CL2594.Contig4_YK, CL2608.Contig1_YK, CL2608.Contig2_YK, CL2626.Contig1_YK, CL2626.Contig2_YK, CL2637.Contig1_YK, CL2637.Contig2_YK, CL2649.Contig1_YK, CL2649.Contig2_YK, CL2685.Contig1_YK, CL2685.Contig2_YK, CL2711.Contig1_YK, CL2711.Contig2_YK, CL2717.Contig2_YK, CL2730.Contig1_YK, CL2730.Contig2_YK, CL2739.Contig1_YK, CL2746.Contig1_YK, CL2746.Contig2_YK, CL2746.Contig3_YK, CL2750.Contig1_YK, CL2750.Contig2_YK, CL2750.Contig3_YK, CL2751.Contig1_YK, CL2751.Contig2_YK, CL2772.Contig2_YK, CL2773.Contig1_YK, CL2798.Contig1_YK, CL2798.Contig2_YK, CL2807.Contig1_YK, CL2807.Contig2_YK, CL2809.Contig1_YK, CL2809.Contig2_YK, CL2834.Contig1_YK, CL2834.Contig2_YK, CL2840.Contig1_YK, CL2840.Contig2_YK, CL2841.Contig1_YK, CL2857.Contig1_YK, CL2857.Contig2_YK, CL286.Contig1_YK, CL286.Contig2_YK, CL286.Contig3_YK, CL2874.Contig1_YK, CL2877.Contig1_YK, CL2878.Contig1_YK, CL2900.Contig1_YK, CL2900.Contig2_YK, CL2907.Contig1_YK, CL2907.Contig2_YK, CL304.Contig1_YK, CL304.Contig2_YK, CL304.Contig3_YK, CL304.Contig4_YK, CL304.Contig6_YK, CL304.Contig7_YK, CL305.Contig1_YK, CL305.Contig2_YK, CL307.Contig10_YK, CL307.Contig11_YK, CL307.Contig12_YK, CL307.Contig1_YK, CL307.Contig2_YK, CL307.Contig3_YK, CL307.Contig4_YK, CL307.Contig5_YK, CL307.Contig6_YK, CL307.Contig7_YK, CL307.Contig8_YK, CL307.Contig9_YK, CL327.Contig1_YK, CL327.Contig2_YK, CL327.Contig3_YK, CL327.Contig4_YK, CL333.Contig1_YK, CL333.Contig2_YK, CL334.Contig1_YK, CL334.Contig2_YK, CL338.Contig1_YK, CL338.Contig2_YK, CL345.Contig1_YK, CL345.Contig2_YK, CL346.Contig1_YK, CL346.Contig2_YK, CL347.Contig1_YK, CL356.Contig2_YK, CL356.Contig3_YK, CL370.Contig1_YK, CL370.Contig2_YK, CL370.Contig3_YK, CL370.Contig4_YK, CL38.Contig1_YK, CL38.Contig2_YK, CL406.Contig1_YK, CL406.Contig2_YK, CL417.Contig1_YK, CL417.Contig2_YK, CL417.Contig3_YK, CL417.Contig4_YK, CL444.Contig1_YK, CL444.Contig2_YK, CL444.Contig3_YK, CL447.Contig1_YK, CL447.Contig2_YK, CL447.Contig3_YK, CL46.Contig1_YK, CL46.Contig2_YK, CL46.Contig3_YK, CL461.Contig1_YK, CL461.Contig2_YK, CL47.Contig1_YK, CL47.Contig2_YK, CL470.Contig1_YK, CL470.Contig2_YK, CL475.Contig1_YK, CL475.Contig2_YK, CL475.Contig3_YK, CL475.Contig4_YK, CL481.Contig1_YK, CL481.Contig2_YK, CL485.Contig1_YK, CL485.Contig3_YK, CL485.Contig4_YK, CL485.Contig5_YK, CL5.Contig1_YK, CL5.Contig3_YK, CL52.Contig1_YK, CL524.Contig1_YK, CL524.Contig2_YK, CL552.Contig1_YK, CL556.Contig1_YK, CL556.Contig2_YK, CL560.Contig1_YK, CL560.Contig2_YK, CL560.Contig3_YK, CL564.Contig10_YK, CL564.Contig13_YK, CL564.Contig14_YK, CL564.Contig16_YK, CL564.Contig2_YK, CL564.Contig3_YK, CL564.Contig5_YK, CL564.Contig6_YK, CL564.Contig7_YK, CL564.Contig8_YK, CL564.Contig9_YK, CL569.Contig1_YK, CL569.Contig2_YK, CL615.Contig3_YK, CL615.Contig4_YK, CL615.Contig5_YK, CL615.Contig7_YK, CL636.Contig1_YK, CL641.Contig1_YK, CL641.Contig2_YK, CL641.Contig3_YK, CL645.Contig1_YK, CL645.Contig2_YK, CL646.Contig1_YK, CL646.Contig2_YK, CL654.Contig1_YK, CL654.Contig2_YK, CL654.Contig3_YK, CL663.Contig1_YK, CL675.Contig2_YK, CL679.Contig1_YK, CL679.Contig2_YK, CL689.Contig1_YK, CL689.Contig2_YK, CL689.Contig3_YK, CL689.Contig4_YK, CL689.Contig5_YK, CL689.Contig6_YK, CL689.Contig7_YK, CL689.Contig8_YK, CL707.Contig1_YK, CL707.Contig2_YK, CL708.Contig1_YK, CL708.Contig2_YK, CL711.Contig1_YK, CL711.Contig2_YK, CL726.Contig1_YK, CL74.Contig1_YK, CL746.Contig1_YK, CL746.Contig2_YK, CL750.Contig1_YK, CL750.Contig2_YK, CL751.Contig2_YK, CL751.Contig3_YK, CL751.Contig4_YK, CL757.Contig1_YK, CL765.Contig1_YK, CL765.Contig2_YK, CL766.Contig1_YK, CL766.Contig2_YK, CL766.Contig3_YK, CL768.Contig1_YK, CL768.Contig2_YK, CL768.Contig3_YK, CL768.Contig4_YK, CL768.Contig5_YK, CL768.Contig6_YK, CL768.Contig7_YK, CL769.Contig1_YK, CL769.Contig2_YK, CL769.Contig3_YK, CL773.Contig1_YK, CL773.Contig2_YK, CL790.Contig1_YK, CL790.Contig2_YK, CL790.Contig3_YK, CL800.Contig1_YK, CL800.Contig2_YK, CL803.Contig1_YK, CL803.Contig2_YK, CL809.Contig1_YK, CL809.Contig2_YK, CL817.Contig1_YK, CL817.Contig3_YK, CL819.Contig1_YK, CL819.Contig2_YK, CL840.Contig1_YK, CL840.Contig2_YK, CL86.Contig1_YK, CL86.Contig2_YK, CL86.Contig3_YK, CL86.Contig4_YK, CL86.Contig5_YK, CL86.Contig6_YK, CL86.Contig7_YK, CL86.Contig8_YK, CL86.Contig9_YK, CL882.Contig1_YK, CL882.Contig2_YK, CL882.Contig3_YK, CL882.Contig4_YK, CL882.Contig5_YK, CL882.Contig6_YK, CL882.Contig7_YK, CL899.Contig1_YK, CL899.Contig2_YK, CL899.Contig3_YK, CL899.Contig4_YK, CL899.Contig5_YK, CL9.Contig1_YK, CL900.Contig1_YK, CL900.Contig2_YK, CL903.Contig1_YK, CL903.Contig2_YK, CL919.Contig1_YK, CL919.Contig2_YK, CL924.Contig10_YK, CL924.Contig11_YK, CL924.Contig12_YK, CL924.Contig1_YK, CL924.Contig2_YK, CL924.Contig3_YK, CL924.Contig4_YK, CL924.Contig5_YK, CL924.Contig6_YK, CL924.Contig7_YK, CL924.Contig8_YK, CL924.Contig9_YK, CL942.Contig1_YK, CL952.Contig1_YK, CL96.Contig1_YK, CL96.Contig2_YK, CL971.Contig1_YK, CL971.Contig2_YK, CL975.Contig1_YK, CL975.Contig2_YK, CL975.Contig3_YK, CL983.Contig1_YK, CL983.Contig2_YK, CL99.Contig2_YK, CL993.Contig1_YK, CL993.Contig2_YK, Unigene10015_YK, Unigene10028_YK, Unigene10029_YK, Unigene10030_YK, Unigene10031_YK, Unigene10082_YK, Unigene10127_YK, Unigene10176_YK, Unigene10189_YK, Unigene10190_YK, Unigene10209_YK, Unigene10210_YK, Unigene10211_YK, Unigene10224_YK, Unigene10262_YK, Unigene10263_YK, Unigene10340_YK, Unigene10354_YK, Unigene10408_YK, Unigene10410_YK, Unigene10501_YK, Unigene10510_YK, Unigene10531_YK, Unigene10540_YK, Unigene10594_YK, Unigene10600_YK, Unigene10638_YK, Unigene10639_YK, Unigene10650_YK, Unigene10651_YK, Unigene10665_YK, Unigene10719_YK, Unigene10755_YK, Unigene10761_YK, Unigene10762_YK, Unigene10777_YK, Unigene10783_YK, Unigene10785_YK, Unigene10810_YK, Unigene10813_YK, Unigene10826_YK, Unigene10827_YK, Unigene10828_YK, Unigene10835_YK, Unigene10876_YK, Unigene10895_YK, Unigene108_YK, Unigene10926_YK, Unigene10938_YK, Unigene10939_YK, Unigene10947_YK, Unigene10981_YK, Unigene10982_YK, Unigene10983_YK, Unigene11036_YK, Unigene11037_YK, Unigene11048_YK, Unigene11076_YK, Unigene11120_YK, Unigene11138_YK, Unigene11146_YK, Unigene11152_YK, Unigene11167_YK, Unigene11177_YK, Unigene11178_YK, Unigene11199_YK, Unigene11200_YK, Unigene11201_YK, Unigene11219_YK, Unigene11222_YK, Unigene11233_YK, Unigene11236_YK, Unigene11253_YK, Unigene11265_YK, Unigene11281_YK, Unigene112_YK, Unigene11303_YK, Unigene11331_YK, Unigene11353_YK, Unigene11354_YK, Unigene11378_YK, Unigene11404_YK, Unigene11439_YK, Unigene11459_YK, Unigene11460_YK, Unigene11461_YK, Unigene11466_YK, Unigene11467_YK, Unigene11540_YK, Unigene11592_YK, Unigene11668_YK, Unigene11669_YK, Unigene11707_YK, Unigene11728_YK, Unigene11739_YK, Unigene11740_YK, Unigene11745_YK, Unigene11746_YK, Unigene11750_YK, Unigene11814_YK, Unigene11828_YK, Unigene11841_YK, Unigene11855_YK, Unigene11856_YK, Unigene11861_YK, Unigene11870_YK, Unigene11909_YK, Unigene11912_YK, Unigene11916_YK, Unigene11926_YK, Unigene11927_YK, Unigene11932_YK, Unigene11938_YK, Unigene11944_YK, Unigene11946_YK, Unigene11947_YK, Unigene11953_YK, Unigene11954_YK, Unigene11956_YK, Unigene11965_YK, Unigene11977_YK, Unigene11978_YK, Unigene12004_YK, Unigene12025_YK, Unigene12049_YK, Unigene12064_YK, Unigene12077_YK, Unigene12081_YK, Unigene12113_YK, Unigene12131_YK, Unigene12132_YK, Unigene12155_YK, Unigene12223_YK, Unigene12259_YK, Unigene12274_YK, Unigene12320_YK, Unigene12359_YK, Unigene12363_YK, Unigene12364_YK, Unigene12376_YK, Unigene12448_YK, Unigene12455_YK, Unigene12513_YK, Unigene1251_YK, Unigene12522_YK, Unigene12599_YK, Unigene12600_YK, Unigene12622_YK, Unigene12636_YK, Unigene12638_YK, Unigene12639_YK, Unigene12642_YK, Unigene12699_YK, Unigene12723_YK, Unigene12764_YK, Unigene12784_YK, Unigene12797_YK, Unigene12805_YK, Unigene12819_YK, Unigene12887_YK, Unigene12930_YK, Unigene12970_YK, Unigene12982_YK, Unigene12986_YK, Unigene12990_YK, Unigene13013_YK, Unigene13019_YK, Unigene13038_YK, Unigene13043_YK, Unigene13046_YK, Unigene13063_YK, Unigene13064_YK, Unigene13065_YK, Unigene13070_YK, Unigene13071_YK, Unigene13076_YK, Unigene13100_YK, Unigene13132_YK, Unigene13134_YK, Unigene13144_YK, Unigene13150_YK, Unigene13151_YK, Unigene13153_YK, Unigene13157_YK, Unigene13166_YK, Unigene13181_YK, Unigene13189_YK, Unigene13190_YK, Unigene13197_YK, Unigene13198_YK, Unigene13207_YK, Unigene13209_YK, Unigene13210_YK, Unigene13214_YK, Unigene13226_YK, Unigene13229_YK, Unigene13231_YK, Unigene13232_YK, Unigene13234_YK, Unigene13246_YK, Unigene13255_YK, Unigene13256_YK, Unigene13269_YK, Unigene13270_YK, Unigene13274_YK, Unigene13282_YK, Unigene13283_YK, Unigene13287_YK, Unigene13289_YK, Unigene13297_YK, Unigene13306_YK, Unigene13309_YK, Unigene13338_YK, Unigene13345_YK, Unigene13353_YK, Unigene13355_YK, Unigene1335_YK, Unigene13375_YK, Unigene13382_YK, Unigene13385_YK, Unigene13389_YK, Unigene13390_YK, Unigene13392_YK, Unigene13401_YK, Unigene13405_YK, Unigene13407_YK, Unigene13415_YK, Unigene13440_YK, Unigene13441_YK, Unigene13444_YK, Unigene13458_YK, Unigene13460_YK, Unigene13462_YK, Unigene13466_YK, Unigene13472_YK, Unigene13473_YK, Unigene13480_YK, Unigene13483_YK, Unigene13485_YK, Unigene13494_YK, Unigene13507_YK, Unigene13526_YK, Unigene13527_YK, Unigene13539_YK, Unigene13547_YK, Unigene13565_YK, Unigene13572_YK, Unigene13582_YK, Unigene13584_YK, Unigene13586_YK, Unigene13592_YK, Unigene13607_YK, Unigene13608_YK, Unigene13611_YK, Unigene13617_YK, Unigene13623_YK, Unigene13639_YK, Unigene13641_YK, Unigene13643_YK, Unigene13673_YK, Unigene13691_YK, Unigene13694_YK, Unigene13696_YK, Unigene13708_YK, Unigene13728_YK, Unigene13744_YK, Unigene13752_YK, Unigene13753_YK, Unigene13777_YK, Unigene13785_YK, Unigene13801_YK, Unigene13818_YK, Unigene13825_YK, Unigene13829_YK, Unigene13832_YK, Unigene13841_YK, Unigene13847_YK, Unigene13848_YK, Unigene13857_YK, Unigene13876_YK, Unigene13878_YK, Unigene13895_YK, Unigene13896_YK, Unigene13899_YK, Unigene13926_YK, Unigene13928_YK, Unigene13963_YK, Unigene13965_YK, Unigene13972_YK, Unigene13974_YK, Unigene13979_YK, Unigene14004_YK, Unigene14006_YK, Unigene14019_YK, Unigene14021_YK, Unigene14028_YK, Unigene14030_YK, Unigene14034_YK, Unigene14039_YK, Unigene14049_YK, Unigene14052_YK, Unigene14053_YK, Unigene14054_YK, Unigene14062_YK, Unigene14070_YK, Unigene14075_YK, Unigene14083_YK, Unigene14087_YK, Unigene14099_YK, Unigene14117_YK, Unigene14121_YK, Unigene14131_YK, Unigene14132_YK, Unigene14152_YK, Unigene14160_YK, Unigene14165_YK, Unigene14192_YK, Unigene14196_YK, Unigene14202_YK, Unigene14204_YK, Unigene14206_YK, Unigene14215_YK, Unigene14216_YK, Unigene14219_YK, Unigene14232_YK, Unigene14258_YK, Unigene14264_YK, Unigene14279_YK, Unigene14283_YK, Unigene14284_YK, Unigene14294_YK, Unigene14297_YK, Unigene14309_YK, Unigene14329_YK, Unigene14341_YK, Unigene14343_YK, Unigene14352_YK, Unigene14355_YK, Unigene14368_YK, Unigene14377_YK, Unigene143_YK, Unigene14409_YK, Unigene14410_YK, Unigene14455_YK, Unigene14461_YK, Unigene14482_YK, Unigene14489_YK, Unigene14490_YK, Unigene14500_YK, Unigene14510_YK, Unigene14519_YK, Unigene14521_YK, Unigene14527_YK, Unigene14542_YK, Unigene14543_YK, Unigene14553_YK, Unigene14557_YK, Unigene14559_YK, Unigene14569_YK, Unigene14578_YK, Unigene14584_YK, Unigene14597_YK, Unigene145_YK, Unigene14606_YK, Unigene14610_YK, Unigene14636_YK, Unigene14675_YK, Unigene14682_YK, Unigene14685_YK, Unigene14690_YK, Unigene14692_YK, Unigene14698_YK, Unigene14700_YK, Unigene14709_YK, Unigene14723_YK, Unigene14733_YK, Unigene14736_YK, Unigene14744_YK, Unigene14745_YK, Unigene14754_YK, Unigene14767_YK, Unigene14789_YK, Unigene14804_YK, Unigene14814_YK, Unigene14817_YK, Unigene14820_YK, Unigene14827_YK, Unigene14837_YK, Unigene14870_YK, Unigene14876_YK, Unigene14905_YK, Unigene14912_YK, Unigene14923_YK, Unigene14925_YK, Unigene14927_YK, Unigene14934_YK, Unigene14936_YK, Unigene14943_YK, Unigene14953_YK, Unigene14959_YK, Unigene14971_YK, Unigene14977_YK, Unigene14991_YK, Unigene14995_YK, Unigene15040_YK, Unigene15047_YK, Unigene15050_YK, Unigene15053_YK, Unigene15054_YK, Unigene15058_YK, Unigene15079_YK, Unigene15082_YK, Unigene15091_YK, Unigene15095_YK, Unigene15130_YK, Unigene15147_YK, Unigene15162_YK, Unigene15171_YK, Unigene15174_YK, Unigene15177_YK, Unigene15184_YK, Unigene15187_YK, Unigene15203_YK, Unigene15217_YK, Unigene15220_YK, Unigene15223_YK, Unigene15234_YK, Unigene15237_YK, Unigene15238_YK, Unigene15253_YK, Unigene15278_YK, Unigene15283_YK, Unigene15299_YK, Unigene15302_YK, Unigene15329_YK, Unigene15330_YK, Unigene15332_YK, Unigene15342_YK, Unigene15345_YK, Unigene15375_YK, Unigene15384_YK, Unigene15411_YK, Unigene15415_YK, Unigene15426_YK, Unigene15430_YK, Unigene15449_YK, Unigene15461_YK, Unigene15478_YK, Unigene15501_YK, Unigene15504_YK, Unigene15508_YK, Unigene15510_YK, Unigene15523_YK, Unigene15529_YK, Unigene15534_YK, Unigene15541_YK, Unigene15550_YK, Unigene15575_YK, Unigene15595_YK, Unigene15606_YK, Unigene1560_YK, Unigene15618_YK, Unigene15674_YK, Unigene15687_YK, Unigene15688_YK, Unigene15718_YK, Unigene15726_YK, Unigene15729_YK, Unigene15764_YK, Unigene15785_YK, Unigene15796_YK, Unigene15811_YK, Unigene15813_YK, Unigene15815_YK, Unigene15830_YK, Unigene15850_YK, Unigene15883_YK, Unigene15894_YK, Unigene158_YK, Unigene15904_YK, Unigene15916_YK, Unigene15935_YK, Unigene15941_YK, Unigene1597_YK, Unigene16034_YK, Unigene16043_YK, Unigene16045_YK, Unigene1604_YK, Unigene16056_YK, Unigene16095_YK, Unigene16122_YK, Unigene16142_YK, Unigene16152_YK, Unigene16177_YK, Unigene16183_YK, Unigene16204_YK, Unigene16227_YK, Unigene16299_YK, Unigene16317_YK, Unigene16326_YK, Unigene16339_YK, Unigene16358_YK, Unigene16366_YK, Unigene16385_YK, Unigene16446_YK, Unigene16465_YK, Unigene16473_YK, Unigene16474_YK, Unigene16481_YK, Unigene16528_YK, Unigene16530_YK, Unigene16564_YK, Unigene16580_YK, Unigene16615_YK, Unigene16686_YK, Unigene16706_YK, Unigene16714_YK, Unigene16715_YK, Unigene16729_YK, Unigene16804_YK, Unigene1682_YK, Unigene16889_YK, Unigene17002_YK, Unigene17013_YK, Unigene17062_YK, Unigene17066_YK, Unigene17090_YK, Unigene17092_YK, Unigene17117_YK, Unigene17180_YK, Unigene17182_YK, Unigene17256_YK, Unigene1730_YK, Unigene17339_YK, Unigene17398_YK, Unigene17406_YK, Unigene17508_YK, Unigene17549_YK, Unigene17631_YK, Unigene17679_YK, Unigene17726_YK, Unigene17739_YK, Unigene1779_YK, Unigene17853_YK, Unigene17859_YK, Unigene17902_YK, Unigene179_YK, Unigene18156_YK, Unigene18192_YK, Unigene181_YK, Unigene18263_YK, Unigene18335_YK, Unigene1834_YK, Unigene18442_YK, Unigene18459_YK, Unigene18791_YK, Unigene18893_YK, Unigene18899_YK, Unigene18945_YK, Unigene19053_YK, Unigene19067_YK, Unigene19097_YK, Unigene19105_YK, Unigene19109_YK, Unigene19132_YK, Unigene1997_YK, Unigene1_YK, Unigene200_YK, Unigene2083_YK, Unigene2094_YK, Unigene2099_YK, Unigene2107_YK, Unigene2119_YK, Unigene2120_YK, Unigene2129_YK, Unigene2130_YK, Unigene2133_YK, Unigene2169_YK, Unigene2204_YK, Unigene2210_YK, Unigene222_YK, Unigene2267_YK, Unigene2274_YK, Unigene2310_YK, Unigene2312_YK, Unigene232_YK, Unigene2336_YK, Unigene2346_YK, Unigene2360_YK, Unigene2366_YK, Unigene241_YK, Unigene2425_YK, Unigene2426_YK, Unigene2439_YK, Unigene2461_YK, Unigene2474_YK, Unigene2490_YK, Unigene2503_YK, Unigene2592_YK, Unigene2596_YK, Unigene2612_YK, Unigene2628_YK, Unigene2629_YK, Unigene2642_YK, Unigene2658_YK, Unigene2664_YK, Unigene2677_YK, Unigene267_YK, Unigene2729_YK, Unigene2752_YK, Unigene2773_YK, Unigene2785_YK, Unigene2794_YK, Unigene2805_YK, Unigene2864_YK, Unigene2865_YK, Unigene2877_YK, Unigene2897_YK, Unigene2906_YK, Unigene2927_YK, Unigene2931_YK, Unigene2932_YK, Unigene2936_YK, Unigene2971_YK, Unigene2986_YK, Unigene2993_YK, Unigene29_YK, Unigene3000_YK, Unigene3003_YK, Unigene3004_YK, Unigene3008_YK, Unigene3022_YK, Unigene3030_YK, Unigene3031_YK, Unigene3049_YK, Unigene3059_YK, Unigene3060_YK, Unigene3092_YK, Unigene3098_YK, Unigene3104_YK, Unigene3105_YK, Unigene3106_YK, Unigene3128_YK, Unigene3133_YK, Unigene3140_YK, Unigene3153_YK, Unigene3158_YK, Unigene3163_YK, Unigene3207_YK, Unigene3221_YK, Unigene3229_YK, Unigene3242_YK, Unigene3243_YK, Unigene3244_YK, Unigene3245_YK, Unigene3275_YK, Unigene3284_YK, Unigene3305_YK, Unigene3314_YK, Unigene332_YK, Unigene3351_YK, Unigene335_YK, Unigene3361_YK, Unigene3381_YK, Unigene3390_YK, Unigene3397_YK, Unigene3407_YK, Unigene3413_YK, Unigene3426_YK, Unigene3435_YK, Unigene3444_YK, Unigene3450_YK, Unigene3457_YK, Unigene3460_YK, Unigene3471_YK, Unigene3475_YK, Unigene3494_YK, Unigene3511_YK, Unigene3528_YK, Unigene3530_YK, Unigene3536_YK, Unigene3612_YK, Unigene3620_YK, Unigene3639_YK, Unigene3642_YK, Unigene3643_YK, Unigene3671_YK, Unigene3679_YK, Unigene3685_YK, Unigene3687_YK, Unigene3688_YK, Unigene3701_YK, Unigene3703_YK, Unigene3704_YK, Unigene3705_YK, Unigene3731_YK, Unigene3737_YK, Unigene3779_YK, Unigene3793_YK, Unigene3794_YK, Unigene3798_YK, Unigene3806_YK, Unigene3807_YK, Unigene3814_YK, Unigene3823_YK, Unigene383_YK, Unigene3840_YK, Unigene3845_YK, Unigene3846_YK, Unigene3855_YK, Unigene3857_YK, Unigene3859_YK, Unigene3868_YK, Unigene3869_YK, Unigene3874_YK, Unigene3882_YK, Unigene3892_YK, Unigene3920_YK, Unigene3926_YK, Unigene3935_YK, Unigene3950_YK, Unigene3959_YK, Unigene3963_YK, Unigene3964_YK, Unigene3972_YK, Unigene3984_YK, Unigene4004_YK, Unigene4033_YK, Unigene4034_YK, Unigene4040_YK, Unigene4041_YK, Unigene4042_YK, Unigene4065_YK, Unigene4072_YK, Unigene4102_YK, Unigene4103_YK, Unigene4117_YK, Unigene4142_YK, Unigene4147_YK, Unigene4148_YK, Unigene4190_YK, Unigene4199_YK, Unigene4212_YK, Unigene4252_YK, Unigene4256_YK, Unigene4257_YK, Unigene4281_YK, Unigene4298_YK, Unigene4316_YK, Unigene4319_YK, Unigene4339_YK, Unigene4372_YK, Unigene4454_YK, Unigene4505_YK, Unigene4550_YK, Unigene456_YK, Unigene457_YK, Unigene4597_YK, Unigene4604_YK, Unigene4605_YK, Unigene4611_YK, Unigene4620_YK, Unigene4674_YK, Unigene4688_YK, Unigene469_YK, Unigene4806_YK, Unigene4829_YK, Unigene4852_YK, Unigene4859_YK, Unigene4884_YK, Unigene4892_YK, Unigene4897_YK, Unigene4902_YK, Unigene4912_YK, Unigene4919_YK, Unigene4992_YK, Unigene5001_YK, Unigene5018_YK, Unigene5019_YK, Unigene5076_YK, Unigene5084_YK, Unigene5110_YK, Unigene5133_YK, Unigene5135_YK, Unigene5136_YK, Unigene5140_YK, Unigene5179_YK, Unigene5181_YK, Unigene5184_YK, Unigene5193_YK, Unigene5196_YK, Unigene5222_YK, Unigene5226_YK, Unigene5238_YK, Unigene523_YK, Unigene5281_YK, Unigene5282_YK, Unigene5289_YK, Unigene5290_YK, Unigene5295_YK, Unigene5297_YK, Unigene52_YK, Unigene5303_YK, Unigene5314_YK, Unigene5321_YK, Unigene5322_YK, Unigene5365_YK, Unigene5368_YK, Unigene5379_YK, Unigene5382_YK, Unigene5412_YK, Unigene5452_YK, Unigene5476_YK, Unigene5477_YK, Unigene5512_YK, Unigene5530_YK, Unigene5546_YK, Unigene5553_YK, Unigene5578_YK, Unigene5581_YK, Unigene5597_YK, Unigene5604_YK, Unigene5623_YK, Unigene5635_YK, Unigene5647_YK, Unigene5670_YK, Unigene5677_YK, Unigene5687_YK, Unigene5696_YK, Unigene5720_YK, Unigene5723_YK, Unigene5754_YK, Unigene5772_YK, Unigene5782_YK, Unigene5794_YK, Unigene5817_YK, Unigene5818_YK, Unigene5832_YK, Unigene5860_YK, Unigene5896_YK, Unigene58_YK, Unigene5924_YK, Unigene5935_YK, Unigene5958_YK, Unigene5972_YK, Unigene5975_YK, Unigene5985_YK, Unigene5995_YK, Unigene5_YK, Unigene6009_YK, Unigene6010_YK, Unigene6012_YK, Unigene6013_YK, Unigene6048_YK, Unigene6093_YK, Unigene6094_YK, Unigene6095_YK, Unigene6125_YK, Unigene6131_YK, Unigene6134_YK, Unigene6143_YK, Unigene6166_YK, Unigene6175_YK, Unigene6179_YK, Unigene61_YK, Unigene6204_YK, Unigene6209_YK, Unigene6223_YK, Unigene6245_YK, Unigene6251_YK, Unigene6280_YK, Unigene6281_YK, Unigene6282_YK, Unigene6285_YK, Unigene6286_YK, Unigene6293_YK, Unigene6343_YK, Unigene6357_YK, Unigene6373_YK, Unigene641_YK, Unigene6455_YK, Unigene6457_YK, Unigene6458_YK, Unigene6487_YK, Unigene6502_YK, Unigene6503_YK, Unigene6508_YK, Unigene6519_YK, Unigene6527_YK, Unigene6584_YK, Unigene6592_YK, Unigene6598_YK, Unigene6615_YK, Unigene6638_YK, Unigene6642_YK, Unigene6643_YK, Unigene665_YK, Unigene6670_YK, Unigene6697_YK, Unigene66_YK, Unigene6713_YK, Unigene6740_YK, Unigene6757_YK, Unigene6789_YK, Unigene6795_YK, Unigene67_YK, Unigene6804_YK, Unigene6805_YK, Unigene6806_YK, Unigene6811_YK, Unigene6840_YK, Unigene6848_YK, Unigene6878_YK, Unigene6895_YK, Unigene6901_YK, Unigene6920_YK, Unigene6947_YK, Unigene6948_YK, Unigene6971_YK, Unigene69_YK, Unigene7019_YK, Unigene7040_YK, Unigene7060_YK, Unigene7062_YK, Unigene7067_YK, Unigene7076_YK, Unigene7086_YK, Unigene7087_YK, Unigene7111_YK, Unigene7119_YK, Unigene7120_YK, Unigene7139_YK, Unigene7183_YK, Unigene7184_YK, Unigene7185_YK, Unigene7228_YK, Unigene7229_YK, Unigene723_YK, Unigene7310_YK, Unigene7367_YK, Unigene7371_YK, Unigene7511_YK, Unigene753_YK, Unigene7558_YK, Unigene7559_YK, Unigene7563_YK, Unigene7589_YK, Unigene7592_YK, Unigene7661_YK, Unigene7668_YK, Unigene7669_YK, Unigene7737_YK, Unigene7797_YK, Unigene7833_YK, Unigene7834_YK, Unigene7835_YK, Unigene7844_YK, Unigene7845_YK, Unigene7846_YK, Unigene7882_YK, Unigene7889_YK, Unigene7898_YK, Unigene7899_YK, Unigene7900_YK, Unigene7901_YK, Unigene7942_YK, Unigene7943_YK, Unigene7945_YK, Unigene7994_YK, Unigene7995_YK, Unigene8003_YK, Unigene8035_YK, Unigene8050_YK, Unigene8059_YK, Unigene8147_YK, Unigene8152_YK, Unigene8158_YK, Unigene828_YK, Unigene8304_YK, Unigene8327_YK, Unigene8361_YK, Unigene8385_YK, Unigene8393_YK, Unigene8394_YK, Unigene8496_YK, Unigene84_YK, Unigene8583_YK, Unigene8592_YK, Unigene8607_YK, Unigene8616_YK, Unigene8617_YK, Unigene8618_YK, Unigene8719_YK, Unigene8722_YK, Unigene8773_YK, Unigene8805_YK, Unigene8806_YK, Unigene8807_YK, Unigene8848_YK, Unigene8849_YK, Unigene8867_YK, Unigene8868_YK, Unigene8906_YK, Unigene8938_YK, Unigene8964_YK, Unigene8975_YK, Unigene8990_YK, Unigene9007_YK, Unigene9168_YK, Unigene9169_YK, Unigene9179_YK, Unigene9180_YK, Unigene9192_YK, Unigene9234_YK, Unigene9256_YK, Unigene9269_YK, Unigene9313_YK, Unigene9315_YK, Unigene9320_YK, Unigene9359_YK, Unigene9360_YK, Unigene9370_YK, Unigene9411_YK, Unigene9412_YK, Unigene9420_YK, Unigene9457_YK, Unigene9470_YK, Unigene9512_YK, Unigene9513_YK, Unigene9517_YK, Unigene9644_YK, Unigene9646_YK, Unigene9693_YK, Unigene9735_YK, Unigene9736_YK, Unigene9780_YK, Unigene9781_YK, Unigene978_YK, Unigene979_YK, Unigene9854_YK, Unigene9856_YK, Unigene9857_YK, Unigene9876_YK, Unigene9877_YK, Unigene9891_YK, Unigene9903_YK, Unigene9904_YK, Unigene9962_YK, Unigene9983_YK, Unigene9984_YK, Unigene9985_YK, Unigene9987_YK, Unigene9988_YK, Unigene9991_YK |
| 2 | [Lysosome](../../../../D:%5C高通量测序结果%5CF14FTSSCKF1242_NEMpnqE%5Cannotation%5CKEGG%5CYK-Unigene.fa_map%5Cmap04142.html) | CL1002.Contig1_YK, CL1002.Contig2_YK, CL1027.Contig1_YK, CL1152.Contig1_YK, CL1152.Contig2_YK, CL1160.Contig1_YK, CL1160.Contig2_YK, CL1264.Contig1_YK, CL1264.Contig2_YK, CL1286.Contig1_YK, CL1286.Contig2_YK, CL1286.Contig3_YK, CL1286.Contig4_YK, CL1286.Contig5_YK, CL1286.Contig6_YK, CL1286.Contig7_YK, CL1286.Contig8_YK, CL1310.Contig3_YK, CL1310.Contig5_YK, CL1361.Contig1_YK, CL1399.Contig1_YK, CL1399.Contig2_YK, CL1399.Contig3_YK, CL1399.Contig4_YK, CL1399.Contig5_YK, CL1399.Contig6_YK, CL1399.Contig8_YK, CL1399.Contig9_YK, CL1420.Contig1_YK, CL1478.Contig1_YK, CL1478.Contig2_YK, CL169.Contig1_YK, CL169.Contig2_YK, CL169.Contig3_YK, CL169.Contig4_YK, CL1723.Contig1_YK, CL1723.Contig2_YK, CL173.Contig1_YK, CL173.Contig2_YK, CL1737.Contig1_YK, CL1737.Contig2_YK, CL1760.Contig1_YK, CL1793.Contig3_YK, CL1826.Contig1_YK, CL1826.Contig2_YK, CL1844.Contig1_YK, CL1844.Contig2_YK, CL1846.Contig1_YK, CL1846.Contig2_YK, CL187.Contig1_YK, CL187.Contig2_YK, CL1935.Contig1_YK, CL1935.Contig2_YK, CL1936.Contig1_YK, CL1960.Contig1_YK, CL1960.Contig2_YK, CL1987.Contig1_YK, CL1987.Contig2_YK, CL1987.Contig3_YK, CL1992.Contig1_YK, CL1992.Contig2_YK, CL1997.Contig1_YK, CL1997.Contig2_YK, CL1998.Contig1_YK, CL2112.Contig1_YK, CL2112.Contig2_YK, CL2115.Contig1_YK, CL2115.Contig2_YK, CL2115.Contig3_YK, CL2144.Contig1_YK, CL2144.Contig2_YK, CL2144.Contig3_YK, CL2185.Contig1_YK, CL2249.Contig2_YK, CL2273.Contig1_YK, CL2273.Contig2_YK, CL2282.Contig1_YK, CL2282.Contig2_YK, CL2342.Contig1_YK, CL2342.Contig2_YK, CL2382.Contig1_YK, CL2397.Contig2_YK, CL2434.Contig1_YK, CL2448.Contig1_YK, CL2448.Contig2_YK, CL2480.Contig1_YK, CL2480.Contig2_YK, CL252.Contig1_YK, CL2533.Contig1_YK, CL2533.Contig2_YK, CL2537.Contig1_YK, CL2537.Contig2_YK, CL2566.Contig1_YK, CL2566.Contig2_YK, CL2640.Contig1_YK, CL2640.Contig2_YK, CL2677.Contig1_YK, CL2714.Contig1_YK, CL2714.Contig2_YK, CL2742.Contig1_YK, CL2750.Contig3_YK, CL2761.Contig1_YK, CL2761.Contig2_YK, CL2877.Contig1_YK, CL288.Contig1_YK, CL288.Contig2_YK, CL297.Contig10_YK, CL297.Contig11_YK, CL297.Contig12_YK, CL297.Contig13_YK, CL297.Contig14_YK, CL297.Contig15_YK, CL297.Contig16_YK, CL297.Contig1_YK, CL297.Contig2_YK, CL297.Contig3_YK, CL297.Contig4_YK, CL297.Contig5_YK, CL297.Contig6_YK, CL297.Contig7_YK, CL297.Contig8_YK, CL297.Contig9_YK, CL310.Contig1_YK, CL310.Contig2_YK, CL393.Contig10_YK, CL393.Contig2_YK, CL393.Contig3_YK, CL393.Contig4_YK, CL393.Contig5_YK, CL393.Contig6_YK, CL393.Contig7_YK, CL393.Contig9_YK, CL443.Contig1_YK, CL443.Contig2_YK, CL443.Contig3_YK, CL513.Contig1_YK, CL513.Contig2_YK, CL513.Contig3_YK, CL513.Contig4_YK, CL513.Contig5_YK, CL604.Contig1_YK, CL604.Contig2_YK, CL604.Contig3_YK, CL604.Contig4_YK, CL604.Contig5_YK, CL604.Contig6_YK, CL604.Contig7_YK, CL66.Contig1_YK, CL66.Contig2_YK, CL660.Contig1_YK, CL671.Contig1_YK, CL671.Contig2_YK, CL707.Contig1_YK, CL707.Contig2_YK, CL727.Contig1_YK, CL727.Contig2_YK, CL742.Contig3_YK, CL742.Contig4_YK, CL746.Contig2_YK, CL808.Contig1_YK, CL808.Contig2_YK, CL808.Contig3_YK, CL808.Contig5_YK, CL808.Contig6_YK, CL809.Contig1_YK, CL809.Contig2_YK, CL827.Contig1_YK, CL827.Contig2_YK, CL827.Contig3_YK, CL878.Contig1_YK, CL878.Contig2_YK, CL878.Contig3_YK, CL92.Contig1_YK, CL92.Contig2_YK, CL92.Contig3_YK, CL92.Contig4_YK, CL92.Contig5_YK, CL939.Contig1_YK, CL939.Contig2_YK, CL945.Contig1_YK, CL945.Contig2_YK, CL945.Contig3_YK, CL945.Contig4_YK, CL955.Contig1_YK, CL955.Contig2_YK, CL973.Contig1_YK, CL973.Contig2_YK, CL982.Contig1_YK, CL982.Contig2_YK, Unigene10213_YK, Unigene10224_YK, Unigene10272_YK, Unigene10306_YK, Unigene10416_YK, Unigene10457_YK, Unigene10458_YK, Unigene10470_YK, Unigene10644_YK, Unigene10660_YK, Unigene10761_YK, Unigene10762_YK, Unigene10771_YK, Unigene10810_YK, Unigene10854_YK, Unigene10946_YK, Unigene10989_YK, Unigene11097_YK, Unigene11098_YK, Unigene11199_YK, Unigene1123_YK, Unigene11251_YK, Unigene11297_YK, Unigene11372_YK, Unigene11373_YK, Unigene11388_YK, Unigene11439_YK, Unigene11441_YK, Unigene1150_YK, Unigene11561_YK, Unigene11614_YK, Unigene11631_YK, Unigene11665_YK, Unigene11888_YK, Unigene12055_YK, Unigene12171_YK, Unigene121_YK, Unigene12247_YK, Unigene12249_YK, Unigene12264_YK, Unigene12320_YK, Unigene12347_YK, Unigene12433_YK, Unigene12647_YK, Unigene12648_YK, Unigene12651_YK, Unigene12695_YK, Unigene12987_YK, Unigene13024_YK, Unigene13061_YK, Unigene13084_YK, Unigene13094_YK, Unigene13103_YK, Unigene13111_YK, Unigene13192_YK, Unigene13213_YK, Unigene13232_YK, Unigene13274_YK, Unigene13398_YK, Unigene13434_YK, Unigene13451_YK, Unigene13454_YK, Unigene13527_YK, Unigene13550_YK, Unigene13572_YK, Unigene13803_YK, Unigene13850_YK, Unigene13859_YK, Unigene13874_YK, Unigene138_YK, Unigene14011_YK, Unigene14019_YK, Unigene14026_YK, Unigene14303_YK, Unigene14441_YK, Unigene1453_YK, Unigene14551_YK, Unigene14616_YK, Unigene14622_YK, Unigene14815_YK, Unigene1486_YK, Unigene1487_YK, Unigene14885_YK, Unigene15046_YK, Unigene15121_YK, Unigene15130_YK, Unigene15181_YK, Unigene15209_YK, Unigene15258_YK, Unigene15353_YK, Unigene15365_YK, Unigene15382_YK, Unigene15461_YK, Unigene15485_YK, Unigene15516_YK, Unigene15581_YK, Unigene15614_YK, Unigene15707_YK, Unigene15737_YK, Unigene15782_YK, Unigene15850_YK, Unigene15856_YK, Unigene15884_YK, Unigene15898_YK, Unigene15980_YK, Unigene16084_YK, Unigene16089_YK, Unigene16151_YK, Unigene16180_YK, Unigene16233_YK, Unigene16411_YK, Unigene16430_YK, Unigene16446_YK, Unigene16455_YK, Unigene16527_YK, Unigene16631_YK, Unigene1666_YK, Unigene16758_YK, Unigene16793_YK, Unigene16861_YK, Unigene16887_YK, Unigene16925_YK, Unigene16947_YK, Unigene16991_YK, Unigene17177_YK, Unigene17195_YK, Unigene17222_YK, Unigene17508_YK, Unigene17546_YK, Unigene17612_YK, Unigene17753_YK, Unigene17891_YK, Unigene18027_YK, Unigene1812_YK, Unigene18242_YK, Unigene18265_YK, Unigene18746_YK, Unigene18866_YK, Unigene2152_YK, Unigene2196_YK, Unigene220_YK, Unigene2221_YK, Unigene2250_YK, Unigene2295_YK, Unigene2296_YK, Unigene2336_YK, Unigene2407_YK, Unigene2425_YK, Unigene2426_YK, Unigene2457_YK, Unigene2471_YK, Unigene2578_YK, Unigene2593_YK, Unigene2713_YK, Unigene2944_YK, Unigene294_YK, Unigene295_YK, Unigene3062_YK, Unigene3065_YK, Unigene3119_YK, Unigene3145_YK, Unigene3192_YK, Unigene3381_YK, Unigene3391_YK, Unigene3416_YK, Unigene3513_YK, Unigene3517_YK, Unigene3556_YK, Unigene3623_YK, Unigene3756_YK, Unigene382_YK, Unigene3983_YK, Unigene3988_YK, Unigene4037_YK, Unigene4108_YK, Unigene4142_YK, Unigene4314_YK, Unigene4317_YK, Unigene4318_YK, Unigene4532_YK, Unigene4574_YK, Unigene4585_YK, Unigene4628_YK, Unigene4718_YK, Unigene4776_YK, Unigene4796_YK, Unigene4810_YK, Unigene4815_YK, Unigene4859_YK, Unigene5007_YK, Unigene5093_YK, Unigene5102_YK, Unigene5201_YK, Unigene5214_YK, Unigene5224_YK, Unigene5225_YK, Unigene5465_YK, Unigene5466_YK, Unigene5541_YK, Unigene5563_YK, Unigene5637_YK, Unigene5680_YK, Unigene5737_YK, Unigene5750_YK, Unigene5763_YK, Unigene5828_YK, Unigene6003_YK, Unigene6004_YK, Unigene6166_YK, Unigene6243_YK, Unigene625_YK, Unigene6263_YK, Unigene6285_YK, Unigene6286_YK, Unigene6321_YK, Unigene6322_YK, Unigene6450_YK, Unigene6454_YK, Unigene6559_YK, Unigene6560_YK, Unigene6585_YK, Unigene6596_YK, Unigene6738_YK, Unigene6739_YK, Unigene6743_YK, Unigene6752_YK, Unigene6757_YK, Unigene6811_YK, Unigene6863_YK, Unigene6864_YK, Unigene6865_YK, Unigene6882_YK, Unigene6960_YK, Unigene6974_YK, Unigene6975_YK, Unigene7095_YK, Unigene7096_YK, Unigene7100_YK, Unigene7101_YK, Unigene7175_YK, Unigene7176_YK, Unigene7228_YK, Unigene7229_YK, Unigene7281_YK, Unigene7362_YK, Unigene7396_YK, Unigene7397_YK, Unigene7398_YK, Unigene7412_YK, Unigene7592_YK, Unigene7704_YK, Unigene7706_YK, Unigene7945_YK, Unigene8120_YK, Unigene8279_YK, Unigene8344_YK, Unigene8345_YK, Unigene8508_YK, Unigene8573_YK, Unigene8592_YK, Unigene8593_YK, Unigene8657_YK, Unigene8726_YK, Unigene8727_YK, Unigene8987_YK, Unigene9010_YK, Unigene9078_YK, Unigene9110_YK, Unigene91_YK, Unigene9210_YK, Unigene9213_YK, Unigene9225_YK, Unigene9226_YK, Unigene9272_YK, Unigene9273_YK, Unigene9274_YK, Unigene9371_YK, Unigene9377_YK, Unigene9395_YK, Unigene9420_YK, Unigene9429_YK, Unigene957_YK, Unigene9616_YK, Unigene9704_YK, Unigene9894_YK, Unigene9928_YK, Unigene9939_YK, Unigene9940_YK |
| 3 | [Focal adhesion](../../../../D:%5C高通量测序结果%5CF14FTSSCKF1242_NEMpnqE%5Cannotation%5CKEGG%5CYK-Unigene.fa_map%5Cmap04510.html) | CL1026.Contig1_YK, CL1026.Contig2_YK, CL1099.Contig1_YK, CL1119.Contig1_YK, CL1119.Contig2_YK, CL1131.Contig2_YK, CL1131.Contig3_YK, CL1138.Contig2_YK, CL1221.Contig1_YK, CL1221.Contig2_YK, CL1221.Contig3_YK, CL1292.Contig1_YK, CL1292.Contig2_YK, CL1310.Contig6_YK, CL1381.Contig1_YK, CL1381.Contig2_YK, CL1384.Contig1_YK, CL1384.Contig2_YK, CL1406.Contig1_YK, CL1406.Contig2_YK, CL1412.Contig1_YK, CL1488.Contig1_YK, CL1488.Contig2_YK, CL1560.Contig1_YK, CL157.Contig1_YK, CL157.Contig2_YK, CL157.Contig3_YK, CL157.Contig4_YK, CL157.Contig6_YK, CL157.Contig7_YK, CL1577.Contig1_YK, CL1577.Contig2_YK, CL1577.Contig3_YK, CL1577.Contig4_YK, CL1578.Contig1_YK, CL1591.Contig1_YK, CL1602.Contig1_YK, CL1602.Contig2_YK, CL1632.Contig1_YK, CL1632.Contig2_YK, CL1666.Contig1_YK, CL1666.Contig2_YK, CL1670.Contig2_YK, CL1734.Contig1_YK, CL1734.Contig2_YK, CL1734.Contig3_YK, CL1734.Contig4_YK, CL1751.Contig1_YK, CL1751.Contig2_YK, CL1758.Contig1_YK, CL1758.Contig2_YK, CL1765.Contig1_YK, CL1765.Contig2_YK, CL1765.Contig3_YK, CL1816.Contig1_YK, CL1816.Contig2_YK, CL1852.Contig2_YK, CL1852.Contig3_YK, CL1885.Contig1_YK, CL1948.Contig1_YK, CL1948.Contig2_YK, CL1948.Contig3_YK, CL1959.Contig2_YK, CL2002.Contig1_YK, CL2002.Contig2_YK, CL2002.Contig3_YK, CL2002.Contig4_YK, CL2002.Contig5_YK, CL2006.Contig1_YK, CL2006.Contig2_YK, CL2006.Contig3_YK, CL2026.Contig1_YK, CL2026.Contig2_YK, CL2031.Contig1_YK, CL2031.Contig2_YK, CL2103.Contig1_YK, CL2103.Contig2_YK, CL215.Contig1_YK, CL2180.Contig1_YK, CL2180.Contig2_YK, CL2224.Contig2_YK, CL2281.Contig1_YK, CL2281.Contig2_YK, CL2360.Contig1_YK, CL2360.Contig2_YK, CL2381.Contig1_YK, CL2381.Contig2_YK, CL2429.Contig1_YK, CL2429.Contig2_YK, CL2438.Contig1_YK, CL2438.Contig2_YK, CL2449.Contig1_YK, CL2493.Contig1_YK, CL2536.Contig1_YK, CL2562.Contig1_YK, CL2562.Contig2_YK, CL2606.Contig1_YK, CL2606.Contig2_YK, CL2612.Contig1_YK, CL2612.Contig2_YK, CL2668.Contig1_YK, CL2668.Contig2_YK, CL2751.Contig1_YK, CL2751.Contig2_YK, CL2787.Contig2_YK, CL2792.Contig1_YK, CL2793.Contig1_YK, CL2793.Contig2_YK, CL2816.Contig1_YK, CL2816.Contig2_YK, CL2886.Contig1_YK, CL2886.Contig2_YK, CL2887.Contig2_YK, CL2905.Contig1_YK, CL2905.Contig2_YK, CL300.Contig1_YK, CL300.Contig2_YK, CL312.Contig1_YK, CL34.Contig2_YK, CL34.Contig3_YK, CL349.Contig1_YK, CL349.Contig2_YK, CL349.Contig4_YK, CL353.Contig4_YK, CL411.Contig1_YK, CL411.Contig2_YK, CL411.Contig3_YK, CL43.Contig1_YK, CL43.Contig2_YK, CL472.Contig1_YK, CL472.Contig2_YK, CL502.Contig1_YK, CL502.Contig2_YK, CL527.Contig10_YK, CL527.Contig1_YK, CL527.Contig5_YK, CL527.Contig6_YK, CL527.Contig8_YK, CL527.Contig9_YK, CL564.Contig1_YK, CL591.Contig1_YK, CL591.Contig2_YK, CL591.Contig3_YK, CL591.Contig4_YK, CL591.Contig5_YK, CL591.Contig6_YK, CL591.Contig7_YK, CL591.Contig8_YK, CL592.Contig2_YK, CL647.Contig1_YK, CL647.Contig2_YK, CL663.Contig1_YK, CL663.Contig3_YK, CL741.Contig2_YK, CL741.Contig3_YK, CL760.Contig1_YK, CL760.Contig2_YK, CL760.Contig3_YK, CL760.Contig4_YK, CL760.Contig5_YK, CL760.Contig6_YK, CL760.Contig7_YK, CL774.Contig1_YK, CL774.Contig2_YK, CL832.Contig1_YK, CL84.Contig2_YK, CL84.Contig3_YK, CL85.Contig1_YK, CL85.Contig2_YK, CL85.Contig3_YK, CL855.Contig2_YK, CL884.Contig1_YK, CL884.Contig3_YK, CL899.Contig1_YK, CL917.Contig2_YK, CL917.Contig3_YK, CL917.Contig5_YK, CL917.Contig6_YK, CL917.Contig7_YK, CL917.Contig8_YK, CL935.Contig1_YK, CL935.Contig2_YK, Unigene10034_YK, Unigene10197_YK, Unigene10198_YK, Unigene10240_YK, Unigene10252_YK, Unigene10341_YK, Unigene10404_YK, Unigene10439_YK, Unigene10440_YK, Unigene10441_YK, Unigene10444_YK, Unigene10494_YK, Unigene10495_YK, Unigene10527_YK, Unigene10550_YK, Unigene10551_YK, Unigene10653_YK, Unigene10664_YK, Unigene10746_YK, Unigene10751_YK, Unigene10752_YK, Unigene10753_YK, Unigene107_YK, Unigene10868_YK, Unigene10940_YK, Unigene10954_YK, Unigene10972_YK, Unigene10993_YK, Unigene11093_YK, Unigene11140_YK, Unigene11141_YK, Unigene11316_YK, Unigene11317_YK, Unigene11335_YK, Unigene11363_YK, Unigene11364_YK, Unigene11481_YK, Unigene11585_YK, Unigene11662_YK, Unigene11696_YK, Unigene11748_YK, Unigene11837_YK, Unigene11860_YK, Unigene11941_YK, Unigene12017_YK, Unigene12128_YK, Unigene12218_YK, Unigene12352_YK, Unigene12353_YK, Unigene12451_YK, Unigene12535_YK, Unigene12536_YK, Unigene12537_YK, Unigene12538_YK, Unigene12539_YK, Unigene12541_YK, Unigene12542_YK, Unigene12543_YK, Unigene12544_YK, Unigene12548_YK, Unigene12567_YK, Unigene12632_YK, Unigene12733_YK, Unigene12784_YK, Unigene12785_YK, Unigene12898_YK, Unigene12899_YK, Unigene12911_YK, Unigene12977_YK, Unigene12991_YK, Unigene13020_YK, Unigene13108_YK, Unigene13180_YK, Unigene13203_YK, Unigene13293_YK, Unigene13296_YK, Unigene13374_YK, Unigene13456_YK, Unigene13528_YK, Unigene13560_YK, Unigene13571_YK, Unigene13670_YK, Unigene13674_YK, Unigene13720_YK, Unigene13723_YK, Unigene13811_YK, Unigene13827_YK, Unigene13954_YK, Unigene13957_YK, Unigene13977_YK, Unigene13985_YK, Unigene14007_YK, Unigene14093_YK, Unigene14097_YK, Unigene14105_YK, Unigene14254_YK, Unigene14296_YK, Unigene14301_YK, Unigene14345_YK, Unigene14399_YK, Unigene14431_YK, Unigene14647_YK, Unigene14657_YK, Unigene14684_YK, Unigene14710_YK, Unigene14735_YK, Unigene14914_YK, Unigene14929_YK, Unigene15017_YK, Unigene15065_YK, Unigene15166_YK, Unigene15246_YK, Unigene15307_YK, Unigene15325_YK, Unigene15348_YK, Unigene15350_YK, Unigene15413_YK, Unigene15497_YK, Unigene15561_YK, Unigene15673_YK, Unigene15695_YK, Unigene15714_YK, Unigene15802_YK, Unigene15899_YK, Unigene15943_YK, Unigene15974_YK, Unigene15979_YK, Unigene1603_YK, Unigene16057_YK, Unigene16061_YK, Unigene16112_YK, Unigene16250_YK, Unigene16409_YK, Unigene1660_YK, Unigene16613_YK, Unigene16698_YK, Unigene16835_YK, Unigene1719_YK, Unigene17212_YK, Unigene17370_YK, Unigene17547_YK, Unigene17960_YK, Unigene17996_YK, Unigene18305_YK, Unigene18316_YK, Unigene18657_YK, Unigene18931_YK, Unigene18946_YK, Unigene18969_YK, Unigene18999_YK, Unigene19044_YK, Unigene19070_YK, Unigene2018_YK, Unigene201_YK, Unigene2080_YK, Unigene2190_YK, Unigene2230_YK, Unigene2244_YK, Unigene2433_YK, Unigene2486_YK, Unigene256_YK, Unigene2666_YK, Unigene2719_YK, Unigene2768_YK, Unigene2791_YK, Unigene2799_YK, Unigene2849_YK, Unigene2899_YK, Unigene2949_YK, Unigene2991_YK, Unigene3067_YK, Unigene3195_YK, Unigene3198_YK, Unigene3204_YK, Unigene3224_YK, Unigene3286_YK, Unigene3293_YK, Unigene3318_YK, Unigene3349_YK, Unigene337_YK, Unigene3591_YK, Unigene3660_YK, Unigene3718_YK, Unigene3728_YK, Unigene372_YK, Unigene3828_YK, Unigene3965_YK, Unigene3966_YK, Unigene4011_YK, Unigene4052_YK, Unigene4054_YK, Unigene4055_YK, Unigene4060_YK, Unigene4203_YK, Unigene4268_YK, Unigene4396_YK, Unigene4465_YK, Unigene4494_YK, Unigene4576_YK, Unigene4689_YK, Unigene4690_YK, Unigene4761_YK, Unigene4769_YK, Unigene4781_YK, Unigene4809_YK, Unigene4840_YK, Unigene4861_YK, Unigene4924_YK, Unigene4963_YK, Unigene4_YK, Unigene5162_YK, Unigene5191_YK, Unigene5202_YK, Unigene5219_YK, Unigene5385_YK, Unigene5386_YK, Unigene5462_YK, Unigene5467_YK, Unigene5468_YK, Unigene5519_YK, Unigene5603_YK, Unigene5642_YK, Unigene5682_YK, Unigene5699_YK, Unigene5851_YK, Unigene5874_YK, Unigene5890_YK, Unigene5925_YK, Unigene6066_YK, Unigene6076_YK, Unigene6135_YK, Unigene6154_YK, Unigene617_YK, Unigene6182_YK, Unigene6229_YK, Unigene6230_YK, Unigene6231_YK, Unigene6261_YK, Unigene6268_YK, Unigene6331_YK, Unigene6367_YK, Unigene6368_YK, Unigene6407_YK, Unigene6443_YK, Unigene6444_YK, Unigene6591_YK, Unigene6664_YK, Unigene6679_YK, Unigene6685_YK, Unigene6686_YK, Unigene6704_YK, Unigene6889_YK, Unigene6890_YK, Unigene6920_YK, Unigene6981_YK, Unigene7147_YK, Unigene7496_YK, Unigene7525_YK, Unigene7590_YK, Unigene7816_YK, Unigene7959_YK, Unigene7998_YK, Unigene8282_YK, Unigene8323_YK, Unigene8324_YK, Unigene8325_YK, Unigene838_YK, Unigene8396_YK, Unigene8462_YK, Unigene8500_YK, Unigene8501_YK, Unigene8569_YK, Unigene8570_YK, Unigene8718_YK, Unigene8755_YK, Unigene8795_YK, Unigene8877_YK, Unigene8899_YK, Unigene8900_YK, Unigene8908_YK, Unigene9036_YK, Unigene9071_YK, Unigene9102_YK, Unigene9323_YK, Unigene9388_YK, Unigene9417_YK, Unigene9584_YK, Unigene9678_YK, Unigene9968_YK, Unigene9969_YK |
| 4 | [Pathways in cancer](../../../../D:%5C高通量测序结果%5CF14FTSSCKF1242_NEMpnqE%5Cannotation%5CKEGG%5CYK-Unigene.fa_map%5Cmap05200.html) | CL1003.Contig1_YK, CL1003.Contig2_YK, CL1029.Contig1_YK, CL1072.Contig1_YK, CL1072.Contig2_YK, CL1076.Contig1_YK, CL1076.Contig2_YK, CL1119.Contig1_YK, CL1119.Contig2_YK, CL117.Contig2_YK, CL1170.Contig1_YK, CL1170.Contig2_YK, CL1170.Contig3_YK, CL1170.Contig4_YK, CL1184.Contig2_YK, CL1206.Contig2_YK, CL1207.Contig1_YK, CL1207.Contig2_YK, CL1207.Contig3_YK, CL1207.Contig4_YK, CL1207.Contig5_YK, CL1223.Contig1_YK, CL1223.Contig3_YK, CL1278.Contig1_YK, CL1278.Contig2_YK, CL1292.Contig1_YK, CL1292.Contig2_YK, CL1310.Contig6_YK, CL1366.Contig1_YK, CL1366.Contig2_YK, CL1381.Contig1_YK, CL1381.Contig2_YK, CL1390.Contig1_YK, CL1390.Contig2_YK, CL1406.Contig2_YK, CL1457.Contig1_YK, CL1457.Contig2_YK, CL1471.Contig1_YK, CL1505.Contig1_YK, CL1505.Contig2_YK, CL1516.Contig1_YK, CL1516.Contig2_YK, CL1538.Contig1_YK, CL1538.Contig2_YK, CL1544.Contig1_YK, CL1544.Contig2_YK, CL157.Contig1_YK, CL157.Contig2_YK, CL157.Contig3_YK, CL157.Contig4_YK, CL157.Contig6_YK, CL157.Contig7_YK, CL1578.Contig1_YK, CL1578.Contig2_YK, CL1585.Contig1_YK, CL1585.Contig2_YK, CL1591.Contig1_YK, CL1602.Contig1_YK, CL1602.Contig2_YK, CL1651.Contig1_YK, CL1651.Contig2_YK, CL1666.Contig1_YK, CL1666.Contig2_YK, CL1679.Contig1_YK, CL1679.Contig2_YK, CL1679.Contig3_YK, CL1740.Contig1_YK, CL1758.Contig1_YK, CL1758.Contig2_YK, CL1765.Contig1_YK, CL1765.Contig2_YK, CL1765.Contig3_YK, CL1792.Contig1_YK, CL1792.Contig2_YK, CL1816.Contig1_YK, CL1816.Contig2_YK, CL1885.Contig1_YK, CL2006.Contig1_YK, CL2006.Contig2_YK, CL2006.Contig3_YK, CL2042.Contig1_YK, CL2147.Contig1_YK, CL2147.Contig2_YK, CL2159.Contig1_YK, CL2159.Contig2_YK, CL2199.Contig1_YK, CL2199.Contig2_YK, CL2199.Contig3_YK, CL2224.Contig2_YK, CL2294.Contig1_YK, CL2294.Contig2_YK, CL2336.Contig1_YK, CL2336.Contig2_YK, CL2342.Contig1_YK, CL2342.Contig2_YK, CL2378.Contig1_YK, CL2378.Contig2_YK, CL2429.Contig1_YK, CL2429.Contig2_YK, CL2438.Contig1_YK, CL2438.Contig2_YK, CL2449.Contig1_YK, CL2476.Contig1_YK, CL2493.Contig1_YK, CL2625.Contig1_YK, CL2648.Contig2_YK, CL2666.Contig1_YK, CL2666.Contig2_YK, CL2668.Contig1_YK, CL2668.Contig2_YK, CL2729.Contig1_YK, CL2751.Contig1_YK, CL2751.Contig2_YK, CL2791.Contig1_YK, CL2791.Contig2_YK, CL2792.Contig1_YK, CL2793.Contig1_YK, CL2793.Contig2_YK, CL2800.Contig1_YK, CL2800.Contig2_YK, CL2831.Contig1_YK, CL2831.Contig2_YK, CL2866.Contig1_YK, CL2886.Contig1_YK, CL367.Contig1_YK, CL37.Contig1_YK, CL374.Contig1_YK, CL383.Contig10_YK, CL383.Contig11_YK, CL383.Contig12_YK, CL383.Contig19_YK, CL383.Contig1_YK, CL383.Contig20_YK, CL383.Contig21_YK, CL383.Contig22_YK, CL383.Contig2_YK, CL383.Contig3_YK, CL383.Contig9_YK, CL387.Contig1_YK, CL387.Contig2_YK, CL387.Contig3_YK, CL387.Contig4_YK, CL387.Contig5_YK, CL425.Contig1_YK, CL425.Contig2_YK, CL438.Contig2_YK, CL438.Contig3_YK, CL475.Contig1_YK, CL475.Contig2_YK, CL475.Contig3_YK, CL475.Contig4_YK, CL502.Contig1_YK, CL502.Contig2_YK, CL552.Contig1_YK, CL577.Contig1_YK, CL577.Contig2_YK, CL577.Contig3_YK, CL577.Contig4_YK, CL577.Contig5_YK, CL577.Contig6_YK, CL591.Contig1_YK, CL591.Contig2_YK, CL591.Contig3_YK, CL591.Contig4_YK, CL591.Contig5_YK, CL591.Contig6_YK, CL591.Contig7_YK, CL591.Contig8_YK, CL592.Contig2_YK, CL599.Contig1_YK, CL599.Contig2_YK, CL627.Contig1_YK, CL627.Contig2_YK, CL647.Contig1_YK, CL647.Contig2_YK, CL671.Contig1_YK, CL671.Contig2_YK, CL720.Contig1_YK, CL720.Contig2_YK, CL727.Contig1_YK, CL730.Contig1_YK, CL730.Contig2_YK, CL741.Contig2_YK, CL741.Contig3_YK, CL760.Contig3_YK, CL760.Contig5_YK, CL760.Contig6_YK, CL760.Contig7_YK, CL774.Contig1_YK, CL774.Contig2_YK, CL775.Contig1_YK, CL775.Contig2_YK, CL814.Contig1_YK, CL814.Contig2_YK, CL83.Contig4_YK, CL832.Contig1_YK, CL837.Contig1_YK, CL837.Contig2_YK, CL856.Contig2_YK, CL884.Contig1_YK, CL884.Contig3_YK, CL917.Contig3_YK, CL917.Contig7_YK, CL961.Contig7_YK, CL979.Contig1_YK, CL979.Contig2_YK, CL979.Contig3_YK, Unigene10072_YK, Unigene1009_YK, Unigene10197_YK, Unigene10198_YK, Unigene10207_YK, Unigene10251_YK, Unigene10271_YK, Unigene10335_YK, Unigene10404_YK, Unigene10439_YK, Unigene10440_YK, Unigene10441_YK, Unigene10494_YK, Unigene10495_YK, Unigene10659_YK, Unigene107_YK, Unigene10843_YK, Unigene10844_YK, Unigene10857_YK, Unigene10954_YK, Unigene10961_YK, Unigene10972_YK, Unigene11008_YK, Unigene11024_YK, Unigene11075_YK, Unigene11093_YK, Unigene11299_YK, Unigene11317_YK, Unigene11335_YK, Unigene11478_YK, Unigene11529_YK, Unigene11570_YK, Unigene11585_YK, Unigene11802_YK, Unigene11860_YK, Unigene11931_YK, Unigene12218_YK, Unigene12239_YK, Unigene12240_YK, Unigene12352_YK, Unigene12353_YK, Unigene12451_YK, Unigene12537_YK, Unigene12539_YK, Unigene12543_YK, Unigene12621_YK, Unigene12743_YK, Unigene13013_YK, Unigene1304_YK, Unigene13206_YK, Unigene13230_YK, Unigene13296_YK, Unigene1331_YK, Unigene13355_YK, Unigene13407_YK, Unigene13436_YK, Unigene13457_YK, Unigene13531_YK, Unigene13599_YK, Unigene13670_YK, Unigene13674_YK, Unigene13713_YK, Unigene13716_YK, Unigene13723_YK, Unigene13747_YK, Unigene13843_YK, Unigene13886_YK, Unigene13893_YK, Unigene13896_YK, Unigene13978_YK, Unigene13985_YK, Unigene14007_YK, Unigene14012_YK, Unigene14097_YK, Unigene14150_YK, Unigene14345_YK, Unigene14382_YK, Unigene14393_YK, Unigene14516_YK, Unigene14550_YK, Unigene14552_YK, Unigene14582_YK, Unigene14625_YK, Unigene14681_YK, Unigene14700_YK, Unigene14710_YK, Unigene14721_YK, Unigene14722_YK, Unigene14805_YK, Unigene14867_YK, Unigene14942_YK, Unigene15034_YK, Unigene15146_YK, Unigene15285_YK, Unigene15325_YK, Unigene15350_YK, Unigene15497_YK, Unigene15512_YK, Unigene15561_YK, Unigene15614_YK, Unigene15698_YK, Unigene15780_YK, Unigene15856_YK, Unigene15891_YK, Unigene15910_YK, Unigene15943_YK, Unigene16061_YK, Unigene16152_YK, Unigene16247_YK, Unigene16250_YK, Unigene16530_YK, Unigene16615_YK, Unigene16698_YK, Unigene16821_YK, Unigene17062_YK, Unigene17113_YK, Unigene17185_YK, Unigene17339_YK, Unigene17790_YK, Unigene17893_YK, Unigene1790_YK, Unigene18210_YK, Unigene18217_YK, Unigene18319_YK, Unigene18590_YK, Unigene18611_YK, Unigene18630_YK, Unigene18657_YK, Unigene18679_YK, Unigene18931_YK, Unigene18955_YK, Unigene18999_YK, Unigene19071_YK, Unigene2033_YK, Unigene2169_YK, Unigene2230_YK, Unigene2398_YK, Unigene2433_YK, Unigene2486_YK, Unigene24_YK, Unigene2652_YK, Unigene2658_YK, Unigene26_YK, Unigene2791_YK, Unigene2799_YK, Unigene2835_YK, Unigene2961_YK, Unigene3032_YK, Unigene3109_YK, Unigene3224_YK, Unigene3245_YK, Unigene3318_YK, Unigene3321_YK, Unigene3349_YK, Unigene3350_YK, Unigene3374_YK, Unigene3384_YK, Unigene3483_YK, Unigene3559_YK, Unigene3591_YK, Unigene3667_YK, Unigene3718_YK, Unigene372_YK, Unigene3764_YK, Unigene4038_YK, Unigene4055_YK, Unigene4128_YK, Unigene4166_YK, Unigene4203_YK, Unigene4268_YK, Unigene4286_YK, Unigene4308_YK, Unigene4428_YK, Unigene4494_YK, Unigene4680_YK, Unigene4781_YK, Unigene4842_YK, Unigene485_YK, Unigene486_YK, Unigene4872_YK, Unigene4924_YK, Unigene4963_YK, Unigene4_YK, Unigene5101_YK, Unigene5219_YK, Unigene5226_YK, Unigene5462_YK, Unigene5502_YK, Unigene5514_YK, Unigene5605_YK, Unigene5642_YK, Unigene5656_YK, Unigene5828_YK, Unigene5941_YK, Unigene5993_YK, Unigene6021_YK, Unigene6027_YK, Unigene6176_YK, Unigene6229_YK, Unigene6230_YK, Unigene6231_YK, Unigene6304_YK, Unigene6380_YK, Unigene6406_YK, Unigene6444_YK, Unigene6561_YK, Unigene6615_YK, Unigene6617_YK, Unigene6664_YK, Unigene6685_YK, Unigene6708_YK, Unigene6814_YK, Unigene6889_YK, Unigene6890_YK, Unigene6963_YK, Unigene7324_YK, Unigene7448_YK, Unigene7496_YK, Unigene7500_YK, Unigene7525_YK, Unigene7590_YK, Unigene7745_YK, Unigene7755_YK, Unigene7816_YK, Unigene7846_YK, Unigene7904_YK, Unigene7998_YK, Unigene8059_YK, Unigene8210_YK, Unigene8259_YK, Unigene8282_YK, Unigene830_YK, Unigene8323_YK, Unigene8324_YK, Unigene8325_YK, Unigene8361_YK, Unigene8378_YK, Unigene8496_YK, Unigene8647_YK, Unigene8718_YK, Unigene8762_YK, Unigene9071_YK, Unigene9098_YK, Unigene9127_YK, Unigene9188_YK, Unigene9190_YK, Unigene9323_YK, Unigene9388_YK, Unigene9423_YK, Unigene9480_YK, Unigene9642_YK, Unigene9649_YK, Unigene9678_YK, Unigene9706_YK, Unigene9732_YK, Unigene97_YK, Unigene9944_YK, Unigene994_YK, Unigene995_YK, Unigene9968_YK, Unigene9969_YK |
| 5 | [Regulation of actin cytoskeleton](../../../../D:%5C高通量测序结果%5CF14FTSSCKF1242_NEMpnqE%5Cannotation%5CKEGG%5CYK-Unigene.fa_map%5Cmap04810.html) | CL1003.Contig1_YK, CL1003.Contig2_YK, CL1019.Contig1_YK, CL1019.Contig2_YK, CL1019.Contig3_YK, CL1019.Contig4_YK, CL1026.Contig1_YK, CL1026.Contig2_YK, CL1131.Contig2_YK, CL1131.Contig3_YK, CL1138.Contig2_YK, CL1156.Contig3_YK, CL1185.Contig1_YK, CL1185.Contig2_YK, CL1221.Contig1_YK, CL1221.Contig2_YK, CL1221.Contig3_YK, CL124.Contig1_YK, CL124.Contig2_YK, CL124.Contig3_YK, CL1292.Contig1_YK, CL1292.Contig2_YK, CL1303.Contig1_YK, CL1303.Contig2_YK, CL1303.Contig3_YK, CL1381.Contig1_YK, CL1381.Contig2_YK, CL1412.Contig1_YK, CL1439.Contig1_YK, CL1439.Contig2_YK, CL1486.Contig2_YK, CL1488.Contig1_YK, CL1488.Contig2_YK, CL1577.Contig1_YK, CL1577.Contig2_YK, CL1577.Contig3_YK, CL1577.Contig4_YK, CL1578.Contig1_YK, CL1578.Contig2_YK, CL1591.Contig1_YK, CL1602.Contig1_YK, CL1602.Contig2_YK, CL1632.Contig1_YK, CL1632.Contig2_YK, CL1666.Contig1_YK, CL1666.Contig2_YK, CL1761.Contig1_YK, CL181.Contig1_YK, CL181.Contig2_YK, CL1814.Contig1_YK, CL1814.Contig2_YK, CL1816.Contig1_YK, CL1816.Contig2_YK, CL1831.Contig3_YK, CL1852.Contig2_YK, CL1852.Contig3_YK, CL1920.Contig1_YK, CL1920.Contig2_YK, CL1948.Contig1_YK, CL1948.Contig2_YK, CL1948.Contig3_YK, CL1990.Contig1_YK, CL1990.Contig2_YK, CL2002.Contig1_YK, CL2002.Contig2_YK, CL2002.Contig3_YK, CL2002.Contig4_YK, CL2002.Contig5_YK, CL2006.Contig1_YK, CL2006.Contig2_YK, CL2006.Contig3_YK, CL2103.Contig1_YK, CL2103.Contig2_YK, CL215.Contig1_YK, CL2180.Contig1_YK, CL2180.Contig2_YK, CL2224.Contig2_YK, CL2253.Contig1_YK, CL2253.Contig2_YK, CL2278.Contig1_YK, CL2278.Contig2_YK, CL2320.Contig1_YK, CL2360.Contig1_YK, CL2360.Contig2_YK, CL2381.Contig1_YK, CL2381.Contig2_YK, CL2429.Contig1_YK, CL2429.Contig2_YK, CL2449.Contig1_YK, CL2527.Contig1_YK, CL2527.Contig3_YK, CL2560.Contig1_YK, CL2560.Contig2_YK, CL2606.Contig1_YK, CL2606.Contig2_YK, CL2612.Contig1_YK, CL2612.Contig2_YK, CL2648.Contig1_YK, CL2669.Contig1_YK, CL2669.Contig2_YK, CL2669.Contig3_YK, CL2669.Contig4_YK, CL2669.Contig5_YK, CL2717.Contig2_YK, CL2774.Contig2_YK, CL2787.Contig2_YK, CL2792.Contig1_YK, CL2816.Contig1_YK, CL2816.Contig2_YK, CL2887.Contig2_YK, CL2896.Contig1_YK, CL2905.Contig1_YK, CL2905.Contig2_YK, CL300.Contig1_YK, CL300.Contig2_YK, CL312.Contig1_YK, CL324.Contig1_YK, CL324.Contig2_YK, CL324.Contig3_YK, CL324.Contig4_YK, CL324.Contig5_YK, CL34.Contig2_YK, CL34.Contig3_YK, CL344.Contig3_YK, CL349.Contig1_YK, CL349.Contig2_YK, CL349.Contig4_YK, CL353.Contig4_YK, CL369.Contig2_YK, CL369.Contig5_YK, CL369.Contig8_YK, CL37.Contig2_YK, CL413.Contig1_YK, CL413.Contig2_YK, CL437.Contig1_YK, CL437.Contig2_YK, CL487.Contig1_YK, CL487.Contig2_YK, CL527.Contig10_YK, CL527.Contig1_YK, CL527.Contig5_YK, CL527.Contig6_YK, CL527.Contig8_YK, CL527.Contig9_YK, CL592.Contig2_YK, CL599.Contig1_YK, CL599.Contig2_YK, CL663.Contig1_YK, CL663.Contig3_YK, CL674.Contig10_YK, CL674.Contig3_YK, CL832.Contig1_YK, CL84.Contig2_YK, CL84.Contig3_YK, CL85.Contig1_YK, CL85.Contig2_YK, CL85.Contig3_YK, CL899.Contig1_YK, CL91.Contig1_YK, CL91.Contig2_YK, CL91.Contig3_YK, CL91.Contig4_YK, CL91.Contig5_YK, CL91.Contig6_YK, CL91.Contig7_YK, CL935.Contig1_YK, CL935.Contig2_YK, CL95.Contig2_YK, CL978.Contig1_YK, CL978.Contig2_YK, Unigene10038_YK, Unigene10039_YK, Unigene10251_YK, Unigene10291_YK, Unigene10355_YK, Unigene10527_YK, Unigene1057_YK, Unigene10695_YK, Unigene107_YK, Unigene10868_YK, Unigene10927_YK, Unigene10972_YK, Unigene11078_YK, Unigene11093_YK, Unigene11140_YK, Unigene11141_YK, Unigene11147_YK, Unigene11274_YK, Unigene11481_YK, Unigene11532_YK, Unigene11662_YK, Unigene11711_YK, Unigene11837_YK, Unigene11884_YK, Unigene12027_YK, Unigene12128_YK, Unigene12334_YK, Unigene12335_YK, Unigene12336_YK, Unigene12337_YK, Unigene12426_YK, Unigene12451_YK, Unigene12491_YK, Unigene12492_YK, Unigene12493_YK, Unigene12494_YK, Unigene12532_YK, Unigene12567_YK, Unigene12632_YK, Unigene12733_YK, Unigene12784_YK, Unigene12785_YK, Unigene12898_YK, Unigene12899_YK, Unigene13020_YK, Unigene13180_YK, Unigene13203_YK, Unigene13245_YK, Unigene13296_YK, Unigene1332_YK, Unigene13383_YK, Unigene13436_YK, Unigene13528_YK, Unigene13560_YK, Unigene13570_YK, Unigene13589_YK, Unigene13598_YK, Unigene13605_YK, Unigene13658_YK, Unigene13670_YK, Unigene13674_YK, Unigene13704_YK, Unigene13720_YK, Unigene13723_YK, Unigene13811_YK, Unigene13827_YK, Unigene13843_YK, Unigene13890_YK, Unigene13920_YK, Unigene13943_YK, Unigene13957_YK, Unigene13977_YK, Unigene13985_YK, Unigene13989_YK, Unigene13990_YK, Unigene14005_YK, Unigene14016_YK, Unigene14035_YK, Unigene14067_YK, Unigene14097_YK, Unigene14105_YK, Unigene14116_YK, Unigene14135_YK, Unigene14143_YK, Unigene14159_YK, Unigene14254_YK, Unigene14327_YK, Unigene14345_YK, Unigene14403_YK, Unigene14420_YK, Unigene14487_YK, Unigene14507_YK, Unigene14600_YK, Unigene14661_YK, Unigene14684_YK, Unigene14713_YK, Unigene14729_YK, Unigene14747_YK, Unigene14856_YK, Unigene14929_YK, Unigene14966_YK, Unigene15034_YK, Unigene15166_YK, Unigene15190_YK, Unigene15246_YK, Unigene15271_YK, Unigene15307_YK, Unigene15308_YK, Unigene15315_YK, Unigene15348_YK, Unigene15497_YK, Unigene15695_YK, Unigene15698_YK, Unigene15713_YK, Unigene15714_YK, Unigene15795_YK, Unigene15899_YK, Unigene15910_YK, Unigene15961_YK, Unigene15974_YK, Unigene15979_YK, Unigene16018_YK, Unigene16057_YK, Unigene16061_YK, Unigene16105_YK, Unigene16112_YK, Unigene16217_YK, Unigene16250_YK, Unigene16409_YK, Unigene16458_YK, Unigene16741_YK, Unigene16748_YK, Unigene16843_YK, Unigene16862_YK, Unigene16869_YK, Unigene16894_YK, Unigene16898_YK, Unigene1689_YK, Unigene17370_YK, Unigene17458_YK, Unigene179_YK, Unigene182_YK, Unigene18316_YK, Unigene18657_YK, Unigene18676_YK, Unigene18798_YK, Unigene18999_YK, Unigene19044_YK, Unigene201_YK, Unigene2080_YK, Unigene2207_YK, Unigene2244_YK, Unigene256_YK, Unigene2620_YK, Unigene2719_YK, Unigene2791_YK, Unigene2799_YK, Unigene2849_YK, Unigene2892_YK, Unigene2899_YK, Unigene2949_YK, Unigene3026_YK, Unigene3034_YK, Unigene3067_YK, Unigene3188_YK, Unigene3189_YK, Unigene3256_YK, Unigene3268_YK, Unigene3271_YK, Unigene3286_YK, Unigene3293_YK, Unigene3336_YK, Unigene3349_YK, Unigene337_YK, Unigene352_YK, Unigene3629_YK, Unigene3667_YK, Unigene372_YK, Unigene3739_YK, Unigene3740_YK, Unigene3745_YK, Unigene3850_YK, Unigene3906_YK, Unigene3916_YK, Unigene4011_YK, Unigene4052_YK, Unigene4074_YK, Unigene4203_YK, Unigene4268_YK, Unigene4294_YK, Unigene4308_YK, Unigene4315_YK, Unigene4396_YK, Unigene4494_YK, Unigene4583_YK, Unigene4680_YK, Unigene4689_YK, Unigene4690_YK, Unigene4747_YK, Unigene4761_YK, Unigene4809_YK, Unigene4825_YK, Unigene4840_YK, Unigene4861_YK, Unigene4912_YK, Unigene4924_YK, Unigene4939_YK, Unigene5052_YK, Unigene5162_YK, Unigene5191_YK, Unigene5219_YK, Unigene5352_YK, Unigene5353_YK, Unigene5385_YK, Unigene5386_YK, Unigene5467_YK, Unigene5468_YK, Unigene5603_YK, Unigene5682_YK, Unigene5819_YK, Unigene5874_YK, Unigene5941_YK, Unigene6066_YK, Unigene6076_YK, Unigene6077_YK, Unigene6087_YK, Unigene6105_YK, Unigene6135_YK, Unigene6146_YK, Unigene6154_YK, Unigene6182_YK, Unigene6250_YK, Unigene6309_YK, Unigene6310_YK, Unigene6331_YK, Unigene6367_YK, Unigene6368_YK, Unigene6407_YK, Unigene6444_YK, Unigene6469_YK, Unigene6514_YK, Unigene6553_YK, Unigene6561_YK, Unigene6591_YK, Unigene6704_YK, Unigene6814_YK, Unigene6850_YK, Unigene6889_YK, Unigene6890_YK, Unigene6920_YK, Unigene6930_YK, Unigene6931_YK, Unigene7324_YK, Unigene7330_YK, Unigene7590_YK, Unigene7662_YK, Unigene7684_YK, Unigene7701_YK, Unigene801_YK, Unigene8323_YK, Unigene8324_YK, Unigene8325_YK, Unigene836_YK, Unigene838_YK, Unigene8396_YK, Unigene8500_YK, Unigene8501_YK, Unigene8569_YK, Unigene8570_YK, Unigene8715_YK, Unigene8755_YK, Unigene8877_YK, Unigene8908_YK, Unigene8997_YK, Unigene9057_YK, Unigene9323_YK, Unigene9373_YK, Unigene9388_YK, Unigene9415_YK, Unigene9417_YK, Unigene9480_YK, Unigene9594_YK, Unigene9649_YK, Unigene9732_YK, Unigene9937_YK, Unigene9989_YK |
| 6 | [Vascular smooth muscle contraction](../../../../D:%5C高通量测序结果%5CF14FTSSCKF1242_NEMpnqE%5Cannotation%5CKEGG%5CYK-Unigene.fa_map%5Cmap04270.html) | CL1019.Contig1_YK, CL1019.Contig2_YK, CL1019.Contig3_YK, CL1019.Contig4_YK, CL1026.Contig1_YK, CL1026.Contig2_YK, CL1041.Contig1_YK, CL1041.Contig2_YK, CL1088.Contig1_YK, CL1088.Contig2_YK, CL1131.Contig2_YK, CL1131.Contig3_YK, CL1138.Contig2_YK, CL1156.Contig3_YK, CL124.Contig1_YK, CL124.Contig2_YK, CL124.Contig3_YK, CL1303.Contig1_YK, CL1303.Contig2_YK, CL1303.Contig3_YK, CL1412.Contig1_YK, CL1469.Contig1_YK, CL1486.Contig2_YK, CL15.Contig10_YK, CL15.Contig11_YK, CL15.Contig12_YK, CL15.Contig13_YK, CL15.Contig14_YK, CL15.Contig15_YK, CL15.Contig16_YK, CL15.Contig17_YK, CL15.Contig18_YK, CL15.Contig19_YK, CL15.Contig1_YK, CL15.Contig20_YK, CL15.Contig21_YK, CL15.Contig22_YK, CL15.Contig23_YK, CL15.Contig24_YK, CL15.Contig2_YK, CL15.Contig3_YK, CL15.Contig4_YK, CL15.Contig5_YK, CL15.Contig6_YK, CL15.Contig7_YK, CL15.Contig8_YK, CL15.Contig9_YK, CL1553.Contig1_YK, CL1553.Contig2_YK, CL1553.Contig3_YK, CL1577.Contig1_YK, CL1577.Contig2_YK, CL1577.Contig3_YK, CL1577.Contig4_YK, CL1578.Contig1_YK, CL1578.Contig2_YK, CL1591.Contig1_YK, CL1602.Contig1_YK, CL1602.Contig2_YK, CL1632.Contig1_YK, CL1632.Contig2_YK, CL1714.Contig2_YK, CL181.Contig1_YK, CL181.Contig2_YK, CL1816.Contig1_YK, CL1816.Contig2_YK, CL1831.Contig3_YK, CL1852.Contig2_YK, CL1852.Contig3_YK, CL1861.Contig1_YK, CL1920.Contig1_YK, CL1920.Contig2_YK, CL1948.Contig1_YK, CL1948.Contig2_YK, CL1948.Contig3_YK, CL1969.Contig1_YK, CL1969.Contig2_YK, CL2006.Contig1_YK, CL2006.Contig2_YK, CL2006.Contig3_YK, CL2103.Contig1_YK, CL2103.Contig2_YK, CL2224.Contig2_YK, CL2361.Contig1_YK, CL2381.Contig1_YK, CL2381.Contig2_YK, CL2428.Contig1_YK, CL2428.Contig2_YK, CL255.Contig1_YK, CL255.Contig2_YK, CL255.Contig3_YK, CL255.Contig4_YK, CL255.Contig5_YK, CL255.Contig6_YK, CL255.Contig7_YK, CL255.Contig8_YK, CL2560.Contig1_YK, CL2560.Contig2_YK, CL2593.Contig1_YK, CL2593.Contig2_YK, CL2593.Contig3_YK, CL2648.Contig1_YK, CL2669.Contig1_YK, CL2669.Contig2_YK, CL2669.Contig3_YK, CL2669.Contig4_YK, CL2669.Contig5_YK, CL2774.Contig2_YK, CL2787.Contig2_YK, CL2792.Contig1_YK, CL2816.Contig2_YK, CL2887.Contig2_YK, CL2905.Contig2_YK, CL295.Contig10_YK, CL295.Contig2_YK, CL295.Contig3_YK, CL295.Contig4_YK, CL295.Contig6_YK, CL295.Contig8_YK, CL295.Contig9_YK, CL304.Contig5_YK, CL307.Contig10_YK, CL307.Contig11_YK, CL307.Contig12_YK, CL307.Contig1_YK, CL307.Contig2_YK, CL307.Contig3_YK, CL307.Contig4_YK, CL307.Contig5_YK, CL307.Contig6_YK, CL307.Contig7_YK, CL307.Contig8_YK, CL307.Contig9_YK, CL312.Contig1_YK, CL34.Contig2_YK, CL34.Contig3_YK, CL341.Contig1_YK, CL341.Contig2_YK, CL344.Contig3_YK, CL353.Contig4_YK, CL369.Contig2_YK, CL369.Contig5_YK, CL369.Contig8_YK, CL37.Contig2_YK, CL487.Contig1_YK, CL487.Contig2_YK, CL527.Contig10_YK, CL527.Contig1_YK, CL527.Contig5_YK, CL527.Contig6_YK, CL527.Contig8_YK, CL527.Contig9_YK, CL568.Contig1_YK, CL568.Contig2_YK, CL591.Contig1_YK, CL591.Contig2_YK, CL591.Contig3_YK, CL591.Contig4_YK, CL591.Contig5_YK, CL591.Contig6_YK, CL591.Contig7_YK, CL591.Contig8_YK, CL599.Contig1_YK, CL599.Contig2_YK, CL663.Contig1_YK, CL663.Contig3_YK, CL79.Contig1_YK, CL79.Contig2_YK, CL79.Contig3_YK, CL79.Contig4_YK, CL79.Contig5_YK, CL79.Contig6_YK, CL79.Contig7_YK, CL79.Contig8_YK, CL838.Contig1_YK, CL838.Contig2_YK, CL838.Contig3_YK, CL838.Contig4_YK, CL838.Contig5_YK, CL838.Contig8_YK, CL85.Contig1_YK, CL85.Contig2_YK, CL85.Contig3_YK, CL884.Contig1_YK, CL884.Contig3_YK, CL899.Contig1_YK, CL902.Contig1_YK, CL902.Contig2_YK, CL91.Contig1_YK, CL91.Contig2_YK, CL91.Contig3_YK, CL91.Contig4_YK, CL91.Contig5_YK, CL91.Contig6_YK, CL91.Contig7_YK, CL935.Contig1_YK, CL935.Contig2_YK, CL95.Contig2_YK, Unigene10194_YK, Unigene10251_YK, Unigene10291_YK, Unigene10355_YK, Unigene10527_YK, Unigene10695_YK, Unigene107_YK, Unigene10908_YK, Unigene10927_YK, Unigene11140_YK, Unigene11141_YK, Unigene11167_YK, Unigene11173_YK, Unigene11174_YK, Unigene11254_YK, Unigene11481_YK, Unigene11496_YK, Unigene11532_YK, Unigene11662_YK, Unigene11837_YK, Unigene11884_YK, Unigene11889_YK, Unigene12027_YK, Unigene12128_YK, Unigene12177_YK, Unigene12178_YK, Unigene12179_YK, Unigene12180_YK, Unigene12216_YK, Unigene12219_YK, Unigene1223_YK, Unigene12292_YK, Unigene12293_YK, Unigene12334_YK, Unigene12335_YK, Unigene12336_YK, Unigene12337_YK, Unigene12426_YK, Unigene12451_YK, Unigene12491_YK, Unigene12492_YK, Unigene12493_YK, Unigene12494_YK, Unigene12532_YK, Unigene12560_YK, Unigene12561_YK, Unigene12563_YK, Unigene12564_YK, Unigene12567_YK, Unigene12631_YK, Unigene12632_YK, Unigene12636_YK, Unigene12638_YK, Unigene12639_YK, Unigene12733_YK, Unigene12785_YK, Unigene13020_YK, Unigene13180_YK, Unigene13237_YK, Unigene13245_YK, Unigene13489_YK, Unigene13528_YK, Unigene13560_YK, Unigene13598_YK, Unigene13674_YK, Unigene13704_YK, Unigene13797_YK, Unigene13827_YK, Unigene13890_YK, Unigene13920_YK, Unigene13937_YK, Unigene13957_YK, Unigene13960_YK, Unigene13985_YK, Unigene14005_YK, Unigene14016_YK, Unigene14047_YK, Unigene14167_YK, Unigene14220_YK, Unigene14243_YK, Unigene14254_YK, Unigene14327_YK, Unigene14345_YK, Unigene14403_YK, Unigene14420_YK, Unigene14661_YK, Unigene14684_YK, Unigene14729_YK, Unigene1488_YK, Unigene14966_YK, Unigene15034_YK, Unigene15057_YK, Unigene15246_YK, Unigene15247_YK, Unigene15271_YK, Unigene15308_YK, Unigene15315_YK, Unigene15348_YK, Unigene15587_YK, Unigene15697_YK, Unigene158_YK, Unigene15961_YK, Unigene15974_YK, Unigene15979_YK, Unigene16000_YK, Unigene16018_YK, Unigene16057_YK, Unigene16102_YK, Unigene16105_YK, Unigene16110_YK, Unigene16112_YK, Unigene16406_YK, Unigene16458_YK, Unigene16741_YK, Unigene16862_YK, Unigene16869_YK, Unigene17035_YK, Unigene17458_YK, Unigene17602_YK, Unigene17932_YK, Unigene182_YK, Unigene18798_YK, Unigene18944_YK, Unigene1895_YK, Unigene1964_YK, Unigene201_YK, Unigene2080_YK, Unigene2143_YK, Unigene2207_YK, Unigene2231_YK, Unigene2289_YK, Unigene254_YK, Unigene2550_YK, Unigene258_YK, Unigene2620_YK, Unigene2657_YK, Unigene2719_YK, Unigene2892_YK, Unigene2899_YK, Unigene3026_YK, Unigene3034_YK, Unigene3188_YK, Unigene3189_YK, Unigene3268_YK, Unigene3271_YK, Unigene331_YK, Unigene337_YK, Unigene3626_YK, Unigene3629_YK, Unigene3739_YK, Unigene3740_YK, Unigene3906_YK, Unigene3916_YK, Unigene4011_YK, Unigene4052_YK, Unigene4074_YK, Unigene4190_YK, Unigene4252_YK, Unigene4315_YK, Unigene4396_YK, Unigene4416_YK, Unigene4494_YK, Unigene4496_YK, Unigene4497_YK, Unigene4505_YK, Unigene4689_YK, Unigene4690_YK, Unigene4747_YK, Unigene4809_YK, Unigene4825_YK, Unigene4827_YK, Unigene4840_YK, Unigene5162_YK, Unigene5191_YK, Unigene5219_YK, Unigene5385_YK, Unigene5386_YK, Unigene5467_YK, Unigene5468_YK, Unigene5476_YK, Unigene5477_YK, Unigene5577_YK, Unigene561_YK, Unigene5682_YK, Unigene5819_YK, Unigene5857_YK, Unigene5874_YK, Unigene5924_YK, Unigene6135_YK, Unigene6146_YK, Unigene6154_YK, Unigene6206_YK, Unigene6227_YK, Unigene6331_YK, Unigene6367_YK, Unigene6368_YK, Unigene6461_YK, Unigene6469_YK, Unigene6582_YK, Unigene6591_YK, Unigene6611_YK, Unigene6670_YK, Unigene6814_YK, Unigene6850_YK, Unigene6915_YK, Unigene6980_YK, Unigene6_YK, Unigene7307_YK, Unigene7308_YK, Unigene7337_YK, Unigene7525_YK, Unigene7662_YK, Unigene7684_YK, Unigene771_YK, Unigene8101_YK, Unigene836_YK, Unigene838_YK, Unigene8396_YK, Unigene8755_YK, Unigene8877_YK, Unigene8892_YK, Unigene8893_YK, Unigene8908_YK, Unigene8997_YK, Unigene8999_YK, Unigene904_YK, Unigene905_YK, Unigene9150_YK, Unigene9198_YK, Unigene9323_YK, Unigene9373_YK, Unigene9388_YK, Unigene9459_YK, Unigene9710_YK, Unigene9937_YK, Unigene9989_YK |
| 7 | [Calcium signaling pathway](../../../../D:%5C高通量测序结果%5CF14FTSSCKF1242_NEMpnqE%5Cannotation%5CKEGG%5CYK-Unigene.fa_map%5Cmap04020.html) | CL1048.Contig1_YK, CL1048.Contig2_YK, CL1088.Contig1_YK, CL1088.Contig2_YK, CL1101.Contig1_YK, CL1101.Contig2_YK, CL1101.Contig3_YK, CL1101.Contig4_YK, CL112.Contig1_YK, CL112.Contig2_YK, CL112.Contig3_YK, CL112.Contig4_YK, CL112.Contig5_YK, CL112.Contig6_YK, CL112.Contig7_YK, CL112.Contig8_YK, CL1228.Contig1_YK, CL1242.Contig1_YK, CL1242.Contig2_YK, CL1260.Contig1_YK, CL1260.Contig2_YK, CL1260.Contig3_YK, CL1292.Contig1_YK, CL1292.Contig2_YK, CL15.Contig10_YK, CL15.Contig11_YK, CL15.Contig12_YK, CL15.Contig13_YK, CL15.Contig14_YK, CL15.Contig15_YK, CL15.Contig16_YK, CL15.Contig17_YK, CL15.Contig18_YK, CL15.Contig19_YK, CL15.Contig1_YK, CL15.Contig20_YK, CL15.Contig21_YK, CL15.Contig22_YK, CL15.Contig23_YK, CL15.Contig24_YK, CL15.Contig2_YK, CL15.Contig3_YK, CL15.Contig4_YK, CL15.Contig5_YK, CL15.Contig6_YK, CL15.Contig7_YK, CL15.Contig8_YK, CL15.Contig9_YK, CL1514.Contig1_YK, CL1514.Contig2_YK, CL1528.Contig1_YK, CL1528.Contig2_YK, CL1612.Contig1_YK, CL1612.Contig2_YK, CL1630.Contig2_YK, CL1632.Contig1_YK, CL1632.Contig2_YK, CL1666.Contig1_YK, CL1666.Contig2_YK, CL1689.Contig2_YK, CL1714.Contig2_YK, CL1852.Contig2_YK, CL1852.Contig3_YK, CL1861.Contig1_YK, CL1890.Contig1_YK, CL1890.Contig2_YK, CL1903.Contig1_YK, CL1903.Contig2_YK, CL1948.Contig1_YK, CL1948.Contig2_YK, CL1948.Contig3_YK, CL1990.Contig1_YK, CL1990.Contig2_YK, CL200.Contig1_YK, CL200.Contig2_YK, CL2032.Contig1_YK, CL2032.Contig2_YK, CL2103.Contig1_YK, CL2103.Contig2_YK, CL2135.Contig1_YK, CL2135.Contig2_YK, CL2224.Contig2_YK, CL2240.Contig1_YK, CL2240.Contig2_YK, CL2283.Contig1_YK, CL2283.Contig2_YK, CL2283.Contig3_YK, CL2283.Contig4_YK, CL2337.Contig1_YK, CL2337.Contig2_YK, CL2429.Contig1_YK, CL2429.Contig2_YK, CL2449.Contig1_YK, CL255.Contig1_YK, CL255.Contig2_YK, CL255.Contig3_YK, CL255.Contig4_YK, CL255.Contig5_YK, CL255.Contig6_YK, CL255.Contig7_YK, CL255.Contig8_YK, CL2593.Contig1_YK, CL2593.Contig2_YK, CL2593.Contig3_YK, CL2635.Contig1_YK, CL2635.Contig2_YK, CL275.Contig1_YK, CL275.Contig2_YK, CL275.Contig3_YK, CL2792.Contig1_YK, CL2894.Contig2_YK, CL295.Contig10_YK, CL295.Contig11_YK, CL295.Contig1_YK, CL295.Contig2_YK, CL295.Contig3_YK, CL295.Contig4_YK, CL295.Contig5_YK, CL295.Contig6_YK, CL295.Contig7_YK, CL295.Contig8_YK, CL295.Contig9_YK, CL307.Contig10_YK, CL307.Contig11_YK, CL307.Contig12_YK, CL307.Contig1_YK, CL307.Contig2_YK, CL307.Contig3_YK, CL307.Contig4_YK, CL307.Contig5_YK, CL307.Contig6_YK, CL307.Contig7_YK, CL307.Contig8_YK, CL307.Contig9_YK, CL34.Contig2_YK, CL34.Contig3_YK, CL353.Contig4_YK, CL431.Contig1_YK, CL431.Contig2_YK, CL431.Contig3_YK, CL475.Contig1_YK, CL475.Contig2_YK, CL475.Contig3_YK, CL475.Contig4_YK, CL527.Contig10_YK, CL527.Contig1_YK, CL527.Contig5_YK, CL527.Contig6_YK, CL527.Contig8_YK, CL527.Contig9_YK, CL529.Contig1_YK, CL529.Contig2_YK, CL529.Contig3_YK, CL529.Contig4_YK, CL529.Contig5_YK, CL529.Contig6_YK, CL568.Contig1_YK, CL568.Contig2_YK, CL591.Contig1_YK, CL591.Contig2_YK, CL591.Contig3_YK, CL591.Contig4_YK, CL591.Contig5_YK, CL591.Contig6_YK, CL591.Contig7_YK, CL591.Contig8_YK, CL663.Contig1_YK, CL663.Contig3_YK, CL79.Contig1_YK, CL79.Contig2_YK, CL79.Contig3_YK, CL79.Contig4_YK, CL79.Contig5_YK, CL79.Contig6_YK, CL79.Contig7_YK, CL79.Contig8_YK, CL838.Contig1_YK, CL838.Contig2_YK, CL838.Contig3_YK, CL838.Contig4_YK, CL838.Contig5_YK, CL838.Contig8_YK, CL85.Contig1_YK, CL85.Contig2_YK, CL85.Contig3_YK, CL884.Contig1_YK, CL884.Contig3_YK, CL899.Contig1_YK, CL902.Contig1_YK, CL902.Contig2_YK, CL957.Contig1_YK, CL957.Contig2_YK, Unigene10096_YK, Unigene10388_YK, Unigene10409_YK, Unigene10493_YK, Unigene10527_YK, Unigene10546_YK, Unigene10547_YK, Unigene10563_YK, Unigene10614_YK, Unigene10826_YK, Unigene10827_YK, Unigene10828_YK, Unigene10829_YK, Unigene10887_YK, Unigene10888_YK, Unigene10908_YK, Unigene11072_YK, Unigene11337_YK, Unigene11837_YK, Unigene11889_YK, Unigene11890_YK, Unigene11937_YK, Unigene12133_YK, Unigene12216_YK, Unigene12219_YK, Unigene1223_YK, Unigene12292_YK, Unigene12293_YK, Unigene12478_YK, Unigene12560_YK, Unigene12561_YK, Unigene12562_YK, Unigene12563_YK, Unigene12564_YK, Unigene12565_YK, Unigene12567_YK, Unigene12632_YK, Unigene12636_YK, Unigene12638_YK, Unigene12639_YK, Unigene12758_YK, Unigene12759_YK, Unigene12760_YK, Unigene12761_YK, Unigene12762_YK, Unigene12763_YK, Unigene12785_YK, Unigene13020_YK, Unigene13154_YK, Unigene13237_YK, Unigene13528_YK, Unigene13877_YK, Unigene13937_YK, Unigene13957_YK, Unigene13960_YK, Unigene14243_YK, Unigene14633_YK, Unigene14684_YK, Unigene14792_YK, Unigene14922_YK, Unigene15057_YK, Unigene15246_YK, Unigene15277_YK, Unigene15587_YK, Unigene158_YK, Unigene15974_YK, Unigene15979_YK, Unigene16000_YK, Unigene16057_YK, Unigene16102_YK, Unigene16406_YK, Unigene16420_YK, Unigene16426_YK, Unigene16804_YK, Unigene16843_YK, Unigene17117_YK, Unigene17811_YK, Unigene18183_YK, Unigene18577_YK, Unigene18798_YK, Unigene1964_YK, Unigene2287_YK, Unigene2289_YK, Unigene2550_YK, Unigene2657_YK, Unigene2719_YK, Unigene2770_YK, Unigene331_YK, Unigene352_YK, Unigene3585_YK, Unigene3626_YK, Unigene3937_YK, Unigene397_YK, Unigene4011_YK, Unigene4062_YK, Unigene4195_YK, Unigene4252_YK, Unigene4416_YK, Unigene4778_YK, Unigene4809_YK, Unigene4827_YK, Unigene4840_YK, Unigene4848_YK, Unigene5162_YK, Unigene5448_YK, Unigene5476_YK, Unigene5477_YK, Unigene5524_YK, Unigene5682_YK, Unigene5857_YK, Unigene5874_YK, Unigene5916_YK, Unigene5922_YK, Unigene6135_YK, Unigene6154_YK, Unigene6206_YK, Unigene6227_YK, Unigene6331_YK, Unigene6461_YK, Unigene6591_YK, Unigene6611_YK, Unigene6691_YK, Unigene6725_YK, Unigene6915_YK, Unigene6980_YK, Unigene6_YK, Unigene7307_YK, Unigene7308_YK, Unigene7382_YK, Unigene7390_YK, Unigene7511_YK, Unigene7525_YK, Unigene7610_YK, Unigene7611_YK, Unigene7822_YK, Unigene7823_YK, Unigene7824_YK, Unigene8101_YK, Unigene8130_YK, Unigene8144_YK, Unigene8301_YK, Unigene8396_YK, Unigene8745_YK, Unigene8867_YK, Unigene8868_YK, Unigene8877_YK, Unigene8908_YK, Unigene8953_YK, Unigene904_YK, Unigene905_YK, Unigene9459_YK, Unigene9581_YK, Unigene9710_YK |
| 8 | [Protein digestion and absorption](../../../../D:%5C高通量测序结果%5CF14FTSSCKF1242_NEMpnqE%5Cannotation%5CKEGG%5CYK-Unigene.fa_map%5Cmap04974.html) | CL1050.Contig1_YK, CL1050.Contig2_YK, CL1055.Contig1_YK, CL1055.Contig2_YK, CL1055.Contig3_YK, CL1058.Contig1_YK, CL1058.Contig2_YK, CL1058.Contig3_YK, CL1058.Contig4_YK, CL111.Contig2_YK, CL1185.Contig1_YK, CL1185.Contig2_YK, CL1230.Contig1_YK, CL1230.Contig2_YK, CL1230.Contig3_YK, CL1230.Contig4_YK, CL1260.Contig1_YK, CL1260.Contig2_YK, CL1260.Contig3_YK, CL134.Contig1_YK, CL1384.Contig1_YK, CL1384.Contig2_YK, CL1406.Contig1_YK, CL1406.Contig2_YK, CL1423.Contig1_YK, CL1423.Contig2_YK, CL1608.Contig2_YK, CL1675.Contig1_YK, CL1675.Contig2_YK, CL1751.Contig1_YK, CL1751.Contig2_YK, CL1771.Contig1_YK, CL1793.Contig2_YK, CL1793.Contig3_YK, CL1796.Contig1_YK, CL1796.Contig2_YK, CL1796.Contig3_YK, CL1796.Contig4_YK, CL1858.Contig1_YK, CL1858.Contig2_YK, CL1959.Contig2_YK, CL2031.Contig1_YK, CL2031.Contig2_YK, CL2083.Contig1_YK, CL2083.Contig2_YK, CL2083.Contig3_YK, CL2115.Contig3_YK, CL2141.Contig1_YK, CL2141.Contig2_YK, CL2141.Contig3_YK, CL2151.Contig1_YK, CL2181.Contig1_YK, CL2181.Contig2_YK, CL2243.Contig2_YK, CL23.Contig4_YK, CL23.Contig5_YK, CL2320.Contig1_YK, CL2320.Contig2_YK, CL2320.Contig3_YK, CL241.Contig1_YK, CL241.Contig3_YK, CL241.Contig4_YK, CL241.Contig7_YK, CL241.Contig9_YK, CL2468.Contig1_YK, CL2536.Contig1_YK, CL2537.Contig1_YK, CL2537.Contig2_YK, CL2562.Contig1_YK, CL2562.Contig2_YK, CL2742.Contig1_YK, CL2790.Contig1_YK, CL2886.Contig1_YK, CL2886.Contig2_YK, CL33.Contig4_YK, CL383.Contig17_YK, CL383.Contig18_YK, CL453.Contig1_YK, CL453.Contig2_YK, CL472.Contig1_YK, CL472.Contig2_YK, CL548.Contig2_YK, CL548.Contig3_YK, CL647.Contig1_YK, CL647.Contig2_YK, CL651.Contig1_YK, CL735.Contig2_YK, CL735.Contig4_YK, CL760.Contig1_YK, CL760.Contig2_YK, CL760.Contig3_YK, CL760.Contig4_YK, CL760.Contig5_YK, CL760.Contig6_YK, CL760.Contig7_YK, CL801.Contig1_YK, CL801.Contig2_YK, CL801.Contig3_YK, CL801.Contig4_YK, CL801.Contig5_YK, CL801.Contig6_YK, CL917.Contig2_YK, CL917.Contig3_YK, CL917.Contig5_YK, CL917.Contig6_YK, CL917.Contig7_YK, CL917.Contig8_YK, CL972.Contig1_YK, CL972.Contig2_YK, CL972.Contig3_YK, CL972.Contig4_YK, Unigene10001_YK, Unigene10034_YK, Unigene10341_YK, Unigene10403_YK, Unigene10404_YK, Unigene10444_YK, Unigene10470_YK, Unigene10544_YK, Unigene10550_YK, Unigene10551_YK, Unigene10591_YK, Unigene10653_YK, Unigene10664_YK, Unigene10672_YK, Unigene10714_YK, Unigene10716_YK, Unigene10950_YK, Unigene11098_YK, Unigene11127_YK, Unigene11224_YK, Unigene11251_YK, Unigene11316_YK, Unigene11317_YK, Unigene11363_YK, Unigene11364_YK, Unigene1150_YK, Unigene11640_YK, Unigene11704_YK, Unigene11705_YK, Unigene11768_YK, Unigene11845_YK, Unigene11846_YK, Unigene11860_YK, Unigene12017_YK, Unigene1208_YK, Unigene1210_YK, Unigene12127_YK, Unigene12190_YK, Unigene12249_YK, Unigene12352_YK, Unigene12353_YK, Unigene12433_YK, Unigene12473_YK, Unigene12535_YK, Unigene12536_YK, Unigene12537_YK, Unigene12538_YK, Unigene12539_YK, Unigene12541_YK, Unigene12542_YK, Unigene12543_YK, Unigene12544_YK, Unigene12548_YK, Unigene12711_YK, Unigene12712_YK, Unigene12715_YK, Unigene12777_YK, Unigene12888_YK, Unigene12889_YK, Unigene12977_YK, Unigene12991_YK, Unigene13055_YK, Unigene13061_YK, Unigene13103_YK, Unigene13108_YK, Unigene13110_YK, Unigene13253_YK, Unigene13293_YK, Unigene1332_YK, Unigene13550_YK, Unigene13750_YK, Unigene14007_YK, Unigene14048_YK, Unigene14060_YK, Unigene14090_YK, Unigene14093_YK, Unigene14399_YK, Unigene14486_YK, Unigene14492_YK, Unigene14710_YK, Unigene14914_YK, Unigene14954_YK, Unigene15065_YK, Unigene15350_YK, Unigene15413_YK, Unigene15602_YK, Unigene15673_YK, Unigene15706_YK, Unigene15719_YK, Unigene15802_YK, Unigene15898_YK, Unigene15921_YK, Unigene15943_YK, Unigene1602_YK, Unigene16031_YK, Unigene16150_YK, Unigene16193_YK, Unigene16222_YK, Unigene16238_YK, Unigene16322_YK, Unigene16382_YK, Unigene16514_YK, Unigene1660_YK, Unigene16613_YK, Unigene16698_YK, Unigene16865_YK, Unigene17112_YK, Unigene17170_YK, Unigene17212_YK, Unigene17547_YK, Unigene17738_YK, Unigene17960_YK, Unigene17990_YK, Unigene17996_YK, Unigene18007_YK, Unigene18305_YK, Unigene18946_YK, Unigene18969_YK, Unigene19070_YK, Unigene19113_YK, Unigene2190_YK, Unigene2221_YK, Unigene2230_YK, Unigene2433_YK, Unigene2486_YK, Unigene2563_YK, Unigene2685_YK, Unigene2770_YK, Unigene2799_YK, Unigene2853_YK, Unigene2991_YK, Unigene3145_YK, Unigene3195_YK, Unigene3198_YK, Unigene3224_YK, Unigene3591_YK, Unigene3660_YK, Unigene3828_YK, Unigene382_YK, Unigene3965_YK, Unigene3966_YK, Unigene39_YK, Unigene4060_YK, Unigene4108_YK, Unigene4344_YK, Unigene4376_YK, Unigene4465_YK, Unigene4531_YK, Unigene4561_YK, Unigene4574_YK, Unigene4576_YK, Unigene4585_YK, Unigene4658_YK, Unigene4659_YK, Unigene4675_YK, Unigene4730_YK, Unigene4781_YK, Unigene4963_YK, Unigene5102_YK, Unigene5118_YK, Unigene5128_YK, Unigene5202_YK, Unigene5235_YK, Unigene5536_YK, Unigene5584_YK, Unigene5585_YK, Unigene5603_YK, Unigene5634_YK, Unigene5763_YK, Unigene5787_YK, Unigene617_YK, Unigene6187_YK, Unigene625_YK, Unigene6261_YK, Unigene6268_YK, Unigene6450_YK, Unigene6454_YK, Unigene6475_YK, Unigene6685_YK, Unigene6686_YK, Unigene6725_YK, Unigene6921_YK, Unigene6981_YK, Unigene7100_YK, Unigene7101_YK, Unigene7147_YK, Unigene7148_YK, Unigene7175_YK, Unigene719_YK, Unigene7245_YK, Unigene7338_YK, Unigene7481_YK, Unigene7673_YK, Unigene7674_YK, Unigene7759_YK, Unigene7998_YK, Unigene8028_YK, Unigene8282_YK, Unigene8309_YK, Unigene8310_YK, Unigene8366_YK, Unigene8453_YK, Unigene8508_YK, Unigene8587_YK, Unigene8589_YK, Unigene8590_YK, Unigene8726_YK, Unigene8727_YK, Unigene8899_YK, Unigene8900_YK, Unigene9009_YK, Unigene9142_YK, Unigene9143_YK, Unigene9344_YK, Unigene9584_YK, Unigene9586_YK, Unigene9594_YK, Unigene9595_YK, Unigene96_YK, Unigene9714_YK, Unigene9715_YK, Unigene9716_YK, Unigene9717_YK, Unigene9849_YK, Unigene985_YK, Unigene9884_YK, Unigene9955_YK |
| 9 | [Amoebiasis](../../../../D:%5C高通量测序结果%5CF14FTSSCKF1242_NEMpnqE%5Cannotation%5CKEGG%5CYK-Unigene.fa_map%5Cmap05146.html) | CL1030.Contig1_YK, CL1030.Contig2_YK, CL1088.Contig1_YK, CL1088.Contig2_YK, CL1204.Contig2_YK, CL1204.Contig3_YK, CL1310.Contig6_YK, CL1358.Contig1_YK, CL1358.Contig2_YK, CL1362.Contig1_YK, CL1362.Contig2_YK, CL1362.Contig3_YK, CL1406.Contig1_YK, CL1406.Contig2_YK, CL1488.Contig1_YK, CL1488.Contig2_YK, CL15.Contig10_YK, CL15.Contig11_YK, CL15.Contig12_YK, CL15.Contig13_YK, CL15.Contig14_YK, CL15.Contig15_YK, CL15.Contig16_YK, CL15.Contig17_YK, CL15.Contig18_YK, CL15.Contig19_YK, CL15.Contig1_YK, CL15.Contig20_YK, CL15.Contig21_YK, CL15.Contig22_YK, CL15.Contig23_YK, CL15.Contig24_YK, CL15.Contig2_YK, CL15.Contig3_YK, CL15.Contig4_YK, CL15.Contig5_YK, CL15.Contig6_YK, CL15.Contig7_YK, CL15.Contig8_YK, CL15.Contig9_YK, CL1557.Contig1_YK, CL1557.Contig2_YK, CL1557.Contig3_YK, CL157.Contig1_YK, CL157.Contig2_YK, CL157.Contig3_YK, CL157.Contig4_YK, CL157.Contig6_YK, CL157.Contig7_YK, CL1591.Contig1_YK, CL1674.Contig1_YK, CL1674.Contig2_YK, CL1751.Contig1_YK, CL1751.Contig2_YK, CL1885.Contig1_YK, CL1951.Contig1_YK, CL1951.Contig2_YK, CL1963.Contig1_YK, CL1963.Contig2_YK, CL1998.Contig1_YK, CL1998.Contig3_YK, CL2020.Contig1_YK, CL2020.Contig2_YK, CL2031.Contig1_YK, CL2031.Contig2_YK, CL2037.Contig1_YK, CL2051.Contig1_YK, CL2051.Contig3_YK, CL2097.Contig2_YK, CL2097.Contig3_YK, CL2288.Contig1_YK, CL2309.Contig1_YK, CL2313.Contig1_YK, CL2313.Contig2_YK, CL2313.Contig3_YK, CL2355.Contig2_YK, CL2536.Contig1_YK, CL255.Contig1_YK, CL255.Contig2_YK, CL255.Contig3_YK, CL255.Contig4_YK, CL255.Contig5_YK, CL255.Contig6_YK, CL255.Contig7_YK, CL255.Contig8_YK, CL2562.Contig1_YK, CL2562.Contig2_YK, CL2601.Contig1_YK, CL2601.Contig2_YK, CL2751.Contig1_YK, CL2751.Contig2_YK, CL2792.Contig1_YK, CL2886.Contig1_YK, CL2886.Contig2_YK, CL300.Contig1_YK, CL300.Contig2_YK, CL307.Contig10_YK, CL307.Contig11_YK, CL307.Contig12_YK, CL307.Contig1_YK, CL307.Contig2_YK, CL307.Contig3_YK, CL307.Contig4_YK, CL307.Contig5_YK, CL307.Contig6_YK, CL307.Contig7_YK, CL307.Contig8_YK, CL307.Contig9_YK, CL308.Contig4_YK, CL308.Contig5_YK, CL472.Contig1_YK, CL472.Contig2_YK, CL502.Contig1_YK, CL502.Contig2_YK, CL591.Contig1_YK, CL591.Contig2_YK, CL591.Contig3_YK, CL591.Contig4_YK, CL591.Contig5_YK, CL591.Contig6_YK, CL591.Contig7_YK, CL591.Contig8_YK, CL647.Contig1_YK, CL647.Contig2_YK, CL760.Contig1_YK, CL760.Contig2_YK, CL760.Contig3_YK, CL760.Contig4_YK, CL760.Contig5_YK, CL760.Contig6_YK, CL760.Contig7_YK, CL807.Contig1_YK, CL807.Contig2_YK, CL884.Contig1_YK, CL884.Contig3_YK, CL917.Contig2_YK, CL917.Contig3_YK, CL917.Contig5_YK, CL917.Contig6_YK, CL917.Contig7_YK, CL917.Contig8_YK, Unigene10017_YK, Unigene10269_YK, Unigene10270_YK, Unigene10341_YK, Unigene10404_YK, Unigene1045_YK, Unigene10494_YK, Unigene10495_YK, Unigene10550_YK, Unigene10551_YK, Unigene10634_YK, Unigene10908_YK, Unigene10914_YK, Unigene10972_YK, Unigene10997_YK, Unigene10998_YK, Unigene11093_YK, Unigene11101_YK, Unigene11179_YK, Unigene11214_YK, Unigene11316_YK, Unigene11317_YK, Unigene11363_YK, Unigene11364_YK, Unigene11452_YK, Unigene11585_YK, Unigene11765_YK, Unigene11818_YK, Unigene11830_YK, Unigene11860_YK, Unigene12013_YK, Unigene12017_YK, Unigene12216_YK, Unigene12219_YK, Unigene1223_YK, Unigene12242_YK, Unigene12281_YK, Unigene12352_YK, Unigene12353_YK, Unigene12535_YK, Unigene12536_YK, Unigene12537_YK, Unigene12538_YK, Unigene12539_YK, Unigene12541_YK, Unigene12542_YK, Unigene12543_YK, Unigene12544_YK, Unigene12548_YK, Unigene12555_YK, Unigene12588_YK, Unigene12602_YK, Unigene12636_YK, Unigene12638_YK, Unigene12639_YK, Unigene12734_YK, Unigene12743_YK, Unigene12777_YK, Unigene12823_YK, Unigene12824_YK, Unigene12977_YK, Unigene12984_YK, Unigene12991_YK, Unigene13083_YK, Unigene13108_YK, Unigene13109_YK, Unigene13137_YK, Unigene13237_YK, Unigene13293_YK, Unigene13883_YK, Unigene13989_YK, Unigene14007_YK, Unigene14093_YK, Unigene14710_YK, Unigene14914_YK, Unigene14964_YK, Unigene15160_YK, Unigene15251_YK, Unigene15350_YK, Unigene15413_YK, Unigene15497_YK, Unigene15673_YK, Unigene15802_YK, Unigene15847_YK, Unigene158_YK, Unigene15943_YK, Unigene16034_YK, Unigene1660_YK, Unigene16698_YK, Unigene16724_YK, Unigene16777_YK, Unigene17212_YK, Unigene17547_YK, Unigene17960_YK, Unigene17973_YK, Unigene17996_YK, Unigene18172_YK, Unigene18183_YK, Unigene18305_YK, Unigene18577_YK, Unigene18946_YK, Unigene19070_YK, Unigene1964_YK, Unigene2230_YK, Unigene2289_YK, Unigene2330_YK, Unigene2433_YK, Unigene2486_YK, Unigene256_YK, Unigene2722_YK, Unigene2791_YK, Unigene2799_YK, Unigene303_YK, Unigene3127_YK, Unigene3195_YK, Unigene3198_YK, Unigene3224_YK, Unigene331_YK, Unigene347_YK, Unigene3591_YK, Unigene3657_YK, Unigene3660_YK, Unigene3718_YK, Unigene3769_YK, Unigene3828_YK, Unigene3937_YK, Unigene3965_YK, Unigene3966_YK, Unigene4019_YK, Unigene4055_YK, Unigene4060_YK, Unigene4081_YK, Unigene41_YK, Unigene4252_YK, Unigene4458_YK, Unigene4465_YK, Unigene4576_YK, Unigene4781_YK, Unigene4900_YK, Unigene4963_YK, Unigene5065_YK, Unigene5202_YK, Unigene5476_YK, Unigene5477_YK, Unigene5741_YK, Unigene5844_YK, Unigene5857_YK, Unigene5863_YK, Unigene59_YK, Unigene6060_YK, Unigene6105_YK, Unigene6182_YK, Unigene6206_YK, Unigene6261_YK, Unigene6268_YK, Unigene6561_YK, Unigene6685_YK, Unigene6686_YK, Unigene6737_YK, Unigene6980_YK, Unigene6981_YK, Unigene70_YK, Unigene7496_YK, Unigene7525_YK, Unigene7590_YK, Unigene7610_YK, Unigene7611_YK, Unigene7998_YK, Unigene8101_YK, Unigene8132_YK, Unigene8134_YK, Unigene8232_YK, Unigene8233_YK, Unigene8282_YK, Unigene8323_YK, Unigene8324_YK, Unigene8325_YK, Unigene8364_YK, Unigene8365_YK, Unigene8371_YK, Unigene8449_YK, Unigene8450_YK, Unigene8451_YK, Unigene8452_YK, Unigene8500_YK, Unigene8501_YK, Unigene88_YK, Unigene904_YK, Unigene905_YK, Unigene9155_YK, Unigene952_YK, Unigene9584_YK, Unigene9634_YK |
| 10 | [Spliceosome](../../../../D:%5C高通量测序结果%5CF14FTSSCKF1242_NEMpnqE%5Cannotation%5CKEGG%5CYK-Unigene.fa_map%5Cmap03040.html) | CL1166.Contig2_YK, CL1204.Contig1_YK, CL1404.Contig2_YK, CL1451.Contig1_YK, CL1451.Contig2_YK, CL1563.Contig1_YK, CL1563.Contig2_YK, CL1637.Contig1_YK, CL1637.Contig2_YK, CL1637.Contig3_YK, CL1637.Contig4_YK, CL1637.Contig5_YK, CL1637.Contig6_YK, CL1637.Contig7_YK, CL1637.Contig8_YK, CL1693.Contig1_YK, CL1693.Contig2_YK, CL1693.Contig3_YK, CL1693.Contig4_YK, CL1693.Contig5_YK, CL1800.Contig1_YK, CL1800.Contig2_YK, CL1807.Contig1_YK, CL1839.Contig1_YK, CL1839.Contig2_YK, CL1839.Contig3_YK, CL1839.Contig4_YK, CL1915.Contig1_YK, CL1915.Contig2_YK, CL1915.Contig3_YK, CL198.Contig1_YK, CL198.Contig2_YK, CL198.Contig3_YK, CL2063.Contig1_YK, CL2063.Contig2_YK, CL2106.Contig1_YK, CL2106.Contig2_YK, CL2107.Contig1_YK, CL2125.Contig2_YK, CL2132.Contig1_YK, CL2132.Contig2_YK, CL2201.Contig1_YK, CL2201.Contig2_YK, CL2201.Contig3_YK, CL2215.Contig1_YK, CL2215.Contig2_YK, CL224.Contig1_YK, CL224.Contig2_YK, CL2253.Contig1_YK, CL2253.Contig2_YK, CL238.Contig2_YK, CL238.Contig3_YK, CL2388.Contig1_YK, CL2388.Contig2_YK, CL2388.Contig3_YK, CL2431.Contig1_YK, CL2431.Contig2_YK, CL2514.Contig1_YK, CL2514.Contig2_YK, CL2534.Contig1_YK, CL2534.Contig2_YK, CL2609.Contig2_YK, CL2633.Contig1_YK, CL2633.Contig2_YK, CL2680.Contig1_YK, CL2694.Contig1_YK, CL2694.Contig2_YK, CL2699.Contig1_YK, CL2699.Contig2_YK, CL2712.Contig1_YK, CL2720.Contig1_YK, CL2805.Contig1_YK, CL2866.Contig2_YK, CL2885.Contig1_YK, CL2885.Contig2_YK, CL350.Contig1_YK, CL350.Contig2_YK, CL350.Contig3_YK, CL368.Contig1_YK, CL368.Contig2_YK, CL490.Contig10_YK, CL490.Contig1_YK, CL490.Contig2_YK, CL490.Contig5_YK, CL522.Contig1_YK, CL522.Contig2_YK, CL522.Contig3_YK, CL561.Contig1_YK, CL561.Contig2_YK, CL674.Contig3_YK, CL674.Contig5_YK, CL674.Contig7_YK, CL674.Contig9_YK, CL700.Contig1_YK, CL700.Contig2_YK, CL749.Contig1_YK, CL749.Contig2_YK, CL789.Contig2_YK, CL811.Contig1_YK, CL811.Contig2_YK, CL811.Contig3_YK, CL811.Contig4_YK, CL869.Contig1_YK, CL869.Contig2_YK, CL906.Contig1_YK, CL906.Contig2_YK, CL906.Contig3_YK, CL922.Contig1_YK, Unigene10079_YK, Unigene10125_YK, Unigene10227_YK, Unigene102_YK, Unigene10335_YK, Unigene10433_YK, Unigene10728_YK, Unigene10892_YK, Unigene11160_YK, Unigene11298_YK, Unigene11685_YK, Unigene11690_YK, Unigene11691_YK, Unigene11692_YK, Unigene11886_YK, Unigene11972_YK, Unigene11993_YK, Unigene12068_YK, Unigene12069_YK, Unigene12070_YK, Unigene12071_YK, Unigene12210_YK, Unigene1225_YK, Unigene12429_YK, Unigene12430_YK, Unigene12524_YK, Unigene12583_YK, Unigene12623_YK, Unigene1272_YK, Unigene13039_YK, Unigene13123_YK, Unigene13193_YK, Unigene13204_YK, Unigene13217_YK, Unigene13321_YK, Unigene13339_YK, Unigene13340_YK, Unigene13341_YK, Unigene13343_YK, Unigene13379_YK, Unigene13391_YK, Unigene13416_YK, Unigene13432_YK, Unigene13436_YK, Unigene13469_YK, Unigene1346_YK, Unigene13503_YK, Unigene13518_YK, Unigene13546_YK, Unigene13553_YK, Unigene13610_YK, Unigene13620_YK, Unigene13628_YK, Unigene13636_YK, Unigene13714_YK, Unigene13820_YK, Unigene13865_YK, Unigene13868_YK, Unigene13918_YK, Unigene1393_YK, Unigene13982_YK, Unigene13987_YK, Unigene14023_YK, Unigene14029_YK, Unigene14066_YK, Unigene14077_YK, Unigene14111_YK, Unigene14128_YK, Unigene14151_YK, Unigene14167_YK, Unigene14168_YK, Unigene14174_YK, Unigene14230_YK, Unigene14247_YK, Unigene14267_YK, Unigene14290_YK, Unigene14291_YK, Unigene14323_YK, Unigene14366_YK, Unigene14400_YK, Unigene14405_YK, Unigene14418_YK, Unigene14463_YK, Unigene14479_YK, Unigene14517_YK, Unigene14603_YK, Unigene14617_YK, Unigene14644_YK, Unigene14665_YK, Unigene14668_YK, Unigene14738_YK, Unigene14763_YK, Unigene147_YK, Unigene14849_YK, Unigene14879_YK, Unigene14883_YK, Unigene15008_YK, Unigene15021_YK, Unigene15103_YK, Unigene15208_YK, Unigene15246_YK, Unigene15250_YK, Unigene15503_YK, Unigene15616_YK, Unigene16055_YK, Unigene16144_YK, Unigene16187_YK, Unigene16199_YK, Unigene16230_YK, Unigene163_YK, Unigene1656_YK, Unigene1657_YK, Unigene16951_YK, Unigene17455_YK, Unigene17543_YK, Unigene17562_YK, Unigene17614_YK, Unigene17725_YK, Unigene18508_YK, Unigene18811_YK, Unigene18943_YK, Unigene18972_YK, Unigene19039_YK, Unigene19055_YK, Unigene19095_YK, Unigene197_YK, Unigene2103_YK, Unigene2342_YK, Unigene260_YK, Unigene2716_YK, Unigene2891_YK, Unigene2901_YK, Unigene303_YK, Unigene3100_YK, Unigene3124_YK, Unigene3162_YK, Unigene3181_YK, Unigene3328_YK, Unigene3388_YK, Unigene3516_YK, Unigene3552_YK, Unigene3553_YK, Unigene3690_YK, Unigene3694_YK, Unigene3767_YK, Unigene3887_YK, Unigene3888_YK, Unigene3974_YK, Unigene4068_YK, Unigene4143_YK, Unigene4407_YK, Unigene4525_YK, Unigene4660_YK, Unigene4720_YK, Unigene4753_YK, Unigene4794_YK, Unigene5010_YK, Unigene5117_YK, Unigene5206_YK, Unigene5381_YK, Unigene5408_YK, Unigene5440_YK, Unigene5441_YK, Unigene5469_YK, Unigene5784_YK, Unigene5825_YK, Unigene5858_YK, Unigene5953_YK, Unigene6017_YK, Unigene6205_YK, Unigene6238_YK, Unigene6416_YK, Unigene6961_YK, Unigene7012_YK, Unigene7370_YK, Unigene7427_YK, Unigene7743_YK, Unigene7837_YK, Unigene7838_YK, Unigene7839_YK, Unigene7875_YK, Unigene8100_YK, Unigene8397_YK, Unigene8413_YK, Unigene8471_YK, Unigene8835_YK, Unigene8902_YK, Unigene9057_YK, Unigene9416_YK, Unigene9993_YK, Unigene9995_YK |
| 11 | [Alzheimer's disease](../../../../D:%5C高通量测序结果%5CF14FTSSCKF1242_NEMpnqE%5Cannotation%5CKEGG%5CYK-Unigene.fa_map%5Cmap05010.html) | CL1103.Contig1_YK, CL1247.Contig1_YK, CL1247.Contig2_YK, CL1284.Contig1_YK, CL1284.Contig2_YK, CL1288.Contig1_YK, CL134.Contig1_YK, CL15.Contig10_YK, CL15.Contig11_YK, CL15.Contig12_YK, CL15.Contig13_YK, CL15.Contig14_YK, CL15.Contig15_YK, CL15.Contig16_YK, CL15.Contig17_YK, CL15.Contig18_YK, CL15.Contig19_YK, CL15.Contig1_YK, CL15.Contig20_YK, CL15.Contig21_YK, CL15.Contig22_YK, CL15.Contig23_YK, CL15.Contig24_YK, CL15.Contig2_YK, CL15.Contig3_YK, CL15.Contig4_YK, CL15.Contig5_YK, CL15.Contig6_YK, CL15.Contig7_YK, CL15.Contig8_YK, CL15.Contig9_YK, CL1611.Contig1_YK, CL1611.Contig2_YK, CL1682.Contig1_YK, CL1714.Contig2_YK, CL1771.Contig1_YK, CL1797.Contig1_YK, CL1797.Contig2_YK, CL1797.Contig3_YK, CL1861.Contig1_YK, CL2135.Contig1_YK, CL2135.Contig2_YK, CL2294.Contig1_YK, CL2294.Contig2_YK, CL23.Contig4_YK, CL23.Contig5_YK, CL241.Contig1_YK, CL241.Contig3_YK, CL241.Contig4_YK, CL241.Contig7_YK, CL241.Contig9_YK, CL2593.Contig1_YK, CL2593.Contig2_YK, CL2593.Contig3_YK, CL2626.Contig1_YK, CL2626.Contig2_YK, CL2635.Contig1_YK, CL2635.Contig2_YK, CL2668.Contig1_YK, CL2668.Contig2_YK, CL295.Contig10_YK, CL295.Contig2_YK, CL295.Contig3_YK, CL295.Contig4_YK, CL295.Contig6_YK, CL295.Contig8_YK, CL295.Contig9_YK, CL307.Contig10_YK, CL307.Contig11_YK, CL307.Contig12_YK, CL307.Contig1_YK, CL307.Contig2_YK, CL307.Contig3_YK, CL307.Contig4_YK, CL307.Contig5_YK, CL307.Contig6_YK, CL307.Contig7_YK, CL307.Contig8_YK, CL307.Contig9_YK, CL35.Contig1_YK, CL35.Contig2_YK, CL361.Contig3_YK, CL361.Contig6_YK, CL431.Contig1_YK, CL431.Contig2_YK, CL431.Contig3_YK, CL459.Contig1_YK, CL459.Contig2_YK, CL548.Contig2_YK, CL548.Contig3_YK, CL564.Contig10_YK, CL564.Contig13_YK, CL564.Contig14_YK, CL564.Contig16_YK, CL564.Contig2_YK, CL564.Contig3_YK, CL564.Contig5_YK, CL564.Contig6_YK, CL564.Contig7_YK, CL564.Contig8_YK, CL564.Contig9_YK, CL568.Contig1_YK, CL568.Contig2_YK, CL735.Contig2_YK, CL735.Contig4_YK, CL774.Contig1_YK, CL774.Contig2_YK, CL838.Contig1_YK, CL838.Contig2_YK, CL838.Contig3_YK, CL838.Contig4_YK, CL838.Contig5_YK, CL838.Contig8_YK, CL902.Contig1_YK, CL902.Contig2_YK, CL957.Contig1_YK, CL957.Contig2_YK, Unigene10096_YK, Unigene10445_YK, Unigene10546_YK, Unigene10547_YK, Unigene10577_YK, Unigene10591_YK, Unigene10614_YK, Unigene10777_YK, Unigene10895_YK, Unigene11740_YK, Unigene11768_YK, Unigene11889_YK, Unigene12025_YK, Unigene1210_YK, Unigene12259_YK, Unigene12376_YK, Unigene12560_YK, Unigene12561_YK, Unigene12563_YK, Unigene12564_YK, Unigene12636_YK, Unigene12638_YK, Unigene12639_YK, Unigene12711_YK, Unigene12712_YK, Unigene12715_YK, Unigene12743_YK, Unigene12888_YK, Unigene12889_YK, Unigene12986_YK, Unigene12990_YK, Unigene13063_YK, Unigene13065_YK, Unigene13135_YK, Unigene13166_YK, Unigene13229_YK, Unigene13231_YK, Unigene13234_YK, Unigene13375_YK, Unigene13401_YK, Unigene13473_YK, Unigene13483_YK, Unigene13484_YK, Unigene13507_YK, Unigene1356_YK, Unigene13584_YK, Unigene13592_YK, Unigene13607_YK, Unigene13608_YK, Unigene13639_YK, Unigene13643_YK, Unigene13674_YK, Unigene13708_YK, Unigene13785_YK, Unigene13801_YK, Unigene13829_YK, Unigene13847_YK, Unigene13877_YK, Unigene13878_YK, Unigene13937_YK, Unigene13960_YK, Unigene14028_YK, Unigene14030_YK, Unigene14052_YK, Unigene14053_YK, Unigene14054_YK, Unigene14117_YK, Unigene14165_YK, Unigene14204_YK, Unigene14243_YK, Unigene14283_YK, Unigene14309_YK, Unigene14331_YK, Unigene14392_YK, Unigene14415_YK, Unigene14455_YK, Unigene14482_YK, Unigene14510_YK, Unigene14527_YK, Unigene14542_YK, Unigene14670_YK, Unigene14709_YK, Unigene14892_YK, Unigene14922_YK, Unigene14977_YK, Unigene15028_YK, Unigene15040_YK, Unigene15057_YK, Unigene15276_YK, Unigene15523_YK, Unigene15529_YK, Unigene15561_YK, Unigene15587_YK, Unigene15764_YK, Unigene158_YK, Unigene16000_YK, Unigene16514_YK, Unigene16613_YK, Unigene16835_YK, Unigene16865_YK, Unigene1730_YK, Unigene17811_YK, Unigene18156_YK, Unigene18679_YK, Unigene19067_YK, Unigene19105_YK, Unigene19109_YK, Unigene19113_YK, Unigene1964_YK, Unigene2249_YK, Unigene2440_YK, Unigene2550_YK, Unigene2785_YK, Unigene2851_YK, Unigene2883_YK, Unigene3133_YK, Unigene3140_YK, Unigene319_YK, Unigene3251_YK, Unigene3397_YK, Unigene3626_YK, Unigene3868_YK, Unigene3905_YK, Unigene39_YK, Unigene4022_YK, Unigene4033_YK, Unigene4062_YK, Unigene4165_YK, Unigene4166_YK, Unigene4252_YK, Unigene4445_YK, Unigene4454_YK, Unigene4561_YK, Unigene4778_YK, Unigene4979_YK, Unigene4_YK, Unigene5018_YK, Unigene5019_YK, Unigene5118_YK, Unigene5322_YK, Unigene5412_YK, Unigene5476_YK, Unigene5477_YK, Unigene5619_YK, Unigene5634_YK, Unigene5832_YK, Unigene5857_YK, Unigene5859_YK, Unigene5975_YK, Unigene6006_YK, Unigene6007_YK, Unigene6206_YK, Unigene6227_YK, Unigene6461_YK, Unigene6475_YK, Unigene6611_YK, Unigene6664_YK, Unigene6789_YK, Unigene6_YK, Unigene719_YK, Unigene7247_YK, Unigene7307_YK, Unigene7308_YK, Unigene7382_YK, Unigene7390_YK, Unigene7661_YK, Unigene7673_YK, Unigene7816_YK, Unigene7994_YK, Unigene7995_YK, Unigene8693_YK, Unigene8694_YK, Unigene9009_YK, Unigene9209_YK, Unigene9459_YK, Unigene9735_YK, Unigene9736_YK, Unigene985_YK, Unigene9903_YK, Unigene9904_YK, Unigene9968_YK, Unigene9969_YK |
| 12 | [MAPK signaling pathway](../../../../D:%5C高通量测序结果%5CF14FTSSCKF1242_NEMpnqE%5Cannotation%5CKEGG%5CYK-Unigene.fa_map%5Cmap04010.html) | CL1003.Contig1_YK, CL1003.Contig2_YK, CL1014.Contig1_YK, CL1014.Contig2_YK, CL1075.Contig1_YK, CL1075.Contig2_YK, CL1088.Contig1_YK, CL1088.Contig2_YK, CL1098.Contig1_YK, CL1098.Contig2_YK, CL1278.Contig1_YK, CL1278.Contig2_YK, CL1292.Contig1_YK, CL1292.Contig2_YK, CL136.Contig1_YK, CL136.Contig2_YK, CL136.Contig3_YK, CL136.Contig4_YK, CL136.Contig5_YK, CL1381.Contig1_YK, CL1381.Contig2_YK, CL1469.Contig1_YK, CL1476.Contig1_YK, CL1476.Contig2_YK, CL1476.Contig3_YK, CL1476.Contig4_YK, CL1482.Contig1_YK, CL1482.Contig2_YK, CL1482.Contig3_YK, CL1602.Contig1_YK, CL1602.Contig2_YK, CL1653.Contig1_YK, CL1653.Contig2_YK, CL1666.Contig1_YK, CL1666.Contig2_YK, CL1680.Contig1_YK, CL1680.Contig2_YK, CL1689.Contig2_YK, CL1691.Contig1_YK, CL1691.Contig2_YK, CL1758.Contig1_YK, CL1758.Contig2_YK, CL1783.Contig2_YK, CL1969.Contig1_YK, CL1969.Contig2_YK, CL1977.Contig1_YK, CL1977.Contig2_YK, CL1977.Contig3_YK, CL1977.Contig4_YK, CL198.Contig1_YK, CL198.Contig2_YK, CL198.Contig3_YK, CL2047.Contig1_YK, CL2047.Contig2_YK, CL2047.Contig3_YK, CL2076.Contig1_YK, CL2076.Contig2_YK, CL2128.Contig1_YK, CL2128.Contig2_YK, CL2128.Contig3_YK, CL2128.Contig4_YK, CL2128.Contig5_YK, CL2128.Contig6_YK, CL2135.Contig1_YK, CL2135.Contig2_YK, CL2153.Contig1_YK, CL2153.Contig2_YK, CL2168.Contig1_YK, CL218.Contig1_YK, CL218.Contig2_YK, CL2247.Contig1_YK, CL2247.Contig2_YK, CL2429.Contig1_YK, CL2429.Contig2_YK, CL2447.Contig1_YK, CL2447.Contig2_YK, CL2449.Contig1_YK, CL255.Contig1_YK, CL255.Contig2_YK, CL255.Contig3_YK, CL255.Contig4_YK, CL255.Contig5_YK, CL255.Contig6_YK, CL255.Contig7_YK, CL255.Contig8_YK, CL2624.Contig1_YK, CL2624.Contig2_YK, CL2635.Contig1_YK, CL2635.Contig2_YK, CL2681.Contig1_YK, CL2720.Contig1_YK, CL2764.Contig1_YK, CL2764.Contig2_YK, CL2793.Contig1_YK, CL2793.Contig2_YK, CL2831.Contig1_YK, CL2831.Contig2_YK, CL2894.Contig1_YK, CL2894.Contig2_YK, CL2896.Contig1_YK, CL411.Contig1_YK, CL411.Contig2_YK, CL411.Contig3_YK, CL43.Contig1_YK, CL43.Contig2_YK, CL431.Contig1_YK, CL431.Contig2_YK, CL431.Contig3_YK, CL438.Contig2_YK, CL438.Contig3_YK, CL44.Contig1_YK, CL44.Contig2_YK, CL516.Contig1_YK, CL526.Contig1_YK, CL526.Contig2_YK, CL539.Contig1_YK, CL539.Contig2_YK, CL539.Contig3_YK, CL539.Contig4_YK, CL591.Contig1_YK, CL591.Contig2_YK, CL591.Contig3_YK, CL591.Contig4_YK, CL591.Contig5_YK, CL591.Contig6_YK, CL591.Contig7_YK, CL591.Contig8_YK, CL592.Contig2_YK, CL732.Contig1_YK, CL732.Contig2_YK, CL832.Contig1_YK, CL838.Contig1_YK, CL838.Contig2_YK, CL838.Contig3_YK, CL838.Contig4_YK, CL838.Contig5_YK, CL838.Contig6_YK, CL838.Contig7_YK, CL838.Contig8_YK, CL884.Contig1_YK, CL884.Contig3_YK, CL921.Contig1_YK, CL921.Contig2_YK, Unigene10096_YK, Unigene10197_YK, Unigene10198_YK, Unigene10546_YK, Unigene10547_YK, Unigene10659_YK, Unigene10746_YK, Unigene107_YK, Unigene10819_YK, Unigene11167_YK, Unigene11274_YK, Unigene11299_YK, Unigene11335_YK, Unigene11478_YK, Unigene11546_YK, Unigene11748_YK, Unigene11784_YK, Unigene11865_YK, Unigene11889_YK, Unigene11890_YK, Unigene12216_YK, Unigene12218_YK, Unigene12219_YK, Unigene1225_YK, Unigene12358_YK, Unigene12436_YK, Unigene12451_YK, Unigene12532_YK, Unigene12743_YK, Unigene13009_YK, Unigene13023_YK, Unigene13203_YK, Unigene13417_YK, Unigene13424_YK, Unigene13436_YK, Unigene13571_YK, Unigene13658_YK, Unigene13670_YK, Unigene13674_YK, Unigene13692_YK, Unigene13723_YK, Unigene13877_YK, Unigene13883_YK, Unigene1393_YK, Unigene14159_YK, Unigene14278_YK, Unigene14296_YK, Unigene14345_YK, Unigene14432_YK, Unigene14456_YK, Unigene14507_YK, Unigene14647_YK, Unigene15178_YK, Unigene15273_YK, Unigene15482_YK, Unigene15497_YK, Unigene15607_YK, Unigene15698_YK, Unigene15786_YK, Unigene15798_YK, Unigene15910_YK, Unigene16000_YK, Unigene16250_YK, Unigene1656_YK, Unigene1657_YK, Unigene16951_YK, Unigene17614_YK, Unigene17725_YK, Unigene17790_YK, Unigene18490_YK, Unigene18574_YK, Unigene18819_YK, Unigene18931_YK, Unigene18969_YK, Unigene19055_YK, Unigene2111_YK, Unigene2116_YK, Unigene2324_YK, Unigene2607_YK, Unigene2913_YK, Unigene3028_YK, Unigene3029_YK, Unigene317_YK, Unigene3286_YK, Unigene3295_YK, Unigene331_YK, Unigene3430_YK, Unigene3439_YK, Unigene3491_YK, Unigene3667_YK, Unigene3692_YK, Unigene372_YK, Unigene3852_YK, Unigene3929_YK, Unigene4062_YK, Unigene4068_YK, Unigene4128_YK, Unigene4190_YK, Unigene4203_YK, Unigene4494_YK, Unigene4505_YK, Unigene4660_YK, Unigene4680_YK, Unigene4754_YK, Unigene4861_YK, Unigene4954_YK, Unigene5045_YK, Unigene5106_YK, Unigene5219_YK, Unigene5447_YK, Unigene5504_YK, Unigene5642_YK, Unigene5908_YK, Unigene5924_YK, Unigene6229_YK, Unigene6230_YK, Unigene6231_YK, Unigene6417_YK, Unigene6444_YK, Unigene6535_YK, Unigene6561_YK, Unigene6670_YK, Unigene6679_YK, Unigene6820_YK, Unigene6907_YK, Unigene712_YK, Unigene7324_YK, Unigene7330_YK, Unigene7382_YK, Unigene7525_YK, Unigene7755_YK, Unigene7837_YK, Unigene7838_YK, Unigene7839_YK, Unigene7920_YK, Unigene8001_YK, Unigene8577_YK, Unigene8578_YK, Unigene8755_YK, Unigene8835_YK, Unigene9071_YK, Unigene9480_YK, Unigene9483_YK, Unigene9484_YK, Unigene9485_YK, Unigene9486_YK, Unigene9649_YK, Unigene9678_YK, Unigene9705_YK, Unigene9732_YK |
| 13 | [HTLV-I infection](../../../../D:%5C高通量测序结果%5CF14FTSSCKF1242_NEMpnqE%5Cannotation%5CKEGG%5CYK-Unigene.fa_map%5Cmap05166.html) | CL1088.Contig1_YK, CL1088.Contig2_YK, CL1098.Contig1_YK, CL1098.Contig2_YK, CL1170.Contig1_YK, CL1170.Contig2_YK, CL1170.Contig3_YK, CL1170.Contig4_YK, CL1228.Contig1_YK, CL1302.Contig3_YK, CL1302.Contig4_YK, CL1390.Contig1_YK, CL1390.Contig2_YK, CL1412.Contig1_YK, CL1412.Contig2_YK, CL1412.Contig3_YK, CL1464.Contig1_YK, CL1476.Contig1_YK, CL1476.Contig2_YK, CL1476.Contig3_YK, CL1476.Contig4_YK, CL1505.Contig1_YK, CL1505.Contig2_YK, CL1514.Contig1_YK, CL1514.Contig2_YK, CL1538.Contig1_YK, CL1538.Contig2_YK, CL1578.Contig1_YK, CL1578.Contig2_YK, CL1653.Contig1_YK, CL1653.Contig2_YK, CL1679.Contig1_YK, CL1679.Contig2_YK, CL1679.Contig3_YK, CL1758.Contig1_YK, CL1758.Contig2_YK, CL1918.Contig1_YK, CL1918.Contig2_YK, CL1977.Contig1_YK, CL1977.Contig2_YK, CL1977.Contig3_YK, CL1977.Contig4_YK, CL204.Contig1_YK, CL204.Contig2_YK, CL2135.Contig1_YK, CL2135.Contig2_YK, CL2281.Contig1_YK, CL2281.Contig2_YK, CL2336.Contig1_YK, CL2336.Contig2_YK, CL2353.Contig1_YK, CL2353.Contig2_YK, CL2402.Contig1_YK, CL2402.Contig2_YK, CL2414.Contig1_YK, CL2414.Contig2_YK, CL255.Contig1_YK, CL255.Contig2_YK, CL255.Contig3_YK, CL255.Contig4_YK, CL255.Contig5_YK, CL255.Contig6_YK, CL255.Contig7_YK, CL255.Contig8_YK, CL2635.Contig1_YK, CL2635.Contig2_YK, CL2666.Contig1_YK, CL2666.Contig2_YK, CL2668.Contig1_YK, CL2668.Contig2_YK, CL2672.Contig2_YK, CL2729.Contig1_YK, CL2791.Contig1_YK, CL2791.Contig2_YK, CL2792.Contig1_YK, CL2793.Contig1_YK, CL2793.Contig2_YK, CL2866.Contig1_YK, CL374.Contig1_YK, CL387.Contig1_YK, CL387.Contig2_YK, CL387.Contig3_YK, CL387.Contig4_YK, CL387.Contig5_YK, CL431.Contig1_YK, CL431.Contig2_YK, CL431.Contig3_YK, CL438.Contig2_YK, CL438.Contig3_YK, CL692.Contig1_YK, CL692.Contig2_YK, CL692.Contig3_YK, CL692.Contig4_YK, CL692.Contig5_YK, CL692.Contig6_YK, CL692.Contig7_YK, CL692.Contig8_YK, CL754.Contig1_YK, CL754.Contig2_YK, CL774.Contig1_YK, CL774.Contig2_YK, CL79.Contig1_YK, CL79.Contig2_YK, CL79.Contig3_YK, CL79.Contig4_YK, CL79.Contig5_YK, CL79.Contig6_YK, CL79.Contig7_YK, CL79.Contig8_YK, CL812.Contig1_YK, CL812.Contig2_YK, CL814.Contig1_YK, CL814.Contig2_YK, CL83.Contig4_YK, CL882.Contig1_YK, CL882.Contig2_YK, CL882.Contig3_YK, CL882.Contig4_YK, CL882.Contig5_YK, CL882.Contig6_YK, CL882.Contig7_YK, CL916.Contig1_YK, CL916.Contig2_YK, Unigene10015_YK, Unigene10072_YK, Unigene10096_YK, Unigene1009_YK, Unigene10409_YK, Unigene104_YK, Unigene10546_YK, Unigene10547_YK, Unigene10659_YK, Unigene10746_YK, Unigene10908_YK, Unigene10954_YK, Unigene10961_YK, Unigene10972_YK, Unigene11008_YK, Unigene11075_YK, Unigene11204_YK, Unigene11299_YK, Unigene11335_YK, Unigene11478_YK, Unigene11566_YK, Unigene1158_YK, Unigene11682_YK, Unigene12058_YK, Unigene12068_YK, Unigene12069_YK, Unigene12070_YK, Unigene12071_YK, Unigene12133_YK, Unigene12216_YK, Unigene12218_YK, Unigene12219_YK, Unigene12292_YK, Unigene12293_YK, Unigene12354_YK, Unigene12777_YK, Unigene13015_YK, Unigene13128_YK, Unigene13281_YK, Unigene13327_YK, Unigene13430_YK, Unigene13494_YK, Unigene13531_YK, Unigene13597_YK, Unigene13618_YK, Unigene13658_YK, Unigene13670_YK, Unigene13843_YK, Unigene13877_YK, Unigene13978_YK, Unigene13989_YK, Unigene14075_YK, Unigene14094_YK, Unigene14335_YK, Unigene14382_YK, Unigene14408_YK, Unigene14425_YK, Unigene14431_YK, Unigene14456_YK, Unigene14516_YK, Unigene14681_YK, Unigene14805_YK, Unigene14844_YK, Unigene14923_YK, Unigene14969_YK, Unigene14980_YK, Unigene15000_YK, Unigene15016_YK, Unigene15055_YK, Unigene15072_YK, Unigene15079_YK, Unigene15111_YK, Unigene15277_YK, Unigene15325_YK, Unigene15497_YK, Unigene15505_YK, Unigene15561_YK, Unigene15582_YK, Unigene15780_YK, Unigene15795_YK, Unigene15910_YK, Unigene1611_YK, Unigene16406_YK, Unigene17790_YK, Unigene17893_YK, Unigene17894_YK, Unigene18557_YK, Unigene18574_YK, Unigene18819_YK, Unigene18931_YK, Unigene2231_YK, Unigene2289_YK, Unigene24_YK, Unigene258_YK, Unigene26_YK, Unigene2736_YK, Unigene2759_YK, Unigene2791_YK, Unigene2794_YK, Unigene2863_YK, Unigene3032_YK, Unigene317_YK, Unigene3318_YK, Unigene331_YK, Unigene3321_YK, Unigene3373_YK, Unigene3439_YK, Unigene3458_YK, Unigene3483_YK, Unigene3487_YK, Unigene3692_YK, Unigene3764_YK, Unigene3793_YK, Unigene3794_YK, Unigene3817_YK, Unigene3845_YK, Unigene3846_YK, Unigene4038_YK, Unigene4062_YK, Unigene4086_YK, Unigene4213_YK, Unigene4286_YK, Unigene4308_YK, Unigene4436_YK, Unigene4680_YK, Unigene4684_YK, Unigene4_YK, Unigene5296_YK, Unigene5356_YK, Unigene5519_YK, Unigene5656_YK, Unigene5916_YK, Unigene5941_YK, Unigene5993_YK, Unigene6027_YK, Unigene6229_YK, Unigene6230_YK, Unigene6231_YK, Unigene6318_YK, Unigene6380_YK, Unigene6426_YK, Unigene6535_YK, Unigene6561_YK, Unigene6592_YK, Unigene6664_YK, Unigene6729_YK, Unigene7152_YK, Unigene7330_YK, Unigene7382_YK, Unigene7458_YK, Unigene7745_YK, Unigene7816_YK, Unigene8259_YK, Unigene8323_YK, Unigene8324_YK, Unigene8325_YK, Unigene8378_YK, Unigene8909_YK, Unigene9071_YK, Unigene9127_YK, Unigene9232_YK, Unigene9233_YK, Unigene9400_YK, Unigene9517_YK, Unigene9642_YK, Unigene9710_YK, Unigene9968_YK, Unigene9969_YK |
| 14 | [Tight junction](../../../../D:%5C高通量测序结果%5CF14FTSSCKF1242_NEMpnqE%5Cannotation%5CKEGG%5CYK-Unigene.fa_map%5Cmap04530.html) | CL1019.Contig1_YK, CL1019.Contig2_YK, CL1019.Contig3_YK, CL1019.Contig4_YK, CL1156.Contig1_YK, CL1156.Contig2_YK, CL1156.Contig3_YK, CL1219.Contig1_YK, CL1221.Contig1_YK, CL1221.Contig2_YK, CL1221.Contig3_YK, CL1223.Contig1_YK, CL1223.Contig3_YK, CL124.Contig1_YK, CL124.Contig2_YK, CL124.Contig3_YK, CL1303.Contig1_YK, CL1303.Contig2_YK, CL1303.Contig3_YK, CL1486.Contig2_YK, CL1577.Contig1_YK, CL1577.Contig2_YK, CL1577.Contig3_YK, CL1577.Contig4_YK, CL1578.Contig2_YK, CL1591.Contig1_YK, CL1620.Contig1_YK, CL1620.Contig2_YK, CL1750.Contig1_YK, CL1750.Contig2_YK, CL1790.Contig1_YK, CL1790.Contig2_YK, CL1790.Contig3_YK, CL1790.Contig4_YK, CL181.Contig1_YK, CL181.Contig2_YK, CL1816.Contig1_YK, CL1816.Contig2_YK, CL1831.Contig2_YK, CL1881.Contig1_YK, CL1881.Contig2_YK, CL1920.Contig1_YK, CL1920.Contig2_YK, CL1949.Contig1_YK, CL1949.Contig2_YK, CL2006.Contig1_YK, CL2006.Contig2_YK, CL2006.Contig3_YK, CL2367.Contig1_YK, CL2367.Contig2_YK, CL2428.Contig1_YK, CL2428.Contig2_YK, CL2438.Contig1_YK, CL2438.Contig2_YK, CL2493.Contig1_YK, CL2505.Contig2_YK, CL2553.Contig1_YK, CL2553.Contig2_YK, CL2560.Contig1_YK, CL2560.Contig2_YK, CL2648.Contig1_YK, CL2669.Contig1_YK, CL2669.Contig2_YK, CL2669.Contig3_YK, CL2669.Contig4_YK, CL2669.Contig5_YK, CL2713.Contig1_YK, CL2713.Contig2_YK, CL2774.Contig2_YK, CL2816.Contig1_YK, CL2816.Contig2_YK, CL2905.Contig1_YK, CL2905.Contig2_YK, CL300.Contig1_YK, CL300.Contig2_YK, CL344.Contig3_YK, CL36.Contig1_YK, CL36.Contig2_YK, CL369.Contig2_YK, CL369.Contig5_YK, CL369.Contig8_YK, CL37.Contig2_YK, CL415.Contig1_YK, CL415.Contig2_YK, CL415.Contig3_YK, CL415.Contig4_YK, CL439.Contig11_YK, CL487.Contig1_YK, CL487.Contig2_YK, CL527.Contig2_YK, CL527.Contig3_YK, CL528.Contig3_YK, CL528.Contig4_YK, CL589.Contig2_YK, CL589.Contig3_YK, CL591.Contig1_YK, CL591.Contig2_YK, CL591.Contig3_YK, CL591.Contig4_YK, CL591.Contig5_YK, CL591.Contig6_YK, CL591.Contig7_YK, CL591.Contig8_YK, CL599.Contig1_YK, CL599.Contig2_YK, CL606.Contig1_YK, CL606.Contig2_YK, CL606.Contig3_YK, CL627.Contig1_YK, CL627.Contig2_YK, CL84.Contig2_YK, CL84.Contig3_YK, CL884.Contig1_YK, CL884.Contig3_YK, CL91.Contig1_YK, CL91.Contig2_YK, CL91.Contig3_YK, CL91.Contig4_YK, CL91.Contig5_YK, CL91.Contig6_YK, CL91.Contig7_YK, CL95.Contig2_YK, Unigene10136_YK, Unigene10251_YK, Unigene10291_YK, Unigene10355_YK, Unigene10574_YK, Unigene10575_YK, Unigene10576_YK, Unigene10695_YK, Unigene10927_YK, Unigene10954_YK, Unigene11026_YK, Unigene11335_YK, Unigene11532_YK, Unigene11884_YK, Unigene11958_YK, Unigene12027_YK, Unigene12218_YK, Unigene12334_YK, Unigene12335_YK, Unigene12336_YK, Unigene12337_YK, Unigene12426_YK, Unigene12438_YK, Unigene12491_YK, Unigene12492_YK, Unigene12493_YK, Unigene12494_YK, Unigene12525_YK, Unigene12526_YK, Unigene12528_YK, Unigene12532_YK, Unigene12898_YK, Unigene12899_YK, Unigene12979_YK, Unigene13069_YK, Unigene13245_YK, Unigene13250_YK, Unigene13264_YK, Unigene13313_YK, Unigene13598_YK, Unigene13615_YK, Unigene13658_YK, Unigene13704_YK, Unigene13720_YK, Unigene13723_YK, Unigene13743_YK, Unigene13811_YK, Unigene13890_YK, Unigene13908_YK, Unigene13920_YK, Unigene13969_YK, Unigene13985_YK, Unigene14005_YK, Unigene14016_YK, Unigene14081_YK, Unigene14254_YK, Unigene14327_YK, Unigene14382_YK, Unigene14403_YK, Unigene14420_YK, Unigene14661_YK, Unigene14729_YK, Unigene14735_YK, Unigene14778_YK, Unigene14805_YK, Unigene14825_YK, Unigene14838_YK, Unigene14929_YK, Unigene14966_YK, Unigene15034_YK, Unigene15247_YK, Unigene15271_YK, Unigene15308_YK, Unigene15315_YK, Unigene15325_YK, Unigene15580_YK, Unigene15599_YK, Unigene15629_YK, Unigene15743_YK, Unigene15910_YK, Unigene15961_YK, Unigene16018_YK, Unigene16105_YK, Unigene16458_YK, Unigene16741_YK, Unigene16862_YK, Unigene16869_YK, Unigene17458_YK, Unigene1779_YK, Unigene18164_YK, Unigene18316_YK, Unigene19044_YK, Unigene2116_YK, Unigene2207_YK, Unigene256_YK, Unigene2620_YK, Unigene2719_YK, Unigene2849_YK, Unigene2892_YK, Unigene3026_YK, Unigene3034_YK, Unigene3040_YK, Unigene3067_YK, Unigene3188_YK, Unigene3189_YK, Unigene3268_YK, Unigene3271_YK, Unigene3318_YK, Unigene3357_YK, Unigene3445_YK, Unigene3446_YK, Unigene3537_YK, Unigene3629_YK, Unigene3712_YK, Unigene3739_YK, Unigene3740_YK, Unigene3906_YK, Unigene3916_YK, Unigene3937_YK, Unigene4051_YK, Unigene4074_YK, Unigene4179_YK, Unigene4203_YK, Unigene4315_YK, Unigene4320_YK, Unigene4494_YK, Unigene4680_YK, Unigene4747_YK, Unigene4825_YK, Unigene5438_YK, Unigene5462_YK, Unigene5835_YK, Unigene5836_YK, Unigene5979_YK, Unigene6146_YK, Unigene6229_YK, Unigene6230_YK, Unigene6231_YK, Unigene6407_YK, Unigene6469_YK, Unigene6540_YK, Unigene6561_YK, Unigene6600_YK, Unigene6814_YK, Unigene6850_YK, Unigene6879_YK, Unigene6880_YK, Unigene7153_YK, Unigene7270_YK, Unigene7330_YK, Unigene7525_YK, Unigene7662_YK, Unigene7684_YK, Unigene7691_YK, Unigene836_YK, Unigene8500_YK, Unigene8501_YK, Unigene8755_YK, Unigene8892_YK, Unigene8893_YK, Unigene8997_YK, Unigene9323_YK, Unigene9373_YK, Unigene9388_YK, Unigene9937_YK, Unigene9989_YK |
| 15 | [Neuroactive ligand-receptor interaction](../../../../D:%5C高通量测序结果%5CF14FTSSCKF1242_NEMpnqE%5Cannotation%5CKEGG%5CYK-Unigene.fa_map%5Cmap04080.html) | CL10.Contig1_YK, CL10.Contig2_YK, CL10.Contig3_YK, CL10.Contig4_YK, CL1016.Contig1_YK, CL1016.Contig2_YK, CL1053.Contig2_YK, CL1117.Contig1_YK, CL1150.Contig1_YK, CL1150.Contig2_YK, CL1205.Contig1_YK, CL1205.Contig2_YK, CL1205.Contig3_YK, CL1205.Contig4_YK, CL1242.Contig1_YK, CL1242.Contig2_YK, CL1487.Contig1_YK, CL155.Contig1_YK, CL155.Contig2_YK, CL1593.Contig1_YK, CL1593.Contig2_YK, CL1612.Contig1_YK, CL1612.Contig2_YK, CL1617.Contig1_YK, CL1617.Contig2_YK, CL1617.Contig3_YK, CL1649.Contig1_YK, CL1649.Contig2_YK, CL1659.Contig1_YK, CL1659.Contig2_YK, CL174.Contig1_YK, CL1767.Contig1_YK, CL1767.Contig2_YK, CL1767.Contig3_YK, CL1767.Contig4_YK, CL1790.Contig1_YK, CL1790.Contig2_YK, CL1790.Contig3_YK, CL1790.Contig4_YK, CL1810.Contig1_YK, CL1810.Contig2_YK, CL1838.Contig1_YK, CL1838.Contig2_YK, CL1856.Contig1_YK, CL1856.Contig2_YK, CL1919.Contig1_YK, CL1919.Contig2_YK, CL1990.Contig1_YK, CL1990.Contig2_YK, CL2032.Contig1_YK, CL2032.Contig2_YK, CL2066.Contig1_YK, CL2066.Contig2_YK, CL2068.Contig1_YK, CL2068.Contig2_YK, CL209.Contig1_YK, CL209.Contig2_YK, CL2117.Contig1_YK, CL2117.Contig2_YK, CL2240.Contig1_YK, CL2240.Contig2_YK, CL2337.Contig1_YK, CL2337.Contig2_YK, CL2407.Contig1_YK, CL2441.Contig1_YK, CL2441.Contig2_YK, CL2491.Contig1_YK, CL2622.Contig1_YK, CL2622.Contig2_YK, CL321.Contig1_YK, CL383.Contig17_YK, CL383.Contig18_YK, CL422.Contig3_YK, CL745.Contig1_YK, CL745.Contig2_YK, CL763.Contig1_YK, CL763.Contig2_YK, CL763.Contig3_YK, CL763.Contig4_YK, CL763.Contig5_YK, CL763.Contig6_YK, CL816.Contig1_YK, CL909.Contig2_YK, CL928.Contig1_YK, CL928.Contig2_YK, CL93.Contig1_YK, CL961.Contig4_YK, CL961.Contig5_YK, CL961.Contig6_YK, CL961.Contig8_YK, CL998.Contig1_YK, CL998.Contig3_YK, Unigene10000_YK, Unigene10020_YK, Unigene103_YK, Unigene10491_YK, Unigene10492_YK, Unigene10493_YK, Unigene10506_YK, Unigene10507_YK, Unigene10534_YK, Unigene10563_YK, Unigene10614_YK, Unigene10676_YK, Unigene10766_YK, Unigene10767_YK, Unigene10781_YK, Unigene10790_YK, Unigene10822_YK, Unigene10829_YK, Unigene1111_YK, Unigene11551_YK, Unigene11836_YK, Unigene12351_YK, Unigene12777_YK, Unigene12866_YK, Unigene12876_YK, Unigene12887_YK, Unigene13015_YK, Unigene1356_YK, Unigene1401_YK, Unigene1450_YK, Unigene14922_YK, Unigene15450_YK, Unigene15454_YK, Unigene15536_YK, Unigene15561_YK, Unigene15697_YK, Unigene15784_YK, Unigene15787_YK, Unigene157_YK, Unigene15967_YK, Unigene16094_YK, Unigene16102_YK, Unigene16191_YK, Unigene16329_YK, Unigene16420_YK, Unigene16426_YK, Unigene16434_YK, Unigene16439_YK, Unigene16467_YK, Unigene16713_YK, Unigene16820_YK, Unigene16843_YK, Unigene16891_YK, Unigene17056_YK, Unigene17435_YK, Unigene17444_YK, Unigene17452_YK, Unigene17478_YK, Unigene17566_YK, Unigene17689_YK, Unigene17739_YK, Unigene17756_YK, Unigene17811_YK, Unigene1803_YK, Unigene18256_YK, Unigene18448_YK, Unigene18664_YK, Unigene18798_YK, Unigene18816_YK, Unigene1919_YK, Unigene1996_YK, Unigene2075_YK, Unigene2121_YK, Unigene2143_YK, Unigene2182_YK, Unigene2340_YK, Unigene240_YK, Unigene2464_YK, Unigene2582_YK, Unigene2583_YK, Unigene2630_YK, Unigene2657_YK, Unigene2720_YK, Unigene304_YK, Unigene319_YK, Unigene352_YK, Unigene3585_YK, Unigene3615_YK, Unigene3727_YK, Unigene397_YK, Unigene3989_YK, Unigene4195_YK, Unigene4291_YK, Unigene4351_YK, Unigene4416_YK, Unigene4445_YK, Unigene4498_YK, Unigene4778_YK, Unigene4799_YK, Unigene4827_YK, Unigene4848_YK, Unigene5011_YK, Unigene5042_YK, Unigene5043_YK, Unigene5313_YK, Unigene5330_YK, Unigene5359_YK, Unigene5360_YK, Unigene5448_YK, Unigene5462_YK, Unigene5524_YK, Unigene5662_YK, Unigene6006_YK, Unigene6007_YK, Unigene6045_YK, Unigene6089_YK, Unigene6196_YK, Unigene6198_YK, Unigene6328_YK, Unigene6362_YK, Unigene6691_YK, Unigene675_YK, Unigene6830_YK, Unigene6915_YK, Unigene6916_YK, Unigene6971_YK, Unigene6972_YK, Unigene708_YK, Unigene7142_YK, Unigene7143_YK, Unigene7149_YK, Unigene7235_YK, Unigene735_YK, Unigene7390_YK, Unigene7391_YK, Unigene7393_YK, Unigene7402_YK, Unigene7531_YK, Unigene7724_YK, Unigene7736_YK, Unigene7787_YK, Unigene7816_YK, Unigene7822_YK, Unigene7823_YK, Unigene7824_YK, Unigene7851_YK, Unigene7853_YK, Unigene7858_YK, Unigene7916_YK, Unigene7927_YK, Unigene7929_YK, Unigene8065_YK, Unigene8074_YK, Unigene8130_YK, Unigene8144_YK, Unigene8146_YK, Unigene8300_YK, Unigene8301_YK, Unigene8410_YK, Unigene8462_YK, Unigene8557_YK, Unigene8583_YK, Unigene8745_YK, Unigene8746_YK, Unigene8788_YK, Unigene8815_YK, Unigene8857_YK, Unigene8858_YK, Unigene8859_YK, Unigene8860_YK, Unigene8951_YK, Unigene8952_YK, Unigene8953_YK, Unigene9086_YK, Unigene9266_YK, Unigene9268_YK, Unigene9300_YK, Unigene9573_YK, Unigene9574_YK, Unigene9575_YK, Unigene9581_YK, Unigene9605_YK, Unigene9702_YK, Unigene9816_YK, Unigene9981_YK, Unigene9983_YK, Unigene9994_YK |
| 16 | [Dilated cardiomyopathy](../../../../D:%5C高通量测序结果%5CF14FTSSCKF1242_NEMpnqE%5Cannotation%5CKEGG%5CYK-Unigene.fa_map%5Cmap05414.html) | CL1019.Contig1_YK, CL1019.Contig2_YK, CL1019.Contig3_YK, CL1019.Contig4_YK, CL1088.Contig1_YK, CL1088.Contig2_YK, CL1156.Contig3_YK, CL1221.Contig1_YK, CL1221.Contig2_YK, CL1221.Contig3_YK, CL124.Contig1_YK, CL124.Contig2_YK, CL124.Contig3_YK, CL1260.Contig1_YK, CL1260.Contig2_YK, CL1260.Contig3_YK, CL1303.Contig1_YK, CL1303.Contig2_YK, CL1303.Contig3_YK, CL136.Contig1_YK, CL136.Contig3_YK, CL1486.Contig2_YK, CL1551.Contig1_YK, CL1551.Contig2_YK, CL1577.Contig1_YK, CL1577.Contig2_YK, CL1577.Contig3_YK, CL1577.Contig4_YK, CL1578.Contig2_YK, CL1591.Contig1_YK, CL1638.Contig1_YK, CL1638.Contig2_YK, CL1710.Contig2_YK, CL181.Contig1_YK, CL181.Contig2_YK, CL1891.Contig1_YK, CL1891.Contig2_YK, CL1920.Contig1_YK, CL1920.Contig2_YK, CL1998.Contig1_YK, CL2051.Contig1_YK, CL2051.Contig3_YK, CL2090.Contig1_YK, CL2090.Contig2_YK, CL2090.Contig3_YK, CL2090.Contig4_YK, CL2103.Contig3_YK, CL2426.Contig1_YK, CL255.Contig1_YK, CL255.Contig2_YK, CL255.Contig3_YK, CL255.Contig4_YK, CL255.Contig5_YK, CL255.Contig6_YK, CL255.Contig7_YK, CL255.Contig8_YK, CL2560.Contig1_YK, CL2560.Contig2_YK, CL2648.Contig1_YK, CL2669.Contig1_YK, CL2669.Contig2_YK, CL2669.Contig3_YK, CL2669.Contig4_YK, CL2669.Contig5_YK, CL2774.Contig2_YK, CL2798.Contig1_YK, CL2798.Contig2_YK, CL2816.Contig1_YK, CL2905.Contig1_YK, CL2905.Contig2_YK, CL344.Contig3_YK, CL367.Contig1_YK, CL369.Contig2_YK, CL369.Contig5_YK, CL369.Contig8_YK, CL37.Contig2_YK, CL487.Contig1_YK, CL487.Contig2_YK, CL502.Contig1_YK, CL502.Contig2_YK, CL503.Contig1_YK, CL599.Contig1_YK, CL599.Contig2_YK, CL663.Contig1_YK, CL75.Contig1_YK, CL75.Contig2_YK, CL75.Contig3_YK, CL79.Contig1_YK, CL79.Contig2_YK, CL79.Contig3_YK, CL79.Contig4_YK, CL79.Contig5_YK, CL79.Contig6_YK, CL79.Contig7_YK, CL79.Contig8_YK, CL838.Contig1_YK, CL838.Contig2_YK, CL838.Contig3_YK, CL838.Contig4_YK, CL838.Contig5_YK, CL838.Contig8_YK, CL84.Contig2_YK, CL84.Contig3_YK, CL91.Contig1_YK, CL91.Contig2_YK, CL91.Contig3_YK, CL91.Contig4_YK, CL91.Contig5_YK, CL91.Contig6_YK, CL91.Contig7_YK, CL95.Contig2_YK, CL957.Contig1_YK, CL957.Contig2_YK, Unigene10251_YK, Unigene10291_YK, Unigene10296_YK, Unigene10297_YK, Unigene10298_YK, Unigene10355_YK, Unigene10695_YK, Unigene10908_YK, Unigene10927_YK, Unigene11080_YK, Unigene11081_YK, Unigene11532_YK, Unigene11679_YK, Unigene11802_YK, Unigene11884_YK, Unigene11889_YK, Unigene12027_YK, Unigene12028_YK, Unigene12216_YK, Unigene12219_YK, Unigene1223_YK, Unigene12292_YK, Unigene12293_YK, Unigene12334_YK, Unigene12335_YK, Unigene12336_YK, Unigene12337_YK, Unigene12361_YK, Unigene12426_YK, Unigene12467_YK, Unigene12491_YK, Unigene12492_YK, Unigene12493_YK, Unigene12494_YK, Unigene12532_YK, Unigene12852_YK, Unigene12898_YK, Unigene12899_YK, Unigene13009_YK, Unigene13237_YK, Unigene13245_YK, Unigene13296_YK, Unigene13308_YK, Unigene13598_YK, Unigene13704_YK, Unigene13720_YK, Unigene13811_YK, Unigene13890_YK, Unigene13920_YK, Unigene14005_YK, Unigene14016_YK, Unigene14112_YK, Unigene14254_YK, Unigene14327_YK, Unigene14403_YK, Unigene14420_YK, Unigene14661_YK, Unigene14729_YK, Unigene14929_YK, Unigene14966_YK, Unigene15034_YK, Unigene15271_YK, Unigene15308_YK, Unigene15315_YK, Unigene15768_YK, Unigene15804_YK, Unigene15961_YK, Unigene16000_YK, Unigene16018_YK, Unigene16105_YK, Unigene16406_YK, Unigene16458_YK, Unigene16741_YK, Unigene16862_YK, Unigene16869_YK, Unigene17458_YK, Unigene18183_YK, Unigene18316_YK, Unigene18577_YK, Unigene18657_YK, Unigene18999_YK, Unigene19071_YK, Unigene19153_YK, Unigene2207_YK, Unigene2231_YK, Unigene2289_YK, Unigene258_YK, Unigene2607_YK, Unigene2620_YK, Unigene2719_YK, Unigene2770_YK, Unigene2808_YK, Unigene2849_YK, Unigene2869_YK, Unigene2892_YK, Unigene3026_YK, Unigene3034_YK, Unigene3067_YK, Unigene3188_YK, Unigene3189_YK, Unigene3268_YK, Unigene3271_YK, Unigene331_YK, Unigene3548_YK, Unigene3629_YK, Unigene3650_YK, Unigene3700_YK, Unigene3716_YK, Unigene3739_YK, Unigene3740_YK, Unigene375_YK, Unigene3852_YK, Unigene3906_YK, Unigene3916_YK, Unigene3934_YK, Unigene4055_YK, Unigene4074_YK, Unigene4268_YK, Unigene4315_YK, Unigene4747_YK, Unigene4825_YK, Unigene4900_YK, Unigene4924_YK, Unigene5603_YK, Unigene5874_YK, Unigene6146_YK, Unigene6270_YK, Unigene6469_YK, Unigene6725_YK, Unigene6814_YK, Unigene6850_YK, Unigene6889_YK, Unigene6890_YK, Unigene6980_YK, Unigene7378_YK, Unigene7483_YK, Unigene7610_YK, Unigene7611_YK, Unigene7662_YK, Unigene7684_YK, Unigene8101_YK, Unigene836_YK, Unigene8371_YK, Unigene8396_YK, Unigene8577_YK, Unigene8578_YK, Unigene8755_YK, Unigene8762_YK, Unigene8874_YK, Unigene8875_YK, Unigene8876_YK, Unigene8997_YK, Unigene9029_YK, Unigene904_YK, Unigene905_YK, Unigene9373_YK, Unigene9447_YK, Unigene9449_YK, Unigene9451_YK, Unigene9634_YK, Unigene9710_YK, Unigene9832_YK, Unigene9937_YK, Unigene9989_YK |
| 17 | [Purine metabolism](../../../../D:%5C高通量测序结果%5CF14FTSSCKF1242_NEMpnqE%5Cannotation%5CKEGG%5CYK-Unigene.fa_map%5Cmap00230.html) | CL1058.Contig4_YK, CL1083.Contig1_YK, CL1083.Contig2_YK, CL1083.Contig3_YK, CL1083.Contig4_YK, CL1178.Contig1_YK, CL1178.Contig2_YK, CL122.Contig2_YK, CL1293.Contig1_YK, CL1293.Contig2_YK, CL1351.Contig1_YK, CL1351.Contig2_YK, CL1441.Contig1_YK, CL1441.Contig2_YK, CL1441.Contig3_YK, CL1441.Contig4_YK, CL1528.Contig1_YK, CL1528.Contig2_YK, CL1560.Contig1_YK, CL1560.Contig2_YK, CL1560.Contig3_YK, CL1569.Contig1_YK, CL1569.Contig2_YK, CL1689.Contig2_YK, CL1786.Contig2_YK, CL1786.Contig3_YK, CL1841.Contig1_YK, CL1841.Contig2_YK, CL2004.Contig1_YK, CL2004.Contig2_YK, CL2042.Contig1_YK, CL2153.Contig1_YK, CL2153.Contig2_YK, CL2153.Contig3_YK, CL2153.Contig4_YK, CL2301.Contig1_YK, CL2301.Contig2_YK, CL2361.Contig1_YK, CL2361.Contig2_YK, CL2471.Contig1_YK, CL2471.Contig2_YK, CL2471.Contig3_YK, CL2471.Contig4_YK, CL249.Contig1_YK, CL2493.Contig1_YK, CL2594.Contig1_YK, CL2594.Contig2_YK, CL2594.Contig3_YK, CL2594.Contig4_YK, CL2637.Contig1_YK, CL2637.Contig2_YK, CL2807.Contig1_YK, CL2807.Contig2_YK, CL2840.Contig1_YK, CL2840.Contig2_YK, CL32.Contig1_YK, CL32.Contig2_YK, CL341.Contig1_YK, CL341.Contig2_YK, CL345.Contig1_YK, CL345.Contig2_YK, CL347.Contig1_YK, CL376.Contig1_YK, CL376.Contig2_YK, CL376.Contig3_YK, CL376.Contig4_YK, CL376.Contig5_YK, CL376.Contig6_YK, CL477.Contig3_YK, CL527.Contig10_YK, CL527.Contig1_YK, CL527.Contig4_YK, CL527.Contig5_YK, CL527.Contig6_YK, CL527.Contig7_YK, CL527.Contig8_YK, CL527.Contig9_YK, CL552.Contig1_YK, CL569.Contig1_YK, CL569.Contig2_YK, CL63.Contig1_YK, CL63.Contig2_YK, CL646.Contig1_YK, CL646.Contig2_YK, CL711.Contig1_YK, CL711.Contig2_YK, CL761.Contig1_YK, CL761.Contig2_YK, CL761.Contig3_YK, CL761.Contig4_YK, CL761.Contig5_YK, CL761.Contig6_YK, CL79.Contig1_YK, CL79.Contig2_YK, CL79.Contig3_YK, CL79.Contig4_YK, CL79.Contig5_YK, CL79.Contig6_YK, CL79.Contig7_YK, CL79.Contig8_YK, Unigene10178_YK, Unigene10194_YK, Unigene10410_YK, Unigene10665_YK, Unigene10908_YK, Unigene11404_YK, Unigene11701_YK, Unigene11728_YK, Unigene11814_YK, Unigene11926_YK, Unigene11927_YK, Unigene11966_YK, Unigene11967_YK, Unigene12177_YK, Unigene12178_YK, Unigene12179_YK, Unigene12180_YK, Unigene1224_YK, Unigene12292_YK, Unigene12293_YK, Unigene12930_YK, Unigene12982_YK, Unigene13071_YK, Unigene13076_YK, Unigene13101_YK, Unigene13246_YK, Unigene13255_YK, Unigene13282_YK, Unigene13338_YK, Unigene13480_YK, Unigene13494_YK, Unigene13565_YK, Unigene13696_YK, Unigene1377_YK, Unigene13818_YK, Unigene13841_YK, Unigene13873_YK, Unigene13895_YK, Unigene13979_YK, Unigene14004_YK, Unigene14054_YK, Unigene14075_YK, Unigene14103_YK, Unigene14196_YK, Unigene14199_YK, Unigene14264_YK, Unigene14368_YK, Unigene14497_YK, Unigene14606_YK, Unigene14610_YK, Unigene14733_YK, Unigene14744_YK, Unigene14789_YK, Unigene14876_YK, Unigene14885_YK, Unigene1488_YK, Unigene14923_YK, Unigene14925_YK, Unigene14927_YK, Unigene14991_YK, Unigene14994_YK, Unigene15079_YK, Unigene15184_YK, Unigene15220_YK, Unigene15246_YK, Unigene15345_YK, Unigene15478_YK, Unigene15575_YK, Unigene15718_YK, Unigene15726_YK, Unigene15729_YK, Unigene15974_YK, Unigene15979_YK, Unigene16066_YK, Unigene16145_YK, Unigene16177_YK, Unigene16211_YK, Unigene16273_YK, Unigene16299_YK, Unigene16358_YK, Unigene16406_YK, Unigene16529_YK, Unigene16653_YK, Unigene17035_YK, Unigene17665_YK, Unigene1779_YK, Unigene17932_YK, Unigene179_YK, Unigene18024_YK, Unigene18033_YK, Unigene18088_YK, Unigene18791_YK, Unigene18899_YK, Unigene1895_YK, Unigene19002_YK, Unigene1932_YK, Unigene1963_YK, Unigene2129_YK, Unigene2189_YK, Unigene2211_YK, Unigene2231_YK, Unigene2267_YK, Unigene2289_YK, Unigene2474_YK, Unigene258_YK, Unigene2628_YK, Unigene2629_YK, Unigene2772_YK, Unigene2794_YK, Unigene2926_YK, Unigene2931_YK, Unigene3031_YK, Unigene3049_YK, Unigene3060_YK, Unigene3314_YK, Unigene3486_YK, Unigene3793_YK, Unigene3794_YK, Unigene3845_YK, Unigene3846_YK, Unigene3934_YK, Unigene3935_YK, Unigene3959_YK, Unigene4002_YK, Unigene4004_YK, Unigene4040_YK, Unigene4041_YK, Unigene4103_YK, Unigene4496_YK, Unigene4497_YK, Unigene4550_YK, Unigene456_YK, Unigene457_YK, Unigene4775_YK, Unigene4935_YK, Unigene4936_YK, Unigene5136_YK, Unigene516_YK, Unigene5196_YK, Unigene5365_YK, Unigene5447_YK, Unigene548_YK, Unigene5512_YK, Unigene5577_YK, Unigene5597_YK, Unigene561_YK, Unigene5670_YK, Unigene5754_YK, Unigene5794_YK, Unigene5817_YK, Unigene5818_YK, Unigene5935_YK, Unigene6070_YK, Unigene6143_YK, Unigene6508_YK, Unigene6582_YK, Unigene6583_YK, Unigene6592_YK, Unigene6819_YK, Unigene6895_YK, Unigene7337_YK, Unigene7669_YK, Unigene7690_YK, Unigene771_YK, Unigene8003_YK, Unigene8385_YK, Unigene8477_YK, Unigene8999_YK, Unigene9150_YK, Unigene9198_YK, Unigene9269_YK, Unigene9411_YK, Unigene9412_YK, Unigene9517_YK, Unigene9710_YK, Unigene9985_YK |
| 18 | [Tuberculosis](../../../../D:%5C高通量测序结果%5CF14FTSSCKF1242_NEMpnqE%5Cannotation%5CKEGG%5CYK-Unigene.fa_map%5Cmap05152.html) | CL1002.Contig1_YK, CL1002.Contig2_YK, CL112.Contig1_YK, CL112.Contig2_YK, CL112.Contig3_YK, CL112.Contig4_YK, CL112.Contig5_YK, CL112.Contig6_YK, CL112.Contig7_YK, CL112.Contig8_YK, CL1286.Contig1_YK, CL1286.Contig2_YK, CL1286.Contig3_YK, CL1286.Contig4_YK, CL1286.Contig5_YK, CL1286.Contig6_YK, CL1286.Contig7_YK, CL1286.Contig8_YK, CL1310.Contig3_YK, CL1310.Contig5_YK, CL1399.Contig4_YK, CL1399.Contig5_YK, CL1420.Contig1_YK, CL1510.Contig1_YK, CL1510.Contig3_YK, CL1599.Contig1_YK, CL1599.Contig2_YK, CL1599.Contig3_YK, CL1714.Contig2_YK, CL173.Contig1_YK, CL173.Contig2_YK, CL1793.Contig3_YK, CL1801.Contig1_YK, CL1801.Contig2_YK, CL1816.Contig1_YK, CL1816.Contig2_YK, CL1826.Contig1_YK, CL1826.Contig2_YK, CL1844.Contig1_YK, CL1844.Contig2_YK, CL1861.Contig1_YK, CL1936.Contig1_YK, CL1960.Contig1_YK, CL1960.Contig2_YK, CL2006.Contig1_YK, CL2006.Contig2_YK, CL2006.Contig3_YK, CL2115.Contig1_YK, CL2115.Contig2_YK, CL2115.Contig3_YK, CL2135.Contig1_YK, CL2135.Contig2_YK, CL2168.Contig1_YK, CL2294.Contig1_YK, CL2294.Contig2_YK, CL2336.Contig1_YK, CL2336.Contig2_YK, CL2382.Contig1_YK, CL2448.Contig1_YK, CL2448.Contig2_YK, CL2449.Contig1_YK, CL2449.Contig2_YK, CL2452.Contig1_YK, CL2452.Contig2_YK, CL2505.Contig1_YK, CL2505.Contig2_YK, CL2537.Contig1_YK, CL2537.Contig2_YK, CL2593.Contig1_YK, CL2593.Contig2_YK, CL2593.Contig3_YK, CL2624.Contig1_YK, CL2624.Contig2_YK, CL2635.Contig1_YK, CL2635.Contig2_YK, CL2677.Contig1_YK, CL2742.Contig1_YK, CL35.Contig1_YK, CL35.Contig2_YK, CL353.Contig1_YK, CL353.Contig3_YK, CL353.Contig6_YK, CL387.Contig1_YK, CL387.Contig2_YK, CL387.Contig3_YK, CL387.Contig4_YK, CL387.Contig5_YK, CL431.Contig1_YK, CL431.Contig2_YK, CL431.Contig3_YK, CL513.Contig1_YK, CL513.Contig2_YK, CL513.Contig3_YK, CL513.Contig4_YK, CL513.Contig5_YK, CL568.Contig1_YK, CL568.Contig2_YK, CL707.Contig1_YK, CL707.Contig2_YK, CL742.Contig3_YK, CL742.Contig4_YK, CL809.Contig1_YK, CL809.Contig2_YK, CL827.Contig1_YK, CL827.Contig2_YK, CL827.Contig3_YK, CL83.Contig4_YK, CL878.Contig1_YK, CL878.Contig2_YK, CL878.Contig3_YK, CL902.Contig1_YK, CL902.Contig2_YK, Unigene10096_YK, Unigene10213_YK, Unigene10224_YK, Unigene10470_YK, Unigene10546_YK, Unigene10547_YK, Unigene10604_YK, Unigene10659_YK, Unigene10694_YK, Unigene10775_YK, Unigene10927_YK, Unigene11097_YK, Unigene11098_YK, Unigene1123_YK, Unigene11246_YK, Unigene11251_YK, Unigene11332_YK, Unigene11335_YK, Unigene11439_YK, Unigene11451_YK, Unigene1150_YK, Unigene11561_YK, Unigene12218_YK, Unigene12247_YK, Unigene12695_YK, Unigene12743_YK, Unigene12987_YK, Unigene13026_YK, Unigene13061_YK, Unigene13103_YK, Unigene13104_YK, Unigene13137_YK, Unigene13161_YK, Unigene13213_YK, Unigene13232_YK, Unigene13274_YK, Unigene13527_YK, Unigene13550_YK, Unigene13572_YK, Unigene13674_YK, Unigene13729_YK, Unigene13877_YK, Unigene138_YK, Unigene13937_YK, Unigene13960_YK, Unigene13985_YK, Unigene14016_YK, Unigene14019_YK, Unigene14094_YK, Unigene14220_YK, Unigene14243_YK, Unigene14327_YK, Unigene14381_YK, Unigene14516_YK, Unigene14559_YK, Unigene14661_YK, Unigene14729_YK, Unigene15028_YK, Unigene15057_YK, Unigene15072_YK, Unigene15076_YK, Unigene15080_YK, Unigene15246_YK, Unigene15365_YK, Unigene15381_YK, Unigene15461_YK, Unigene15587_YK, Unigene15737_YK, Unigene15898_YK, Unigene16015_YK, Unigene16411_YK, Unigene16527_YK, Unigene16758_YK, Unigene16861_YK, Unigene16869_YK, Unigene16914_YK, Unigene16947_YK, Unigene17195_YK, Unigene17867_YK, Unigene18027_YK, Unigene18183_YK, Unigene182_YK, Unigene18577_YK, Unigene18679_YK, Unigene18722_YK, Unigene2137_YK, Unigene2197_YK, Unigene2221_YK, Unigene2250_YK, Unigene2276_YK, Unigene2295_YK, Unigene2296_YK, Unigene2407_YK, Unigene2457_YK, Unigene2471_YK, Unigene2550_YK, Unigene2899_YK, Unigene3119_YK, Unigene3145_YK, Unigene333_YK, Unigene3381_YK, Unigene3626_YK, Unigene3761_YK, Unigene3769_YK, Unigene382_YK, Unigene4062_YK, Unigene4100_YK, Unigene4108_YK, Unigene4213_YK, Unigene4380_YK, Unigene4471_YK, Unigene4494_YK, Unigene4532_YK, Unigene4574_YK, Unigene4825_YK, Unigene5102_YK, Unigene5586_YK, Unigene5680_YK, Unigene5737_YK, Unigene5763_YK, Unigene5819_YK, Unigene6112_YK, Unigene6166_YK, Unigene6227_YK, Unigene6229_YK, Unigene6230_YK, Unigene6231_YK, Unigene625_YK, Unigene6321_YK, Unigene6322_YK, Unigene6407_YK, Unigene6450_YK, Unigene6454_YK, Unigene6461_YK, Unigene6596_YK, Unigene6611_YK, Unigene6960_YK, Unigene6_YK, Unigene7100_YK, Unigene7101_YK, Unigene7176_YK, Unigene7281_YK, Unigene7307_YK, Unigene7308_YK, Unigene7362_YK, Unigene7382_YK, Unigene7511_YK, Unigene7590_YK, Unigene7610_YK, Unigene7611_YK, Unigene7755_YK, Unigene8279_YK, Unigene8508_YK, Unigene8657_YK, Unigene9071_YK, Unigene9127_YK, Unigene91_YK, Unigene9323_YK, Unigene9388_YK, Unigene9395_YK, Unigene9429_YK, Unigene9459_YK, Unigene957_YK, Unigene9616_YK |
| 19 | [Huntington's disease](../../../../D:%5C高通量测序结果%5CF14FTSSCKF1242_NEMpnqE%5Cannotation%5CKEGG%5CYK-Unigene.fa_map%5Cmap05016.html) | CL1058.Contig4_YK, CL1103.Contig1_YK, CL1228.Contig1_YK, CL1247.Contig1_YK, CL1247.Contig2_YK, CL1288.Contig1_YK, CL1328.Contig1_YK, CL15.Contig10_YK, CL15.Contig11_YK, CL15.Contig12_YK, CL15.Contig13_YK, CL15.Contig14_YK, CL15.Contig15_YK, CL15.Contig16_YK, CL15.Contig17_YK, CL15.Contig18_YK, CL15.Contig19_YK, CL15.Contig1_YK, CL15.Contig20_YK, CL15.Contig21_YK, CL15.Contig22_YK, CL15.Contig23_YK, CL15.Contig24_YK, CL15.Contig2_YK, CL15.Contig3_YK, CL15.Contig4_YK, CL15.Contig5_YK, CL15.Contig6_YK, CL15.Contig7_YK, CL15.Contig8_YK, CL15.Contig9_YK, CL1514.Contig1_YK, CL1514.Contig2_YK, CL1544.Contig1_YK, CL1544.Contig2_YK, CL1560.Contig1_YK, CL1560.Contig2_YK, CL1560.Contig3_YK, CL1611.Contig1_YK, CL1611.Contig2_YK, CL1682.Contig1_YK, CL2024.Contig1_YK, CL204.Contig1_YK, CL204.Contig2_YK, CL2240.Contig1_YK, CL2240.Contig2_YK, CL2294.Contig1_YK, CL2294.Contig2_YK, CL2336.Contig1_YK, CL2336.Contig2_YK, CL2424.Contig1_YK, CL2424.Contig2_YK, CL2626.Contig1_YK, CL2626.Contig2_YK, CL2637.Contig1_YK, CL2637.Contig2_YK, CL295.Contig10_YK, CL295.Contig2_YK, CL295.Contig4_YK, CL295.Contig6_YK, CL307.Contig10_YK, CL307.Contig11_YK, CL307.Contig12_YK, CL307.Contig1_YK, CL307.Contig2_YK, CL307.Contig3_YK, CL307.Contig4_YK, CL307.Contig5_YK, CL307.Contig6_YK, CL307.Contig7_YK, CL307.Contig8_YK, CL307.Contig9_YK, CL317.Contig1_YK, CL317.Contig2_YK, CL35.Contig1_YK, CL35.Contig2_YK, CL369.Contig5_YK, CL369.Contig6_YK, CL369.Contig8_YK, CL387.Contig1_YK, CL387.Contig2_YK, CL387.Contig3_YK, CL387.Contig4_YK, CL387.Contig5_YK, CL564.Contig10_YK, CL564.Contig13_YK, CL564.Contig14_YK, CL564.Contig16_YK, CL564.Contig2_YK, CL564.Contig3_YK, CL564.Contig5_YK, CL564.Contig6_YK, CL564.Contig7_YK, CL564.Contig8_YK, CL564.Contig9_YK, CL569.Contig1_YK, CL569.Contig2_YK, CL83.Contig4_YK, CL940.Contig2_YK, Unigene10273_YK, Unigene10275_YK, Unigene10409_YK, Unigene10493_YK, Unigene10614_YK, Unigene10659_YK, Unigene10665_YK, Unigene10677_YK, Unigene10678_YK, Unigene10777_YK, Unigene10829_YK, Unigene10895_YK, Unigene11072_YK, Unigene11740_YK, Unigene12025_YK, Unigene12133_YK, Unigene12259_YK, Unigene12376_YK, Unigene12560_YK, Unigene12561_YK, Unigene12563_YK, Unigene12564_YK, Unigene12615_YK, Unigene12636_YK, Unigene12638_YK, Unigene12639_YK, Unigene12743_YK, Unigene12881_YK, Unigene12882_YK, Unigene12982_YK, Unigene12990_YK, Unigene13063_YK, Unigene13065_YK, Unigene13076_YK, Unigene13166_YK, Unigene13173_YK, Unigene13192_YK, Unigene13221_YK, Unigene13229_YK, Unigene13231_YK, Unigene13234_YK, Unigene13282_YK, Unigene13375_YK, Unigene13401_YK, Unigene13430_YK, Unigene13454_YK, Unigene13473_YK, Unigene13483_YK, Unigene13486_YK, Unigene13507_YK, Unigene1356_YK, Unigene13584_YK, Unigene13592_YK, Unigene13607_YK, Unigene13608_YK, Unigene13639_YK, Unigene13643_YK, Unigene13696_YK, Unigene13708_YK, Unigene13785_YK, Unigene13801_YK, Unigene13829_YK, Unigene13841_YK, Unigene13847_YK, Unigene13878_YK, Unigene139_YK, Unigene14028_YK, Unigene14030_YK, Unigene14052_YK, Unigene14053_YK, Unigene14054_YK, Unigene14094_YK, Unigene14126_YK, Unigene14165_YK, Unigene14204_YK, Unigene14283_YK, Unigene14309_YK, Unigene14374_YK, Unigene14440_YK, Unigene14455_YK, Unigene14482_YK, Unigene14510_YK, Unigene14516_YK, Unigene14527_YK, Unigene14542_YK, Unigene14589_YK, Unigene14789_YK, Unigene14927_YK, Unigene14977_YK, Unigene14991_YK, Unigene15028_YK, Unigene15040_YK, Unigene15271_YK, Unigene15277_YK, Unigene15396_YK, Unigene15523_YK, Unigene15529_YK, Unigene15729_YK, Unigene15764_YK, Unigene15877_YK, Unigene158_YK, Unigene16177_YK, Unigene16337_YK, Unigene1730_YK, Unigene17811_YK, Unigene179_YK, Unigene18156_YK, Unigene18679_YK, Unigene19067_YK, Unigene19105_YK, Unigene19109_YK, Unigene1964_YK, Unigene2785_YK, Unigene2900_YK, Unigene3031_YK, Unigene3034_YK, Unigene3037_YK, Unigene3049_YK, Unigene3090_YK, Unigene3133_YK, Unigene3140_YK, Unigene319_YK, Unigene3219_YK, Unigene3362_YK, Unigene3363_YK, Unigene3397_YK, Unigene3416_YK, Unigene3585_YK, Unigene3868_YK, Unigene4033_YK, Unigene4099_YK, Unigene4252_YK, Unigene4435_YK, Unigene4445_YK, Unigene4454_YK, Unigene4563_YK, Unigene4631_YK, Unigene4778_YK, Unigene5018_YK, Unigene5019_YK, Unigene5136_YK, Unigene5196_YK, Unigene5322_YK, Unigene5412_YK, Unigene5476_YK, Unigene5477_YK, Unigene5511_YK, Unigene5832_YK, Unigene5857_YK, Unigene5916_YK, Unigene5961_YK, Unigene5975_YK, Unigene6006_YK, Unigene6007_YK, Unigene6206_YK, Unigene6247_YK, Unigene6406_YK, Unigene6508_YK, Unigene663_YK, Unigene6789_YK, Unigene6895_YK, Unigene7152_YK, Unigene7390_YK, Unigene7661_YK, Unigene7669_YK, Unigene8776_YK, Unigene8777_YK, Unigene8822_YK, Unigene9127_YK, Unigene9735_YK, Unigene9736_YK, Unigene9903_YK, Unigene9904_YK, Unigene9985_YK |
| 20 | [Bile secretion](../../../../D:%5C高通量测序结果%5CF14FTSSCKF1242_NEMpnqE%5Cannotation%5CKEGG%5CYK-Unigene.fa_map%5Cmap04976.html) | CL1058.Contig1_YK, CL1058.Contig2_YK, CL1058.Contig3_YK, CL1058.Contig4_YK, CL1088.Contig1_YK, CL1088.Contig2_YK, CL1175.Contig1_YK, CL1175.Contig2_YK, CL1185.Contig1_YK, CL1185.Contig2_YK, CL1213.Contig1_YK, CL1213.Contig2_YK, CL127.Contig1_YK, CL127.Contig2_YK, CL1276.Contig1_YK, CL1301.Contig1_YK, CL1301.Contig2_YK, CL1385.Contig1_YK, CL1454.Contig1_YK, CL1454.Contig2_YK, CL1454.Contig3_YK, CL1454.Contig4_YK, CL1456.Contig3_YK, CL1516.Contig1_YK, CL1516.Contig2_YK, CL1627.Contig1_YK, CL1627.Contig2_YK, CL1627.Contig3_YK, CL1627.Contig4_YK, CL1652.Contig1_YK, CL1652.Contig2_YK, CL1681.Contig1_YK, CL1681.Contig2_YK, CL1681.Contig3_YK, CL1703.Contig1_YK, CL1703.Contig2_YK, CL1703.Contig3_YK, CL1748.Contig1_YK, CL1888.Contig1_YK, CL1994.Contig1_YK, CL1994.Contig2_YK, CL2003.Contig1_YK, CL2003.Contig2_YK, CL2003.Contig3_YK, CL2003.Contig4_YK, CL2003.Contig5_YK, CL2012.Contig1_YK, CL2012.Contig2_YK, CL2083.Contig1_YK, CL2083.Contig2_YK, CL2083.Contig3_YK, CL2092.Contig1_YK, CL2092.Contig2_YK, CL2256.Contig1_YK, CL2256.Contig4_YK, CL2320.Contig1_YK, CL2320.Contig2_YK, CL2320.Contig3_YK, CL2398.Contig1_YK, CL2398.Contig2_YK, CL2419.Contig1_YK, CL243.Contig1_YK, CL243.Contig2_YK, CL2455.Contig1_YK, CL2455.Contig2_YK, CL2459.Contig1_YK, CL2459.Contig2_YK, CL2475.Contig1_YK, CL255.Contig1_YK, CL255.Contig2_YK, CL255.Contig3_YK, CL255.Contig4_YK, CL255.Contig5_YK, CL255.Contig6_YK, CL255.Contig7_YK, CL255.Contig8_YK, CL2561.Contig1_YK, CL268.Contig2_YK, CL268.Contig3_YK, CL268.Contig5_YK, CL268.Contig7_YK, CL2751.Contig1_YK, CL2751.Contig2_YK, CL2791.Contig1_YK, CL2791.Contig2_YK, CL2820.Contig1_YK, CL2820.Contig2_YK, CL2852.Contig1_YK, CL2852.Contig2_YK, CL2878.Contig1_YK, CL374.Contig1_YK, CL636.Contig1_YK, CL648.Contig1_YK, CL648.Contig2_YK, CL65.Contig1_YK, CL65.Contig2_YK, CL691.Contig1_YK, CL691.Contig2_YK, CL730.Contig1_YK, CL730.Contig2_YK, CL765.Contig1_YK, CL765.Contig2_YK, CL769.Contig1_YK, CL769.Contig2_YK, CL769.Contig3_YK, CL79.Contig1_YK, CL79.Contig2_YK, CL79.Contig3_YK, CL79.Contig4_YK, CL79.Contig5_YK, CL79.Contig6_YK, CL79.Contig7_YK, CL79.Contig8_YK, CL801.Contig1_YK, CL801.Contig2_YK, CL801.Contig3_YK, CL801.Contig4_YK, CL801.Contig5_YK, CL801.Contig6_YK, CL803.Contig1_YK, CL803.Contig2_YK, CL842.Contig2_YK, CL857.Contig1_YK, CL857.Contig2_YK, CL979.Contig1_YK, CL979.Contig2_YK, CL979.Contig3_YK, Unigene10001_YK, Unigene10006_YK, Unigene10078_YK, Unigene1009_YK, Unigene10135_YK, Unigene10244_YK, Unigene10292_YK, Unigene10293_YK, Unigene10501_YK, Unigene10650_YK, Unigene10755_YK, Unigene10841_YK, Unigene10842_YK, Unigene10908_YK, Unigene10961_YK, Unigene11189_YK, Unigene11253_YK, Unigene11455_YK, Unigene11466_YK, Unigene11467_YK, Unigene11672_YK, Unigene11673_YK, Unigene11674_YK, Unigene12131_YK, Unigene12132_YK, Unigene12216_YK, Unigene12219_YK, Unigene1223_YK, Unigene12292_YK, Unigene12293_YK, Unigene12699_YK, Unigene12764_YK, Unigene12797_YK, Unigene13055_YK, Unigene13093_YK, Unigene13237_YK, Unigene1332_YK, Unigene13531_YK, Unigene13750_YK, Unigene14049_YK, Unigene14219_YK, Unigene14741_YK, Unigene15086_YK, Unigene15177_YK, Unigene15212_YK, Unigene1560_YK, Unigene15725_YK, Unigene15830_YK, Unigene15875_YK, Unigene15952_YK, Unigene15996_YK, Unigene16032_YK, Unigene16150_YK, Unigene16317_YK, Unigene16322_YK, Unigene16406_YK, Unigene16833_YK, Unigene17113_YK, Unigene17378_YK, Unigene17726_YK, Unigene17893_YK, Unigene18124_YK, Unigene18338_YK, Unigene18738_YK, Unigene1938_YK, Unigene1970_YK, Unigene1997_YK, Unigene2120_YK, Unigene2215_YK, Unigene2231_YK, Unigene2289_YK, Unigene2501_YK, Unigene258_YK, Unigene331_YK, Unigene334_YK, Unigene3435_YK, Unigene3475_YK, Unigene3764_YK, Unigene4038_YK, Unigene4286_YK, Unigene4484_YK, Unigene4485_YK, Unigene4531_YK, Unigene4546_YK, Unigene4705_YK, Unigene4706_YK, Unigene4749_YK, Unigene4852_YK, Unigene5021_YK, Unigene5022_YK, Unigene5147_YK, Unigene5635_YK, Unigene5723_YK, Unigene6203_YK, Unigene6380_YK, Unigene6428_YK, Unigene6429_YK, Unigene6642_YK, Unigene6643_YK, Unigene6980_YK, Unigene7245_YK, Unigene7294_YK, Unigene7338_YK, Unigene7397_YK, Unigene7630_YK, Unigene7631_YK, Unigene7632_YK, Unigene7633_YK, Unigene7745_YK, Unigene7759_YK, Unigene7942_YK, Unigene7943_YK, Unigene7948_YK, Unigene7949_YK, Unigene8101_YK, Unigene8259_YK, Unigene8360_YK, Unigene8382_YK, Unigene8383_YK, Unigene8393_YK, Unigene8394_YK, Unigene84_YK, Unigene8587_YK, Unigene8589_YK, Unigene8590_YK, Unigene8813_YK, Unigene886_YK, Unigene904_YK, Unigene905_YK, Unigene940_YK, Unigene9439_YK, Unigene9440_YK, Unigene9441_YK, Unigene9442_YK, Unigene9443_YK, Unigene9594_YK, Unigene9595_YK, Unigene9602_YK, Unigene9710_YK, Unigene9715_YK, Unigene9716_YK, Unigene9717_YK, Unigene9854_YK, Unigene9856_YK, Unigene9857_YK, Unigene9884_YK |
| 21 | [Epstein-Barr virus infection](../../../../D:%5C高通量测序结果%5CF14FTSSCKF1242_NEMpnqE%5Cannotation%5CKEGG%5CYK-Unigene.fa_map%5Cmap05169.html) | CL1058.Contig4_YK, CL1088.Contig1_YK, CL1088.Contig2_YK, CL1098.Contig1_YK, CL1098.Contig2_YK, CL1144.Contig1_YK, CL1144.Contig2_YK, CL1271.Contig2_YK, CL1308.Contig1_YK, CL1308.Contig2_YK, CL1314.Contig1_YK, CL1314.Contig2_YK, CL1314.Contig3_YK, CL1396.Contig1_YK, CL1396.Contig2_YK, CL1447.Contig1_YK, CL1447.Contig2_YK, CL1448.Contig1_YK, CL1448.Contig2_YK, CL1544.Contig1_YK, CL1544.Contig2_YK, CL1560.Contig1_YK, CL1560.Contig2_YK, CL1560.Contig3_YK, CL1735.Contig1_YK, CL1735.Contig2_YK, CL1758.Contig1_YK, CL1758.Contig2_YK, CL1977.Contig1_YK, CL1977.Contig2_YK, CL1977.Contig3_YK, CL1977.Contig4_YK, CL198.Contig1_YK, CL198.Contig2_YK, CL198.Contig3_YK, CL2008.Contig1_YK, CL204.Contig1_YK, CL204.Contig2_YK, CL2116.Contig1_YK, CL2116.Contig2_YK, CL2153.Contig1_YK, CL2153.Contig2_YK, CL2153.Contig3_YK, CL2153.Contig4_YK, CL2168.Contig1_YK, CL2199.Contig1_YK, CL2199.Contig2_YK, CL2199.Contig3_YK, CL2336.Contig1_YK, CL2336.Contig2_YK, CL2426.Contig2_YK, CL2449.Contig1_YK, CL2449.Contig2_YK, CL2532.Contig1_YK, CL2532.Contig2_YK, CL255.Contig1_YK, CL255.Contig2_YK, CL255.Contig3_YK, CL255.Contig4_YK, CL255.Contig5_YK, CL255.Contig6_YK, CL255.Contig7_YK, CL255.Contig8_YK, CL2624.Contig1_YK, CL2624.Contig2_YK, CL2637.Contig1_YK, CL2637.Contig2_YK, CL2668.Contig1_YK, CL2668.Contig2_YK, CL2720.Contig1_YK, CL2792.Contig1_YK, CL2793.Contig1_YK, CL2793.Contig2_YK, CL2887.Contig1_YK, CL2887.Contig2_YK, CL387.Contig1_YK, CL387.Contig2_YK, CL387.Contig3_YK, CL387.Contig4_YK, CL387.Contig5_YK, CL475.Contig1_YK, CL475.Contig2_YK, CL475.Contig3_YK, CL475.Contig4_YK, CL477.Contig3_YK, CL527.Contig10_YK, CL527.Contig1_YK, CL527.Contig4_YK, CL527.Contig5_YK, CL527.Contig6_YK, CL527.Contig7_YK, CL527.Contig8_YK, CL527.Contig9_YK, CL569.Contig1_YK, CL569.Contig2_YK, CL63.Contig1_YK, CL63.Contig2_YK, CL710.Contig1_YK, CL774.Contig1_YK, CL774.Contig2_YK, CL815.Contig1_YK, CL815.Contig2_YK, CL815.Contig3_YK, CL815.Contig4_YK, CL815.Contig5_YK, CL815.Contig6_YK, CL83.Contig4_YK, CL940.Contig2_YK, Unigene10178_YK, Unigene10659_YK, Unigene10665_YK, Unigene10857_YK, Unigene10972_YK, Unigene11335_YK, Unigene11478_YK, Unigene11499_YK, Unigene11701_YK, Unigene12216_YK, Unigene12218_YK, Unigene12219_YK, Unigene1225_YK, Unigene12777_YK, Unigene12982_YK, Unigene13076_YK, Unigene13128_YK, Unigene13129_YK, Unigene13282_YK, Unigene13313_YK, Unigene13336_YK, Unigene13430_YK, Unigene13446_YK, Unigene13566_YK, Unigene13644_YK, Unigene13661_YK, Unigene13670_YK, Unigene13690_YK, Unigene13696_YK, Unigene13710_YK, Unigene13788_YK, Unigene13841_YK, Unigene13873_YK, Unigene13883_YK, Unigene1393_YK, Unigene13979_YK, Unigene14196_YK, Unigene14218_YK, Unigene14264_YK, Unigene14339_YK, Unigene14381_YK, Unigene14415_YK, Unigene14470_YK, Unigene14497_YK, Unigene14516_YK, Unigene14709_YK, Unigene14733_YK, Unigene14789_YK, Unigene14867_YK, Unigene14925_YK, Unigene14927_YK, Unigene14991_YK, Unigene149_YK, Unigene15080_YK, Unigene15246_YK, Unigene15273_YK, Unigene15345_YK, Unigene15478_YK, Unigene15482_YK, Unigene15497_YK, Unigene15561_YK, Unigene15565_YK, Unigene15726_YK, Unigene15729_YK, Unigene15974_YK, Unigene15979_YK, Unigene16066_YK, Unigene1611_YK, Unigene16177_YK, Unigene16211_YK, Unigene16299_YK, Unigene1656_YK, Unigene1657_YK, Unigene17614_YK, Unigene17725_YK, Unigene179_YK, Unigene18183_YK, Unigene18577_YK, Unigene18638_YK, Unigene18716_YK, Unigene18931_YK, Unigene19055_YK, Unigene19134_YK, Unigene2189_YK, Unigene2376_YK, Unigene2779_YK, Unigene2791_YK, Unigene2888_YK, Unigene2913_YK, Unigene2926_YK, Unigene297_YK, Unigene3031_YK, Unigene3049_YK, Unigene3118_YK, Unigene3122_YK, Unigene3182_YK, Unigene331_YK, Unigene3430_YK, Unigene3448_YK, Unigene3548_YK, Unigene3692_YK, Unigene3934_YK, Unigene4002_YK, Unigene4068_YK, Unigene4103_YK, Unigene4166_YK, Unigene4244_YK, Unigene4292_YK, Unigene4660_YK, Unigene4775_YK, Unigene4906_YK, Unigene4_YK, Unigene5065_YK, Unigene5136_YK, Unigene5196_YK, Unigene5292_YK, Unigene5296_YK, Unigene5558_YK, Unigene5794_YK, Unigene6229_YK, Unigene6230_YK, Unigene6231_YK, Unigene6238_YK, Unigene6406_YK, Unigene6508_YK, Unigene6535_YK, Unigene6645_YK, Unigene6664_YK, Unigene6879_YK, Unigene6880_YK, Unigene6895_YK, Unigene7152_YK, Unigene7462_YK, Unigene7463_YK, Unigene7590_YK, Unigene7610_YK, Unigene7611_YK, Unigene7669_YK, Unigene7755_YK, Unigene7816_YK, Unigene7837_YK, Unigene7838_YK, Unigene7839_YK, Unigene8184_YK, Unigene8185_YK, Unigene8323_YK, Unigene8324_YK, Unigene8325_YK, Unigene8378_YK, Unigene8835_YK, Unigene9071_YK, Unigene9127_YK, Unigene9173_YK, Unigene9706_YK, Unigene989_YK, Unigene990_YK, Unigene994_YK, Unigene9968_YK, Unigene9969_YK, Unigene9985_YK, Unigene9998_YK |
| 22 | [Gastric acid secretion](../../../../D:%5C高通量测序结果%5CF14FTSSCKF1242_NEMpnqE%5Cannotation%5CKEGG%5CYK-Unigene.fa_map%5Cmap04971.html) | CL1058.Contig1_YK, CL1058.Contig2_YK, CL1058.Contig3_YK, CL1058.Contig4_YK, CL1088.Contig1_YK, CL1088.Contig2_YK, CL112.Contig1_YK, CL112.Contig2_YK, CL112.Contig3_YK, CL112.Contig4_YK, CL112.Contig5_YK, CL112.Contig6_YK, CL112.Contig7_YK, CL112.Contig8_YK, CL1185.Contig1_YK, CL1185.Contig2_YK, CL1221.Contig1_YK, CL1221.Contig2_YK, CL1221.Contig3_YK, CL1454.Contig1_YK, CL1454.Contig2_YK, CL1454.Contig3_YK, CL1454.Contig4_YK, CL15.Contig10_YK, CL15.Contig11_YK, CL15.Contig12_YK, CL15.Contig13_YK, CL15.Contig14_YK, CL15.Contig15_YK, CL15.Contig16_YK, CL15.Contig17_YK, CL15.Contig18_YK, CL15.Contig19_YK, CL15.Contig1_YK, CL15.Contig20_YK, CL15.Contig21_YK, CL15.Contig22_YK, CL15.Contig23_YK, CL15.Contig24_YK, CL15.Contig2_YK, CL15.Contig3_YK, CL15.Contig4_YK, CL15.Contig5_YK, CL15.Contig6_YK, CL15.Contig7_YK, CL15.Contig8_YK, CL15.Contig9_YK, CL1627.Contig1_YK, CL1627.Contig2_YK, CL1627.Contig3_YK, CL1627.Contig4_YK, CL1632.Contig1_YK, CL1632.Contig2_YK, CL1714.Contig2_YK, CL1852.Contig2_YK, CL1852.Contig3_YK, CL1858.Contig1_YK, CL1858.Contig2_YK, CL1861.Contig1_YK, CL1948.Contig1_YK, CL1948.Contig2_YK, CL1948.Contig3_YK, CL1990.Contig1_YK, CL1990.Contig2_YK, CL2103.Contig1_YK, CL2103.Contig2_YK, CL2224.Contig2_YK, CL2320.Contig1_YK, CL255.Contig1_YK, CL255.Contig2_YK, CL255.Contig3_YK, CL255.Contig4_YK, CL255.Contig5_YK, CL255.Contig6_YK, CL255.Contig7_YK, CL255.Contig8_YK, CL2593.Contig1_YK, CL2593.Contig2_YK, CL2593.Contig3_YK, CL2792.Contig1_YK, CL2905.Contig1_YK, CL2905.Contig2_YK, CL295.Contig10_YK, CL295.Contig2_YK, CL295.Contig3_YK, CL295.Contig4_YK, CL295.Contig6_YK, CL295.Contig8_YK, CL295.Contig9_YK, CL307.Contig10_YK, CL307.Contig11_YK, CL307.Contig12_YK, CL307.Contig1_YK, CL307.Contig2_YK, CL307.Contig3_YK, CL307.Contig4_YK, CL307.Contig5_YK, CL307.Contig6_YK, CL307.Contig7_YK, CL307.Contig8_YK, CL307.Contig9_YK, CL321.Contig1_YK, CL321.Contig2_YK, CL34.Contig2_YK, CL34.Contig3_YK, CL353.Contig4_YK, CL422.Contig3_YK, CL527.Contig10_YK, CL527.Contig1_YK, CL527.Contig5_YK, CL527.Contig6_YK, CL527.Contig8_YK, CL527.Contig9_YK, CL568.Contig1_YK, CL568.Contig2_YK, CL591.Contig1_YK, CL591.Contig2_YK, CL591.Contig3_YK, CL591.Contig4_YK, CL591.Contig5_YK, CL591.Contig6_YK, CL591.Contig7_YK, CL591.Contig8_YK, CL663.Contig1_YK, CL663.Contig3_YK, CL716.Contig1_YK, CL716.Contig2_YK, CL716.Contig3_YK, CL79.Contig1_YK, CL79.Contig2_YK, CL79.Contig3_YK, CL79.Contig4_YK, CL79.Contig5_YK, CL79.Contig6_YK, CL79.Contig7_YK, CL79.Contig8_YK, CL801.Contig1_YK, CL801.Contig2_YK, CL801.Contig3_YK, CL801.Contig4_YK, CL801.Contig5_YK, CL801.Contig6_YK, CL84.Contig2_YK, CL84.Contig3_YK, CL85.Contig1_YK, CL85.Contig2_YK, CL85.Contig3_YK, CL884.Contig1_YK, CL884.Contig3_YK, CL899.Contig1_YK, CL902.Contig1_YK, CL902.Contig2_YK, Unigene10001_YK, Unigene10006_YK, Unigene10527_YK, Unigene10805_YK, Unigene10908_YK, Unigene11224_YK, Unigene11636_YK, Unigene11837_YK, Unigene12216_YK, Unigene12219_YK, Unigene1223_YK, Unigene12292_YK, Unigene12293_YK, Unigene12560_YK, Unigene12561_YK, Unigene12563_YK, Unigene12564_YK, Unigene12567_YK, Unigene12632_YK, Unigene12636_YK, Unigene12638_YK, Unigene12639_YK, Unigene12785_YK, Unigene12898_YK, Unigene12899_YK, Unigene13020_YK, Unigene13055_YK, Unigene13237_YK, Unigene1332_YK, Unigene13528_YK, Unigene13720_YK, Unigene13750_YK, Unigene13811_YK, Unigene13937_YK, Unigene13957_YK, Unigene13960_YK, Unigene13969_YK, Unigene14243_YK, Unigene14684_YK, Unigene14929_YK, Unigene15057_YK, Unigene15246_YK, Unigene15587_YK, Unigene158_YK, Unigene15952_YK, Unigene15974_YK, Unigene15979_YK, Unigene16057_YK, Unigene16406_YK, Unigene16426_YK, Unigene16843_YK, Unigene18164_YK, Unigene18316_YK, Unigene1964_YK, Unigene2231_YK, Unigene2289_YK, Unigene2550_YK, Unigene258_YK, Unigene2719_YK, Unigene2849_YK, Unigene3067_YK, Unigene331_YK, Unigene352_YK, Unigene3626_YK, Unigene3937_YK, Unigene397_YK, Unigene4011_YK, Unigene4252_YK, Unigene4705_YK, Unigene4706_YK, Unigene4809_YK, Unigene4840_YK, Unigene5162_YK, Unigene5438_YK, Unigene5476_YK, Unigene5477_YK, Unigene5682_YK, Unigene5857_YK, Unigene5874_YK, Unigene6135_YK, Unigene6154_YK, Unigene6206_YK, Unigene6227_YK, Unigene6331_YK, Unigene6461_YK, Unigene6591_YK, Unigene6611_YK, Unigene6980_YK, Unigene6_YK, Unigene7142_YK, Unigene7143_YK, Unigene7307_YK, Unigene7308_YK, Unigene7525_YK, Unigene7822_YK, Unigene8101_YK, Unigene8396_YK, Unigene8587_YK, Unigene8588_YK, Unigene8589_YK, Unigene8590_YK, Unigene8813_YK, Unigene8877_YK, Unigene8908_YK, Unigene904_YK, Unigene905_YK, Unigene9086_YK, Unigene9459_YK, Unigene9594_YK, Unigene9605_YK, Unigene9710_YK, Unigene9715_YK, Unigene9716_YK, Unigene9717_YK, Unigene9884_YK |
| 23 | [Hypertrophic cardiomyopathy (HCM)](../../../../D:%5C高通量测序结果%5CF14FTSSCKF1242_NEMpnqE%5Cannotation%5CKEGG%5CYK-Unigene.fa_map%5Cmap05410.html) | CL1019.Contig1_YK, CL1019.Contig2_YK, CL1019.Contig3_YK, CL1019.Contig4_YK, CL1156.Contig3_YK, CL1221.Contig1_YK, CL1221.Contig2_YK, CL1221.Contig3_YK, CL124.Contig1_YK, CL124.Contig2_YK, CL124.Contig3_YK, CL1260.Contig1_YK, CL1260.Contig2_YK, CL1260.Contig3_YK, CL1303.Contig1_YK, CL1303.Contig2_YK, CL1303.Contig3_YK, CL136.Contig1_YK, CL136.Contig3_YK, CL1486.Contig2_YK, CL1551.Contig1_YK, CL1551.Contig2_YK, CL1577.Contig1_YK, CL1577.Contig2_YK, CL1577.Contig3_YK, CL1577.Contig4_YK, CL1578.Contig2_YK, CL1591.Contig1_YK, CL1606.Contig1_YK, CL1606.Contig2_YK, CL1606.Contig3_YK, CL1638.Contig1_YK, CL1638.Contig2_YK, CL1710.Contig2_YK, CL181.Contig1_YK, CL181.Contig2_YK, CL1891.Contig1_YK, CL1891.Contig2_YK, CL1920.Contig1_YK, CL1920.Contig2_YK, CL1998.Contig1_YK, CL2051.Contig1_YK, CL2051.Contig3_YK, CL2090.Contig1_YK, CL2090.Contig2_YK, CL2090.Contig3_YK, CL2090.Contig4_YK, CL2103.Contig3_YK, CL2426.Contig1_YK, CL25.Contig10_YK, CL25.Contig12_YK, CL25.Contig14_YK, CL25.Contig15_YK, CL25.Contig18_YK, CL25.Contig20_YK, CL25.Contig21_YK, CL25.Contig22_YK, CL25.Contig23_YK, CL25.Contig24_YK, CL25.Contig25_YK, CL25.Contig2_YK, CL25.Contig3_YK, CL25.Contig5_YK, CL25.Contig6_YK, CL25.Contig7_YK, CL25.Contig8_YK, CL25.Contig9_YK, CL2542.Contig1_YK, CL2542.Contig2_YK, CL2560.Contig1_YK, CL2560.Contig2_YK, CL2648.Contig1_YK, CL2669.Contig1_YK, CL2669.Contig2_YK, CL2669.Contig3_YK, CL2669.Contig4_YK, CL2669.Contig5_YK, CL2774.Contig2_YK, CL2798.Contig1_YK, CL2798.Contig2_YK, CL2816.Contig1_YK, CL2905.Contig1_YK, CL2905.Contig2_YK, CL344.Contig3_YK, CL367.Contig1_YK, CL369.Contig2_YK, CL369.Contig5_YK, CL369.Contig8_YK, CL37.Contig2_YK, CL487.Contig1_YK, CL487.Contig2_YK, CL502.Contig1_YK, CL502.Contig2_YK, CL503.Contig1_YK, CL599.Contig1_YK, CL599.Contig2_YK, CL663.Contig1_YK, CL75.Contig1_YK, CL75.Contig2_YK, CL75.Contig3_YK, CL838.Contig1_YK, CL838.Contig2_YK, CL838.Contig3_YK, CL838.Contig4_YK, CL838.Contig5_YK, CL838.Contig8_YK, CL84.Contig2_YK, CL84.Contig3_YK, CL91.Contig1_YK, CL91.Contig2_YK, CL91.Contig3_YK, CL91.Contig4_YK, CL91.Contig5_YK, CL91.Contig6_YK, CL91.Contig7_YK, CL95.Contig2_YK, CL957.Contig1_YK, CL957.Contig2_YK, Unigene10251_YK, Unigene10291_YK, Unigene10296_YK, Unigene10297_YK, Unigene10298_YK, Unigene10355_YK, Unigene10695_YK, Unigene10927_YK, Unigene11080_YK, Unigene11081_YK, Unigene11532_YK, Unigene115_YK, Unigene11679_YK, Unigene11802_YK, Unigene11884_YK, Unigene11889_YK, Unigene12027_YK, Unigene12028_YK, Unigene12334_YK, Unigene12335_YK, Unigene12336_YK, Unigene12337_YK, Unigene12361_YK, Unigene12426_YK, Unigene12467_YK, Unigene12491_YK, Unigene12492_YK, Unigene12493_YK, Unigene12494_YK, Unigene12532_YK, Unigene12852_YK, Unigene12880_YK, Unigene12898_YK, Unigene12899_YK, Unigene13009_YK, Unigene13245_YK, Unigene13296_YK, Unigene13308_YK, Unigene13598_YK, Unigene13704_YK, Unigene13720_YK, Unigene13811_YK, Unigene13890_YK, Unigene13920_YK, Unigene14005_YK, Unigene14016_YK, Unigene14112_YK, Unigene14254_YK, Unigene14327_YK, Unigene14403_YK, Unigene14420_YK, Unigene14661_YK, Unigene14729_YK, Unigene14929_YK, Unigene14966_YK, Unigene15034_YK, Unigene15271_YK, Unigene15308_YK, Unigene15315_YK, Unigene15768_YK, Unigene15804_YK, Unigene15961_YK, Unigene16000_YK, Unigene16018_YK, Unigene16105_YK, Unigene16458_YK, Unigene16741_YK, Unigene16862_YK, Unigene16869_YK, Unigene17296_YK, Unigene17458_YK, Unigene18316_YK, Unigene18657_YK, Unigene18999_YK, Unigene19071_YK, Unigene19153_YK, Unigene2207_YK, Unigene2607_YK, Unigene2620_YK, Unigene2719_YK, Unigene2770_YK, Unigene2808_YK, Unigene2849_YK, Unigene2869_YK, Unigene2892_YK, Unigene3026_YK, Unigene3034_YK, Unigene3038_YK, Unigene3039_YK, Unigene3067_YK, Unigene3188_YK, Unigene3189_YK, Unigene3268_YK, Unigene3271_YK, Unigene3548_YK, Unigene3629_YK, Unigene3650_YK, Unigene3700_YK, Unigene3716_YK, Unigene3739_YK, Unigene3740_YK, Unigene375_YK, Unigene3852_YK, Unigene3906_YK, Unigene3916_YK, Unigene3934_YK, Unigene4055_YK, Unigene4074_YK, Unigene4268_YK, Unigene4315_YK, Unigene4675_YK, Unigene4747_YK, Unigene4825_YK, Unigene4900_YK, Unigene4924_YK, Unigene5603_YK, Unigene5874_YK, Unigene6146_YK, Unigene6270_YK, Unigene6469_YK, Unigene6725_YK, Unigene6814_YK, Unigene6850_YK, Unigene6889_YK, Unigene6890_YK, Unigene7378_YK, Unigene7483_YK, Unigene7662_YK, Unigene7684_YK, Unigene836_YK, Unigene8371_YK, Unigene8396_YK, Unigene8577_YK, Unigene8578_YK, Unigene8755_YK, Unigene8762_YK, Unigene8874_YK, Unigene8875_YK, Unigene8876_YK, Unigene8997_YK, Unigene9029_YK, Unigene9373_YK, Unigene9447_YK, Unigene9449_YK, Unigene9451_YK, Unigene9634_YK, Unigene9832_YK, Unigene9937_YK, Unigene9989_YK |
| 24 | [Insulin signaling pathway](../../../../D:%5C高通量测序结果%5CF14FTSSCKF1242_NEMpnqE%5Cannotation%5CKEGG%5CYK-Unigene.fa_map%5Cmap04910.html) | CL1026.Contig1_YK, CL1026.Contig2_YK, CL1088.Contig1_YK, CL1088.Contig2_YK, CL1099.Contig1_YK, CL1119.Contig1_YK, CL1119.Contig2_YK, CL1315.Contig1_YK, CL1315.Contig2_YK, CL1412.Contig1_YK, CL1500.Contig1_YK, CL1500.Contig2_YK, CL1500.Contig3_YK, CL1602.Contig1_YK, CL1602.Contig2_YK, CL1606.Contig1_YK, CL1606.Contig2_YK, CL1606.Contig3_YK, CL1714.Contig2_YK, CL1861.Contig1_YK, CL194.Contig1_YK, CL194.Contig2_YK, CL194.Contig3_YK, CL194.Contig4_YK, CL200.Contig1_YK, CL200.Contig2_YK, CL21.Contig1_YK, CL21.Contig2_YK, CL229.Contig1_YK, CL229.Contig2_YK, CL229.Contig3_YK, CL229.Contig4_YK, CL238.Contig1_YK, CL238.Contig2_YK, CL238.Contig3_YK, CL25.Contig10_YK, CL25.Contig12_YK, CL25.Contig14_YK, CL25.Contig15_YK, CL25.Contig18_YK, CL25.Contig20_YK, CL25.Contig21_YK, CL25.Contig22_YK, CL25.Contig23_YK, CL25.Contig24_YK, CL25.Contig25_YK, CL25.Contig2_YK, CL25.Contig3_YK, CL25.Contig5_YK, CL25.Contig6_YK, CL25.Contig7_YK, CL25.Contig8_YK, CL25.Contig9_YK, CL2542.Contig1_YK, CL2542.Contig2_YK, CL255.Contig1_YK, CL255.Contig2_YK, CL255.Contig3_YK, CL255.Contig4_YK, CL255.Contig5_YK, CL255.Contig6_YK, CL255.Contig7_YK, CL255.Contig8_YK, CL2593.Contig1_YK, CL2593.Contig2_YK, CL2593.Contig3_YK, CL2668.Contig1_YK, CL2668.Contig2_YK, CL2791.Contig2_YK, CL2792.Contig1_YK, CL2800.Contig1_YK, CL2800.Contig2_YK, CL2898.Contig2_YK, CL312.Contig1_YK, CL369.Contig4_YK, CL369.Contig6_YK, CL376.Contig1_YK, CL376.Contig2_YK, CL376.Contig3_YK, CL376.Contig4_YK, CL376.Contig5_YK, CL376.Contig6_YK, CL524.Contig1_YK, CL524.Contig2_YK, CL568.Contig1_YK, CL568.Contig2_YK, CL577.Contig1_YK, CL577.Contig2_YK, CL577.Contig3_YK, CL577.Contig4_YK, CL577.Contig5_YK, CL577.Contig6_YK, CL639.Contig1_YK, CL639.Contig2_YK, CL774.Contig1_YK, CL774.Contig2_YK, CL859.Contig10_YK, CL859.Contig1_YK, CL859.Contig2_YK, CL859.Contig3_YK, CL859.Contig4_YK, CL859.Contig5_YK, CL859.Contig6_YK, CL859.Contig7_YK, CL859.Contig8_YK, CL859.Contig9_YK, CL899.Contig1_YK, CL899.Contig2_YK, CL899.Contig3_YK, CL899.Contig4_YK, CL899.Contig5_YK, CL902.Contig1_YK, CL902.Contig2_YK, CL935.Contig1_YK, CL935.Contig2_YK, Unigene10137_YK, Unigene10197_YK, Unigene10198_YK, Unigene10623_YK, Unigene10746_YK, Unigene10751_YK, Unigene10752_YK, Unigene10753_YK, Unigene107_YK, Unigene10940_YK, Unigene10972_YK, Unigene10993_YK, Unigene11140_YK, Unigene11141_YK, Unigene11335_YK, Unigene11337_YK, Unigene115_YK, Unigene11662_YK, Unigene11938_YK, Unigene11982_YK, Unigene12128_YK, Unigene12216_YK, Unigene12218_YK, Unigene12219_YK, Unigene12451_YK, Unigene12733_YK, Unigene12830_YK, Unigene12831_YK, Unigene12832_YK, Unigene12833_YK, Unigene12834_YK, Unigene12836_YK, Unigene12837_YK, Unigene12838_YK, Unigene12839_YK, Unigene12840_YK, Unigene12841_YK, Unigene12844_YK, Unigene12845_YK, Unigene12846_YK, Unigene12847_YK, Unigene12848_YK, Unigene12880_YK, Unigene13005_YK, Unigene13023_YK, Unigene13132_YK, Unigene13180_YK, Unigene13240_YK, Unigene13289_YK, Unigene13326_YK, Unigene13374_YK, Unigene13674_YK, Unigene13937_YK, Unigene13960_YK, Unigene14206_YK, Unigene14243_YK, Unigene14345_YK, Unigene14429_YK, Unigene14451_YK, Unigene14577_YK, Unigene14633_YK, Unigene14792_YK, Unigene14942_YK, Unigene15017_YK, Unigene15034_YK, Unigene15057_YK, Unigene15247_YK, Unigene15348_YK, Unigene15497_YK, Unigene15512_YK, Unigene15550_YK, Unigene15561_YK, Unigene15587_YK, Unigene15798_YK, Unigene15910_YK, Unigene16112_YK, Unigene16461_YK, Unigene16653_YK, Unigene201_YK, Unigene2080_YK, Unigene2273_YK, Unigene2490_YK, Unigene2550_YK, Unigene2646_YK, Unigene2791_YK, Unigene2827_YK, Unigene2864_YK, Unigene2865_YK, Unigene3038_YK, Unigene3039_YK, Unigene3281_YK, Unigene3293_YK, Unigene3302_YK, Unigene331_YK, Unigene3357_YK, Unigene337_YK, Unigene3626_YK, Unigene372_YK, Unigene4196_YK, Unigene4256_YK, Unigene4680_YK, Unigene4688_YK, Unigene4689_YK, Unigene4690_YK, Unigene4794_YK, Unigene4_YK, Unigene5037_YK, Unigene5219_YK, Unigene5385_YK, Unigene5386_YK, Unigene5467_YK, Unigene5468_YK, Unigene5524_YK, Unigene5642_YK, Unigene5908_YK, Unigene6227_YK, Unigene6229_YK, Unigene6230_YK, Unigene6231_YK, Unigene6282_YK, Unigene6367_YK, Unigene6368_YK, Unigene6451_YK, Unigene6461_YK, Unigene6561_YK, Unigene6611_YK, Unigene6664_YK, Unigene6726_YK, Unigene6747_YK, Unigene6963_YK, Unigene6_YK, Unigene7040_YK, Unigene7183_YK, Unigene7307_YK, Unigene7308_YK, Unigene7311_YK, Unigene7312_YK, Unigene7371_YK, Unigene7816_YK, Unigene8323_YK, Unigene8324_YK, Unigene8325_YK, Unigene8327_YK, Unigene838_YK, Unigene9036_YK, Unigene9071_YK, Unigene9459_YK, Unigene9968_YK, Unigene9969_YK |
| 25 | [Dopaminergic synapse](../../../../D:%5C高通量测序结果%5CF14FTSSCKF1242_NEMpnqE%5Cannotation%5CKEGG%5CYK-Unigene.fa_map%5Cmap04728.html) | CL1026.Contig1_YK, CL1026.Contig2_YK, CL1088.Contig1_YK, CL1088.Contig2_YK, CL1098.Contig1_YK, CL1098.Contig2_YK, CL112.Contig1_YK, CL112.Contig2_YK, CL112.Contig3_YK, CL112.Contig4_YK, CL112.Contig5_YK, CL112.Contig6_YK, CL112.Contig7_YK, CL112.Contig8_YK, CL1128.Contig1_YK, CL1128.Contig2_YK, CL1412.Contig1_YK, CL15.Contig10_YK, CL15.Contig11_YK, CL15.Contig12_YK, CL15.Contig13_YK, CL15.Contig14_YK, CL15.Contig15_YK, CL15.Contig16_YK, CL15.Contig17_YK, CL15.Contig18_YK, CL15.Contig19_YK, CL15.Contig1_YK, CL15.Contig20_YK, CL15.Contig21_YK, CL15.Contig22_YK, CL15.Contig23_YK, CL15.Contig24_YK, CL15.Contig2_YK, CL15.Contig3_YK, CL15.Contig4_YK, CL15.Contig5_YK, CL15.Contig6_YK, CL15.Contig7_YK, CL15.Contig8_YK, CL15.Contig9_YK, CL1653.Contig1_YK, CL1653.Contig2_YK, CL1689.Contig2_YK, CL1714.Contig2_YK, CL1861.Contig1_YK, CL1933.Contig1_YK, CL1933.Contig2_YK, CL1949.Contig1_YK, CL1949.Contig2_YK, CL1968.Contig1_YK, CL1968.Contig2_YK, CL1977.Contig1_YK, CL1977.Contig2_YK, CL1977.Contig3_YK, CL1977.Contig4_YK, CL2168.Contig1_YK, CL2192.Contig1_YK, CL2192.Contig2_YK, CL2265.Contig1_YK, CL2265.Contig2_YK, CL255.Contig1_YK, CL255.Contig2_YK, CL255.Contig3_YK, CL255.Contig4_YK, CL255.Contig5_YK, CL255.Contig6_YK, CL255.Contig7_YK, CL255.Contig8_YK, CL2593.Contig1_YK, CL2593.Contig2_YK, CL2593.Contig3_YK, CL26.Contig1_YK, CL2624.Contig1_YK, CL2624.Contig2_YK, CL2668.Contig1_YK, CL2668.Contig2_YK, CL278.Contig3_YK, CL278.Contig4_YK, CL295.Contig10_YK, CL295.Contig2_YK, CL295.Contig3_YK, CL295.Contig4_YK, CL295.Contig6_YK, CL295.Contig8_YK, CL295.Contig9_YK, CL307.Contig10_YK, CL307.Contig11_YK, CL307.Contig12_YK, CL307.Contig1_YK, CL307.Contig2_YK, CL307.Contig3_YK, CL307.Contig4_YK, CL307.Contig5_YK, CL307.Contig6_YK, CL307.Contig7_YK, CL307.Contig8_YK, CL307.Contig9_YK, CL312.Contig1_YK, CL568.Contig1_YK, CL568.Contig2_YK, CL591.Contig1_YK, CL591.Contig2_YK, CL591.Contig3_YK, CL591.Contig4_YK, CL591.Contig5_YK, CL591.Contig6_YK, CL591.Contig7_YK, CL591.Contig8_YK, CL606.Contig1_YK, CL606.Contig2_YK, CL606.Contig3_YK, CL774.Contig1_YK, CL774.Contig2_YK, CL838.Contig1_YK, CL838.Contig2_YK, CL838.Contig3_YK, CL838.Contig4_YK, CL838.Contig5_YK, CL838.Contig8_YK, CL884.Contig1_YK, CL884.Contig3_YK, CL889.Contig1_YK, CL889.Contig2_YK, CL889.Contig3_YK, CL889.Contig4_YK, CL902.Contig1_YK, CL902.Contig2_YK, CL935.Contig1_YK, CL935.Contig2_YK, Unigene10209_YK, Unigene10210_YK, Unigene10211_YK, Unigene10546_YK, Unigene10547_YK, Unigene10614_YK, Unigene10627_YK, Unigene10908_YK, Unigene11140_YK, Unigene11141_YK, Unigene11335_YK, Unigene11662_YK, Unigene11889_YK, Unigene11890_YK, Unigene12128_YK, Unigene12216_YK, Unigene12218_YK, Unigene12219_YK, Unigene1223_YK, Unigene12560_YK, Unigene12561_YK, Unigene12563_YK, Unigene12564_YK, Unigene12636_YK, Unigene12638_YK, Unigene12639_YK, Unigene12733_YK, Unigene13069_YK, Unigene13180_YK, Unigene13237_YK, Unigene1356_YK, Unigene13670_YK, Unigene13692_YK, Unigene13937_YK, Unigene13960_YK, Unigene13969_YK, Unigene14080_YK, Unigene14094_YK, Unigene14159_YK, Unigene14243_YK, Unigene14276_YK, Unigene14507_YK, Unigene15057_YK, Unigene15348_YK, Unigene15561_YK, Unigene15587_YK, Unigene15877_YK, Unigene158_YK, Unigene16000_YK, Unigene16112_YK, Unigene16638_YK, Unigene17790_YK, Unigene18164_YK, Unigene18574_YK, Unigene18798_YK, Unigene18819_YK, Unigene1964_YK, Unigene201_YK, Unigene2080_YK, Unigene2148_YK, Unigene2550_YK, Unigene258_YK, Unigene319_YK, Unigene3200_YK, Unigene3214_YK, Unigene3219_YK, Unigene331_YK, Unigene337_YK, Unigene3424_YK, Unigene3439_YK, Unigene3596_YK, Unigene3626_YK, Unigene3885_YK, Unigene3937_YK, Unigene4062_YK, Unigene4179_YK, Unigene4252_YK, Unigene4419_YK, Unigene4445_YK, Unigene4564_YK, Unigene4565_YK, Unigene4689_YK, Unigene4690_YK, Unigene4772_YK, Unigene4_YK, Unigene5385_YK, Unigene5386_YK, Unigene5438_YK, Unigene5467_YK, Unigene5468_YK, Unigene5476_YK, Unigene5477_YK, Unigene5835_YK, Unigene5836_YK, Unigene5857_YK, Unigene6006_YK, Unigene6007_YK, Unigene6206_YK, Unigene6227_YK, Unigene6229_YK, Unigene6230_YK, Unigene6231_YK, Unigene6367_YK, Unigene6368_YK, Unigene6461_YK, Unigene6611_YK, Unigene6664_YK, Unigene6980_YK, Unigene6_YK, Unigene7153_YK, Unigene7307_YK, Unigene7308_YK, Unigene7391_YK, Unigene7525_YK, Unigene765_YK, Unigene7816_YK, Unigene8101_YK, Unigene838_YK, Unigene8606_YK, Unigene8677_YK, Unigene8678_YK, Unigene8679_YK, Unigene8682_YK, Unigene8683_YK, Unigene904_YK, Unigene905_YK, Unigene9071_YK, Unigene9168_YK, Unigene9169_YK, Unigene9459_YK, Unigene9968_YK, Unigene9969_YK, Unigene9984_YK |
| 26 | [RNA transport](../../../../D:%5C高通量测序结果%5CF14FTSSCKF1242_NEMpnqE%5Cannotation%5CKEGG%5CYK-Unigene.fa_map%5Cmap03013.html) | CL1032.Contig1_YK, CL1114.Contig1_YK, CL1114.Contig2_YK, CL1114.Contig4_YK, CL1114.Contig5_YK, CL1114.Contig6_YK, CL1114.Contig9_YK, CL1115.Contig2_YK, CL1134.Contig1_YK, CL1134.Contig2_YK, CL1144.Contig1_YK, CL1144.Contig2_YK, CL117.Contig2_YK, CL1244.Contig2_YK, CL1244.Contig3_YK, CL1324.Contig3_YK, CL1404.Contig2_YK, CL1451.Contig1_YK, CL1451.Contig2_YK, CL146.Contig2_YK, CL146.Contig4_YK, CL1463.Contig1_YK, CL1463.Contig2_YK, CL1474.Contig1_YK, CL1761.Contig1_YK, CL1761.Contig2_YK, CL1792.Contig1_YK, CL1792.Contig2_YK, CL1840.Contig1_YK, CL1840.Contig2_YK, CL1898.Contig1_YK, CL1898.Contig2_YK, CL1915.Contig1_YK, CL1915.Contig2_YK, CL1915.Contig3_YK, CL2008.Contig1_YK, CL2008.Contig2_YK, CL2054.Contig1_YK, CL2062.Contig1_YK, CL2062.Contig2_YK, CL2062.Contig3_YK, CL2069.Contig1_YK, CL2224.Contig2_YK, CL2359.Contig2_YK, CL2527.Contig1_YK, CL2527.Contig3_YK, CL2554.Contig1_YK, CL2554.Contig2_YK, CL2587.Contig2_YK, CL2648.Contig2_YK, CL27.Contig1_YK, CL2788.Contig1_YK, CL2788.Contig2_YK, CL2805.Contig1_YK, CL34.Contig1_YK, CL37.Contig1_YK, CL414.Contig1_YK, CL414.Contig2_YK, CL477.Contig1_YK, CL500.Contig1_YK, CL522.Contig1_YK, CL522.Contig2_YK, CL522.Contig3_YK, CL599.Contig1_YK, CL599.Contig2_YK, CL625.Contig1_YK, CL625.Contig2_YK, CL644.Contig1_YK, CL644.Contig2_YK, CL662.Contig1_YK, CL662.Contig2_YK, CL783.Contig1_YK, CL783.Contig2_YK, CL783.Contig3_YK, CL788.Contig1_YK, CL95.Contig2_YK, Unigene10060_YK, Unigene10077_YK, Unigene10251_YK, Unigene10683_YK, Unigene10889_YK, Unigene10901_YK, Unigene11321_YK, Unigene11402_YK, Unigene11447_YK, Unigene12210_YK, Unigene12458_YK, Unigene12524_YK, Unigene12583_YK, Unigene12777_YK, Unigene12852_YK, Unigene13112_YK, Unigene1311_YK, Unigene13128_YK, Unigene13141_YK, Unigene13193_YK, Unigene13230_YK, Unigene13240_YK, Unigene13241_YK, Unigene13250_YK, Unigene13258_YK, Unigene13266_YK, Unigene13300_YK, Unigene13333_YK, Unigene13340_YK, Unigene13343_YK, Unigene13358_YK, Unigene13432_YK, Unigene13484_YK, Unigene13519_YK, Unigene13524_YK, Unigene13557_YK, Unigene13588_YK, Unigene13660_YK, Unigene13669_YK, Unigene13685_YK, Unigene13690_YK, Unigene13693_YK, Unigene13746_YK, Unigene13821_YK, Unigene13864_YK, Unigene13871_YK, Unigene13893_YK, Unigene13917_YK, Unigene13962_YK, Unigene14010_YK, Unigene14013_YK, Unigene14079_YK, Unigene14207_YK, Unigene14218_YK, Unigene14228_YK, Unigene14272_YK, Unigene14327_YK, Unigene14359_YK, Unigene14464_YK, Unigene14536_YK, Unigene14539_YK, Unigene14598_YK, Unigene14603_YK, Unigene14784_YK, Unigene147_YK, Unigene14803_YK, Unigene14904_YK, Unigene15015_YK, Unigene15034_YK, Unigene15056_YK, Unigene15153_YK, Unigene15163_YK, Unigene15215_YK, Unigene15376_YK, Unigene15394_YK, Unigene15427_YK, Unigene15491_YK, Unigene15512_YK, Unigene15520_YK, Unigene15612_YK, Unigene15669_YK, Unigene15690_YK, Unigene15734_YK, Unigene15857_YK, Unigene15891_YK, Unigene16057_YK, Unigene16826_YK, Unigene16880_YK, Unigene17_YK, Unigene18069_YK, Unigene18161_YK, Unigene18493_YK, Unigene19052_YK, Unigene19131_YK, Unigene2009_YK, Unigene2100_YK, Unigene2109_YK, Unigene2173_YK, Unigene2436_YK, Unigene2797_YK, Unigene2813_YK, Unigene2819_YK, Unigene2827_YK, Unigene2866_YK, Unigene2876_YK, Unigene2889_YK, Unigene2942_YK, Unigene2949_YK, Unigene2981_YK, Unigene2984_YK, Unigene3015_YK, Unigene3149_YK, Unigene3156_YK, Unigene3199_YK, Unigene3270_YK, Unigene3281_YK, Unigene3350_YK, Unigene3395_YK, Unigene3518_YK, Unigene3527_YK, Unigene3556_YK, Unigene3601_YK, Unigene370_YK, Unigene3822_YK, Unigene3880_YK, Unigene3907_YK, Unigene4034_YK, Unigene4071_YK, Unigene4072_YK, Unigene4096_YK, Unigene4183_YK, Unigene4208_YK, Unigene4525_YK, Unigene4794_YK, Unigene5010_YK, Unigene5037_YK, Unigene5065_YK, Unigene5206_YK, Unigene5296_YK, Unigene5300_YK, Unigene5337_YK, Unigene5354_YK, Unigene5370_YK, Unigene5755_YK, Unigene577_YK, Unigene5825_YK, Unigene5899_YK, Unigene5900_YK, Unigene5964_YK, Unigene5977_YK, Unigene5986_YK, Unigene6039_YK, Unigene6046_YK, Unigene6104_YK, Unigene6167_YK, Unigene6205_YK, Unigene6228_YK, Unigene6334_YK, Unigene6336_YK, Unigene6411_YK, Unigene6664_YK, Unigene6716_YK, Unigene6717_YK, Unigene6814_YK, Unigene6892_YK, Unigene7011_YK, Unigene7012_YK, Unigene7082_YK, Unigene7237_YK, Unigene7370_YK, Unigene7594_YK, Unigene7651_YK, Unigene7841_YK, Unigene7875_YK, Unigene8490_YK, Unigene8491_YK, Unigene8492_YK, Unigene8691_YK, Unigene8692_YK, Unigene9051_YK, Unigene9258_YK, Unigene9259_YK, Unigene9557_YK, Unigene9625_YK, Unigene9944_YK |
| 27 | [Protein processing in endoplasmic reticulum](../../../../D:%5C高通量测序结果%5CF14FTSSCKF1242_NEMpnqE%5Cannotation%5CKEGG%5CYK-Unigene.fa_map%5Cmap04141.html) | CL1004.Contig1_YK, CL1004.Contig2_YK, CL1207.Contig1_YK, CL1207.Contig2_YK, CL1207.Contig3_YK, CL1207.Contig4_YK, CL1207.Contig5_YK, CL1218.Contig1_YK, CL1218.Contig2_YK, CL1266.Contig1_YK, CL1266.Contig2_YK, CL1266.Contig3_YK, CL1266.Contig4_YK, CL1271.Contig2_YK, CL1366.Contig1_YK, CL1366.Contig2_YK, CL1457.Contig1_YK, CL1457.Contig2_YK, CL1653.Contig1_YK, CL1653.Contig2_YK, CL1686.Contig1_YK, CL1686.Contig2_YK, CL1732.Contig1_YK, CL1741.Contig1_YK, CL1741.Contig2_YK, CL1741.Contig3_YK, CL1741.Contig4_YK, CL1893.Contig1_YK, CL1893.Contig2_YK, CL198.Contig1_YK, CL198.Contig2_YK, CL198.Contig3_YK, CL2104.Contig1_YK, CL2104.Contig2_YK, CL2122.Contig1_YK, CL2122.Contig2_YK, CL2155.Contig1_YK, CL2155.Contig2_YK, CL2414.Contig1_YK, CL2414.Contig2_YK, CL2521.Contig1_YK, CL2521.Contig2_YK, CL2627.Contig1_YK, CL2627.Contig2_YK, CL2670.Contig1_YK, CL2702.Contig1_YK, CL271.Contig1_YK, CL2720.Contig1_YK, CL2752.Contig1_YK, CL296.Contig10_YK, CL296.Contig11_YK, CL296.Contig12_YK, CL296.Contig13_YK, CL296.Contig14_YK, CL296.Contig15_YK, CL296.Contig16_YK, CL296.Contig1_YK, CL296.Contig2_YK, CL296.Contig3_YK, CL296.Contig4_YK, CL296.Contig5_YK, CL296.Contig6_YK, CL296.Contig7_YK, CL296.Contig8_YK, CL296.Contig9_YK, CL474.Contig1_YK, CL539.Contig1_YK, CL539.Contig2_YK, CL539.Contig3_YK, CL539.Contig4_YK, CL964.Contig1_YK, CL964.Contig2_YK, CL964.Contig3_YK, CL964.Contig4_YK, Unigene10347_YK, Unigene10451_YK, Unigene10665_YK, Unigene10765_YK, Unigene10800_YK, Unigene10947_YK, Unigene11024_YK, Unigene11163_YK, Unigene11408_YK, Unigene11553_YK, Unigene11799_YK, Unigene11800_YK, Unigene11846_YK, Unigene1214_YK, Unigene1225_YK, Unigene12644_YK, Unigene12858_YK, Unigene13012_YK, Unigene13014_YK, Unigene13041_YK, Unigene13057_YK, Unigene13074_YK, Unigene13113_YK, Unigene13117_YK, Unigene13136_YK, Unigene13149_YK, Unigene13155_YK, Unigene13252_YK, Unigene13281_YK, Unigene13309_YK, Unigene13322_YK, Unigene13324_YK, Unigene13327_YK, Unigene13376_YK, Unigene13387_YK, Unigene13406_YK, Unigene13412_YK, Unigene13415_YK, Unigene13418_YK, Unigene13457_YK, Unigene13466_YK, Unigene13526_YK, Unigene13576_YK, Unigene13583_YK, Unigene13609_YK, Unigene13612_YK, Unigene13617_YK, Unigene13645_YK, Unigene13650_YK, Unigene13678_YK, Unigene13684_YK, Unigene13728_YK, Unigene13746_YK, Unigene13756_YK, Unigene13791_YK, Unigene13883_YK, Unigene1393_YK, Unigene13962_YK, Unigene13995_YK, Unigene14020_YK, Unigene14024_YK, Unigene14036_YK, Unigene14211_YK, Unigene14298_YK, Unigene14348_YK, Unigene14394_YK, Unigene14415_YK, Unigene14501_YK, Unigene14512_YK, Unigene14709_YK, Unigene14772_YK, Unigene14837_YK, Unigene14851_YK, Unigene15055_YK, Unigene15100_YK, Unigene15214_YK, Unigene15305_YK, Unigene15332_YK, Unigene15335_YK, Unigene15393_YK, Unigene15839_YK, Unigene16217_YK, Unigene1656_YK, Unigene1657_YK, Unigene16835_YK, Unigene17614_YK, Unigene17725_YK, Unigene17881_YK, Unigene1790_YK, Unigene18210_YK, Unigene18319_YK, Unigene18352_YK, Unigene18574_YK, Unigene18590_YK, Unigene18611_YK, Unigene18630_YK, Unigene18819_YK, Unigene18955_YK, Unigene19055_YK, Unigene19132_YK, Unigene19133_YK, Unigene2033_YK, Unigene2243_YK, Unigene2329_YK, Unigene2745_YK, Unigene2749_YK, Unigene2757_YK, Unigene2851_YK, Unigene2883_YK, Unigene2887_YK, Unigene2957_YK, Unigene3111_YK, Unigene3211_YK, Unigene3225_YK, Unigene323_YK, Unigene3268_YK, Unigene3277_YK, Unigene3315_YK, Unigene3316_YK, Unigene3374_YK, Unigene3439_YK, Unigene3493_YK, Unigene3621_YK, Unigene376_YK, Unigene3806_YK, Unigene3807_YK, Unigene3883_YK, Unigene3926_YK, Unigene4068_YK, Unigene4117_YK, Unigene4243_YK, Unigene4493_YK, Unigene4536_YK, Unigene4610_YK, Unigene4660_YK, Unigene4727_YK, Unigene4803_YK, Unigene485_YK, Unigene4868_YK, Unigene486_YK, Unigene4876_YK, Unigene4994_YK, Unigene5266_YK, Unigene5414_YK, Unigene5495_YK, Unigene5595_YK, Unigene5663_YK, Unigene5895_YK, Unigene5995_YK, Unigene5999_YK, Unigene6106_YK, Unigene6214_YK, Unigene6223_YK, Unigene6337_YK, Unigene6355_YK, Unigene6421_YK, Unigene6669_YK, Unigene6690_YK, Unigene6860_YK, Unigene6861_YK, Unigene7500_YK, Unigene7680_YK, Unigene7837_YK, Unigene7838_YK, Unigene7839_YK, Unigene8395_YK, Unigene8835_YK, Unigene9071_YK, Unigene9154_YK, Unigene9232_YK, Unigene9233_YK, Unigene9310_YK, Unigene9525_YK |
| 28 | [Wnt signaling pathway](../../../../D:%5C高通量测序结果%5CF14FTSSCKF1242_NEMpnqE%5Cannotation%5CKEGG%5CYK-Unigene.fa_map%5Cmap04310.html) | CL1004.Contig1_YK, CL1004.Contig2_YK, CL1088.Contig1_YK, CL1088.Contig2_YK, CL112.Contig1_YK, CL112.Contig2_YK, CL112.Contig3_YK, CL112.Contig4_YK, CL112.Contig5_YK, CL112.Contig6_YK, CL112.Contig7_YK, CL112.Contig8_YK, CL1170.Contig1_YK, CL1170.Contig2_YK, CL1170.Contig3_YK, CL1170.Contig4_YK, CL1282.Contig1_YK, CL1282.Contig2_YK, CL1381.Contig1_YK, CL1381.Contig2_YK, CL1390.Contig1_YK, CL1390.Contig2_YK, CL15.Contig10_YK, CL15.Contig11_YK, CL15.Contig12_YK, CL15.Contig13_YK, CL15.Contig14_YK, CL15.Contig15_YK, CL15.Contig16_YK, CL15.Contig17_YK, CL15.Contig18_YK, CL15.Contig19_YK, CL15.Contig1_YK, CL15.Contig20_YK, CL15.Contig21_YK, CL15.Contig22_YK, CL15.Contig23_YK, CL15.Contig24_YK, CL15.Contig2_YK, CL15.Contig3_YK, CL15.Contig4_YK, CL15.Contig5_YK, CL15.Contig6_YK, CL15.Contig7_YK, CL15.Contig8_YK, CL15.Contig9_YK, CL1538.Contig1_YK, CL1538.Contig2_YK, CL1577.Contig1_YK, CL1577.Contig2_YK, CL1577.Contig3_YK, CL1577.Contig4_YK, CL1578.Contig1_YK, CL1578.Contig2_YK, CL1679.Contig1_YK, CL1679.Contig2_YK, CL1679.Contig3_YK, CL17.Contig1_YK, CL17.Contig2_YK, CL1758.Contig1_YK, CL1758.Contig2_YK, CL1816.Contig1_YK, CL1816.Contig2_YK, CL1949.Contig1_YK, CL1949.Contig2_YK, CL2006.Contig1_YK, CL2006.Contig2_YK, CL2006.Contig3_YK, CL2135.Contig1_YK, CL2135.Contig2_YK, CL2288.Contig1_YK, CL2288.Contig2_YK, CL2333.Contig1_YK, CL2333.Contig2_YK, CL2336.Contig1_YK, CL2336.Contig2_YK, CL2411.Contig1_YK, CL2411.Contig2_YK, CL2411.Contig3_YK, CL2422.Contig1_YK, CL2422.Contig2_YK, CL2544.Contig1_YK, CL2544.Contig2_YK, CL255.Contig1_YK, CL255.Contig2_YK, CL255.Contig3_YK, CL255.Contig4_YK, CL255.Contig5_YK, CL255.Contig6_YK, CL255.Contig7_YK, CL255.Contig8_YK, CL2635.Contig1_YK, CL2635.Contig2_YK, CL2666.Contig1_YK, CL2666.Contig2_YK, CL2668.Contig1_YK, CL2668.Contig2_YK, CL2729.Contig1_YK, CL2783.Contig1_YK, CL2793.Contig1_YK, CL2793.Contig2_YK, CL2817.Contig1_YK, CL2817.Contig2_YK, CL2826.Contig1_YK, CL2826.Contig2_YK, CL2866.Contig1_YK, CL2887.Contig2_YK, CL307.Contig10_YK, CL307.Contig11_YK, CL307.Contig12_YK, CL307.Contig1_YK, CL307.Contig2_YK, CL307.Contig3_YK, CL307.Contig4_YK, CL307.Contig5_YK, CL307.Contig6_YK, CL307.Contig7_YK, CL307.Contig8_YK, CL307.Contig9_YK, CL387.Contig1_YK, CL387.Contig2_YK, CL387.Contig3_YK, CL387.Contig4_YK, CL387.Contig5_YK, CL425.Contig1_YK, CL425.Contig2_YK, CL431.Contig1_YK, CL431.Contig2_YK, CL431.Contig3_YK, CL591.Contig1_YK, CL591.Contig2_YK, CL591.Contig3_YK, CL591.Contig4_YK, CL591.Contig5_YK, CL591.Contig6_YK, CL591.Contig7_YK, CL591.Contig8_YK, CL720.Contig1_YK, CL720.Contig2_YK, CL774.Contig1_YK, CL774.Contig2_YK, CL814.Contig1_YK, CL814.Contig2_YK, CL83.Contig4_YK, CL832.Contig1_YK, CL884.Contig1_YK, CL884.Contig3_YK, Unigene10096_YK, Unigene10238_YK, Unigene10546_YK, Unigene10547_YK, Unigene10577_YK, Unigene10621_YK, Unigene10659_YK, Unigene10954_YK, Unigene11008_YK, Unigene11075_YK, Unigene11477_YK, Unigene12216_YK, Unigene12219_YK, Unigene12636_YK, Unigene12638_YK, Unigene12639_YK, Unigene13069_YK, Unigene13313_YK, Unigene13332_YK, Unigene13457_YK, Unigene13558_YK, Unigene13560_YK, Unigene13594_YK, Unigene13791_YK, Unigene13843_YK, Unigene13877_YK, Unigene13985_YK, Unigene14013_YK, Unigene14254_YK, Unigene14265_YK, Unigene14276_YK, Unigene14516_YK, Unigene14550_YK, Unigene14598_YK, Unigene14681_YK, Unigene14721_YK, Unigene14892_YK, Unigene15325_YK, Unigene15370_YK, Unigene15561_YK, Unigene15709_YK, Unigene15780_YK, Unigene158_YK, Unigene16250_YK, Unigene16684_YK, Unigene17880_YK, Unigene18931_YK, Unigene26_YK, Unigene2813_YK, Unigene3032_YK, Unigene317_YK, Unigene3214_YK, Unigene3315_YK, Unigene3316_YK, Unigene3318_YK, Unigene331_YK, Unigene3321_YK, Unigene3348_YK, Unigene3430_YK, Unigene3544_YK, Unigene3715_YK, Unigene4062_YK, Unigene4179_YK, Unigene4203_YK, Unigene4252_YK, Unigene4308_YK, Unigene4396_YK, Unigene4494_YK, Unigene4508_YK, Unigene4_YK, Unigene5191_YK, Unigene5476_YK, Unigene5477_YK, Unigene5495_YK, Unigene5519_YK, Unigene5656_YK, Unigene5941_YK, Unigene5993_YK, Unigene6027_YK, Unigene6664_YK, Unigene6772_YK, Unigene6879_YK, Unigene6880_YK, Unigene6998_YK, Unigene7153_YK, Unigene7382_YK, Unigene7525_YK, Unigene7701_YK, Unigene7816_YK, Unigene7904_YK, Unigene8001_YK, Unigene9071_YK, Unigene9127_YK, Unigene9323_YK, Unigene9388_YK, Unigene9642_YK, Unigene9968_YK, Unigene9969_YK |
| 29 | [Salivary secretion](../../../../D:%5C高通量测序结果%5CF14FTSSCKF1242_NEMpnqE%5Cannotation%5CKEGG%5CYK-Unigene.fa_map%5Cmap04970.html) | CL1041.Contig1_YK, CL1041.Contig2_YK, CL1058.Contig1_YK, CL1058.Contig2_YK, CL1058.Contig3_YK, CL1058.Contig4_YK, CL1088.Contig1_YK, CL1088.Contig2_YK, CL1185.Contig1_YK, CL1185.Contig2_YK, CL1323.Contig1_YK, CL1323.Contig2_YK, CL1362.Contig3_YK, CL15.Contig10_YK, CL15.Contig11_YK, CL15.Contig12_YK, CL15.Contig13_YK, CL15.Contig14_YK, CL15.Contig15_YK, CL15.Contig16_YK, CL15.Contig17_YK, CL15.Contig18_YK, CL15.Contig19_YK, CL15.Contig1_YK, CL15.Contig20_YK, CL15.Contig21_YK, CL15.Contig22_YK, CL15.Contig23_YK, CL15.Contig24_YK, CL15.Contig2_YK, CL15.Contig3_YK, CL15.Contig4_YK, CL15.Contig5_YK, CL15.Contig6_YK, CL15.Contig7_YK, CL15.Contig8_YK, CL15.Contig9_YK, CL1553.Contig1_YK, CL1553.Contig2_YK, CL1553.Contig3_YK, CL1557.Contig1_YK, CL1627.Contig1_YK, CL1627.Contig2_YK, CL1627.Contig3_YK, CL1627.Contig4_YK, CL1630.Contig2_YK, CL1664.Contig1_YK, CL1664.Contig2_YK, CL1714.Contig2_YK, CL1861.Contig1_YK, CL1990.Contig1_YK, CL1990.Contig2_YK, CL2253.Contig1_YK, CL2253.Contig2_YK, CL2283.Contig1_YK, CL2283.Contig2_YK, CL2283.Contig3_YK, CL2283.Contig4_YK, CL2320.Contig1_YK, CL2355.Contig1_YK, CL255.Contig1_YK, CL255.Contig2_YK, CL255.Contig3_YK, CL255.Contig4_YK, CL255.Contig5_YK, CL255.Contig6_YK, CL255.Contig7_YK, CL255.Contig8_YK, CL2593.Contig1_YK, CL2593.Contig2_YK, CL2593.Contig3_YK, CL2665.Contig1_YK, CL2665.Contig2_YK, CL295.Contig10_YK, CL295.Contig2_YK, CL295.Contig3_YK, CL295.Contig4_YK, CL295.Contig6_YK, CL295.Contig8_YK, CL295.Contig9_YK, CL307.Contig10_YK, CL307.Contig11_YK, CL307.Contig12_YK, CL307.Contig1_YK, CL307.Contig2_YK, CL307.Contig3_YK, CL307.Contig4_YK, CL307.Contig5_YK, CL307.Contig6_YK, CL307.Contig7_YK, CL307.Contig8_YK, CL307.Contig9_YK, CL416.Contig2_YK, CL416.Contig3_YK, CL477.Contig2_YK, CL499.Contig1_YK, CL499.Contig2_YK, CL529.Contig1_YK, CL529.Contig2_YK, CL529.Contig3_YK, CL529.Contig4_YK, CL529.Contig5_YK, CL529.Contig6_YK, CL568.Contig1_YK, CL568.Contig2_YK, CL591.Contig1_YK, CL591.Contig2_YK, CL591.Contig3_YK, CL591.Contig4_YK, CL591.Contig5_YK, CL591.Contig6_YK, CL591.Contig7_YK, CL591.Contig8_YK, CL657.Contig1_YK, CL657.Contig2_YK, CL79.Contig1_YK, CL79.Contig2_YK, CL79.Contig3_YK, CL79.Contig4_YK, CL79.Contig5_YK, CL79.Contig6_YK, CL79.Contig7_YK, CL79.Contig8_YK, CL801.Contig1_YK, CL801.Contig2_YK, CL801.Contig3_YK, CL801.Contig4_YK, CL801.Contig5_YK, CL801.Contig6_YK, CL884.Contig1_YK, CL884.Contig3_YK, CL902.Contig1_YK, CL902.Contig2_YK, CL914.Contig1_YK, CL914.Contig2_YK, Unigene10001_YK, Unigene10449_YK, Unigene107_YK, Unigene10887_YK, Unigene10888_YK, Unigene10908_YK, Unigene10999_YK, Unigene11002_YK, Unigene11173_YK, Unigene11174_YK, Unigene11496_YK, Unigene11837_YK, Unigene11937_YK, Unigene12182_YK, Unigene12216_YK, Unigene12219_YK, Unigene1223_YK, Unigene12292_YK, Unigene12293_YK, Unigene12478_YK, Unigene12560_YK, Unigene12561_YK, Unigene12563_YK, Unigene12564_YK, Unigene12636_YK, Unigene12638_YK, Unigene12639_YK, Unigene12768_YK, Unigene12824_YK, Unigene12870_YK, Unigene13055_YK, Unigene13094_YK, Unigene13237_YK, Unigene1332_YK, Unigene13342_YK, Unigene13750_YK, Unigene13937_YK, Unigene13960_YK, Unigene14243_YK, Unigene14692_YK, Unigene15057_YK, Unigene15587_YK, Unigene158_YK, Unigene16406_YK, Unigene16678_YK, Unigene16940_YK, Unigene17466_YK, Unigene18798_YK, Unigene19144_YK, Unigene1964_YK, Unigene2231_YK, Unigene2287_YK, Unigene2289_YK, Unigene2377_YK, Unigene254_YK, Unigene2550_YK, Unigene258_YK, Unigene303_YK, Unigene331_YK, Unigene352_YK, Unigene3626_YK, Unigene4087_YK, Unigene4244_YK, Unigene4252_YK, Unigene4411_YK, Unigene4713_YK, Unigene5476_YK, Unigene5477_YK, Unigene5557_YK, Unigene5577_YK, Unigene5857_YK, Unigene6011_YK, Unigene6206_YK, Unigene6227_YK, Unigene6413_YK, Unigene6461_YK, Unigene6611_YK, Unigene6980_YK, Unigene6_YK, Unigene7307_YK, Unigene7308_YK, Unigene7337_YK, Unigene7525_YK, Unigene7579_YK, Unigene7926_YK, Unigene8101_YK, Unigene8561_YK, Unigene8562_YK, Unigene8563_YK, Unigene8564_YK, Unigene8587_YK, Unigene8589_YK, Unigene8590_YK, Unigene904_YK, Unigene905_YK, Unigene9198_YK, Unigene9356_YK, Unigene9357_YK, Unigene9358_YK, Unigene9459_YK, Unigene9527_YK, Unigene9594_YK, Unigene9710_YK, Unigene9715_YK, Unigene9716_YK, Unigene9717_YK, Unigene9884_YK, Unigene9934_YK |
| 30 | [Transcriptional misregulation in cancer](../../../../D:%5C高通量测序结果%5CF14FTSSCKF1242_NEMpnqE%5Cannotation%5CKEGG%5CYK-Unigene.fa_map%5Cmap05202.html) | CL1072.Contig1_YK, CL1119.Contig1_YK, CL1119.Contig2_YK, CL1133.Contig1_YK, CL1133.Contig3_YK, CL1133.Contig5_YK, CL1184.Contig2_YK, CL1278.Contig1_YK, CL1278.Contig2_YK, CL1373.Contig1_YK, CL1373.Contig2_YK, CL1373.Contig3_YK, CL1516.Contig1_YK, CL1516.Contig2_YK, CL1538.Contig2_YK, CL1544.Contig1_YK, CL1544.Contig2_YK, CL1576.Contig1_YK, CL1576.Contig2_YK, CL1581.Contig1_YK, CL1581.Contig2_YK, CL1599.Contig1_YK, CL1599.Contig2_YK, CL1599.Contig3_YK, CL1605.Contig1_YK, CL1605.Contig2_YK, CL1689.Contig2_YK, CL1691.Contig1_YK, CL1691.Contig2_YK, CL1693.Contig1_YK, CL1693.Contig2_YK, CL1693.Contig3_YK, CL1693.Contig4_YK, CL1693.Contig5_YK, CL1740.Contig1_YK, CL1765.Contig1_YK, CL1765.Contig2_YK, CL1765.Contig3_YK, CL1862.Contig1_YK, CL1862.Contig2_YK, CL1898.Contig1_YK, CL1898.Contig2_YK, CL216.Contig1_YK, CL2254.Contig1_YK, CL2254.Contig2_YK, CL2254.Contig3_YK, CL2315.Contig1_YK, CL249.Contig1_YK, CL26.Contig1_YK, CL2672.Contig2_YK, CL2681.Contig1_YK, CL2800.Contig1_YK, CL2800.Contig2_YK, CL282.Contig1_YK, CL282.Contig2_YK, CL282.Contig3_YK, CL282.Contig4_YK, CL286.Contig1_YK, CL286.Contig2_YK, CL286.Contig3_YK, CL2889.Contig1_YK, CL2889.Contig2_YK, CL308.Contig1_YK, CL308.Contig3_YK, CL383.Contig10_YK, CL383.Contig11_YK, CL383.Contig12_YK, CL383.Contig19_YK, CL383.Contig1_YK, CL383.Contig20_YK, CL383.Contig21_YK, CL383.Contig22_YK, CL383.Contig2_YK, CL383.Contig3_YK, CL383.Contig9_YK, CL488.Contig1_YK, CL526.Contig1_YK, CL526.Contig2_YK, CL577.Contig1_YK, CL577.Contig2_YK, CL577.Contig3_YK, CL577.Contig4_YK, CL577.Contig5_YK, CL577.Contig6_YK, CL730.Contig1_YK, CL730.Contig2_YK, CL732.Contig1_YK, CL732.Contig2_YK, CL741.Contig2_YK, CL741.Contig3_YK, CL814.Contig1_YK, CL814.Contig2_YK, CL870.Contig1_YK, CL870.Contig3_YK, CL961.Contig7_YK, CL979.Contig1_YK, CL979.Contig2_YK, CL979.Contig3_YK, Unigene10074_YK, Unigene10156_YK, Unigene10207_YK, Unigene10430_YK, Unigene10431_YK, Unigene10432_YK, Unigene10439_YK, Unigene10440_YK, Unigene10441_YK, Unigene10502_YK, Unigene10504_YK, Unigene10659_YK, Unigene10857_YK, Unigene10899_YK, Unigene11093_YK, Unigene11204_YK, Unigene11212_YK, Unigene11313_YK, Unigene11448_YK, Unigene11536_YK, Unigene11537_YK, Unigene11718_YK, Unigene11719_YK, Unigene11851_YK, Unigene11916_YK, Unigene11918_YK, Unigene11972_YK, Unigene12813_YK, Unigene13097_YK, Unigene1331_YK, Unigene13334_YK, Unigene13388_YK, Unigene13424_YK, Unigene13470_YK, Unigene13518_YK, Unigene13582_YK, Unigene13603_YK, Unigene13796_YK, Unigene13912_YK, Unigene13954_YK, Unigene13964_YK, Unigene13981_YK, Unigene14257_YK, Unigene14302_YK, Unigene14348_YK, Unigene14496_YK, Unigene14541_YK, Unigene14617_YK, Unigene14628_YK, Unigene14644_YK, Unigene14742_YK, Unigene14749_YK, Unigene147_YK, Unigene14989_YK, Unigene15021_YK, Unigene15043_YK, Unigene15083_YK, Unigene15145_YK, Unigene15147_YK, Unigene15251_YK, Unigene15593_YK, Unigene15608_YK, Unigene15722_YK, Unigene15844_YK, Unigene15870_YK, Unigene16613_YK, Unigene16821_YK, Unigene17003_YK, Unigene17113_YK, Unigene18183_YK, Unigene18284_YK, Unigene18508_YK, Unigene18577_YK, Unigene18916_YK, Unigene19001_YK, Unigene19025_YK, Unigene2275_YK, Unigene2342_YK, Unigene2364_YK, Unigene2433_YK, Unigene2533_YK, Unigene2578_YK, Unigene2802_YK, Unigene2852_YK, Unigene2886_YK, Unigene2887_YK, Unigene3037_YK, Unigene3161_YK, Unigene3216_YK, Unigene3347_YK, Unigene3385_YK, Unigene3414_YK, Unigene3479_YK, Unigene3532_YK, Unigene3559_YK, Unigene3635_YK, Unigene3690_YK, Unigene3768_YK, Unigene3852_YK, Unigene3929_YK, Unigene3992_YK, Unigene4128_YK, Unigene4313_YK, Unigene4754_YK, Unigene4979_YK, Unigene5168_YK, Unigene5331_YK, Unigene5519_YK, Unigene5608_YK, Unigene5699_YK, Unigene5890_YK, Unigene5937_YK, Unigene6010_YK, Unigene6012_YK, Unigene6037_YK, Unigene6304_YK, Unigene6406_YK, Unigene6508_YK, Unigene6512_YK, Unigene6617_YK, Unigene665_YK, Unigene6845_YK, Unigene6846_YK, Unigene7021_YK, Unigene7181_YK, Unigene7391_YK, Unigene7436_YK, Unigene7590_YK, Unigene7610_YK, Unigene7611_YK, Unigene7944_YK, Unigene8067_YK, Unigene813_YK, Unigene8532_YK, Unigene8533_YK, Unigene8647_YK, Unigene8718_YK, Unigene8900_YK, Unigene9642_YK, Unigene9678_YK, Unigene9705_YK, Unigene9807_YK, Unigene9909_YK |
| 31 | [Endocytosis](../../../../D:%5C高通量测序结果%5CF14FTSSCKF1242_NEMpnqE%5Cannotation%5CKEGG%5CYK-Unigene.fa_map%5Cmap04144.html) | CL1.Contig1_YK, CL1.Contig2_YK, CL1008.Contig1_YK, CL1008.Contig2_YK, CL1119.Contig1_YK, CL1119.Contig2_YK, CL1143.Contig1_YK, CL1143.Contig2_YK, CL1275.Contig1_YK, CL1275.Contig2_YK, CL1292.Contig1_YK, CL1292.Contig2_YK, CL1659.Contig1_YK, CL166.Contig1_YK, CL166.Contig2_YK, CL1666.Contig1_YK, CL1666.Contig2_YK, CL1748.Contig1_YK, CL1790.Contig1_YK, CL1790.Contig2_YK, CL1790.Contig3_YK, CL1790.Contig4_YK, CL1814.Contig1_YK, CL1816.Contig1_YK, CL1816.Contig2_YK, CL198.Contig1_YK, CL198.Contig2_YK, CL198.Contig3_YK, CL2006.Contig1_YK, CL2006.Contig2_YK, CL2006.Contig3_YK, CL2110.Contig1_YK, CL2110.Contig2_YK, CL2149.Contig1_YK, CL2149.Contig2_YK, CL2238.Contig1_YK, CL2421.Contig1_YK, CL2421.Contig2_YK, CL2429.Contig1_YK, CL2429.Contig2_YK, CL2449.Contig1_YK, CL2505.Contig1_YK, CL2505.Contig2_YK, CL270.Contig1_YK, CL270.Contig2_YK, CL2720.Contig1_YK, CL2896.Contig1_YK, CL353.Contig1_YK, CL353.Contig3_YK, CL353.Contig6_YK, CL438.Contig2_YK, CL438.Contig3_YK, CL45.Contig1_YK, CL45.Contig4_YK, CL45.Contig5_YK, CL592.Contig2_YK, CL728.Contig1_YK, CL728.Contig2_YK, CL741.Contig2_YK, CL741.Contig3_YK, CL839.Contig1_YK, CL839.Contig2_YK, CL863.Contig2_YK, CL899.Contig2_YK, CL899.Contig3_YK, CL964.Contig1_YK, CL964.Contig2_YK, CL964.Contig3_YK, CL964.Contig4_YK, CL966.Contig1_YK, CL966.Contig2_YK, CL966.Contig3_YK, CL966.Contig4_YK, CL966.Contig5_YK, CL966.Contig6_YK, CL991.Contig1_YK, CL991.Contig2_YK, Unigene10439_YK, Unigene10440_YK, Unigene10441_YK, Unigene10488_YK, Unigene10558_YK, Unigene10568_YK, Unigene10604_YK, Unigene10658_YK, Unigene10694_YK, Unigene10927_YK, Unigene11155_YK, Unigene11194_YK, Unigene11215_YK, Unigene11274_YK, Unigene11289_YK, Unigene11299_YK, Unigene11332_YK, Unigene11344_YK, Unigene11435_YK, Unigene11497_YK, Unigene11605_YK, Unigene11606_YK, Unigene1225_YK, Unigene12569_YK, Unigene12570_YK, Unigene12667_YK, Unigene12784_YK, Unigene12866_YK, Unigene12887_YK, Unigene12911_YK, Unigene12993_YK, Unigene13137_YK, Unigene13154_YK, Unigene13192_YK, Unigene13222_YK, Unigene13238_YK, Unigene13267_YK, Unigene13348_YK, Unigene13433_YK, Unigene13454_YK, Unigene13535_YK, Unigene13543_YK, Unigene13606_YK, Unigene13636_YK, Unigene13642_YK, Unigene13676_YK, Unigene13692_YK, Unigene13723_YK, Unigene13733_YK, Unigene13774_YK, Unigene13836_YK, Unigene13840_YK, Unigene13896_YK, Unigene1393_YK, Unigene13954_YK, Unigene13985_YK, Unigene14016_YK, Unigene14220_YK, Unigene14231_YK, Unigene14327_YK, Unigene14397_YK, Unigene14403_YK, Unigene14430_YK, Unigene14512_YK, Unigene14582_YK, Unigene14589_YK, Unigene14647_YK, Unigene14650_YK, Unigene14657_YK, Unigene14661_YK, Unigene14729_YK, Unigene14825_YK, Unigene14942_YK, Unigene15014_YK, Unigene15076_YK, Unigene15084_YK, Unigene15212_YK, Unigene15246_YK, Unigene15247_YK, Unigene15621_YK, Unigene15698_YK, Unigene15752_YK, Unigene15992_YK, Unigene16041_YK, Unigene1611_YK, Unigene1656_YK, Unigene1657_YK, Unigene16869_YK, Unigene1719_YK, Unigene17614_YK, Unigene17725_YK, Unigene19055_YK, Unigene1919_YK, Unigene2273_YK, Unigene2634_YK, Unigene2652_YK, Unigene2704_YK, Unigene27_YK, Unigene2930_YK, Unigene3045_YK, Unigene3056_YK, Unigene3073_YK, Unigene3074_YK, Unigene3096_YK, Unigene3173_YK, Unigene3293_YK, Unigene3321_YK, Unigene3350_YK, Unigene3357_YK, Unigene3360_YK, Unigene3416_YK, Unigene3588_YK, Unigene3761_YK, Unigene3769_YK, Unigene3936_YK, Unigene3958_YK, Unigene4066_YK, Unigene4067_YK, Unigene4068_YK, Unigene4203_YK, Unigene4304_YK, Unigene4330_YK, Unigene4423_YK, Unigene4435_YK, Unigene4471_YK, Unigene4494_YK, Unigene4660_YK, Unigene4749_YK, Unigene4825_YK, Unigene4975_YK, Unigene5254_YK, Unigene5327_YK, Unigene5334_YK, Unigene5362_YK, Unigene5511_YK, Unigene5699_YK, Unigene5890_YK, Unigene6027_YK, Unigene6365_YK, Unigene6407_YK, Unigene6444_YK, Unigene6920_YK, Unigene6971_YK, Unigene7755_YK, Unigene7837_YK, Unigene7838_YK, Unigene7839_YK, Unigene7840_YK, Unigene7959_YK, Unigene8065_YK, Unigene8583_YK, Unigene8718_YK, Unigene8835_YK, Unigene8905_YK, Unigene8933_YK, Unigene8934_YK, Unigene9323_YK, Unigene9388_YK, Unigene9480_YK, Unigene9649_YK, Unigene9678_YK, Unigene9732_YK, Unigene9847_YK |
| 32 | [Chemokine signaling pathway](../../../../D:%5C高通量测序结果%5CF14FTSSCKF1242_NEMpnqE%5Cannotation%5CKEGG%5CYK-Unigene.fa_map%5Cmap04062.html) | CL1088.Contig1_YK, CL1088.Contig2_YK, CL1381.Contig1_YK, CL1381.Contig2_YK, CL15.Contig10_YK, CL15.Contig11_YK, CL15.Contig12_YK, CL15.Contig13_YK, CL15.Contig14_YK, CL15.Contig15_YK, CL15.Contig16_YK, CL15.Contig17_YK, CL15.Contig18_YK, CL15.Contig19_YK, CL15.Contig1_YK, CL15.Contig20_YK, CL15.Contig21_YK, CL15.Contig22_YK, CL15.Contig23_YK, CL15.Contig24_YK, CL15.Contig2_YK, CL15.Contig3_YK, CL15.Contig4_YK, CL15.Contig5_YK, CL15.Contig6_YK, CL15.Contig7_YK, CL15.Contig8_YK, CL15.Contig9_YK, CL1505.Contig1_YK, CL1505.Contig2_YK, CL1577.Contig1_YK, CL1577.Contig2_YK, CL1577.Contig3_YK, CL1577.Contig4_YK, CL1602.Contig1_YK, CL1602.Contig2_YK, CL1659.Contig1_YK, CL166.Contig1_YK, CL166.Contig2_YK, CL1790.Contig1_YK, CL1790.Contig2_YK, CL1790.Contig3_YK, CL1790.Contig4_YK, CL1816.Contig1_YK, CL1816.Contig2_YK, CL2002.Contig1_YK, CL2002.Contig2_YK, CL2002.Contig3_YK, CL2002.Contig4_YK, CL2002.Contig5_YK, CL2006.Contig1_YK, CL2006.Contig2_YK, CL2006.Contig3_YK, CL215.Contig1_YK, CL2180.Contig1_YK, CL2180.Contig2_YK, CL2265.Contig1_YK, CL2265.Contig2_YK, CL2428.Contig1_YK, CL2428.Contig2_YK, CL255.Contig1_YK, CL255.Contig2_YK, CL255.Contig3_YK, CL255.Contig4_YK, CL255.Contig5_YK, CL255.Contig6_YK, CL255.Contig7_YK, CL255.Contig8_YK, CL2612.Contig1_YK, CL2612.Contig2_YK, CL2668.Contig1_YK, CL2668.Contig2_YK, CL2792.Contig1_YK, CL2800.Contig1_YK, CL2800.Contig2_YK, CL2887.Contig2_YK, CL307.Contig10_YK, CL307.Contig11_YK, CL307.Contig12_YK, CL307.Contig1_YK, CL307.Contig2_YK, CL307.Contig3_YK, CL307.Contig4_YK, CL307.Contig5_YK, CL307.Contig6_YK, CL307.Contig7_YK, CL307.Contig8_YK, CL307.Contig9_YK, CL349.Contig1_YK, CL349.Contig2_YK, CL349.Contig4_YK, CL577.Contig1_YK, CL577.Contig2_YK, CL577.Contig5_YK, CL591.Contig1_YK, CL591.Contig2_YK, CL591.Contig3_YK, CL591.Contig4_YK, CL591.Contig5_YK, CL591.Contig6_YK, CL591.Contig7_YK, CL591.Contig8_YK, CL674.Contig10_YK, CL674.Contig3_YK, CL774.Contig1_YK, CL774.Contig2_YK, CL79.Contig1_YK, CL79.Contig2_YK, CL79.Contig3_YK, CL79.Contig4_YK, CL79.Contig5_YK, CL79.Contig6_YK, CL79.Contig7_YK, CL79.Contig8_YK, CL832.Contig1_YK, CL884.Contig1_YK, CL884.Contig3_YK, Unigene10072_YK, Unigene10197_YK, Unigene10198_YK, Unigene10558_YK, Unigene10627_YK, Unigene107_YK, Unigene10908_YK, Unigene10940_YK, Unigene10972_YK, Unigene10993_YK, Unigene11093_YK, Unigene11335_YK, Unigene11344_YK, Unigene12216_YK, Unigene12218_YK, Unigene12219_YK, Unigene12292_YK, Unigene12293_YK, Unigene12451_YK, Unigene12636_YK, Unigene12638_YK, Unigene12639_YK, Unigene12866_YK, Unigene12887_YK, Unigene13203_YK, Unigene13560_YK, Unigene13571_YK, Unigene13674_YK, Unigene13692_YK, Unigene13723_YK, Unigene13969_YK, Unigene13977_YK, Unigene13985_YK, Unigene14080_YK, Unigene14105_YK, Unigene14159_YK, Unigene14254_YK, Unigene14345_YK, Unigene14507_YK, Unigene15247_YK, Unigene15497_YK, Unigene15561_YK, Unigene15714_YK, Unigene15795_YK, Unigene158_YK, Unigene15910_YK, Unigene16250_YK, Unigene16406_YK, Unigene16409_YK, Unigene17370_YK, Unigene179_YK, Unigene18164_YK, Unigene1919_YK, Unigene2231_YK, Unigene2289_YK, Unigene24_YK, Unigene258_YK, Unigene2791_YK, Unigene2899_YK, Unigene2949_YK, Unigene3286_YK, Unigene3293_YK, Unigene331_YK, Unigene3357_YK, Unigene3483_YK, Unigene372_YK, Unigene3885_YK, Unigene3937_YK, Unigene4203_YK, Unigene4252_YK, Unigene4396_YK, Unigene4494_YK, Unigene4583_YK, Unigene4680_YK, Unigene4761_YK, Unigene4861_YK, Unigene4_YK, Unigene5066_YK, Unigene5191_YK, Unigene5219_YK, Unigene5438_YK, Unigene5476_YK, Unigene5477_YK, Unigene5642_YK, Unigene6066_YK, Unigene6229_YK, Unigene6230_YK, Unigene6231_YK, Unigene6309_YK, Unigene6310_YK, Unigene6362_YK, Unigene6407_YK, Unigene6553_YK, Unigene6561_YK, Unigene6664_YK, Unigene6971_YK, Unigene7142_YK, Unigene7525_YK, Unigene7577_YK, Unigene7590_YK, Unigene7816_YK, Unigene8065_YK, Unigene8323_YK, Unigene8324_YK, Unigene8325_YK, Unigene8378_YK, Unigene8569_YK, Unigene8570_YK, Unigene8583_YK, Unigene8892_YK, Unigene8893_YK, Unigene9036_YK, Unigene9323_YK, Unigene9388_YK, Unigene9710_YK, Unigene9968_YK, Unigene9969_YK |
| 33 | [Glutamatergic synapse](../../../../D:%5C高通量测序结果%5CF14FTSSCKF1242_NEMpnqE%5Cannotation%5CKEGG%5CYK-Unigene.fa_map%5Cmap04724.html) | CL1088.Contig1_YK, CL1088.Contig2_YK, CL1205.Contig1_YK, CL1205.Contig2_YK, CL1205.Contig3_YK, CL1205.Contig4_YK, CL1469.Contig1_YK, CL15.Contig10_YK, CL15.Contig11_YK, CL15.Contig12_YK, CL15.Contig13_YK, CL15.Contig14_YK, CL15.Contig15_YK, CL15.Contig16_YK, CL15.Contig17_YK, CL15.Contig18_YK, CL15.Contig19_YK, CL15.Contig1_YK, CL15.Contig20_YK, CL15.Contig21_YK, CL15.Contig22_YK, CL15.Contig23_YK, CL15.Contig24_YK, CL15.Contig2_YK, CL15.Contig3_YK, CL15.Contig4_YK, CL15.Contig5_YK, CL15.Contig6_YK, CL15.Contig7_YK, CL15.Contig8_YK, CL15.Contig9_YK, CL166.Contig1_YK, CL166.Contig2_YK, CL1689.Contig2_YK, CL1896.Contig1_YK, CL1896.Contig2_YK, CL1896.Contig3_YK, CL1969.Contig1_YK, CL1969.Contig2_YK, CL2117.Contig1_YK, CL2117.Contig2_YK, CL2135.Contig1_YK, CL2135.Contig2_YK, CL2240.Contig1_YK, CL2240.Contig2_YK, CL2265.Contig1_YK, CL2265.Contig2_YK, CL2316.Contig1_YK, CL2316.Contig2_YK, CL255.Contig1_YK, CL255.Contig2_YK, CL255.Contig3_YK, CL255.Contig4_YK, CL255.Contig5_YK, CL255.Contig6_YK, CL255.Contig7_YK, CL255.Contig8_YK, CL2635.Contig1_YK, CL2635.Contig2_YK, CL2672.Contig1_YK, CL2799.Contig1_YK, CL2799.Contig2_YK, CL295.Contig10_YK, CL295.Contig2_YK, CL295.Contig3_YK, CL295.Contig4_YK, CL295.Contig6_YK, CL295.Contig8_YK, CL295.Contig9_YK, CL307.Contig10_YK, CL307.Contig11_YK, CL307.Contig12_YK, CL307.Contig1_YK, CL307.Contig2_YK, CL307.Contig3_YK, CL307.Contig4_YK, CL307.Contig5_YK, CL307.Contig6_YK, CL307.Contig7_YK, CL307.Contig8_YK, CL307.Contig9_YK, CL431.Contig1_YK, CL431.Contig2_YK, CL431.Contig3_YK, CL591.Contig1_YK, CL591.Contig2_YK, CL591.Contig3_YK, CL591.Contig4_YK, CL591.Contig5_YK, CL591.Contig6_YK, CL591.Contig7_YK, CL591.Contig8_YK, CL605.Contig1_YK, CL605.Contig2_YK, CL605.Contig3_YK, CL605.Contig4_YK, CL766.Contig1_YK, CL766.Contig2_YK, CL766.Contig3_YK, CL79.Contig1_YK, CL79.Contig2_YK, CL79.Contig3_YK, CL79.Contig4_YK, CL79.Contig5_YK, CL79.Contig6_YK, CL79.Contig7_YK, CL79.Contig8_YK, CL838.Contig1_YK, CL838.Contig2_YK, CL838.Contig3_YK, CL838.Contig4_YK, CL838.Contig5_YK, CL838.Contig8_YK, CL884.Contig1_YK, CL884.Contig3_YK, CL924.Contig10_YK, CL924.Contig11_YK, CL924.Contig12_YK, CL924.Contig1_YK, CL924.Contig2_YK, CL924.Contig3_YK, CL924.Contig4_YK, CL924.Contig5_YK, CL924.Contig6_YK, CL924.Contig7_YK, CL924.Contig8_YK, CL924.Contig9_YK, Unigene10007_YK, Unigene10096_YK, Unigene10491_YK, Unigene10492_YK, Unigene10493_YK, Unigene10546_YK, Unigene10547_YK, Unigene10614_YK, Unigene10627_YK, Unigene10766_YK, Unigene10767_YK, Unigene10829_YK, Unigene10908_YK, Unigene11167_YK, Unigene11344_YK, Unigene11889_YK, Unigene12072_YK, Unigene12216_YK, Unigene12219_YK, Unigene1223_YK, Unigene12292_YK, Unigene12293_YK, Unigene12348_YK, Unigene12351_YK, Unigene12560_YK, Unigene12561_YK, Unigene12563_YK, Unigene12564_YK, Unigene12622_YK, Unigene12636_YK, Unigene12638_YK, Unigene12639_YK, Unigene13237_YK, Unigene1335_YK, Unigene1356_YK, Unigene13674_YK, Unigene13877_YK, Unigene13896_YK, Unigene13969_YK, Unigene14080_YK, Unigene14159_YK, Unigene14507_YK, Unigene14708_YK, Unigene14922_YK, Unigene158_YK, Unigene16000_YK, Unigene16120_YK, Unigene16406_YK, Unigene16439_YK, Unigene17435_YK, Unigene17811_YK, Unigene18164_YK, Unigene181_YK, Unigene1964_YK, Unigene2148_YK, Unigene2231_YK, Unigene2289_YK, Unigene258_YK, Unigene319_YK, Unigene3200_YK, Unigene331_YK, Unigene3585_YK, Unigene3596_YK, Unigene3885_YK, Unigene3937_YK, Unigene4062_YK, Unigene4190_YK, Unigene4252_YK, Unigene4445_YK, Unigene4505_YK, Unigene4564_YK, Unigene4565_YK, Unigene4772_YK, Unigene4778_YK, Unigene5438_YK, Unigene5476_YK, Unigene5477_YK, Unigene5857_YK, Unigene5924_YK, Unigene6006_YK, Unigene6007_YK, Unigene6206_YK, Unigene663_YK, Unigene6670_YK, Unigene6697_YK, Unigene6980_YK, Unigene7028_YK, Unigene7095_YK, Unigene735_YK, Unigene7382_YK, Unigene7390_YK, Unigene7391_YK, Unigene7412_YK, Unigene7525_YK, Unigene765_YK, Unigene8101_YK, Unigene8485_YK, Unigene8486_YK, Unigene8487_YK, Unigene8557_YK, Unigene904_YK, Unigene905_YK, Unigene9225_YK, Unigene9518_YK, Unigene9710_YK |
| 34 | [Salmonella infection](../../../../D:%5C高通量测序结果%5CF14FTSSCKF1242_NEMpnqE%5Cannotation%5CKEGG%5CYK-Unigene.fa_map%5Cmap05132.html) | CL1019.Contig1_YK, CL1019.Contig2_YK, CL1019.Contig3_YK, CL1019.Contig4_YK, CL1156.Contig3_YK, CL1221.Contig1_YK, CL1221.Contig2_YK, CL1221.Contig3_YK, CL124.Contig1_YK, CL124.Contig2_YK, CL124.Contig3_YK, CL1303.Contig1_YK, CL1303.Contig2_YK, CL1303.Contig3_YK, CL1381.Contig1_YK, CL1381.Contig2_YK, CL1486.Contig2_YK, CL1556.Contig1_YK, CL1556.Contig2_YK, CL1577.Contig1_YK, CL1577.Contig2_YK, CL1577.Contig3_YK, CL1577.Contig4_YK, CL1578.Contig2_YK, CL1591.Contig1_YK, CL1758.Contig1_YK, CL1758.Contig2_YK, CL1777.Contig1_YK, CL1777.Contig2_YK, CL181.Contig1_YK, CL181.Contig2_YK, CL1814.Contig1_YK, CL1814.Contig2_YK, CL1817.Contig1_YK, CL1817.Contig2_YK, CL1920.Contig1_YK, CL1920.Contig2_YK, CL2168.Contig1_YK, CL2560.Contig1_YK, CL2560.Contig2_YK, CL2624.Contig1_YK, CL2624.Contig2_YK, CL2648.Contig1_YK, CL2669.Contig1_YK, CL2669.Contig2_YK, CL2669.Contig3_YK, CL2669.Contig4_YK, CL2669.Contig5_YK, CL2717.Contig2_YK, CL2774.Contig2_YK, CL2793.Contig1_YK, CL2793.Contig2_YK, CL2887.Contig2_YK, CL2905.Contig1_YK, CL2905.Contig2_YK, CL344.Contig3_YK, CL369.Contig2_YK, CL369.Contig5_YK, CL369.Contig8_YK, CL37.Contig2_YK, CL411.Contig1_YK, CL411.Contig2_YK, CL411.Contig3_YK, CL43.Contig1_YK, CL43.Contig2_YK, CL487.Contig1_YK, CL487.Contig2_YK, CL599.Contig1_YK, CL599.Contig2_YK, CL674.Contig10_YK, CL674.Contig3_YK, CL832.Contig1_YK, CL84.Contig2_YK, CL84.Contig3_YK, CL91.Contig1_YK, CL91.Contig2_YK, CL91.Contig3_YK, CL91.Contig4_YK, CL91.Contig5_YK, CL91.Contig6_YK, CL91.Contig7_YK, CL95.Contig2_YK, Unigene10251_YK, Unigene10291_YK, Unigene10355_YK, Unigene10695_YK, Unigene10775_YK, Unigene10927_YK, Unigene11532_YK, Unigene11884_YK, Unigene12027_YK, Unigene12334_YK, Unigene12335_YK, Unigene12336_YK, Unigene12337_YK, Unigene12426_YK, Unigene12491_YK, Unigene12492_YK, Unigene12493_YK, Unigene12494_YK, Unigene12532_YK, Unigene12898_YK, Unigene12899_YK, Unigene13245_YK, Unigene13560_YK, Unigene13570_YK, Unigene13598_YK, Unigene13652_YK, Unigene13674_YK, Unigene13704_YK, Unigene13720_YK, Unigene13723_YK, Unigene13811_YK, Unigene13890_YK, Unigene13920_YK, Unigene14005_YK, Unigene14016_YK, Unigene14035_YK, Unigene14254_YK, Unigene14296_YK, Unigene14327_YK, Unigene14403_YK, Unigene14420_YK, Unigene14487_YK, Unigene14600_YK, Unigene14661_YK, Unigene14713_YK, Unigene14729_YK, Unigene14747_YK, Unigene14929_YK, Unigene14966_YK, Unigene15034_YK, Unigene15271_YK, Unigene15308_YK, Unigene15315_YK, Unigene15381_YK, Unigene1554_YK, Unigene15795_YK, Unigene15961_YK, Unigene16018_YK, Unigene16105_YK, Unigene16250_YK, Unigene16458_YK, Unigene16741_YK, Unigene16862_YK, Unigene16869_YK, Unigene16898_YK, Unigene1700_YK, Unigene17236_YK, Unigene17458_YK, Unigene17660_YK, Unigene17790_YK, Unigene1787_YK, Unigene1788_YK, Unigene179_YK, Unigene18316_YK, Unigene18676_YK, Unigene18931_YK, Unigene18969_YK, Unigene2137_YK, Unigene2207_YK, Unigene2276_YK, Unigene2620_YK, Unigene2719_YK, Unigene2849_YK, Unigene2892_YK, Unigene3026_YK, Unigene3034_YK, Unigene3067_YK, Unigene3188_YK, Unigene3189_YK, Unigene3268_YK, Unigene3271_YK, Unigene3279_YK, Unigene3629_YK, Unigene3739_YK, Unigene3740_YK, Unigene3769_YK, Unigene3850_YK, Unigene3906_YK, Unigene3916_YK, Unigene4074_YK, Unigene4180_YK, Unigene4203_YK, Unigene4315_YK, Unigene4380_YK, Unigene4396_YK, Unigene4494_YK, Unigene4583_YK, Unigene4747_YK, Unigene476_YK, Unigene4825_YK, Unigene4912_YK, Unigene5191_YK, Unigene5592_YK, Unigene6000_YK, Unigene6001_YK, Unigene6002_YK, Unigene6146_YK, Unigene6309_YK, Unigene6310_YK, Unigene6340_YK, Unigene6341_YK, Unigene6350_YK, Unigene6351_YK, Unigene6363_YK, Unigene6469_YK, Unigene6553_YK, Unigene6561_YK, Unigene6600_YK, Unigene6679_YK, Unigene6814_YK, Unigene6850_YK, Unigene7053_YK, Unigene7054_YK, Unigene7191_YK, Unigene737_YK, Unigene738_YK, Unigene7662_YK, Unigene7684_YK, Unigene801_YK, Unigene836_YK, Unigene8483_YK, Unigene8495_YK, Unigene8755_YK, Unigene8997_YK, Unigene9071_YK, Unigene9373_YK, Unigene9504_YK, Unigene9505_YK, Unigene9937_YK, Unigene9989_YK |
| 35 | [Serotonergic synapse](../../../../D:%5C高通量测序结果%5CF14FTSSCKF1242_NEMpnqE%5Cannotation%5CKEGG%5CYK-Unigene.fa_map%5Cmap04726.html) | CL1063.Contig1_YK, CL1063.Contig2_YK, CL1088.Contig1_YK, CL1088.Contig2_YK, CL1304.Contig1_YK, CL1304.Contig2_YK, CL1469.Contig1_YK, CL15.Contig10_YK, CL15.Contig11_YK, CL15.Contig12_YK, CL15.Contig13_YK, CL15.Contig14_YK, CL15.Contig15_YK, CL15.Contig16_YK, CL15.Contig17_YK, CL15.Contig18_YK, CL15.Contig19_YK, CL15.Contig1_YK, CL15.Contig20_YK, CL15.Contig21_YK, CL15.Contig22_YK, CL15.Contig23_YK, CL15.Contig24_YK, CL15.Contig2_YK, CL15.Contig3_YK, CL15.Contig4_YK, CL15.Contig5_YK, CL15.Contig6_YK, CL15.Contig7_YK, CL15.Contig8_YK, CL15.Contig9_YK, CL1602.Contig1_YK, CL1602.Contig2_YK, CL1689.Contig2_YK, CL1691.Contig1_YK, CL1691.Contig2_YK, CL1838.Contig1_YK, CL1838.Contig2_YK, CL1919.Contig1_YK, CL1919.Contig2_YK, CL1933.Contig1_YK, CL1933.Contig2_YK, CL1961.Contig1_YK, CL1968.Contig1_YK, CL1968.Contig2_YK, CL1969.Contig1_YK, CL1969.Contig2_YK, CL2012.Contig1_YK, CL2012.Contig2_YK, CL2265.Contig1_YK, CL2265.Contig2_YK, CL2337.Contig1_YK, CL2337.Contig2_YK, CL2379.Contig2_YK, CL2407.Contig1_YK, CL255.Contig1_YK, CL255.Contig2_YK, CL255.Contig3_YK, CL255.Contig4_YK, CL255.Contig5_YK, CL255.Contig6_YK, CL255.Contig7_YK, CL255.Contig8_YK, CL2681.Contig1_YK, CL278.Contig3_YK, CL278.Contig4_YK, CL2798.Contig1_YK, CL2798.Contig2_YK, CL2852.Contig1_YK, CL2852.Contig2_YK, CL295.Contig10_YK, CL295.Contig2_YK, CL295.Contig3_YK, CL295.Contig4_YK, CL295.Contig6_YK, CL295.Contig8_YK, CL295.Contig9_YK, CL307.Contig10_YK, CL307.Contig11_YK, CL307.Contig12_YK, CL307.Contig1_YK, CL307.Contig2_YK, CL307.Contig3_YK, CL307.Contig4_YK, CL307.Contig5_YK, CL307.Contig6_YK, CL307.Contig7_YK, CL307.Contig8_YK, CL307.Contig9_YK, CL338.Contig2_YK, CL356.Contig2_YK, CL526.Contig1_YK, CL526.Contig2_YK, CL591.Contig1_YK, CL591.Contig2_YK, CL591.Contig3_YK, CL591.Contig4_YK, CL591.Contig5_YK, CL591.Contig6_YK, CL591.Contig7_YK, CL591.Contig8_YK, CL838.Contig1_YK, CL838.Contig2_YK, CL838.Contig3_YK, CL838.Contig4_YK, CL838.Contig5_YK, CL838.Contig8_YK, CL884.Contig1_YK, CL884.Contig3_YK, CL909.Contig2_YK, CL928.Contig1_YK, CL928.Contig2_YK, Unigene10028_YK, Unigene10029_YK, Unigene10031_YK, Unigene10209_YK, Unigene10210_YK, Unigene10211_YK, Unigene10408_YK, Unigene10445_YK, Unigene10627_YK, Unigene10790_YK, Unigene10908_YK, Unigene11167_YK, Unigene11200_YK, Unigene11201_YK, Unigene11461_YK, Unigene11889_YK, Unigene11890_YK, Unigene12216_YK, Unigene12219_YK, Unigene1223_YK, Unigene12451_YK, Unigene12522_YK, Unigene12560_YK, Unigene12561_YK, Unigene12563_YK, Unigene12564_YK, Unigene12636_YK, Unigene12638_YK, Unigene12639_YK, Unigene12743_YK, Unigene13135_YK, Unigene13237_YK, Unigene13424_YK, Unigene13674_YK, Unigene13969_YK, Unigene14080_YK, Unigene14159_YK, Unigene14345_YK, Unigene14507_YK, Unigene14581_YK, Unigene15454_YK, Unigene15784_YK, Unigene158_YK, Unigene15910_YK, Unigene16000_YK, Unigene16094_YK, Unigene16420_YK, Unigene16613_YK, Unigene16638_YK, Unigene18164_YK, Unigene18798_YK, Unigene1964_YK, Unigene2148_YK, Unigene2501_YK, Unigene258_YK, Unigene2630_YK, Unigene3200_YK, Unigene331_YK, Unigene3596_YK, Unigene3727_YK, Unigene3885_YK, Unigene3929_YK, Unigene3937_YK, Unigene397_YK, Unigene4190_YK, Unigene4195_YK, Unigene4252_YK, Unigene4419_YK, Unigene4505_YK, Unigene4564_YK, Unigene4565_YK, Unigene4680_YK, Unigene4754_YK, Unigene4772_YK, Unigene4848_YK, Unigene4979_YK, Unigene4992_YK, Unigene5043_YK, Unigene5219_YK, Unigene5438_YK, Unigene5476_YK, Unigene5477_YK, Unigene5857_YK, Unigene5924_YK, Unigene6045_YK, Unigene6196_YK, Unigene6198_YK, Unigene6206_YK, Unigene6561_YK, Unigene6670_YK, Unigene6691_YK, Unigene6804_YK, Unigene6910_YK, Unigene6980_YK, Unigene7525_YK, Unigene7623_YK, Unigene765_YK, Unigene7822_YK, Unigene7823_YK, Unigene7824_YK, Unigene8101_YK, Unigene8144_YK, Unigene827_YK, Unigene8468_YK, Unigene8679_YK, Unigene8745_YK, Unigene8746_YK, Unigene904_YK, Unigene905_YK, Unigene9168_YK, Unigene9169_YK, Unigene9179_YK, Unigene9180_YK, Unigene9315_YK, Unigene9581_YK, Unigene9705_YK, Unigene9816_YK, Unigene9984_YK |
| 36 | [Vibrio cholerae infection](../../../../D:%5C高通量测序结果%5CF14FTSSCKF1242_NEMpnqE%5Cannotation%5CKEGG%5CYK-Unigene.fa_map%5Cmap05110.html) | CL1002.Contig1_YK, CL1002.Contig2_YK, CL1030.Contig1_YK, CL1030.Contig2_YK, CL1088.Contig1_YK, CL1088.Contig2_YK, CL1204.Contig2_YK, CL1204.Contig3_YK, CL1211.Contig2_YK, CL1211.Contig4_YK, CL1221.Contig1_YK, CL1221.Contig2_YK, CL1221.Contig3_YK, CL1286.Contig1_YK, CL1286.Contig2_YK, CL1286.Contig3_YK, CL1286.Contig4_YK, CL1286.Contig5_YK, CL1286.Contig6_YK, CL1286.Contig7_YK, CL1286.Contig8_YK, CL1362.Contig1_YK, CL1362.Contig2_YK, CL1362.Contig3_YK, CL1557.Contig1_YK, CL1557.Contig2_YK, CL1557.Contig3_YK, CL1674.Contig1_YK, CL1674.Contig2_YK, CL1826.Contig1_YK, CL1826.Contig2_YK, CL1858.Contig1_YK, CL1858.Contig2_YK, CL1951.Contig1_YK, CL1951.Contig2_YK, CL1963.Contig1_YK, CL1963.Contig2_YK, CL1998.Contig1_YK, CL1998.Contig3_YK, CL2020.Contig1_YK, CL2020.Contig2_YK, CL2037.Contig1_YK, CL2051.Contig1_YK, CL2051.Contig3_YK, CL2097.Contig2_YK, CL2097.Contig3_YK, CL2275.Contig1_YK, CL2275.Contig2_YK, CL2309.Contig1_YK, CL2313.Contig1_YK, CL2313.Contig2_YK, CL2313.Contig3_YK, CL2355.Contig2_YK, CL2506.Contig2_YK, CL255.Contig1_YK, CL255.Contig2_YK, CL255.Contig3_YK, CL255.Contig4_YK, CL255.Contig5_YK, CL255.Contig6_YK, CL255.Contig7_YK, CL255.Contig8_YK, CL2601.Contig1_YK, CL2601.Contig2_YK, CL2627.Contig1_YK, CL2905.Contig1_YK, CL2905.Contig2_YK, CL308.Contig4_YK, CL308.Contig5_YK, CL475.Contig1_YK, CL475.Contig2_YK, CL475.Contig3_YK, CL475.Contig4_YK, CL591.Contig1_YK, CL591.Contig2_YK, CL591.Contig3_YK, CL591.Contig4_YK, CL591.Contig5_YK, CL591.Contig6_YK, CL591.Contig7_YK, CL591.Contig8_YK, CL657.Contig1_YK, CL657.Contig2_YK, CL707.Contig1_YK, CL707.Contig2_YK, CL79.Contig1_YK, CL79.Contig2_YK, CL79.Contig3_YK, CL79.Contig4_YK, CL79.Contig5_YK, CL79.Contig6_YK, CL79.Contig7_YK, CL79.Contig8_YK, CL807.Contig1_YK, CL807.Contig2_YK, CL809.Contig1_YK, CL809.Contig2_YK, CL84.Contig2_YK, CL84.Contig3_YK, CL884.Contig1_YK, CL884.Contig3_YK, Unigene10017_YK, Unigene10224_YK, Unigene10262_YK, Unigene10263_YK, Unigene10269_YK, Unigene10270_YK, Unigene1045_YK, Unigene10634_YK, Unigene10914_YK, Unigene10997_YK, Unigene10998_YK, Unigene11101_YK, Unigene11179_YK, Unigene11214_YK, Unigene11439_YK, Unigene11452_YK, Unigene11765_YK, Unigene11818_YK, Unigene11830_YK, Unigene12013_YK, Unigene12182_YK, Unigene12216_YK, Unigene12219_YK, Unigene1223_YK, Unigene12242_YK, Unigene12281_YK, Unigene12292_YK, Unigene12293_YK, Unigene12555_YK, Unigene12588_YK, Unigene12602_YK, Unigene12734_YK, Unigene12777_YK, Unigene12823_YK, Unigene12824_YK, Unigene12898_YK, Unigene12899_YK, Unigene12984_YK, Unigene13046_YK, Unigene13074_YK, Unigene13083_YK, Unigene13109_YK, Unigene13210_YK, Unigene13232_YK, Unigene13237_YK, Unigene13274_YK, Unigene13405_YK, Unigene13464_YK, Unigene13527_YK, Unigene13572_YK, Unigene13720_YK, Unigene13756_YK, Unigene13811_YK, Unigene13963_YK, Unigene13989_YK, Unigene14019_YK, Unigene14473_YK, Unigene14929_YK, Unigene14964_YK, Unigene15160_YK, Unigene15251_YK, Unigene15461_YK, Unigene15847_YK, Unigene16678_YK, Unigene16724_YK, Unigene16777_YK, Unigene16940_YK, Unigene17973_YK, Unigene18172_YK, Unigene18316_YK, Unigene19144_YK, Unigene2330_YK, Unigene2366_YK, Unigene2722_YK, Unigene2849_YK, Unigene303_YK, Unigene3067_YK, Unigene3127_YK, Unigene331_YK, Unigene3381_YK, Unigene347_YK, Unigene3621_YK, Unigene3657_YK, Unigene4019_YK, Unigene4081_YK, Unigene41_YK, Unigene4713_YK, Unigene4900_YK, Unigene5065_YK, Unigene5741_YK, Unigene5844_YK, Unigene5863_YK, Unigene5999_YK, Unigene59_YK, Unigene6060_YK, Unigene6105_YK, Unigene6166_YK, Unigene6209_YK, Unigene6600_YK, Unigene6737_YK, Unigene6980_YK, Unigene69_YK, Unigene70_YK, Unigene7525_YK, Unigene8101_YK, Unigene8132_YK, Unigene8134_YK, Unigene8232_YK, Unigene8233_YK, Unigene8364_YK, Unigene8365_YK, Unigene8371_YK, Unigene8449_YK, Unigene8450_YK, Unigene8451_YK, Unigene8452_YK, Unigene8751_YK, Unigene88_YK, Unigene904_YK, Unigene905_YK, Unigene9155_YK, Unigene9356_YK, Unigene9357_YK, Unigene9358_YK, Unigene952_YK, Unigene9634_YK |
| 37 | [Pancreatic secretion](../../../../D:%5C高通量测序结果%5CF14FTSSCKF1242_NEMpnqE%5Cannotation%5CKEGG%5CYK-Unigene.fa_map%5Cmap04972.html) | CL1058.Contig1_YK, CL1058.Contig2_YK, CL1058.Contig3_YK, CL1058.Contig4_YK, CL1185.Contig1_YK, CL1185.Contig2_YK, CL1242.Contig1_YK, CL1242.Contig2_YK, CL1381.Contig1_YK, CL1381.Contig2_YK, CL1454.Contig1_YK, CL1454.Contig2_YK, CL1454.Contig3_YK, CL1454.Contig4_YK, CL1469.Contig1_YK, CL15.Contig10_YK, CL15.Contig11_YK, CL15.Contig12_YK, CL15.Contig13_YK, CL15.Contig14_YK, CL15.Contig15_YK, CL15.Contig16_YK, CL15.Contig17_YK, CL15.Contig18_YK, CL15.Contig19_YK, CL15.Contig1_YK, CL15.Contig20_YK, CL15.Contig21_YK, CL15.Contig22_YK, CL15.Contig23_YK, CL15.Contig24_YK, CL15.Contig2_YK, CL15.Contig3_YK, CL15.Contig4_YK, CL15.Contig5_YK, CL15.Contig6_YK, CL15.Contig7_YK, CL15.Contig8_YK, CL15.Contig9_YK, CL1627.Contig1_YK, CL1627.Contig2_YK, CL1627.Contig3_YK, CL1627.Contig4_YK, CL1630.Contig2_YK, CL1703.Contig1_YK, CL1703.Contig2_YK, CL1703.Contig3_YK, CL1816.Contig1_YK, CL1816.Contig2_YK, CL1858.Contig1_YK, CL1858.Contig2_YK, CL1969.Contig1_YK, CL1969.Contig2_YK, CL1990.Contig1_YK, CL1990.Contig2_YK, CL2006.Contig1_YK, CL2006.Contig2_YK, CL2006.Contig3_YK, CL2044.Contig1_YK, CL2044.Contig2_YK, CL2283.Contig1_YK, CL2283.Contig2_YK, CL2283.Contig3_YK, CL2283.Contig4_YK, CL2320.Contig1_YK, CL2820.Contig1_YK, CL2820.Contig2_YK, CL295.Contig10_YK, CL295.Contig2_YK, CL295.Contig3_YK, CL295.Contig4_YK, CL295.Contig6_YK, CL295.Contig8_YK, CL295.Contig9_YK, CL307.Contig10_YK, CL307.Contig11_YK, CL307.Contig12_YK, CL307.Contig1_YK, CL307.Contig2_YK, CL307.Contig3_YK, CL307.Contig4_YK, CL307.Contig5_YK, CL307.Contig6_YK, CL307.Contig7_YK, CL307.Contig8_YK, CL307.Contig9_YK, CL383.Contig17_YK, CL383.Contig18_YK, CL529.Contig1_YK, CL529.Contig2_YK, CL529.Contig3_YK, CL529.Contig4_YK, CL529.Contig5_YK, CL529.Contig6_YK, CL591.Contig1_YK, CL591.Contig2_YK, CL591.Contig3_YK, CL591.Contig4_YK, CL591.Contig5_YK, CL591.Contig6_YK, CL591.Contig7_YK, CL591.Contig8_YK, CL657.Contig1_YK, CL657.Contig2_YK, CL79.Contig1_YK, CL79.Contig2_YK, CL79.Contig3_YK, CL79.Contig4_YK, CL79.Contig5_YK, CL79.Contig6_YK, CL79.Contig7_YK, CL79.Contig8_YK, CL801.Contig1_YK, CL801.Contig2_YK, CL801.Contig3_YK, CL801.Contig4_YK, CL801.Contig5_YK, CL801.Contig6_YK, CL832.Contig1_YK, CL884.Contig1_YK, CL884.Contig3_YK, CL957.Contig1_YK, CL957.Contig2_YK, Unigene10001_YK, Unigene10006_YK, Unigene10078_YK, Unigene10437_YK, Unigene10887_YK, Unigene10888_YK, Unigene10908_YK, Unigene11167_YK, Unigene11173_YK, Unigene11174_YK, Unigene11496_YK, Unigene11937_YK, Unigene12182_YK, Unigene1223_YK, Unigene12292_YK, Unigene12293_YK, Unigene12478_YK, Unigene12560_YK, Unigene12561_YK, Unigene12563_YK, Unigene12564_YK, Unigene12636_YK, Unigene12638_YK, Unigene12639_YK, Unigene12777_YK, Unigene13055_YK, Unigene13237_YK, Unigene1324_YK, Unigene1332_YK, Unigene13571_YK, Unigene13750_YK, Unigene13985_YK, Unigene14048_YK, Unigene14100_YK, Unigene14692_YK, Unigene14954_YK, Unigene15719_YK, Unigene158_YK, Unigene15952_YK, Unigene16045_YK, Unigene16222_YK, Unigene16238_YK, Unigene16250_YK, Unigene16406_YK, Unigene16678_YK, Unigene16940_YK, Unigene19144_YK, Unigene1964_YK, Unigene2231_YK, Unigene2287_YK, Unigene2289_YK, Unigene254_YK, Unigene2563_YK, Unigene258_YK, Unigene2811_YK, Unigene2959_YK, Unigene352_YK, Unigene4042_YK, Unigene4190_YK, Unigene4203_YK, Unigene4252_YK, Unigene4494_YK, Unigene4505_YK, Unigene4658_YK, Unigene4659_YK, Unigene4705_YK, Unigene4706_YK, Unigene4713_YK, Unigene5334_YK, Unigene5476_YK, Unigene5477_YK, Unigene5857_YK, Unigene5924_YK, Unigene6206_YK, Unigene6670_YK, Unigene6980_YK, Unigene7119_YK, Unigene7525_YK, Unigene8101_YK, Unigene8587_YK, Unigene8589_YK, Unigene8590_YK, Unigene8813_YK, Unigene8933_YK, Unigene8934_YK, Unigene904_YK, Unigene905_YK, Unigene9323_YK, Unigene9356_YK, Unigene9357_YK, Unigene9358_YK, Unigene9388_YK, Unigene9594_YK, Unigene96_YK, Unigene9710_YK, Unigene9715_YK, Unigene9716_YK, Unigene9717_YK, Unigene9849_YK, Unigene9884_YK |
| 38 | [Herpes simplex infection](../../../../D:%5C高通量测序结果%5CF14FTSSCKF1242_NEMpnqE%5Cannotation%5CKEGG%5CYK-Unigene.fa_map%5Cmap05168.html) | CL1004.Contig1_YK, CL1004.Contig2_YK, CL1026.Contig1_YK, CL1026.Contig2_YK, CL1166.Contig2_YK, CL1328.Contig1_YK, CL1380.Contig1_YK, CL1412.Contig1_YK, CL1448.Contig1_YK, CL1448.Contig2_YK, CL1451.Contig1_YK, CL1451.Contig2_YK, CL1560.Contig1_YK, CL1560.Contig2_YK, CL1560.Contig3_YK, CL1563.Contig1_YK, CL1563.Contig2_YK, CL1637.Contig1_YK, CL1637.Contig2_YK, CL1637.Contig3_YK, CL1637.Contig4_YK, CL1637.Contig5_YK, CL1637.Contig6_YK, CL1637.Contig7_YK, CL1637.Contig8_YK, CL1758.Contig1_YK, CL1758.Contig2_YK, CL1795.Contig1_YK, CL204.Contig1_YK, CL204.Contig2_YK, CL2126.Contig1_YK, CL2126.Contig2_YK, CL2132.Contig1_YK, CL2132.Contig2_YK, CL2199.Contig1_YK, CL2199.Contig2_YK, CL2199.Contig3_YK, CL2294.Contig1_YK, CL2294.Contig2_YK, CL2336.Contig1_YK, CL2336.Contig2_YK, CL2364.Contig1_YK, CL2432.Contig1_YK, CL2432.Contig2_YK, CL2532.Contig1_YK, CL2532.Contig2_YK, CL26.Contig1_YK, CL2609.Contig2_YK, CL2637.Contig1_YK, CL2637.Contig2_YK, CL2705.Contig1_YK, CL2705.Contig2_YK, CL2705.Contig3_YK, CL2705.Contig4_YK, CL2793.Contig1_YK, CL2793.Contig2_YK, CL2855.Contig1_YK, CL2883.Contig1_YK, CL2887.Contig1_YK, CL2887.Contig2_YK, CL298.Contig1_YK, CL298.Contig2_YK, CL312.Contig1_YK, CL387.Contig1_YK, CL387.Contig2_YK, CL387.Contig3_YK, CL387.Contig4_YK, CL387.Contig5_YK, CL569.Contig1_YK, CL569.Contig2_YK, CL617.Contig1_YK, CL617.Contig2_YK, CL789.Contig2_YK, CL815.Contig1_YK, CL815.Contig2_YK, CL815.Contig3_YK, CL815.Contig4_YK, CL815.Contig5_YK, CL815.Contig6_YK, CL83.Contig4_YK, CL935.Contig1_YK, CL935.Contig2_YK, Unigene10012_YK, Unigene10464_YK, Unigene10659_YK, Unigene10665_YK, Unigene10919_YK, Unigene109_YK, Unigene11140_YK, Unigene11141_YK, Unigene11246_YK, Unigene11298_YK, Unigene11499_YK, Unigene11662_YK, Unigene11886_YK, Unigene11919_YK, Unigene12128_YK, Unigene12299_YK, Unigene12300_YK, Unigene12303_YK, Unigene12305_YK, Unigene12307_YK, Unigene12309_YK, Unigene12429_YK, Unigene12430_YK, Unigene12440_YK, Unigene12591_YK, Unigene12592_YK, Unigene12613_YK, Unigene1272_YK, Unigene12733_YK, Unigene12743_YK, Unigene12982_YK, Unigene13180_YK, Unigene13282_YK, Unigene13313_YK, Unigene13340_YK, Unigene13343_YK, Unigene13430_YK, Unigene13436_YK, Unigene13469_YK, Unigene13533_YK, Unigene13791_YK, Unigene13824_YK, Unigene13962_YK, Unigene14180_YK, Unigene14248_YK, Unigene14339_YK, Unigene14415_YK, Unigene14516_YK, Unigene14674_YK, Unigene14707_YK, Unigene14709_YK, Unigene14766_YK, Unigene14867_YK, Unigene14883_YK, Unigene14922_YK, Unigene15250_YK, Unigene15348_YK, Unigene15482_YK, Unigene15526_YK, Unigene16015_YK, Unigene16112_YK, Unigene1611_YK, Unigene16144_YK, Unigene16311_YK, Unigene16715_YK, Unigene17790_YK, Unigene179_YK, Unigene18679_YK, Unigene18811_YK, Unigene18931_YK, Unigene18943_YK, Unigene18972_YK, Unigene201_YK, Unigene2080_YK, Unigene2315_YK, Unigene237_YK, Unigene260_YK, Unigene2689_YK, Unigene2791_YK, Unigene2852_YK, Unigene2915_YK, Unigene3231_YK, Unigene3315_YK, Unigene3316_YK, Unigene337_YK, Unigene3399_YK, Unigene3424_YK, Unigene3430_YK, Unigene3441_YK, Unigene3448_YK, Unigene3699_YK, Unigene3887_YK, Unigene3888_YK, Unigene4166_YK, Unigene4308_YK, Unigene4411_YK, Unigene4648_YK, Unigene4689_YK, Unigene4690_YK, Unigene4794_YK, Unigene5136_YK, Unigene5196_YK, Unigene5233_YK, Unigene5385_YK, Unigene5386_YK, Unigene5467_YK, Unigene5468_YK, Unigene5495_YK, Unigene5558_YK, Unigene5586_YK, Unigene6046_YK, Unigene6334_YK, Unigene6367_YK, Unigene6368_YK, Unigene6508_YK, Unigene6769_YK, Unigene6879_YK, Unigene6880_YK, Unigene7152_YK, Unigene7180_YK, Unigene7370_YK, Unigene7427_YK, Unigene7607_YK, Unigene7669_YK, Unigene7755_YK, Unigene8378_YK, Unigene838_YK, Unigene8397_YK, Unigene8461_YK, Unigene8822_YK, Unigene8902_YK, Unigene9071_YK, Unigene9127_YK, Unigene9377_YK, Unigene9706_YK, Unigene994_YK, Unigene9985_YK |
| 39 | [ECM-receptor interaction](../../../../D:%5C高通量测序结果%5CF14FTSSCKF1242_NEMpnqE%5Cannotation%5CKEGG%5CYK-Unigene.fa_map%5Cmap04512.html) | CL1310.Contig3_YK, CL1310.Contig5_YK, CL1310.Contig6_YK, CL1384.Contig1_YK, CL1384.Contig2_YK, CL1406.Contig1_YK, CL1406.Contig2_YK, CL157.Contig1_YK, CL157.Contig2_YK, CL157.Contig3_YK, CL157.Contig4_YK, CL157.Contig6_YK, CL157.Contig7_YK, CL1591.Contig1_YK, CL1670.Contig2_YK, CL1734.Contig1_YK, CL1734.Contig2_YK, CL1734.Contig3_YK, CL1734.Contig4_YK, CL1751.Contig1_YK, CL1751.Contig2_YK, CL1885.Contig1_YK, CL1959.Contig2_YK, CL1998.Contig1_YK, CL2026.Contig1_YK, CL2026.Contig2_YK, CL2031.Contig1_YK, CL2031.Contig2_YK, CL2051.Contig1_YK, CL2051.Contig3_YK, CL2363.Contig1_YK, CL2363.Contig2_YK, CL2536.Contig1_YK, CL2561.Contig1_YK, CL2561.Contig2_YK, CL2562.Contig1_YK, CL2562.Contig2_YK, CL2751.Contig1_YK, CL2751.Contig2_YK, CL2792.Contig1_YK, CL2886.Contig1_YK, CL2886.Contig2_YK, CL344.Contig3_YK, CL37.Contig2_YK, CL472.Contig1_YK, CL472.Contig2_YK, CL49.Contig10_YK, CL49.Contig11_YK, CL49.Contig12_YK, CL49.Contig13_YK, CL49.Contig14_YK, CL49.Contig15_YK, CL49.Contig16_YK, CL49.Contig17_YK, CL49.Contig18_YK, CL49.Contig19_YK, CL49.Contig1_YK, CL49.Contig20_YK, CL49.Contig21_YK, CL49.Contig2_YK, CL49.Contig3_YK, CL49.Contig4_YK, CL49.Contig5_YK, CL49.Contig6_YK, CL49.Contig7_YK, CL49.Contig8_YK, CL49.Contig9_YK, CL502.Contig1_YK, CL502.Contig2_YK, CL564.Contig1_YK, CL647.Contig1_YK, CL647.Contig2_YK, CL760.Contig1_YK, CL760.Contig2_YK, CL760.Contig3_YK, CL760.Contig4_YK, CL760.Contig5_YK, CL760.Contig6_YK, CL760.Contig7_YK, CL855.Contig2_YK, CL917.Contig2_YK, CL917.Contig3_YK, CL917.Contig5_YK, CL917.Contig6_YK, CL917.Contig7_YK, CL917.Contig8_YK, Unigene10034_YK, Unigene10291_YK, Unigene10341_YK, Unigene10404_YK, Unigene10444_YK, Unigene10494_YK, Unigene10495_YK, Unigene10550_YK, Unigene10551_YK, Unigene10653_YK, Unigene10664_YK, Unigene11316_YK, Unigene11317_YK, Unigene11363_YK, Unigene11364_YK, Unigene11585_YK, Unigene11696_YK, Unigene11860_YK, Unigene11941_YK, Unigene12017_YK, Unigene12352_YK, Unigene12353_YK, Unigene12535_YK, Unigene12536_YK, Unigene12537_YK, Unigene12538_YK, Unigene12539_YK, Unigene12541_YK, Unigene12542_YK, Unigene12543_YK, Unigene12544_YK, Unigene12548_YK, Unigene12977_YK, Unigene12991_YK, Unigene13108_YK, Unigene13109_YK, Unigene13293_YK, Unigene13296_YK, Unigene14007_YK, Unigene14093_YK, Unigene14259_YK, Unigene14399_YK, Unigene14710_YK, Unigene14914_YK, Unigene15037_YK, Unigene15065_YK, Unigene15350_YK, Unigene15413_YK, Unigene15673_YK, Unigene15802_YK, Unigene15943_YK, Unigene1603_YK, Unigene1660_YK, Unigene16613_YK, Unigene16698_YK, Unigene17212_YK, Unigene17547_YK, Unigene17960_YK, Unigene17996_YK, Unigene18305_YK, Unigene18657_YK, Unigene18946_YK, Unigene18999_YK, Unigene19070_YK, Unigene2018_YK, Unigene2190_YK, Unigene2230_YK, Unigene2433_YK, Unigene2486_YK, Unigene257_YK, Unigene2666_YK, Unigene2768_YK, Unigene2799_YK, Unigene2869_YK, Unigene2991_YK, Unigene3127_YK, Unigene3195_YK, Unigene3198_YK, Unigene3204_YK, Unigene3224_YK, Unigene3591_YK, Unigene3660_YK, Unigene3718_YK, Unigene3728_YK, Unigene3828_YK, Unigene3965_YK, Unigene3966_YK, Unigene4054_YK, Unigene4055_YK, Unigene4060_YK, Unigene4087_YK, Unigene4268_YK, Unigene4465_YK, Unigene4576_YK, Unigene4769_YK, Unigene4781_YK, Unigene4900_YK, Unigene4924_YK, Unigene4963_YK, Unigene5202_YK, Unigene5603_YK, Unigene5851_YK, Unigene5874_YK, Unigene5925_YK, Unigene617_YK, Unigene6261_YK, Unigene6268_YK, Unigene6443_YK, Unigene6685_YK, Unigene6686_YK, Unigene6889_YK, Unigene6890_YK, Unigene6981_YK, Unigene7147_YK, Unigene7496_YK, Unigene7764_YK, Unigene7998_YK, Unigene8282_YK, Unigene8360_YK, Unigene8371_YK, Unigene8462_YK, Unigene8736_YK, Unigene8756_YK, Unigene8795_YK, Unigene8899_YK, Unigene8900_YK, Unigene9102_YK, Unigene9295_YK, Unigene9296_YK, Unigene9584_YK, Unigene9634_YK |
| 40 | [Melanogenesis](../../../../D:%5C高通量测序结果%5CF14FTSSCKF1242_NEMpnqE%5Cannotation%5CKEGG%5CYK-Unigene.fa_map%5Cmap04916.html) | CL1072.Contig1_YK, CL1072.Contig2_YK, CL1088.Contig1_YK, CL1088.Contig2_YK, CL112.Contig1_YK, CL112.Contig2_YK, CL112.Contig3_YK, CL112.Contig4_YK, CL112.Contig5_YK, CL112.Contig6_YK, CL112.Contig7_YK, CL112.Contig8_YK, CL1170.Contig1_YK, CL1170.Contig2_YK, CL1170.Contig3_YK, CL1170.Contig4_YK, CL15.Contig10_YK, CL15.Contig11_YK, CL15.Contig12_YK, CL15.Contig13_YK, CL15.Contig14_YK, CL15.Contig15_YK, CL15.Contig16_YK, CL15.Contig17_YK, CL15.Contig18_YK, CL15.Contig19_YK, CL15.Contig1_YK, CL15.Contig20_YK, CL15.Contig21_YK, CL15.Contig22_YK, CL15.Contig23_YK, CL15.Contig24_YK, CL15.Contig2_YK, CL15.Contig3_YK, CL15.Contig4_YK, CL15.Contig5_YK, CL15.Contig6_YK, CL15.Contig7_YK, CL15.Contig8_YK, CL15.Contig9_YK, CL1530.Contig1_YK, CL1530.Contig2_YK, CL1530.Contig3_YK, CL1530.Contig4_YK, CL1538.Contig1_YK, CL1538.Contig2_YK, CL1679.Contig1_YK, CL1679.Contig2_YK, CL1679.Contig3_YK, CL1714.Contig2_YK, CL1861.Contig1_YK, CL2336.Contig1_YK, CL2336.Contig2_YK, CL255.Contig1_YK, CL255.Contig2_YK, CL255.Contig3_YK, CL255.Contig4_YK, CL255.Contig5_YK, CL255.Contig6_YK, CL255.Contig7_YK, CL255.Contig8_YK, CL2593.Contig1_YK, CL2593.Contig2_YK, CL2593.Contig3_YK, CL2666.Contig1_YK, CL2666.Contig2_YK, CL2668.Contig1_YK, CL2668.Contig2_YK, CL2729.Contig1_YK, CL2866.Contig1_YK, CL307.Contig10_YK, CL307.Contig11_YK, CL307.Contig12_YK, CL307.Contig1_YK, CL307.Contig2_YK, CL307.Contig3_YK, CL307.Contig4_YK, CL307.Contig5_YK, CL307.Contig6_YK, CL307.Contig7_YK, CL307.Contig8_YK, CL307.Contig9_YK, CL334.Contig1_YK, CL334.Contig2_YK, CL387.Contig1_YK, CL387.Contig2_YK, CL387.Contig3_YK, CL387.Contig4_YK, CL387.Contig5_YK, CL568.Contig1_YK, CL568.Contig2_YK, CL591.Contig1_YK, CL591.Contig2_YK, CL591.Contig3_YK, CL591.Contig4_YK, CL591.Contig5_YK, CL591.Contig6_YK, CL591.Contig7_YK, CL591.Contig8_YK, CL615.Contig3_YK, CL615.Contig4_YK, CL615.Contig5_YK, CL615.Contig7_YK, CL720.Contig1_YK, CL720.Contig2_YK, CL774.Contig1_YK, CL774.Contig2_YK, CL79.Contig1_YK, CL79.Contig2_YK, CL79.Contig3_YK, CL79.Contig4_YK, CL79.Contig5_YK, CL79.Contig6_YK, CL79.Contig7_YK, CL79.Contig8_YK, CL814.Contig1_YK, CL814.Contig2_YK, CL83.Contig4_YK, CL884.Contig1_YK, CL884.Contig3_YK, CL902.Contig1_YK, CL902.Contig2_YK, Unigene10659_YK, Unigene10908_YK, Unigene10954_YK, Unigene11008_YK, Unigene11075_YK, Unigene12216_YK, Unigene12219_YK, Unigene1223_YK, Unigene12292_YK, Unigene12293_YK, Unigene12636_YK, Unigene12638_YK, Unigene12639_YK, Unigene13237_YK, Unigene13674_YK, Unigene13937_YK, Unigene13960_YK, Unigene13969_YK, Unigene14094_YK, Unigene14243_YK, Unigene14345_YK, Unigene14516_YK, Unigene14681_YK, Unigene15057_YK, Unigene15325_YK, Unigene15330_YK, Unigene15561_YK, Unigene15587_YK, Unigene15595_YK, Unigene15877_YK, Unigene158_YK, Unigene15910_YK, Unigene16406_YK, Unigene18164_YK, Unigene1964_YK, Unigene2148_YK, Unigene2231_YK, Unigene2289_YK, Unigene2550_YK, Unigene258_YK, Unigene26_YK, Unigene3200_YK, Unigene3219_YK, Unigene3318_YK, Unigene331_YK, Unigene335_YK, Unigene3596_YK, Unigene3626_YK, Unigene3937_YK, Unigene4034_YK, Unigene4252_YK, Unigene4564_YK, Unigene4565_YK, Unigene4680_YK, Unigene4772_YK, Unigene4_YK, Unigene5219_YK, Unigene5438_YK, Unigene5476_YK, Unigene5477_YK, Unigene5656_YK, Unigene5857_YK, Unigene5993_YK, Unigene6206_YK, Unigene6227_YK, Unigene6461_YK, Unigene6561_YK, Unigene6611_YK, Unigene6638_YK, Unigene6664_YK, Unigene6980_YK, Unigene6_YK, Unigene7307_YK, Unigene7308_YK, Unigene7525_YK, Unigene765_YK, Unigene7816_YK, Unigene8101_YK, Unigene8718_YK, Unigene904_YK, Unigene905_YK, Unigene9127_YK, Unigene9459_YK, Unigene9642_YK, Unigene9710_YK, Unigene9780_YK, Unigene9781_YK, Unigene9968_YK, Unigene9969_YK |
| 41 | [Cardiac muscle contraction](../../../../D:%5C高通量测序结果%5CF14FTSSCKF1242_NEMpnqE%5Cannotation%5CKEGG%5CYK-Unigene.fa_map%5Cmap04260.html) | CL1019.Contig1_YK, CL1019.Contig2_YK, CL1019.Contig3_YK, CL1019.Contig4_YK, CL1058.Contig1_YK, CL1058.Contig2_YK, CL1058.Contig3_YK, CL1058.Contig4_YK, CL1156.Contig3_YK, CL1185.Contig1_YK, CL1185.Contig2_YK, CL124.Contig1_YK, CL124.Contig2_YK, CL124.Contig3_YK, CL1247.Contig1_YK, CL1247.Contig2_YK, CL1260.Contig1_YK, CL1260.Contig2_YK, CL1260.Contig3_YK, CL1303.Contig1_YK, CL1303.Contig2_YK, CL1303.Contig3_YK, CL136.Contig1_YK, CL136.Contig3_YK, CL1486.Contig2_YK, CL1577.Contig1_YK, CL1577.Contig2_YK, CL1577.Contig3_YK, CL1577.Contig4_YK, CL1578.Contig2_YK, CL1591.Contig1_YK, CL1611.Contig1_YK, CL1611.Contig2_YK, CL1682.Contig1_YK, CL181.Contig1_YK, CL181.Contig2_YK, CL1891.Contig1_YK, CL1891.Contig2_YK, CL1920.Contig1_YK, CL1920.Contig2_YK, CL1931.Contig2_YK, CL2320.Contig1_YK, CL2560.Contig1_YK, CL2560.Contig2_YK, CL2626.Contig1_YK, CL2626.Contig2_YK, CL2648.Contig1_YK, CL2669.Contig1_YK, CL2669.Contig2_YK, CL2669.Contig3_YK, CL2669.Contig4_YK, CL2669.Contig5_YK, CL2774.Contig2_YK, CL2816.Contig1_YK, CL2905.Contig2_YK, CL344.Contig3_YK, CL367.Contig1_YK, CL369.Contig2_YK, CL369.Contig5_YK, CL369.Contig8_YK, CL37.Contig2_YK, CL487.Contig1_YK, CL487.Contig2_YK, CL599.Contig1_YK, CL599.Contig2_YK, CL801.Contig1_YK, CL801.Contig2_YK, CL801.Contig3_YK, CL801.Contig4_YK, CL801.Contig5_YK, CL801.Contig6_YK, CL838.Contig1_YK, CL838.Contig2_YK, CL838.Contig3_YK, CL838.Contig4_YK, CL838.Contig5_YK, CL838.Contig8_YK, CL91.Contig1_YK, CL91.Contig2_YK, CL91.Contig3_YK, CL91.Contig4_YK, CL91.Contig5_YK, CL91.Contig6_YK, CL91.Contig7_YK, CL95.Contig2_YK, CL957.Contig1_YK, CL957.Contig2_YK, Unigene10001_YK, Unigene10251_YK, Unigene10291_YK, Unigene10355_YK, Unigene10695_YK, Unigene10777_YK, Unigene10927_YK, Unigene10936_YK, Unigene11532_YK, Unigene11740_YK, Unigene11802_YK, Unigene11884_YK, Unigene11889_YK, Unigene12027_YK, Unigene12334_YK, Unigene12335_YK, Unigene12336_YK, Unigene12337_YK, Unigene12376_YK, Unigene12426_YK, Unigene12491_YK, Unigene12492_YK, Unigene12493_YK, Unigene12494_YK, Unigene12532_YK, Unigene12990_YK, Unigene13009_YK, Unigene13055_YK, Unigene13229_YK, Unigene13245_YK, Unigene1332_YK, Unigene13507_YK, Unigene13598_YK, Unigene13608_YK, Unigene13704_YK, Unigene13750_YK, Unigene13890_YK, Unigene13920_YK, Unigene14005_YK, Unigene14016_YK, Unigene14204_YK, Unigene14254_YK, Unigene14327_YK, Unigene14403_YK, Unigene14420_YK, Unigene14455_YK, Unigene14510_YK, Unigene14661_YK, Unigene14729_YK, Unigene14929_YK, Unigene14966_YK, Unigene15034_YK, Unigene15271_YK, Unigene15308_YK, Unigene15315_YK, Unigene15529_YK, Unigene15764_YK, Unigene15961_YK, Unigene16000_YK, Unigene16018_YK, Unigene16105_YK, Unigene16458_YK, Unigene16741_YK, Unigene16862_YK, Unigene16869_YK, Unigene17458_YK, Unigene18156_YK, Unigene19067_YK, Unigene19071_YK, Unigene19109_YK, Unigene2207_YK, Unigene2607_YK, Unigene2620_YK, Unigene2719_YK, Unigene2770_YK, Unigene2892_YK, Unigene3026_YK, Unigene3034_YK, Unigene3133_YK, Unigene3188_YK, Unigene3189_YK, Unigene3268_YK, Unigene3271_YK, Unigene3629_YK, Unigene3739_YK, Unigene3740_YK, Unigene3852_YK, Unigene3868_YK, Unigene3906_YK, Unigene3916_YK, Unigene4074_YK, Unigene4315_YK, Unigene4454_YK, Unigene4747_YK, Unigene4825_YK, Unigene5975_YK, Unigene6146_YK, Unigene6270_YK, Unigene6469_YK, Unigene6725_YK, Unigene6789_YK, Unigene6814_YK, Unigene6850_YK, Unigene7662_YK, Unigene7684_YK, Unigene836_YK, Unigene8577_YK, Unigene8578_YK, Unigene8587_YK, Unigene8589_YK, Unigene8590_YK, Unigene8755_YK, Unigene8762_YK, Unigene8997_YK, Unigene9373_YK, Unigene9594_YK, Unigene9715_YK, Unigene9716_YK, Unigene9717_YK, Unigene9884_YK, Unigene9903_YK, Unigene9904_YK, Unigene9937_YK, Unigene9989_YK |
| 42 | [Ubiquitin mediated proteolysis](../../../../D:%5C高通量测序结果%5CF14FTSSCKF1242_NEMpnqE%5Cannotation%5CKEGG%5CYK-Unigene.fa_map%5Cmap04120.html) | CL1004.Contig1_YK, CL1004.Contig2_YK, CL1029.Contig1_YK, CL110.Contig1_YK, CL110.Contig2_YK, CL1117.Contig1_YK, CL1412.Contig1_YK, CL1412.Contig2_YK, CL1412.Contig3_YK, CL1437.Contig1_YK, CL1437.Contig3_YK, CL155.Contig1_YK, CL155.Contig2_YK, CL1659.Contig1_YK, CL1659.Contig2_YK, CL174.Contig1_YK, CL1765.Contig1_YK, CL1765.Contig2_YK, CL1765.Contig3_YK, CL179.Contig1_YK, CL179.Contig2_YK, CL179.Contig4_YK, CL179.Contig5_YK, CL1795.Contig1_YK, CL1856.Contig1_YK, CL1856.Contig2_YK, CL1910.Contig1_YK, CL1918.Contig1_YK, CL1918.Contig2_YK, CL2122.Contig1_YK, CL2122.Contig2_YK, CL2125.Contig2_YK, CL2135.Contig2_YK, CL2174.Contig1_YK, CL2174.Contig2_YK, CL2353.Contig1_YK, CL2353.Contig2_YK, CL2432.Contig1_YK, CL2432.Contig2_YK, CL2898.Contig2_YK, CL296.Contig10_YK, CL296.Contig11_YK, CL296.Contig12_YK, CL296.Contig13_YK, CL296.Contig14_YK, CL296.Contig15_YK, CL296.Contig16_YK, CL296.Contig1_YK, CL296.Contig2_YK, CL296.Contig3_YK, CL296.Contig4_YK, CL296.Contig5_YK, CL296.Contig6_YK, CL296.Contig7_YK, CL296.Contig8_YK, CL296.Contig9_YK, CL481.Contig1_YK, CL481.Contig2_YK, CL756.Contig1_YK, CL756.Contig2_YK, CL756.Contig3_YK, CL756.Contig4_YK, CL756.Contig5_YK, CL756.Contig6_YK, CL775.Contig1_YK, CL775.Contig2_YK, CL816.Contig1_YK, CL837.Contig1_YK, CL837.Contig2_YK, CL841.Contig3_YK, CL841.Contig4_YK, CL841.Contig5_YK, CL841.Contig6_YK, CL841.Contig7_YK, CL841.Contig8_YK, CL93.Contig1_YK, CL944.Contig1_YK, Unigene10000_YK, Unigene10173_YK, Unigene10289_YK, Unigene10347_YK, Unigene104_YK, Unigene10534_YK, Unigene10618_YK, Unigene10619_YK, Unigene10620_YK, Unigene10623_YK, Unigene10946_YK, Unigene11548_YK, Unigene11566_YK, Unigene11863_YK, Unigene12058_YK, Unigene12068_YK, Unigene12069_YK, Unigene12070_YK, Unigene12071_YK, Unigene12239_YK, Unigene12240_YK, Unigene12621_YK, Unigene12777_YK, Unigene12866_YK, Unigene12887_YK, Unigene12911_YK, Unigene13012_YK, Unigene13073_YK, Unigene13206_YK, Unigene13218_YK, Unigene13258_YK, Unigene13337_YK, Unigene13339_YK, Unigene13387_YK, Unigene13408_YK, Unigene13457_YK, Unigene13511_YK, Unigene13594_YK, Unigene13616_YK, Unigene13625_YK, Unigene13657_YK, Unigene13748_YK, Unigene13791_YK, Unigene13886_YK, Unigene13956_YK, Unigene13959_YK, Unigene13993_YK, Unigene14038_YK, Unigene14069_YK, Unigene14150_YK, Unigene14188_YK, Unigene14393_YK, Unigene14408_YK, Unigene14501_YK, Unigene14588_YK, Unigene14717_YK, Unigene14718_YK, Unigene14843_YK, Unigene14867_YK, Unigene14868_YK, Unigene14942_YK, Unigene14980_YK, Unigene15000_YK, Unigene15146_YK, Unigene15193_YK, Unigene15214_YK, Unigene15305_YK, Unigene15393_YK, Unigene15505_YK, Unigene15526_YK, Unigene15547_YK, Unigene15674_YK, Unigene15775_YK, Unigene15901_YK, Unigene16311_YK, Unigene1673_YK, Unigene1719_YK, Unigene17452_YK, Unigene17689_YK, Unigene2164_YK, Unigene2168_YK, Unigene2329_YK, Unigene2646_YK, Unigene2736_YK, Unigene2745_YK, Unigene2863_YK, Unigene2867_YK, Unigene3095_YK, Unigene3246_YK, Unigene3277_YK, Unigene3315_YK, Unigene3316_YK, Unigene3461_YK, Unigene3526_YK, Unigene3699_YK, Unigene3801_YK, Unigene3802_YK, Unigene4014_YK, Unigene4086_YK, Unigene4135_YK, Unigene4224_YK, Unigene4336_YK, Unigene4508_YK, Unigene4799_YK, Unigene4975_YK, Unigene4994_YK, Unigene5064_YK, Unigene5243_YK, Unigene5495_YK, Unigene5595_YK, Unigene5617_YK, Unigene5995_YK, Unigene6089_YK, Unigene6115_YK, Unigene6176_YK, Unigene6318_YK, Unigene6481_YK, Unigene6496_YK, Unigene6729_YK, Unigene6971_YK, Unigene7589_YK, Unigene7743_YK, Unigene7755_YK, Unigene7959_YK, Unigene8065_YK, Unigene8210_YK, Unigene8583_YK, Unigene8660_YK, Unigene8661_YK, Unigene8909_YK, Unigene9390_YK, Unigene9423_YK |
| 43 | [GnRH signaling pathway](../../../../D:%5C高通量测序结果%5CF14FTSSCKF1242_NEMpnqE%5Cannotation%5CKEGG%5CYK-Unigene.fa_map%5Cmap04912.html) | CL1088.Contig1_YK, CL1088.Contig2_YK, CL112.Contig1_YK, CL112.Contig2_YK, CL112.Contig3_YK, CL112.Contig4_YK, CL112.Contig5_YK, CL112.Contig6_YK, CL112.Contig7_YK, CL112.Contig8_YK, CL1292.Contig1_YK, CL1292.Contig2_YK, CL1469.Contig1_YK, CL15.Contig10_YK, CL15.Contig11_YK, CL15.Contig12_YK, CL15.Contig13_YK, CL15.Contig14_YK, CL15.Contig15_YK, CL15.Contig16_YK, CL15.Contig17_YK, CL15.Contig18_YK, CL15.Contig19_YK, CL15.Contig1_YK, CL15.Contig20_YK, CL15.Contig21_YK, CL15.Contig22_YK, CL15.Contig23_YK, CL15.Contig24_YK, CL15.Contig2_YK, CL15.Contig3_YK, CL15.Contig4_YK, CL15.Contig5_YK, CL15.Contig6_YK, CL15.Contig7_YK, CL15.Contig8_YK, CL15.Contig9_YK, CL1653.Contig1_YK, CL1653.Contig2_YK, CL1666.Contig1_YK, CL1666.Contig2_YK, CL1714.Contig2_YK, CL1758.Contig1_YK, CL1758.Contig2_YK, CL1861.Contig1_YK, CL1969.Contig1_YK, CL1969.Contig2_YK, CL2168.Contig1_YK, CL2428.Contig1_YK, CL2428.Contig2_YK, CL2429.Contig1_YK, CL2429.Contig2_YK, CL2449.Contig1_YK, CL255.Contig1_YK, CL255.Contig2_YK, CL255.Contig3_YK, CL255.Contig4_YK, CL255.Contig5_YK, CL255.Contig6_YK, CL255.Contig7_YK, CL255.Contig8_YK, CL2593.Contig1_YK, CL2593.Contig2_YK, CL2593.Contig3_YK, CL2624.Contig1_YK, CL2624.Contig2_YK, CL2793.Contig1_YK, CL2793.Contig2_YK, CL295.Contig10_YK, CL295.Contig2_YK, CL295.Contig3_YK, CL295.Contig4_YK, CL295.Contig6_YK, CL295.Contig8_YK, CL295.Contig9_YK, CL307.Contig10_YK, CL307.Contig11_YK, CL307.Contig12_YK, CL307.Contig1_YK, CL307.Contig2_YK, CL307.Contig3_YK, CL307.Contig4_YK, CL307.Contig5_YK, CL307.Contig6_YK, CL307.Contig7_YK, CL307.Contig8_YK, CL307.Contig9_YK, CL568.Contig1_YK, CL568.Contig2_YK, CL591.Contig1_YK, CL591.Contig2_YK, CL591.Contig3_YK, CL591.Contig4_YK, CL591.Contig5_YK, CL591.Contig6_YK, CL591.Contig7_YK, CL591.Contig8_YK, CL79.Contig1_YK, CL79.Contig2_YK, CL79.Contig3_YK, CL79.Contig4_YK, CL79.Contig5_YK, CL79.Contig6_YK, CL79.Contig7_YK, CL79.Contig8_YK, CL838.Contig1_YK, CL838.Contig2_YK, CL838.Contig3_YK, CL838.Contig4_YK, CL838.Contig5_YK, CL838.Contig8_YK, CL884.Contig1_YK, CL884.Contig3_YK, CL902.Contig1_YK, CL902.Contig2_YK, CL921.Contig1_YK, CL921.Contig2_YK, Unigene10197_YK, Unigene10198_YK, Unigene10746_YK, Unigene107_YK, Unigene10908_YK, Unigene11167_YK, Unigene11889_YK, Unigene12216_YK, Unigene12219_YK, Unigene1223_YK, Unigene12292_YK, Unigene12293_YK, Unigene12560_YK, Unigene12561_YK, Unigene12563_YK, Unigene12564_YK, Unigene12636_YK, Unigene12638_YK, Unigene12639_YK, Unigene13237_YK, Unigene13674_YK, Unigene13723_YK, Unigene13896_YK, Unigene13937_YK, Unigene13960_YK, Unigene1401_YK, Unigene14243_YK, Unigene14278_YK, Unigene14345_YK, Unigene14456_YK, Unigene15057_YK, Unigene15247_YK, Unigene15273_YK, Unigene15587_YK, Unigene15607_YK, Unigene158_YK, Unigene15910_YK, Unigene16000_YK, Unigene16102_YK, Unigene16406_YK, Unigene16951_YK, Unigene17056_YK, Unigene18574_YK, Unigene18819_YK, Unigene18931_YK, Unigene1964_YK, Unigene2231_YK, Unigene2289_YK, Unigene2324_YK, Unigene2550_YK, Unigene258_YK, Unigene2913_YK, Unigene331_YK, Unigene3439_YK, Unigene3626_YK, Unigene3692_YK, Unigene372_YK, Unigene4190_YK, Unigene4203_YK, Unigene4252_YK, Unigene4505_YK, Unigene4680_YK, Unigene5219_YK, Unigene5476_YK, Unigene5477_YK, Unigene5642_YK, Unigene5857_YK, Unigene5924_YK, Unigene6206_YK, Unigene6227_YK, Unigene6407_YK, Unigene6461_YK, Unigene6535_YK, Unigene6561_YK, Unigene6611_YK, Unigene6670_YK, Unigene6980_YK, Unigene6_YK, Unigene7307_YK, Unigene7308_YK, Unigene7393_YK, Unigene7402_YK, Unigene7525_YK, Unigene8101_YK, Unigene8892_YK, Unigene8893_YK, Unigene904_YK, Unigene905_YK, Unigene9071_YK, Unigene9459_YK, Unigene9710_YK |
| 44 | [Long-term potentiation](../../../../D:%5C高通量测序结果%5CF14FTSSCKF1242_NEMpnqE%5Cannotation%5CKEGG%5CYK-Unigene.fa_map%5Cmap04720.html) | CL1026.Contig1_YK, CL1026.Contig2_YK, CL1088.Contig1_YK, CL1088.Contig2_YK, CL112.Contig1_YK, CL112.Contig2_YK, CL112.Contig3_YK, CL112.Contig4_YK, CL112.Contig5_YK, CL112.Contig6_YK, CL112.Contig7_YK, CL112.Contig8_YK, CL1131.Contig2_YK, CL1131.Contig3_YK, CL1138.Contig2_YK, CL1412.Contig1_YK, CL15.Contig10_YK, CL15.Contig11_YK, CL15.Contig12_YK, CL15.Contig13_YK, CL15.Contig14_YK, CL15.Contig15_YK, CL15.Contig16_YK, CL15.Contig17_YK, CL15.Contig18_YK, CL15.Contig19_YK, CL15.Contig1_YK, CL15.Contig20_YK, CL15.Contig21_YK, CL15.Contig22_YK, CL15.Contig23_YK, CL15.Contig24_YK, CL15.Contig2_YK, CL15.Contig3_YK, CL15.Contig4_YK, CL15.Contig5_YK, CL15.Contig6_YK, CL15.Contig7_YK, CL15.Contig8_YK, CL15.Contig9_YK, CL1578.Contig1_YK, CL1602.Contig1_YK, CL1602.Contig2_YK, CL1653.Contig1_YK, CL1653.Contig2_YK, CL1714.Contig2_YK, CL1861.Contig1_YK, CL2135.Contig1_YK, CL2135.Contig2_YK, CL2240.Contig1_YK, CL2240.Contig2_YK, CL2336.Contig1_YK, CL2336.Contig2_YK, CL2381.Contig1_YK, CL2381.Contig2_YK, CL255.Contig1_YK, CL255.Contig2_YK, CL255.Contig3_YK, CL255.Contig4_YK, CL255.Contig5_YK, CL255.Contig6_YK, CL255.Contig7_YK, CL255.Contig8_YK, CL2593.Contig1_YK, CL2593.Contig2_YK, CL2593.Contig3_YK, CL2635.Contig1_YK, CL2635.Contig2_YK, CL2764.Contig1_YK, CL2764.Contig2_YK, CL2787.Contig2_YK, CL295.Contig10_YK, CL295.Contig2_YK, CL295.Contig3_YK, CL295.Contig4_YK, CL295.Contig6_YK, CL295.Contig8_YK, CL295.Contig9_YK, CL307.Contig10_YK, CL307.Contig11_YK, CL307.Contig12_YK, CL307.Contig1_YK, CL307.Contig2_YK, CL307.Contig3_YK, CL307.Contig4_YK, CL307.Contig5_YK, CL307.Contig6_YK, CL307.Contig7_YK, CL307.Contig8_YK, CL307.Contig9_YK, CL312.Contig1_YK, CL387.Contig1_YK, CL387.Contig2_YK, CL387.Contig3_YK, CL387.Contig4_YK, CL387.Contig5_YK, CL431.Contig1_YK, CL431.Contig2_YK, CL431.Contig3_YK, CL568.Contig1_YK, CL568.Contig2_YK, CL591.Contig1_YK, CL591.Contig2_YK, CL591.Contig3_YK, CL591.Contig4_YK, CL591.Contig5_YK, CL591.Contig6_YK, CL591.Contig7_YK, CL591.Contig8_YK, CL83.Contig4_YK, CL884.Contig1_YK, CL884.Contig3_YK, CL902.Contig1_YK, CL902.Contig2_YK, CL935.Contig1_YK, CL935.Contig2_YK, Unigene10096_YK, Unigene10493_YK, Unigene10546_YK, Unigene10547_YK, Unigene10614_YK, Unigene10659_YK, Unigene10829_YK, Unigene10908_YK, Unigene11140_YK, Unigene11141_YK, Unigene11481_YK, Unigene11662_YK, Unigene12128_YK, Unigene12216_YK, Unigene12219_YK, Unigene12451_YK, Unigene12560_YK, Unigene12561_YK, Unigene12563_YK, Unigene12564_YK, Unigene12636_YK, Unigene12638_YK, Unigene12639_YK, Unigene12733_YK, Unigene13180_YK, Unigene1356_YK, Unigene13571_YK, Unigene13674_YK, Unigene13827_YK, Unigene13877_YK, Unigene13937_YK, Unigene13960_YK, Unigene14243_YK, Unigene14345_YK, Unigene14516_YK, Unigene14581_YK, Unigene14922_YK, Unigene15057_YK, Unigene15348_YK, Unigene15587_YK, Unigene158_YK, Unigene15910_YK, Unigene16112_YK, Unigene17811_YK, Unigene18574_YK, Unigene18819_YK, Unigene1964_YK, Unigene201_YK, Unigene2080_YK, Unigene2289_YK, Unigene2550_YK, Unigene319_YK, Unigene331_YK, Unigene337_YK, Unigene3439_YK, Unigene3585_YK, Unigene3626_YK, Unigene4052_YK, Unigene4062_YK, Unigene4252_YK, Unigene4445_YK, Unigene4680_YK, Unigene4689_YK, Unigene4690_YK, Unigene4778_YK, Unigene5219_YK, Unigene5385_YK, Unigene5386_YK, Unigene5467_YK, Unigene5468_YK, Unigene5476_YK, Unigene5477_YK, Unigene5857_YK, Unigene6006_YK, Unigene6007_YK, Unigene6206_YK, Unigene6227_YK, Unigene6367_YK, Unigene6368_YK, Unigene6461_YK, Unigene6561_YK, Unigene6611_YK, Unigene6_YK, Unigene7307_YK, Unigene7308_YK, Unigene7382_YK, Unigene7390_YK, Unigene7391_YK, Unigene7525_YK, Unigene838_YK, Unigene9127_YK, Unigene9459_YK |
| 45 | [Alcoholism](../../../../D:%5C高通量测序结果%5CF14FTSSCKF1242_NEMpnqE%5Cannotation%5CKEGG%5CYK-Unigene.fa_map%5Cmap05034.html) | CL1026.Contig1_YK, CL1026.Contig2_YK, CL1088.Contig1_YK, CL1088.Contig2_YK, CL1098.Contig1_YK, CL1098.Contig2_YK, CL1412.Contig1_YK, CL1544.Contig1_YK, CL1544.Contig2_YK, CL1602.Contig1_YK, CL1602.Contig2_YK, CL1653.Contig1_YK, CL1653.Contig2_YK, CL1704.Contig1_YK, CL1714.Contig2_YK, CL1809.Contig1_YK, CL1809.Contig2_YK, CL1809.Contig3_YK, CL1809.Contig4_YK, CL1861.Contig1_YK, CL1933.Contig1_YK, CL1933.Contig2_YK, CL1968.Contig1_YK, CL1968.Contig2_YK, CL1977.Contig1_YK, CL1977.Contig2_YK, CL1977.Contig3_YK, CL1977.Contig4_YK, CL2192.Contig1_YK, CL2192.Contig2_YK, CL2254.Contig1_YK, CL2254.Contig2_YK, CL2254.Contig3_YK, CL2265.Contig1_YK, CL2265.Contig2_YK, CL255.Contig1_YK, CL255.Contig2_YK, CL255.Contig3_YK, CL255.Contig4_YK, CL255.Contig5_YK, CL255.Contig6_YK, CL255.Contig7_YK, CL255.Contig8_YK, CL2593.Contig1_YK, CL2593.Contig2_YK, CL2593.Contig3_YK, CL2846.Contig1_YK, CL312.Contig1_YK, CL365.Contig1_YK, CL365.Contig2_YK, CL365.Contig3_YK, CL568.Contig1_YK, CL568.Contig2_YK, CL821.Contig1_YK, CL821.Contig2_YK, CL821.Contig3_YK, CL821.Contig4_YK, CL821.Contig5_YK, CL902.Contig1_YK, CL902.Contig2_YK, CL935.Contig1_YK, CL935.Contig2_YK, CL97.Contig1_YK, CL97.Contig2_YK, Unigene10197_YK, Unigene10198_YK, Unigene10209_YK, Unigene10210_YK, Unigene10211_YK, Unigene10614_YK, Unigene10627_YK, Unigene107_YK, Unigene10908_YK, Unigene10940_YK, Unigene10993_YK, Unigene11140_YK, Unigene11141_YK, Unigene11448_YK, Unigene11567_YK, Unigene11568_YK, Unigene11662_YK, Unigene11706_YK, Unigene12021_YK, Unigene12128_YK, Unigene12216_YK, Unigene12219_YK, Unigene1223_YK, Unigene12451_YK, Unigene12733_YK, Unigene12813_YK, Unigene13174_YK, Unigene13180_YK, Unigene13237_YK, Unigene1356_YK, Unigene13670_YK, Unigene13674_YK, Unigene13937_YK, Unigene13960_YK, Unigene13969_YK, Unigene14045_YK, Unigene14080_YK, Unigene14094_YK, Unigene14159_YK, Unigene14243_YK, Unigene14306_YK, Unigene14345_YK, Unigene14507_YK, Unigene14628_YK, Unigene14647_YK, Unigene14696_YK, Unigene14922_YK, Unigene15057_YK, Unigene15348_YK, Unigene15394_YK, Unigene15587_YK, Unigene15877_YK, Unigene15910_YK, Unigene16112_YK, Unigene16638_YK, Unigene17811_YK, Unigene18164_YK, Unigene18574_YK, Unigene18798_YK, Unigene18819_YK, Unigene1964_YK, Unigene201_YK, Unigene2080_YK, Unigene2148_YK, Unigene2459_YK, Unigene2550_YK, Unigene258_YK, Unigene2657_YK, Unigene3103_YK, Unigene3193_YK, Unigene319_YK, Unigene3200_YK, Unigene3219_YK, Unigene331_YK, Unigene337_YK, Unigene3439_YK, Unigene3596_YK, Unigene3619_YK, Unigene3626_YK, Unigene3628_YK, Unigene372_YK, Unigene3885_YK, Unigene3937_YK, Unigene3938_YK, Unigene4239_YK, Unigene4313_YK, Unigene4322_YK, Unigene4416_YK, Unigene4419_YK, Unigene4445_YK, Unigene4564_YK, Unigene4565_YK, Unigene4680_YK, Unigene4689_YK, Unigene4690_YK, Unigene4772_YK, Unigene4778_YK, Unigene4827_YK, Unigene5219_YK, Unigene5385_YK, Unigene5386_YK, Unigene5438_YK, Unigene5467_YK, Unigene5468_YK, Unigene5608_YK, Unigene5642_YK, Unigene6006_YK, Unigene6007_YK, Unigene6227_YK, Unigene6367_YK, Unigene6368_YK, Unigene6406_YK, Unigene6461_YK, Unigene6561_YK, Unigene6611_YK, Unigene6645_YK, Unigene6980_YK, Unigene6_YK, Unigene7307_YK, Unigene7308_YK, Unigene7390_YK, Unigene7556_YK, Unigene765_YK, Unigene7920_YK, Unigene8101_YK, Unigene838_YK, Unigene8606_YK, Unigene8677_YK, Unigene8678_YK, Unigene8679_YK, Unigene8682_YK, Unigene8683_YK, Unigene9036_YK, Unigene904_YK, Unigene905_YK, Unigene9168_YK, Unigene9169_YK, Unigene9459_YK, Unigene9566_YK, Unigene9706_YK, Unigene9984_YK |
| 46 | [Cholinergic synapse](../../../../D:%5C高通量测序结果%5CF14FTSSCKF1242_NEMpnqE%5Cannotation%5CKEGG%5CYK-Unigene.fa_map%5Cmap04725.html) | CL1088.Contig1_YK, CL1088.Contig2_YK, CL112.Contig1_YK, CL112.Contig2_YK, CL112.Contig3_YK, CL112.Contig4_YK, CL112.Contig5_YK, CL112.Contig6_YK, CL112.Contig7_YK, CL112.Contig8_YK, CL15.Contig10_YK, CL15.Contig11_YK, CL15.Contig12_YK, CL15.Contig13_YK, CL15.Contig14_YK, CL15.Contig15_YK, CL15.Contig16_YK, CL15.Contig17_YK, CL15.Contig18_YK, CL15.Contig19_YK, CL15.Contig1_YK, CL15.Contig20_YK, CL15.Contig21_YK, CL15.Contig22_YK, CL15.Contig23_YK, CL15.Contig24_YK, CL15.Contig2_YK, CL15.Contig3_YK, CL15.Contig4_YK, CL15.Contig5_YK, CL15.Contig6_YK, CL15.Contig7_YK, CL15.Contig8_YK, CL15.Contig9_YK, CL1653.Contig1_YK, CL1653.Contig2_YK, CL1689.Contig2_YK, CL1858.Contig1_YK, CL1858.Contig2_YK, CL1921.Contig1_YK, CL1921.Contig2_YK, CL1990.Contig1_YK, CL1990.Contig2_YK, CL2032.Contig1_YK, CL2032.Contig2_YK, CL2265.Contig1_YK, CL2265.Contig2_YK, CL2337.Contig1_YK, CL2337.Contig2_YK, CL2509.Contig1_YK, CL2509.Contig2_YK, CL255.Contig1_YK, CL255.Contig2_YK, CL255.Contig3_YK, CL255.Contig4_YK, CL255.Contig5_YK, CL255.Contig6_YK, CL255.Contig7_YK, CL255.Contig8_YK, CL278.Contig3_YK, CL278.Contig4_YK, CL278.Contig5_YK, CL278.Contig6_YK, CL2792.Contig1_YK, CL295.Contig10_YK, CL295.Contig2_YK, CL295.Contig3_YK, CL295.Contig4_YK, CL295.Contig6_YK, CL295.Contig8_YK, CL295.Contig9_YK, CL307.Contig10_YK, CL307.Contig11_YK, CL307.Contig12_YK, CL307.Contig1_YK, CL307.Contig2_YK, CL307.Contig3_YK, CL307.Contig4_YK, CL307.Contig5_YK, CL307.Contig6_YK, CL307.Contig7_YK, CL307.Contig8_YK, CL307.Contig9_YK, CL372.Contig1_YK, CL372.Contig2_YK, CL372.Contig3_YK, CL372.Contig4_YK, CL372.Contig5_YK, CL372.Contig6_YK, CL372.Contig7_YK, CL372.Contig8_YK, CL591.Contig1_YK, CL591.Contig2_YK, CL591.Contig3_YK, CL591.Contig4_YK, CL591.Contig5_YK, CL591.Contig6_YK, CL591.Contig7_YK, CL591.Contig8_YK, CL79.Contig1_YK, CL79.Contig2_YK, CL79.Contig3_YK, CL79.Contig4_YK, CL79.Contig5_YK, CL79.Contig6_YK, CL79.Contig7_YK, CL79.Contig8_YK, CL838.Contig1_YK, CL838.Contig2_YK, CL838.Contig3_YK, CL838.Contig4_YK, CL838.Contig5_YK, CL838.Contig8_YK, CL884.Contig1_YK, CL884.Contig3_YK, Unigene10474_YK, Unigene10563_YK, Unigene10627_YK, Unigene10908_YK, Unigene10972_YK, Unigene11335_YK, Unigene11889_YK, Unigene11890_YK, Unigene12199_YK, Unigene12216_YK, Unigene12218_YK, Unigene12219_YK, Unigene12292_YK, Unigene12293_YK, Unigene12560_YK, Unigene12561_YK, Unigene12563_YK, Unigene12564_YK, Unigene12636_YK, Unigene12638_YK, Unigene12639_YK, Unigene13674_YK, Unigene13969_YK, Unigene14080_YK, Unigene14094_YK, Unigene14159_YK, Unigene14345_YK, Unigene14507_YK, Unigene1469_YK, Unigene14735_YK, Unigene14926_YK, Unigene15497_YK, Unigene15877_YK, Unigene158_YK, Unigene15910_YK, Unigene16000_YK, Unigene16406_YK, Unigene17478_YK, Unigene17790_YK, Unigene18164_YK, Unigene18574_YK, Unigene18798_YK, Unigene18819_YK, Unigene1964_YK, Unigene2098_YK, Unigene2148_YK, Unigene2231_YK, Unigene2289_YK, Unigene258_YK, Unigene2791_YK, Unigene3200_YK, Unigene3219_YK, Unigene331_YK, Unigene3439_YK, Unigene352_YK, Unigene3596_YK, Unigene3753_YK, Unigene3885_YK, Unigene3937_YK, Unigene4252_YK, Unigene4564_YK, Unigene4565_YK, Unigene4680_YK, Unigene4772_YK, Unigene5219_YK, Unigene5330_YK, Unigene5438_YK, Unigene5476_YK, Unigene5477_YK, Unigene5857_YK, Unigene5944_YK, Unigene6206_YK, Unigene6229_YK, Unigene6230_YK, Unigene6231_YK, Unigene6561_YK, Unigene6900_YK, Unigene6952_YK, Unigene7120_YK, Unigene7525_YK, Unigene765_YK, Unigene8323_YK, Unigene8324_YK, Unigene8325_YK, Unigene8951_YK, Unigene8953_YK, Unigene9710_YK, Unigene9763_YK, Unigene9764_YK |
| 47 | [Oocyte meiosis](../../../../D:%5C高通量测序结果%5CF14FTSSCKF1242_NEMpnqE%5Cannotation%5CKEGG%5CYK-Unigene.fa_map%5Cmap04114.html) | CL1004.Contig1_YK, CL1004.Contig2_YK, CL1026.Contig1_YK, CL1026.Contig2_YK, CL1088.Contig1_YK, CL1088.Contig2_YK, CL11.Contig3_YK, CL1119.Contig1_YK, CL1119.Contig2_YK, CL112.Contig1_YK, CL112.Contig2_YK, CL112.Contig3_YK, CL112.Contig4_YK, CL112.Contig5_YK, CL112.Contig6_YK, CL112.Contig7_YK, CL112.Contig8_YK, CL1308.Contig1_YK, CL1308.Contig2_YK, CL1324.Contig3_YK, CL1412.Contig1_YK, CL1412.Contig2_YK, CL1412.Contig3_YK, CL1464.Contig1_YK, CL1531.Contig1_YK, CL1531.Contig2_YK, CL1714.Contig2_YK, CL1861.Contig1_YK, CL1918.Contig1_YK, CL1918.Contig2_YK, CL1949.Contig1_YK, CL1949.Contig2_YK, CL2135.Contig1_YK, CL2135.Contig2_YK, CL2168.Contig1_YK, CL2199.Contig1_YK, CL2199.Contig2_YK, CL2199.Contig3_YK, CL2353.Contig1_YK, CL2353.Contig2_YK, CL2532.Contig1_YK, CL2532.Contig2_YK, CL255.Contig1_YK, CL255.Contig2_YK, CL255.Contig3_YK, CL255.Contig4_YK, CL255.Contig5_YK, CL255.Contig6_YK, CL255.Contig7_YK, CL255.Contig8_YK, CL2593.Contig1_YK, CL2593.Contig2_YK, CL2593.Contig3_YK, CL2624.Contig1_YK, CL2624.Contig2_YK, CL2635.Contig1_YK, CL2635.Contig2_YK, CL2764.Contig1_YK, CL2764.Contig2_YK, CL295.Contig10_YK, CL295.Contig2_YK, CL295.Contig3_YK, CL295.Contig4_YK, CL295.Contig6_YK, CL295.Contig8_YK, CL295.Contig9_YK, CL312.Contig1_YK, CL424.Contig1_YK, CL424.Contig2_YK, CL424.Contig3_YK, CL431.Contig1_YK, CL431.Contig2_YK, CL431.Contig3_YK, CL568.Contig1_YK, CL568.Contig2_YK, CL79.Contig1_YK, CL79.Contig2_YK, CL79.Contig3_YK, CL79.Contig4_YK, CL79.Contig5_YK, CL79.Contig6_YK, CL79.Contig7_YK, CL79.Contig8_YK, CL902.Contig1_YK, CL902.Contig2_YK, CL935.Contig1_YK, CL935.Contig2_YK, Unigene10096_YK, Unigene104_YK, Unigene10546_YK, Unigene10547_YK, Unigene10785_YK, Unigene10818_YK, Unigene10908_YK, Unigene11140_YK, Unigene11141_YK, Unigene11566_YK, Unigene11662_YK, Unigene12058_YK, Unigene12068_YK, Unigene12069_YK, Unigene12070_YK, Unigene12071_YK, Unigene12128_YK, Unigene12216_YK, Unigene12219_YK, Unigene12292_YK, Unigene12293_YK, Unigene12512_YK, Unigene12560_YK, Unigene12561_YK, Unigene12563_YK, Unigene12564_YK, Unigene12733_YK, Unigene12777_YK, Unigene12792_YK, Unigene12793_YK, Unigene13069_YK, Unigene13180_YK, Unigene13457_YK, Unigene13594_YK, Unigene13674_YK, Unigene13791_YK, Unigene13877_YK, Unigene13915_YK, Unigene13937_YK, Unigene13960_YK, Unigene14243_YK, Unigene14276_YK, Unigene14318_YK, Unigene14345_YK, Unigene14408_YK, Unigene14425_YK, Unigene14601_YK, Unigene14612_YK, Unigene14969_YK, Unigene14980_YK, Unigene15000_YK, Unigene15057_YK, Unigene15348_YK, Unigene15505_YK, Unigene15587_YK, Unigene15836_YK, Unigene16112_YK, Unigene16406_YK, Unigene18716_YK, Unigene19134_YK, Unigene201_YK, Unigene2080_YK, Unigene2231_YK, Unigene2289_YK, Unigene2550_YK, Unigene258_YK, Unigene2600_YK, Unigene2736_YK, Unigene2814_YK, Unigene2863_YK, Unigene2905_YK, Unigene3165_YK, Unigene3174_YK, Unigene3214_YK, Unigene3315_YK, Unigene3316_YK, Unigene331_YK, Unigene337_YK, Unigene3428_YK, Unigene3491_YK, Unigene3626_YK, Unigene4062_YK, Unigene4086_YK, Unigene4114_YK, Unigene4166_YK, Unigene4179_YK, Unigene4428_YK, Unigene4436_YK, Unigene4488_YK, Unigene4684_YK, Unigene4689_YK, Unigene4690_YK, Unigene4767_YK, Unigene5219_YK, Unigene5385_YK, Unigene5386_YK, Unigene5467_YK, Unigene5468_YK, Unigene5495_YK, Unigene6227_YK, Unigene6318_YK, Unigene6367_YK, Unigene6368_YK, Unigene6461_YK, Unigene6611_YK, Unigene6729_YK, Unigene6859_YK, Unigene6_YK, Unigene7153_YK, Unigene7307_YK, Unigene7308_YK, Unigene7382_YK, Unigene838_YK, Unigene8909_YK, Unigene9459_YK, Unigene9706_YK, Unigene9710_YK |
| 48 | [Adherens junction](../../../../D:%5C高通量测序结果%5CF14FTSSCKF1242_NEMpnqE%5Cannotation%5CKEGG%5CYK-Unigene.fa_map%5Cmap04520.html) | CL1003.Contig1_YK, CL1003.Contig2_YK, CL1043.Contig1_YK, CL1119.Contig1_YK, CL1119.Contig2_YK, CL1136.Contig1_YK, CL1221.Contig1_YK, CL1221.Contig2_YK, CL1221.Contig3_YK, CL1223.Contig1_YK, CL1223.Contig3_YK, CL1292.Contig1_YK, CL1292.Contig2_YK, CL1332.Contig1_YK, CL1332.Contig2_YK, CL1332.Contig3_YK, CL1332.Contig4_YK, CL1381.Contig1_YK, CL1381.Contig2_YK, CL1390.Contig1_YK, CL1390.Contig2_YK, CL1488.Contig1_YK, CL1488.Contig2_YK, CL1624.Contig1_YK, CL1624.Contig3_YK, CL1666.Contig1_YK, CL1666.Contig2_YK, CL170.Contig2_YK, CL1790.Contig1_YK, CL1790.Contig2_YK, CL1790.Contig3_YK, CL1790.Contig4_YK, CL1814.Contig1_YK, CL1814.Contig2_YK, CL1816.Contig1_YK, CL1816.Contig2_YK, CL1831.Contig2_YK, CL186.Contig1_YK, CL186.Contig2_YK, CL186.Contig3_YK, CL186.Contig4_YK, CL186.Contig5_YK, CL186.Contig6_YK, CL186.Contig7_YK, CL186.Contig8_YK, CL1930.Contig1_YK, CL1930.Contig2_YK, CL1930.Contig3_YK, CL1930.Contig4_YK, CL2006.Contig1_YK, CL2006.Contig2_YK, CL2006.Contig3_YK, CL2154.Contig1_YK, CL2154.Contig2_YK, CL2180.Contig1_YK, CL2180.Contig2_YK, CL2214.Contig1_YK, CL2214.Contig2_YK, CL2336.Contig1_YK, CL2336.Contig2_YK, CL2429.Contig1_YK, CL2429.Contig2_YK, CL2449.Contig1_YK, CL2732.Contig1_YK, CL2732.Contig2_YK, CL2856.Contig1_YK, CL2856.Contig2_YK, CL2905.Contig1_YK, CL2905.Contig2_YK, CL300.Contig1_YK, CL300.Contig2_YK, CL387.Contig1_YK, CL387.Contig2_YK, CL387.Contig3_YK, CL387.Contig4_YK, CL387.Contig5_YK, CL415.Contig1_YK, CL415.Contig2_YK, CL415.Contig3_YK, CL415.Contig4_YK, CL438.Contig2_YK, CL438.Contig3_YK, CL512.Contig2_YK, CL512.Contig3_YK, CL522.Contig1_YK, CL522.Contig2_YK, CL627.Contig1_YK, CL627.Contig2_YK, CL650.Contig1_YK, CL650.Contig2_YK, CL667.Contig1_YK, CL667.Contig2_YK, CL667.Contig3_YK, CL67.Contig1_YK, CL67.Contig2_YK, CL674.Contig10_YK, CL674.Contig3_YK, CL695.Contig2_YK, CL695.Contig3_YK, CL695.Contig4_YK, CL720.Contig1_YK, CL720.Contig2_YK, CL741.Contig2_YK, CL741.Contig3_YK, CL78.Contig1_YK, CL78.Contig2_YK, CL78.Contig3_YK, CL83.Contig4_YK, CL832.Contig1_YK, CL84.Contig2_YK, CL84.Contig3_YK, Unigene10308_YK, Unigene10439_YK, Unigene10440_YK, Unigene10441_YK, Unigene10659_YK, Unigene10954_YK, Unigene11116_YK, Unigene11117_YK, Unigene11299_YK, Unigene11350_YK, Unigene12044_YK, Unigene12045_YK, Unigene12090_YK, Unigene12091_YK, Unigene12238_YK, Unigene12417_YK, Unigene12418_YK, Unigene12419_YK, Unigene12420_YK, Unigene12532_YK, Unigene12616_YK, Unigene12898_YK, Unigene12899_YK, Unigene13313_YK, Unigene13383_YK, Unigene13674_YK, Unigene13720_YK, Unigene13723_YK, Unigene13743_YK, Unigene13811_YK, Unigene13985_YK, Unigene14081_YK, Unigene14254_YK, Unigene14516_YK, Unigene14577_YK, Unigene14735_YK, Unigene14929_YK, Unigene15190_YK, Unigene15325_YK, Unigene15458_YK, Unigene15647_YK, Unigene15774_YK, Unigene15780_YK, Unigene15795_YK, Unigene16236_YK, Unigene16250_YK, Unigene16898_YK, Unigene17974_YK, Unigene179_YK, Unigene18316_YK, Unigene2140_YK, Unigene2273_YK, Unigene2390_YK, Unigene256_YK, Unigene2849_YK, Unigene3032_YK, Unigene3067_YK, Unigene3293_YK, Unigene3318_YK, Unigene3321_YK, Unigene3328_YK, Unigene3430_YK, Unigene3495_YK, Unigene3906_YK, Unigene4203_YK, Unigene4494_YK, Unigene4583_YK, Unigene4761_YK, Unigene6027_YK, Unigene6182_YK, Unigene6309_YK, Unigene6310_YK, Unigene6407_YK, Unigene6553_YK, Unigene6600_YK, Unigene6879_YK, Unigene6880_YK, Unigene712_YK, Unigene7447_YK, Unigene8001_YK, Unigene800_YK, Unigene801_YK, Unigene8500_YK, Unigene8501_YK, Unigene8552_YK, Unigene8839_YK, Unigene9036_YK, Unigene9127_YK, Unigene9323_YK, Unigene9388_YK, Unigene9732_YK, Unigene9840_YK |
| 49 | [Cell cycle](../../../../D:%5C高通量测序结果%5CF14FTSSCKF1242_NEMpnqE%5Cannotation%5CKEGG%5CYK-Unigene.fa_map%5Cmap04110.html) | CL1004.Contig1_YK, CL1004.Contig2_YK, CL11.Contig3_YK, CL110.Contig1_YK, CL110.Contig2_YK, CL1117.Contig1_YK, CL130.Contig1_YK, CL1308.Contig1_YK, CL1308.Contig2_YK, CL1390.Contig1_YK, CL1390.Contig2_YK, CL1412.Contig1_YK, CL1412.Contig2_YK, CL1412.Contig3_YK, CL1464.Contig1_YK, CL1531.Contig1_YK, CL1531.Contig2_YK, CL1544.Contig1_YK, CL1544.Contig2_YK, CL155.Contig1_YK, CL155.Contig2_YK, CL1659.Contig1_YK, CL1659.Contig2_YK, CL174.Contig1_YK, CL1856.Contig1_YK, CL1856.Contig2_YK, CL1910.Contig1_YK, CL1918.Contig1_YK, CL1918.Contig2_YK, CL1983.Contig1_YK, CL1983.Contig2_YK, CL1983.Contig3_YK, CL2018.Contig1_YK, CL2018.Contig2_YK, CL2069.Contig1_YK, CL2135.Contig2_YK, CL2199.Contig1_YK, CL2199.Contig2_YK, CL2199.Contig3_YK, CL2336.Contig1_YK, CL2336.Contig2_YK, CL2353.Contig1_YK, CL2353.Contig2_YK, CL2532.Contig1_YK, CL2532.Contig2_YK, CL2668.Contig1_YK, CL2668.Contig2_YK, CL2672.Contig2_YK, CL2898.Contig2_YK, CL378.Contig1_YK, CL378.Contig2_YK, CL378.Contig3_YK, CL378.Contig4_YK, CL378.Contig5_YK, CL387.Contig1_YK, CL387.Contig2_YK, CL387.Contig3_YK, CL387.Contig4_YK, CL387.Contig5_YK, CL424.Contig1_YK, CL424.Contig2_YK, CL424.Contig3_YK, CL774.Contig1_YK, CL774.Contig2_YK, CL816.Contig1_YK, CL83.Contig4_YK, CL833.Contig1_YK, CL841.Contig3_YK, CL841.Contig4_YK, CL841.Contig5_YK, CL841.Contig6_YK, CL841.Contig7_YK, CL841.Contig8_YK, CL93.Contig1_YK, Unigene10000_YK, Unigene10173_YK, Unigene10299_YK, Unigene104_YK, Unigene10534_YK, Unigene10623_YK, Unigene10659_YK, Unigene10785_YK, Unigene10818_YK, Unigene10819_YK, Unigene10857_YK, Unigene11204_YK, Unigene11259_YK, Unigene11284_YK, Unigene11548_YK, Unigene11566_YK, Unigene11863_YK, Unigene11931_YK, Unigene12001_YK, Unigene12058_YK, Unigene12068_YK, Unigene12069_YK, Unigene12070_YK, Unigene12071_YK, Unigene12777_YK, Unigene12792_YK, Unigene12793_YK, Unigene12866_YK, Unigene12887_YK, Unigene12911_YK, Unigene13457_YK, Unigene13491_YK, Unigene13597_YK, Unigene13618_YK, Unigene13657_YK, Unigene13791_YK, Unigene13903_YK, Unigene13978_YK, Unigene14027_YK, Unigene14177_YK, Unigene14255_YK, Unigene14318_YK, Unigene14335_YK, Unigene14382_YK, Unigene14408_YK, Unigene14425_YK, Unigene14516_YK, Unigene14596_YK, Unigene14601_YK, Unigene14626_YK, Unigene14805_YK, Unigene14844_YK, Unigene14867_YK, Unigene14969_YK, Unigene14980_YK, Unigene15000_YK, Unigene15034_YK, Unigene1543_YK, Unigene15485_YK, Unigene15505_YK, Unigene15517_YK, Unigene15558_YK, Unigene15561_YK, Unigene15780_YK, Unigene1673_YK, Unigene1719_YK, Unigene17452_YK, Unigene17689_YK, Unigene18490_YK, Unigene18716_YK, Unigene19134_YK, Unigene2646_YK, Unigene2736_YK, Unigene2815_YK, Unigene2816_YK, Unigene2863_YK, Unigene2905_YK, Unigene3032_YK, Unigene3042_YK, Unigene3165_YK, Unigene3174_YK, Unigene3315_YK, Unigene3316_YK, Unigene3321_YK, Unigene3384_YK, Unigene3431_YK, Unigene3491_YK, Unigene3526_YK, Unigene3841_YK, Unigene4086_YK, Unigene4114_YK, Unigene4166_YK, Unigene4428_YK, Unigene4436_YK, Unigene4684_YK, Unigene4767_YK, Unigene4799_YK, Unigene4_YK, Unigene5356_YK, Unigene5451_YK, Unigene5495_YK, Unigene5519_YK, Unigene5617_YK, Unigene6027_YK, Unigene6089_YK, Unigene6306_YK, Unigene6318_YK, Unigene6406_YK, Unigene6664_YK, Unigene6715_YK, Unigene6729_YK, Unigene6859_YK, Unigene6899_YK, Unigene6971_YK, Unigene7448_YK, Unigene7816_YK, Unigene7959_YK, Unigene8065_YK, Unigene8329_YK, Unigene8583_YK, Unigene8909_YK, Unigene9127_YK, Unigene9390_YK, Unigene9706_YK, Unigene9968_YK, Unigene9969_YK |
| 50 | [Retrograde endocannabinoid signaling](../../../../D:%5C高通量测序结果%5CF14FTSSCKF1242_NEMpnqE%5Cannotation%5CKEGG%5CYK-Unigene.fa_map%5Cmap04723.html) | CL1088.Contig1_YK, CL1088.Contig2_YK, CL1098.Contig1_YK, CL1098.Contig2_YK, CL1289.Contig1_YK, CL1402.Contig1_YK, CL1402.Contig2_YK, CL149.Contig1_YK, CL149.Contig2_YK, CL15.Contig10_YK, CL15.Contig11_YK, CL15.Contig12_YK, CL15.Contig13_YK, CL15.Contig14_YK, CL15.Contig15_YK, CL15.Contig16_YK, CL15.Contig17_YK, CL15.Contig18_YK, CL15.Contig19_YK, CL15.Contig1_YK, CL15.Contig20_YK, CL15.Contig21_YK, CL15.Contig22_YK, CL15.Contig23_YK, CL15.Contig24_YK, CL15.Contig2_YK, CL15.Contig3_YK, CL15.Contig4_YK, CL15.Contig5_YK, CL15.Contig6_YK, CL15.Contig7_YK, CL15.Contig8_YK, CL15.Contig9_YK, CL1689.Contig2_YK, CL1767.Contig2_YK, CL1838.Contig1_YK, CL1838.Contig2_YK, CL1919.Contig1_YK, CL1919.Contig2_YK, CL2168.Contig1_YK, CL2181.Contig1_YK, CL2181.Contig2_YK, CL2240.Contig1_YK, CL2240.Contig2_YK, CL2260.Contig1_YK, CL2260.Contig2_YK, CL2265.Contig1_YK, CL2265.Contig2_YK, CL2407.Contig1_YK, CL255.Contig1_YK, CL255.Contig2_YK, CL255.Contig3_YK, CL255.Contig4_YK, CL255.Contig5_YK, CL255.Contig6_YK, CL255.Contig7_YK, CL255.Contig8_YK, CL2622.Contig1_YK, CL2622.Contig2_YK, CL2624.Contig1_YK, CL2624.Contig2_YK, CL278.Contig3_YK, CL278.Contig4_YK, CL2790.Contig1_YK, CL2790.Contig2_YK, CL295.Contig10_YK, CL295.Contig2_YK, CL295.Contig3_YK, CL295.Contig4_YK, CL295.Contig6_YK, CL295.Contig8_YK, CL295.Contig9_YK, CL307.Contig10_YK, CL307.Contig11_YK, CL307.Contig12_YK, CL307.Contig1_YK, CL307.Contig2_YK, CL307.Contig3_YK, CL307.Contig4_YK, CL307.Contig5_YK, CL307.Contig6_YK, CL307.Contig7_YK, CL307.Contig8_YK, CL307.Contig9_YK, CL591.Contig1_YK, CL591.Contig2_YK, CL591.Contig3_YK, CL591.Contig4_YK, CL591.Contig5_YK, CL591.Contig6_YK, CL591.Contig7_YK, CL591.Contig8_YK, CL670.Contig1_YK, CL79.Contig1_YK, CL79.Contig2_YK, CL79.Contig3_YK, CL79.Contig4_YK, CL79.Contig5_YK, CL79.Contig6_YK, CL79.Contig7_YK, CL79.Contig8_YK, CL838.Contig1_YK, CL838.Contig2_YK, CL838.Contig3_YK, CL838.Contig4_YK, CL838.Contig5_YK, CL838.Contig8_YK, CL884.Contig1_YK, CL884.Contig3_YK, CL972.Contig1_YK, CL972.Contig2_YK, CL972.Contig3_YK, CL972.Contig4_YK, Unigene10310_YK, Unigene10493_YK, Unigene10520_YK, Unigene10627_YK, Unigene10781_YK, Unigene10790_YK, Unigene10829_YK, Unigene10908_YK, Unigene11463_YK, Unigene11836_YK, Unigene11889_YK, Unigene11890_YK, Unigene12216_YK, Unigene12219_YK, Unigene12292_YK, Unigene12293_YK, Unigene12348_YK, Unigene12560_YK, Unigene12561_YK, Unigene12563_YK, Unigene12564_YK, Unigene12636_YK, Unigene12638_YK, Unigene12639_YK, Unigene13674_YK, Unigene13969_YK, Unigene14080_YK, Unigene14159_YK, Unigene14507_YK, Unigene1450_YK, Unigene15095_YK, Unigene15706_YK, Unigene15784_YK, Unigene157_YK, Unigene158_YK, Unigene16000_YK, Unigene16094_YK, Unigene16406_YK, Unigene18164_YK, Unigene1964_YK, Unigene2121_YK, Unigene2148_YK, Unigene2231_YK, Unigene2289_YK, Unigene258_YK, Unigene2630_YK, Unigene2685_YK, Unigene3200_YK, Unigene331_YK, Unigene3427_YK, Unigene3585_YK, Unigene3596_YK, Unigene3727_YK, Unigene3885_YK, Unigene3937_YK, Unigene4252_YK, Unigene4291_YK, Unigene4564_YK, Unigene4565_YK, Unigene4772_YK, Unigene5438_YK, Unigene5476_YK, Unigene5477_YK, Unigene5780_YK, Unigene5781_YK, Unigene5857_YK, Unigene5980_YK, Unigene6206_YK, Unigene6504_YK, Unigene6921_YK, Unigene7095_YK, Unigene7391_YK, Unigene7412_YK, Unigene7481_YK, Unigene7525_YK, Unigene765_YK, Unigene8485_YK, Unigene8486_YK, Unigene8487_YK, Unigene9071_YK, Unigene9225_YK, Unigene9702_YK, Unigene9710_YK |
| 51 | [mRNA surveillance pathway](../../../../D:%5C高通量测序结果%5CF14FTSSCKF1242_NEMpnqE%5Cannotation%5CKEGG%5CYK-Unigene.fa_map%5Cmap03015.html) | CL1026.Contig1_YK, CL1026.Contig2_YK, CL1114.Contig1_YK, CL1114.Contig2_YK, CL1114.Contig4_YK, CL1114.Contig5_YK, CL1114.Contig6_YK, CL1114.Contig9_YK, CL1115.Contig2_YK, CL1134.Contig1_YK, CL1134.Contig2_YK, CL1244.Contig2_YK, CL1244.Contig3_YK, CL1324.Contig3_YK, CL1404.Contig2_YK, CL1412.Contig1_YK, CL1451.Contig1_YK, CL1451.Contig2_YK, CL146.Contig2_YK, CL146.Contig4_YK, CL1474.Contig1_YK, CL1814.Contig2_YK, CL1840.Contig1_YK, CL1840.Contig2_YK, CL1897.Contig1_YK, CL1897.Contig2_YK, CL1897.Contig3_YK, CL1949.Contig1_YK, CL1949.Contig2_YK, CL2267.Contig1_YK, CL2267.Contig2_YK, CL2267.Contig3_YK, CL2317.Contig1_YK, CL2317.Contig2_YK, CL2324.Contig1_YK, CL2324.Contig3_YK, CL2470.Contig2_YK, CL2554.Contig1_YK, CL2554.Contig2_YK, CL2587.Contig2_YK, CL27.Contig1_YK, CL2788.Contig1_YK, CL2788.Contig2_YK, CL312.Contig1_YK, CL34.Contig1_YK, CL388.Contig1_YK, CL388.Contig2_YK, CL388.Contig3_YK, CL388.Contig4_YK, CL477.Contig1_YK, CL500.Contig1_YK, CL606.Contig1_YK, CL606.Contig2_YK, CL606.Contig3_YK, CL662.Contig1_YK, CL662.Contig2_YK, CL783.Contig1_YK, CL783.Contig2_YK, CL783.Contig3_YK, CL889.Contig1_YK, CL889.Contig2_YK, CL889.Contig3_YK, CL889.Contig4_YK, CL935.Contig1_YK, CL935.Contig2_YK, CL95.Contig2_YK, CL964.Contig1_YK, CL964.Contig2_YK, CL964.Contig3_YK, CL964.Contig4_YK, Unigene10011_YK, Unigene102_YK, Unigene10683_YK, Unigene10696_YK, Unigene11140_YK, Unigene11141_YK, Unigene11321_YK, Unigene11447_YK, Unigene11662_YK, Unigene12128_YK, Unigene12458_YK, Unigene12583_YK, Unigene12733_YK, Unigene12790_YK, Unigene13069_YK, Unigene13160_YK, Unigene13180_YK, Unigene13193_YK, Unigene13250_YK, Unigene13325_YK, Unigene13340_YK, Unigene13343_YK, Unigene13432_YK, Unigene13470_YK, Unigene13484_YK, Unigene13516_YK, Unigene13542_YK, Unigene13557_YK, Unigene13660_YK, Unigene13685_YK, Unigene13849_YK, Unigene14003_YK, Unigene14156_YK, Unigene14252_YK, Unigene14272_YK, Unigene14276_YK, Unigene14464_YK, Unigene14603_YK, Unigene14691_YK, Unigene14703_YK, Unigene147_YK, Unigene14838_YK, Unigene15015_YK, Unigene15204_YK, Unigene15307_YK, Unigene15335_YK, Unigene15348_YK, Unigene15427_YK, Unigene15630_YK, Unigene15642_YK, Unigene15899_YK, Unigene16112_YK, Unigene16217_YK, Unigene201_YK, Unigene2080_YK, Unigene2100_YK, Unigene2173_YK, Unigene2749_YK, Unigene2792_YK, Unigene2866_YK, Unigene2949_YK, Unigene312_YK, Unigene3138_YK, Unigene3199_YK, Unigene3214_YK, Unigene3343_YK, Unigene337_YK, Unigene3395_YK, Unigene3518_YK, Unigene3601_YK, Unigene3880_YK, Unigene3960_YK, Unigene4179_YK, Unigene4181_YK, Unigene4248_YK, Unigene4340_YK, Unigene4525_YK, Unigene4689_YK, Unigene4690_YK, Unigene4750_YK, Unigene4794_YK, Unigene5010_YK, Unigene5206_YK, Unigene5370_YK, Unigene5385_YK, Unigene5386_YK, Unigene5467_YK, Unigene5468_YK, Unigene5755_YK, Unigene577_YK, Unigene5815_YK, Unigene5835_YK, Unigene5836_YK, Unigene5977_YK, Unigene5989_YK, Unigene5990_YK, Unigene6040_YK, Unigene6205_YK, Unigene6334_YK, Unigene6367_YK, Unigene6368_YK, Unigene6411_YK, Unigene6524_YK, Unigene6635_YK, Unigene6664_YK, Unigene6704_YK, Unigene6877_YK, Unigene6965_YK, Unigene7012_YK, Unigene7047_YK, Unigene7082_YK, Unigene7153_YK, Unigene7370_YK, Unigene7594_YK, Unigene7875_YK, Unigene838_YK, Unigene8490_YK, Unigene8491_YK, Unigene8492_YK, Unigene8691_YK, Unigene8692_YK, Unigene9258_YK, Unigene9259_YK, Unigene9557_YK, Unigene9625_YK |
| 52 | [Phagosome](../../../../D:%5C高通量测序结果%5CF14FTSSCKF1242_NEMpnqE%5Cannotation%5CKEGG%5CYK-Unigene.fa_map%5Cmap04145.html) | CL1002.Contig1_YK, CL1002.Contig2_YK, CL1008.Contig1_YK, CL1008.Contig2_YK, CL1146.Contig1_YK, CL1146.Contig2_YK, CL1221.Contig1_YK, CL1221.Contig2_YK, CL1221.Contig3_YK, CL1286.Contig1_YK, CL1286.Contig2_YK, CL1286.Contig3_YK, CL1286.Contig4_YK, CL1286.Contig5_YK, CL1286.Contig6_YK, CL1286.Contig7_YK, CL1286.Contig8_YK, CL1310.Contig3_YK, CL1310.Contig5_YK, CL1367.Contig1_YK, CL1367.Contig2_YK, CL1381.Contig1_YK, CL1381.Contig2_YK, CL1760.Contig1_YK, CL1817.Contig1_YK, CL1817.Contig2_YK, CL1826.Contig1_YK, CL1826.Contig2_YK, CL2248.Contig1_YK, CL2248.Contig2_YK, CL2275.Contig1_YK, CL2275.Contig2_YK, CL2414.Contig1_YK, CL2414.Contig2_YK, CL2452.Contig1_YK, CL2452.Contig2_YK, CL2505.Contig1_YK, CL2505.Contig2_YK, CL2897.Contig1_YK, CL2897.Contig2_YK, CL2905.Contig1_YK, CL2905.Contig2_YK, CL353.Contig1_YK, CL353.Contig3_YK, CL353.Contig6_YK, CL366.Contig1_YK, CL366.Contig2_YK, CL366.Contig3_YK, CL707.Contig1_YK, CL707.Contig2_YK, CL809.Contig1_YK, CL809.Contig2_YK, CL832.Contig1_YK, CL84.Contig2_YK, CL84.Contig3_YK, Unigene10213_YK, Unigene10224_YK, Unigene10262_YK, Unigene10263_YK, Unigene10604_YK, Unigene10644_YK, Unigene10694_YK, Unigene10927_YK, Unigene11196_YK, Unigene11246_YK, Unigene11332_YK, Unigene11439_YK, Unigene11451_YK, Unigene11714_YK, Unigene11715_YK, Unigene11716_YK, Unigene11718_YK, Unigene11719_YK, Unigene11805_YK, Unigene11806_YK, Unigene11807_YK, Unigene11808_YK, Unigene11916_YK, Unigene12690_YK, Unigene12691_YK, Unigene12784_YK, Unigene12898_YK, Unigene12899_YK, Unigene13024_YK, Unigene13046_YK, Unigene13074_YK, Unigene13137_YK, Unigene13161_YK, Unigene13210_YK, Unigene13232_YK, Unigene13274_YK, Unigene13296_YK, Unigene13327_YK, Unigene13342_YK, Unigene13405_YK, Unigene13527_YK, Unigene13572_YK, Unigene13582_YK, Unigene13636_YK, Unigene13652_YK, Unigene13720_YK, Unigene13756_YK, Unigene13811_YK, Unigene13840_YK, Unigene13963_YK, Unigene14016_YK, Unigene14019_YK, Unigene14220_YK, Unigene14327_YK, Unigene14379_YK, Unigene14381_YK, Unigene14559_YK, Unigene14661_YK, Unigene14729_YK, Unigene14929_YK, Unigene15055_YK, Unigene15062_YK, Unigene15076_YK, Unigene15246_YK, Unigene15461_YK, Unigene1554_YK, Unigene1603_YK, Unigene1611_YK, Unigene16250_YK, Unigene16869_YK, Unigene16914_YK, Unigene1700_YK, Unigene17236_YK, Unigene17385_YK, Unigene17660_YK, Unigene17867_YK, Unigene1787_YK, Unigene1788_YK, Unigene18183_YK, Unigene18316_YK, Unigene18388_YK, Unigene18577_YK, Unigene18999_YK, Unigene19065_YK, Unigene2366_YK, Unigene2578_YK, Unigene2704_YK, Unigene2849_YK, Unigene3067_YK, Unigene3119_YK, Unigene3279_YK, Unigene3381_YK, Unigene3621_YK, Unigene3761_YK, Unigene3769_YK, Unigene4203_YK, Unigene4268_YK, Unigene4471_YK, Unigene4494_YK, Unigene4769_YK, Unigene476_YK, Unigene4825_YK, Unigene4924_YK, Unigene6000_YK, Unigene6001_YK, Unigene6002_YK, Unigene6010_YK, Unigene6012_YK, Unigene6038_YK, Unigene6112_YK, Unigene6144_YK, Unigene6166_YK, Unigene6209_YK, Unigene6340_YK, Unigene6341_YK, Unigene6350_YK, Unigene6351_YK, Unigene6363_YK, Unigene6443_YK, Unigene6561_YK, Unigene665_YK, Unigene6890_YK, Unigene69_YK, Unigene7053_YK, Unigene7054_YK, Unigene7191_YK, Unigene737_YK, Unigene738_YK, Unigene7397_YK, Unigene7610_YK, Unigene7611_YK, Unigene8289_YK, Unigene8483_YK, Unigene8495_YK, Unigene9232_YK, Unigene9233_YK, Unigene9504_YK, Unigene9505_YK, Unigene9957_YK |
| 53 | [Gap junction](../../../../D:%5C高通量测序结果%5CF14FTSSCKF1242_NEMpnqE%5Cannotation%5CKEGG%5CYK-Unigene.fa_map%5Cmap04540.html) | CL1041.Contig1_YK, CL1041.Contig2_YK, CL1088.Contig1_YK, CL1088.Contig2_YK, CL1146.Contig1_YK, CL1146.Contig2_YK, CL1292.Contig1_YK, CL1292.Contig2_YK, CL1367.Contig1_YK, CL1367.Contig2_YK, CL15.Contig10_YK, CL15.Contig11_YK, CL15.Contig12_YK, CL15.Contig13_YK, CL15.Contig14_YK, CL15.Contig15_YK, CL15.Contig16_YK, CL15.Contig17_YK, CL15.Contig18_YK, CL15.Contig19_YK, CL15.Contig1_YK, CL15.Contig20_YK, CL15.Contig21_YK, CL15.Contig22_YK, CL15.Contig23_YK, CL15.Contig24_YK, CL15.Contig2_YK, CL15.Contig3_YK, CL15.Contig4_YK, CL15.Contig5_YK, CL15.Contig6_YK, CL15.Contig7_YK, CL15.Contig8_YK, CL15.Contig9_YK, CL1553.Contig1_YK, CL1553.Contig2_YK, CL1553.Contig3_YK, CL1666.Contig1_YK, CL1666.Contig2_YK, CL2199.Contig1_YK, CL2199.Contig2_YK, CL2199.Contig3_YK, CL2240.Contig1_YK, CL2240.Contig2_YK, CL2248.Contig1_YK, CL2248.Contig2_YK, CL2333.Contig1_YK, CL2333.Contig2_YK, CL2429.Contig1_YK, CL2429.Contig2_YK, CL2447.Contig1_YK, CL2447.Contig2_YK, CL2449.Contig1_YK, CL2532.Contig1_YK, CL2532.Contig2_YK, CL255.Contig1_YK, CL255.Contig2_YK, CL255.Contig3_YK, CL255.Contig4_YK, CL255.Contig5_YK, CL255.Contig6_YK, CL255.Contig7_YK, CL255.Contig8_YK, CL2783.Contig1_YK, CL2897.Contig1_YK, CL2897.Contig2_YK, CL295.Contig10_YK, CL295.Contig2_YK, CL295.Contig3_YK, CL295.Contig4_YK, CL295.Contig6_YK, CL295.Contig8_YK, CL295.Contig9_YK, CL307.Contig10_YK, CL307.Contig11_YK, CL307.Contig12_YK, CL307.Contig1_YK, CL307.Contig2_YK, CL307.Contig3_YK, CL307.Contig4_YK, CL307.Contig5_YK, CL307.Contig6_YK, CL307.Contig7_YK, CL307.Contig8_YK, CL307.Contig9_YK, CL366.Contig1_YK, CL366.Contig2_YK, CL366.Contig3_YK, CL591.Contig1_YK, CL591.Contig2_YK, CL591.Contig3_YK, CL591.Contig4_YK, CL591.Contig5_YK, CL591.Contig6_YK, CL591.Contig7_YK, CL591.Contig8_YK, CL592.Contig2_YK, CL79.Contig1_YK, CL79.Contig2_YK, CL79.Contig3_YK, CL79.Contig4_YK, CL79.Contig5_YK, CL79.Contig6_YK, CL79.Contig7_YK, CL79.Contig8_YK, CL884.Contig1_YK, CL884.Contig3_YK, Unigene10197_YK, Unigene10198_YK, Unigene10493_YK, Unigene107_YK, Unigene10829_YK, Unigene10908_YK, Unigene11196_YK, Unigene11714_YK, Unigene11715_YK, Unigene11716_YK, Unigene11805_YK, Unigene11806_YK, Unigene11807_YK, Unigene11808_YK, Unigene12216_YK, Unigene12219_YK, Unigene1223_YK, Unigene12292_YK, Unigene12293_YK, Unigene12560_YK, Unigene12561_YK, Unigene12563_YK, Unigene12564_YK, Unigene12636_YK, Unigene12638_YK, Unigene12639_YK, Unigene12690_YK, Unigene12691_YK, Unigene13237_YK, Unigene13674_YK, Unigene13969_YK, Unigene14278_YK, Unigene14345_YK, Unigene14456_YK, Unigene158_YK, Unigene15910_YK, Unigene16406_YK, Unigene16420_YK, Unigene16951_YK, Unigene17385_YK, Unigene18164_YK, Unigene18388_YK, Unigene18798_YK, Unigene19065_YK, Unigene1964_YK, Unigene2231_YK, Unigene2289_YK, Unigene2324_YK, Unigene258_YK, Unigene331_YK, Unigene3585_YK, Unigene372_YK, Unigene3937_YK, Unigene397_YK, Unigene4252_YK, Unigene4680_YK, Unigene5219_YK, Unigene5438_YK, Unigene5476_YK, Unigene5477_YK, Unigene5577_YK, Unigene5642_YK, Unigene5857_YK, Unigene6038_YK, Unigene6206_YK, Unigene6407_YK, Unigene6444_YK, Unigene6561_YK, Unigene6600_YK, Unigene6980_YK, Unigene708_YK, Unigene7337_YK, Unigene7525_YK, Unigene7822_YK, Unigene7823_YK, Unigene7824_YK, Unigene8101_YK, Unigene8289_YK, Unigene8745_YK, Unigene8815_YK, Unigene904_YK, Unigene905_YK, Unigene9198_YK, Unigene9710_YK, Unigene9957_YK |
| 54 | [Ribosome](../../../../D:%5C高通量测序结果%5CF14FTSSCKF1242_NEMpnqE%5Cannotation%5CKEGG%5CYK-Unigene.fa_map%5Cmap03010.html) | CL1011.Contig1_YK, CL105.Contig1_YK, CL1369.Contig1_YK, CL1600.Contig1_YK, CL1692.Contig1_YK, CL1716.Contig1_YK, CL1716.Contig2_YK, CL1716.Contig3_YK, CL1761.Contig1_YK, CL1761.Contig2_YK, CL1780.Contig1_YK, CL1981.Contig1_YK, CL1981.Contig2_YK, CL2236.Contig1_YK, CL2236.Contig2_YK, CL226.Contig1_YK, CL226.Contig2_YK, CL2266.Contig1_YK, CL2298.Contig1_YK, CL2351.Contig1_YK, CL2485.Contig1_YK, CL2485.Contig2_YK, CL2487.Contig1_YK, CL2498.Contig1_YK, CL2498.Contig2_YK, CL2511.Contig1_YK, CL2511.Contig2_YK, CL2617.Contig1_YK, CL2654.Contig1_YK, CL2654.Contig2_YK, CL2663.Contig1_YK, CL2686.Contig8_YK, CL2709.Contig1_YK, CL2724.Contig1_YK, CL273.Contig1_YK, CL273.Contig2_YK, CL2748.Contig1_YK, CL2765.Contig1_YK, CL2765.Contig2_YK, CL2784.Contig1_YK, CL2836.Contig1_YK, CL2836.Contig2_YK, CL2850.Contig2_YK, CL557.Contig1_YK, CL557.Contig2_YK, CL678.Contig1_YK, CL795.Contig1_YK, CL795.Contig2_YK, Unigene10339_YK, Unigene10956_YK, Unigene1115_YK, Unigene11219_YK, Unigene11857_YK, Unigene12080_YK, Unigene1233_YK, Unigene1264_YK, Unigene12754_YK, Unigene1292_YK, Unigene12989_YK, Unigene12995_YK, Unigene12996_YK, Unigene12998_YK, Unigene12999_YK, Unigene13002_YK, Unigene13005_YK, Unigene13011_YK, Unigene13016_YK, Unigene13018_YK, Unigene13021_YK, Unigene13025_YK, Unigene13029_YK, Unigene13031_YK, Unigene13033_YK, Unigene13035_YK, Unigene13040_YK, Unigene13044_YK, Unigene13045_YK, Unigene13051_YK, Unigene13062_YK, Unigene13067_YK, Unigene13068_YK, Unigene13072_YK, Unigene13075_YK, Unigene13078_YK, Unigene13096_YK, Unigene13131_YK, Unigene13186_YK, Unigene13195_YK, Unigene13244_YK, Unigene13265_YK, Unigene13328_YK, Unigene13505_YK, Unigene13587_YK, Unigene13689_YK, Unigene13931_YK, Unigene13994_YK, Unigene16273_YK, Unigene16362_YK, Unigene16416_YK, Unigene16424_YK, Unigene16437_YK, Unigene16461_YK, Unigene16501_YK, Unigene16539_YK, Unigene16593_YK, Unigene16596_YK, Unigene16610_YK, Unigene16624_YK, Unigene16674_YK, Unigene16700_YK, Unigene16730_YK, Unigene16745_YK, Unigene16749_YK, Unigene16775_YK, Unigene16816_YK, Unigene16854_YK, Unigene16863_YK, Unigene16873_YK, Unigene16877_YK, Unigene16886_YK, Unigene16897_YK, Unigene16920_YK, Unigene16922_YK, Unigene16934_YK, Unigene16988_YK, Unigene17041_YK, Unigene17049_YK, Unigene17088_YK, Unigene17133_YK, Unigene17162_YK, Unigene17243_YK, Unigene17250_YK, Unigene17275_YK, Unigene17345_YK, Unigene17381_YK, Unigene17425_YK, Unigene17429_YK, Unigene17431_YK, Unigene17438_YK, Unigene17518_YK, Unigene17545_YK, Unigene17593_YK, Unigene17681_YK, Unigene17748_YK, Unigene17946_YK, Unigene17948_YK, Unigene18004_YK, Unigene18399_YK, Unigene18463_YK, Unigene18497_YK, Unigene1878_YK, Unigene1908_YK, Unigene1959_YK, Unigene3115_YK, Unigene3125_YK, Unigene3184_YK, Unigene3191_YK, Unigene3212_YK, Unigene3534_YK, Unigene4007_YK, Unigene4253_YK, Unigene4254_YK, Unigene4328_YK, Unigene4329_YK, Unigene4373_YK, Unigene4374_YK, Unigene458_YK, Unigene4694_YK, Unigene4894_YK, Unigene4895_YK, Unigene5170_YK, Unigene611_YK, Unigene6266_YK, Unigene6291_YK, Unigene671_YK, Unigene672_YK, Unigene6980_YK, Unigene720_YK, Unigene7360_YK, Unigene7361_YK, Unigene739_YK, Unigene740_YK, Unigene7512_YK, Unigene7709_YK, Unigene7827_YK, Unigene8673_YK, Unigene8674_YK, Unigene8880_YK, Unigene8881_YK, Unigene9618_YK, Unigene9619_YK |
| 55 | [Phosphatidylinositol signaling system](../../../../D:%5C高通量测序结果%5CF14FTSSCKF1242_NEMpnqE%5Cannotation%5CKEGG%5CYK-Unigene.fa_map%5Cmap04070.html) | CL1062.Contig10_YK, CL1062.Contig11_YK, CL1062.Contig1_YK, CL1062.Contig2_YK, CL1062.Contig3_YK, CL1062.Contig4_YK, CL1062.Contig5_YK, CL1062.Contig6_YK, CL1062.Contig7_YK, CL1062.Contig8_YK, CL1062.Contig9_YK, CL1101.Contig1_YK, CL1101.Contig2_YK, CL1101.Contig3_YK, CL1101.Contig4_YK, CL1379.Contig1_YK, CL1379.Contig2_YK, CL15.Contig10_YK, CL15.Contig11_YK, CL15.Contig12_YK, CL15.Contig13_YK, CL15.Contig14_YK, CL15.Contig15_YK, CL15.Contig16_YK, CL15.Contig17_YK, CL15.Contig18_YK, CL15.Contig19_YK, CL15.Contig1_YK, CL15.Contig20_YK, CL15.Contig21_YK, CL15.Contig22_YK, CL15.Contig23_YK, CL15.Contig24_YK, CL15.Contig2_YK, CL15.Contig3_YK, CL15.Contig4_YK, CL15.Contig5_YK, CL15.Contig6_YK, CL15.Contig7_YK, CL15.Contig8_YK, CL15.Contig9_YK, CL1500.Contig1_YK, CL1500.Contig2_YK, CL1500.Contig3_YK, CL1714.Contig2_YK, CL1861.Contig1_YK, CL1903.Contig1_YK, CL1903.Contig2_YK, CL2438.Contig1_YK, CL2438.Contig2_YK, CL2493.Contig1_YK, CL2504.Contig1_YK, CL2504.Contig2_YK, CL2593.Contig1_YK, CL2593.Contig2_YK, CL2593.Contig3_YK, CL2792.Contig1_YK, CL295.Contig10_YK, CL295.Contig11_YK, CL295.Contig1_YK, CL295.Contig2_YK, CL295.Contig3_YK, CL295.Contig4_YK, CL295.Contig5_YK, CL295.Contig6_YK, CL295.Contig7_YK, CL295.Contig8_YK, CL295.Contig9_YK, CL307.Contig10_YK, CL307.Contig11_YK, CL307.Contig12_YK, CL307.Contig1_YK, CL307.Contig2_YK, CL307.Contig3_YK, CL307.Contig4_YK, CL307.Contig5_YK, CL307.Contig6_YK, CL307.Contig7_YK, CL307.Contig8_YK, CL307.Contig9_YK, CL447.Contig1_YK, CL447.Contig2_YK, CL447.Contig3_YK, CL475.Contig1_YK, CL475.Contig2_YK, CL475.Contig3_YK, CL475.Contig4_YK, CL568.Contig1_YK, CL568.Contig2_YK, CL591.Contig1_YK, CL591.Contig2_YK, CL591.Contig3_YK, CL591.Contig4_YK, CL591.Contig5_YK, CL591.Contig6_YK, CL591.Contig7_YK, CL591.Contig8_YK, CL86.Contig1_YK, CL86.Contig2_YK, CL86.Contig3_YK, CL86.Contig4_YK, CL86.Contig5_YK, CL86.Contig6_YK, CL86.Contig7_YK, CL86.Contig8_YK, CL86.Contig9_YK, CL884.Contig1_YK, CL884.Contig3_YK, CL902.Contig1_YK, CL902.Contig2_YK, Unigene10826_YK, Unigene10827_YK, Unigene10828_YK, Unigene10972_YK, Unigene11076_YK, Unigene11668_YK, Unigene11669_YK, Unigene11938_YK, Unigene12560_YK, Unigene12561_YK, Unigene12562_YK, Unigene12563_YK, Unigene12564_YK, Unigene12565_YK, Unigene12636_YK, Unigene12638_YK, Unigene12639_YK, Unigene12784_YK, Unigene13744_YK, Unigene13937_YK, Unigene13943_YK, Unigene13960_YK, Unigene14087_YK, Unigene14243_YK, Unigene14559_YK, Unigene15057_YK, Unigene15091_YK, Unigene15497_YK, Unigene15550_YK, Unigene15587_YK, Unigene158_YK, Unigene16474_YK, Unigene16804_YK, Unigene17117_YK, Unigene2550_YK, Unigene2752_YK, Unigene2791_YK, Unigene29_YK, Unigene3153_YK, Unigene3626_YK, Unigene3963_YK, Unigene3964_YK, Unigene3972_YK, Unigene4102_YK, Unigene4252_YK, Unigene4256_YK, Unigene4257_YK, Unigene4688_YK, Unigene5462_YK, Unigene5476_YK, Unigene5477_YK, Unigene5546_YK, Unigene5604_YK, Unigene61_YK, Unigene6227_YK, Unigene6461_YK, Unigene6611_YK, Unigene6795_YK, Unigene6920_YK, Unigene6_YK, Unigene7183_YK, Unigene7184_YK, Unigene7185_YK, Unigene7307_YK, Unigene7308_YK, Unigene7525_YK, Unigene7844_YK, Unigene7845_YK, Unigene7898_YK, Unigene7899_YK, Unigene7900_YK, Unigene7901_YK, Unigene8158_YK, Unigene8323_YK, Unigene8324_YK, Unigene8325_YK, Unigene8327_YK, Unigene8867_YK, Unigene8868_YK, Unigene9459_YK |
| 56 | [Viral myocarditis](../../../../D:%5C高通量测序结果%5CF14FTSSCKF1242_NEMpnqE%5Cannotation%5CKEGG%5CYK-Unigene.fa_map%5Cmap05416.html) | CL1019.Contig1_YK, CL1019.Contig2_YK, CL1019.Contig3_YK, CL1019.Contig4_YK, CL1156.Contig3_YK, CL1221.Contig1_YK, CL1221.Contig2_YK, CL1221.Contig3_YK, CL124.Contig1_YK, CL124.Contig2_YK, CL124.Contig3_YK, CL1303.Contig1_YK, CL1303.Contig2_YK, CL1303.Contig3_YK, CL1381.Contig1_YK, CL1381.Contig2_YK, CL1486.Contig2_YK, CL1577.Contig1_YK, CL1577.Contig2_YK, CL1577.Contig3_YK, CL1577.Contig4_YK, CL1578.Contig2_YK, CL1591.Contig1_YK, CL1761.Contig1_YK, CL1761.Contig2_YK, CL181.Contig1_YK, CL181.Contig2_YK, CL1898.Contig1_YK, CL1898.Contig2_YK, CL1920.Contig1_YK, CL1920.Contig2_YK, CL1998.Contig1_YK, CL2051.Contig1_YK, CL2051.Contig3_YK, CL2294.Contig1_YK, CL2294.Contig2_YK, CL2560.Contig1_YK, CL2560.Contig2_YK, CL2648.Contig1_YK, CL2669.Contig1_YK, CL2669.Contig2_YK, CL2669.Contig3_YK, CL2669.Contig4_YK, CL2669.Contig5_YK, CL2774.Contig2_YK, CL2798.Contig1_YK, CL2798.Contig2_YK, CL2905.Contig1_YK, CL2905.Contig2_YK, CL344.Contig3_YK, CL369.Contig2_YK, CL369.Contig5_YK, CL369.Contig8_YK, CL37.Contig2_YK, CL487.Contig1_YK, CL487.Contig2_YK, CL502.Contig1_YK, CL502.Contig2_YK, CL503.Contig1_YK, CL599.Contig1_YK, CL599.Contig2_YK, CL75.Contig1_YK, CL75.Contig2_YK, CL75.Contig3_YK, CL832.Contig1_YK, CL84.Contig2_YK, CL84.Contig3_YK, CL91.Contig1_YK, CL91.Contig2_YK, CL91.Contig3_YK, CL91.Contig4_YK, CL91.Contig5_YK, CL91.Contig6_YK, CL91.Contig7_YK, CL95.Contig2_YK, Unigene10251_YK, Unigene10291_YK, Unigene10355_YK, Unigene10695_YK, Unigene10927_YK, Unigene11532_YK, Unigene11884_YK, Unigene11931_YK, Unigene12027_YK, Unigene12334_YK, Unigene12335_YK, Unigene12336_YK, Unigene12337_YK, Unigene12361_YK, Unigene12426_YK, Unigene12491_YK, Unigene12492_YK, Unigene12493_YK, Unigene12494_YK, Unigene12532_YK, Unigene12743_YK, Unigene12898_YK, Unigene12899_YK, Unigene13245_YK, Unigene13598_YK, Unigene13704_YK, Unigene13720_YK, Unigene13811_YK, Unigene13890_YK, Unigene13920_YK, Unigene14005_YK, Unigene14016_YK, Unigene14254_YK, Unigene14327_YK, Unigene14403_YK, Unigene14420_YK, Unigene14657_YK, Unigene14661_YK, Unigene14729_YK, Unigene14735_YK, Unigene14929_YK, Unigene14966_YK, Unigene15034_YK, Unigene15271_YK, Unigene15308_YK, Unigene15315_YK, Unigene15804_YK, Unigene15891_YK, Unigene15961_YK, Unigene16018_YK, Unigene16105_YK, Unigene1611_YK, Unigene16250_YK, Unigene16458_YK, Unigene16741_YK, Unigene16862_YK, Unigene16869_YK, Unigene17458_YK, Unigene18183_YK, Unigene18316_YK, Unigene18493_YK, Unigene18577_YK, Unigene18679_YK, Unigene2207_YK, Unigene2436_YK, Unigene2620_YK, Unigene2719_YK, Unigene2808_YK, Unigene2849_YK, Unigene2869_YK, Unigene2892_YK, Unigene3026_YK, Unigene3034_YK, Unigene3067_YK, Unigene3188_YK, Unigene3189_YK, Unigene3268_YK, Unigene3271_YK, Unigene3384_YK, Unigene3629_YK, Unigene3650_YK, Unigene370_YK, Unigene3739_YK, Unigene3740_YK, Unigene3906_YK, Unigene3916_YK, Unigene4055_YK, Unigene4074_YK, Unigene4203_YK, Unigene4315_YK, Unigene4494_YK, Unigene4747_YK, Unigene4825_YK, Unigene4900_YK, Unigene5300_YK, Unigene6146_YK, Unigene6469_YK, Unigene6814_YK, Unigene6850_YK, Unigene7448_YK, Unigene7483_YK, Unigene7610_YK, Unigene7611_YK, Unigene7662_YK, Unigene7684_YK, Unigene836_YK, Unigene8371_YK, Unigene8755_YK, Unigene8997_YK, Unigene9373_YK, Unigene9634_YK, Unigene9937_YK, Unigene9989_YK |
| 57 | [Progesterone-mediated oocyte maturation](../../../../D:%5C高通量测序结果%5CF14FTSSCKF1242_NEMpnqE%5Cannotation%5CKEGG%5CYK-Unigene.fa_map%5Cmap04914.html) | CL1088.Contig1_YK, CL1088.Contig2_YK, CL11.Contig3_YK, CL110.Contig1_YK, CL110.Contig2_YK, CL1117.Contig1_YK, CL1119.Contig1_YK, CL1119.Contig2_YK, CL1207.Contig1_YK, CL1207.Contig2_YK, CL1207.Contig3_YK, CL1207.Contig4_YK, CL1207.Contig5_YK, CL1412.Contig1_YK, CL1412.Contig2_YK, CL1412.Contig3_YK, CL1457.Contig1_YK, CL1457.Contig2_YK, CL1464.Contig1_YK, CL1531.Contig1_YK, CL1531.Contig2_YK, CL155.Contig1_YK, CL155.Contig2_YK, CL1602.Contig1_YK, CL1602.Contig2_YK, CL1659.Contig1_YK, CL1659.Contig2_YK, CL174.Contig1_YK, CL1856.Contig1_YK, CL1856.Contig2_YK, CL1910.Contig1_YK, CL1918.Contig1_YK, CL1918.Contig2_YK, CL2069.Contig1_YK, CL2135.Contig2_YK, CL2168.Contig1_YK, CL2199.Contig1_YK, CL2199.Contig2_YK, CL2199.Contig3_YK, CL2532.Contig1_YK, CL2532.Contig2_YK, CL255.Contig1_YK, CL255.Contig2_YK, CL255.Contig3_YK, CL255.Contig4_YK, CL255.Contig5_YK, CL255.Contig6_YK, CL255.Contig7_YK, CL255.Contig8_YK, CL2624.Contig1_YK, CL2624.Contig2_YK, CL2764.Contig1_YK, CL2764.Contig2_YK, CL2792.Contig1_YK, CL2898.Contig2_YK, CL376.Contig1_YK, CL376.Contig2_YK, CL376.Contig3_YK, CL376.Contig4_YK, CL376.Contig5_YK, CL376.Contig6_YK, CL79.Contig1_YK, CL79.Contig2_YK, CL79.Contig3_YK, CL79.Contig4_YK, CL79.Contig5_YK, CL79.Contig6_YK, CL79.Contig7_YK, CL79.Contig8_YK, CL816.Contig1_YK, CL841.Contig3_YK, CL841.Contig4_YK, CL841.Contig5_YK, CL841.Contig6_YK, CL841.Contig7_YK, CL841.Contig8_YK, CL93.Contig1_YK, Unigene10000_YK, Unigene10173_YK, Unigene104_YK, Unigene10534_YK, Unigene10623_YK, Unigene10818_YK, Unigene10819_YK, Unigene10908_YK, Unigene10972_YK, Unigene11335_YK, Unigene11548_YK, Unigene11566_YK, Unigene11863_YK, Unigene12058_YK, Unigene12216_YK, Unigene12218_YK, Unigene12219_YK, Unigene12292_YK, Unigene12293_YK, Unigene12451_YK, Unigene12512_YK, Unigene12777_YK, Unigene12866_YK, Unigene12887_YK, Unigene12911_YK, Unigene13657_YK, Unigene13674_YK, Unigene13915_YK, Unigene13969_YK, Unigene14345_YK, Unigene14408_YK, Unigene14425_YK, Unigene14601_YK, Unigene14612_YK, Unigene14969_YK, Unigene14980_YK, Unigene15000_YK, Unigene15034_YK, Unigene15497_YK, Unigene15505_YK, Unigene15836_YK, Unigene15910_YK, Unigene16406_YK, Unigene16653_YK, Unigene1673_YK, Unigene1719_YK, Unigene17452_YK, Unigene17689_YK, Unigene18164_YK, Unigene18210_YK, Unigene18319_YK, Unigene18590_YK, Unigene18630_YK, Unigene18955_YK, Unigene2033_YK, Unigene2231_YK, Unigene2289_YK, Unigene258_YK, Unigene2600_YK, Unigene2646_YK, Unigene2736_YK, Unigene2791_YK, Unigene2863_YK, Unigene3165_YK, Unigene3174_YK, Unigene331_YK, Unigene3428_YK, Unigene3491_YK, Unigene3526_YK, Unigene3937_YK, Unigene4086_YK, Unigene4114_YK, Unigene4166_YK, Unigene4436_YK, Unigene4680_YK, Unigene4684_YK, Unigene4767_YK, Unigene4799_YK, Unigene485_YK, Unigene486_YK, Unigene5219_YK, Unigene5438_YK, Unigene5617_YK, Unigene6089_YK, Unigene6229_YK, Unigene6230_YK, Unigene6231_YK, Unigene6318_YK, Unigene6561_YK, Unigene6729_YK, Unigene6859_YK, Unigene6971_YK, Unigene7500_YK, Unigene7959_YK, Unigene8065_YK, Unigene8323_YK, Unigene8324_YK, Unigene8325_YK, Unigene8583_YK, Unigene8909_YK, Unigene9071_YK, Unigene9390_YK, Unigene9706_YK, Unigene9710_YK |
| 58 | [Influenza A](../../../../D:%5C高通量测序结果%5CF14FTSSCKF1242_NEMpnqE%5Cannotation%5CKEGG%5CYK-Unigene.fa_map%5Cmap05164.html) | CL1098.Contig1_YK, CL1098.Contig2_YK, CL1221.Contig1_YK, CL1221.Contig2_YK, CL1221.Contig3_YK, CL1228.Contig1_YK, CL1448.Contig1_YK, CL1448.Contig2_YK, CL1514.Contig1_YK, CL1514.Contig2_YK, CL1758.Contig1_YK, CL1758.Contig2_YK, CL1977.Contig1_YK, CL1977.Contig2_YK, CL1977.Contig3_YK, CL1977.Contig4_YK, CL198.Contig1_YK, CL198.Contig2_YK, CL198.Contig3_YK, CL2008.Contig1_YK, CL2008.Contig2_YK, CL2168.Contig1_YK, CL2267.Contig1_YK, CL2267.Contig2_YK, CL2267.Contig3_YK, CL2294.Contig1_YK, CL2294.Contig2_YK, CL2324.Contig1_YK, CL2324.Contig3_YK, CL2336.Contig1_YK, CL2336.Contig2_YK, CL2624.Contig1_YK, CL2624.Contig2_YK, CL2668.Contig1_YK, CL2668.Contig2_YK, CL2720.Contig1_YK, CL2792.Contig1_YK, CL2793.Contig1_YK, CL2793.Contig2_YK, CL2905.Contig1_YK, CL2905.Contig2_YK, CL383.Contig17_YK, CL383.Contig18_YK, CL387.Contig1_YK, CL387.Contig2_YK, CL387.Contig3_YK, CL387.Contig4_YK, CL387.Contig5_YK, CL587.Contig2_YK, CL591.Contig1_YK, CL591.Contig2_YK, CL591.Contig3_YK, CL591.Contig4_YK, CL591.Contig5_YK, CL591.Contig6_YK, CL591.Contig7_YK, CL591.Contig8_YK, CL774.Contig1_YK, CL774.Contig2_YK, CL815.Contig1_YK, CL815.Contig2_YK, CL815.Contig3_YK, CL815.Contig4_YK, CL815.Contig5_YK, CL815.Contig6_YK, CL83.Contig4_YK, CL84.Contig2_YK, CL84.Contig3_YK, CL882.Contig1_YK, CL882.Contig2_YK, CL882.Contig3_YK, CL882.Contig4_YK, CL882.Contig5_YK, CL882.Contig6_YK, CL882.Contig7_YK, CL884.Contig1_YK, CL884.Contig3_YK, CL956.Contig1_YK, CL956.Contig2_YK, CL956.Contig3_YK, CL956.Contig4_YK, Unigene10015_YK, Unigene10229_YK, Unigene10409_YK, Unigene10431_YK, Unigene10534_YK, Unigene10659_YK, Unigene10972_YK, Unigene11252_YK, Unigene11335_YK, Unigene12133_YK, Unigene12218_YK, Unigene1225_YK, Unigene12777_YK, Unigene12898_YK, Unigene12899_YK, Unigene13014_YK, Unigene13128_YK, Unigene13436_YK, Unigene13650_YK, Unigene13670_YK, Unigene13674_YK, Unigene13720_YK, Unigene13796_YK, Unigene13811_YK, Unigene1393_YK, Unigene13962_YK, Unigene14010_YK, Unigene14252_YK, Unigene14339_YK, Unigene14345_YK, Unigene14415_YK, Unigene14464_YK, Unigene14516_YK, Unigene14709_YK, Unigene14929_YK, Unigene15080_YK, Unigene15273_YK, Unigene15277_YK, Unigene15497_YK, Unigene15561_YK, Unigene15616_YK, Unigene16015_YK, Unigene1656_YK, Unigene1657_YK, Unigene17614_YK, Unigene17725_YK, Unigene18316_YK, Unigene18679_YK, Unigene18931_YK, Unigene19055_YK, Unigene2689_YK, Unigene2791_YK, Unigene2849_YK, Unigene2913_YK, Unigene302_YK, Unigene3067_YK, Unigene3129_YK, Unigene3206_YK, Unigene3355_YK, Unigene3692_YK, Unigene4068_YK, Unigene4124_YK, Unigene4125_YK, Unigene4181_YK, Unigene4648_YK, Unigene4660_YK, Unigene4670_YK, Unigene4_YK, Unigene5219_YK, Unigene5558_YK, Unigene5916_YK, Unigene6071_YK, Unigene6205_YK, Unigene6228_YK, Unigene6229_YK, Unigene6230_YK, Unigene6231_YK, Unigene6334_YK, Unigene6535_YK, Unigene6646_YK, Unigene6664_YK, Unigene7012_YK, Unigene7525_YK, Unigene7816_YK, Unigene7837_YK, Unigene7838_YK, Unigene7839_YK, Unigene8323_YK, Unigene8324_YK, Unigene8325_YK, Unigene8378_YK, Unigene8461_YK, Unigene8835_YK, Unigene9071_YK, Unigene9127_YK, Unigene9968_YK, Unigene9969_YK |
| 59 | [Amphetamine addiction](../../../../D:%5C高通量测序结果%5CF14FTSSCKF1242_NEMpnqE%5Cannotation%5CKEGG%5CYK-Unigene.fa_map%5Cmap05031.html) | CL1026.Contig1_YK, CL1026.Contig2_YK, CL1088.Contig1_YK, CL1088.Contig2_YK, CL1098.Contig1_YK, CL1098.Contig2_YK, CL112.Contig1_YK, CL112.Contig2_YK, CL112.Contig3_YK, CL112.Contig4_YK, CL112.Contig5_YK, CL112.Contig6_YK, CL112.Contig7_YK, CL112.Contig8_YK, CL1412.Contig1_YK, CL1544.Contig1_YK, CL1544.Contig2_YK, CL1653.Contig1_YK, CL1653.Contig2_YK, CL1704.Contig1_YK, CL1714.Contig2_YK, CL1758.Contig1_YK, CL1758.Contig2_YK, CL1861.Contig1_YK, CL1933.Contig1_YK, CL1933.Contig2_YK, CL1968.Contig1_YK, CL1968.Contig2_YK, CL1977.Contig1_YK, CL1977.Contig2_YK, CL1977.Contig3_YK, CL1977.Contig4_YK, CL2135.Contig1_YK, CL2135.Contig2_YK, CL2192.Contig1_YK, CL2192.Contig2_YK, CL255.Contig1_YK, CL255.Contig2_YK, CL255.Contig3_YK, CL255.Contig4_YK, CL255.Contig5_YK, CL255.Contig6_YK, CL255.Contig7_YK, CL255.Contig8_YK, CL2593.Contig1_YK, CL2593.Contig2_YK, CL2593.Contig3_YK, CL2635.Contig1_YK, CL2635.Contig2_YK, CL2793.Contig1_YK, CL2793.Contig2_YK, CL312.Contig1_YK, CL431.Contig1_YK, CL431.Contig2_YK, CL431.Contig3_YK, CL568.Contig1_YK, CL568.Contig2_YK, CL591.Contig1_YK, CL591.Contig2_YK, CL591.Contig3_YK, CL591.Contig4_YK, CL591.Contig5_YK, CL591.Contig6_YK, CL591.Contig7_YK, CL591.Contig8_YK, CL838.Contig1_YK, CL838.Contig2_YK, CL838.Contig3_YK, CL838.Contig4_YK, CL838.Contig5_YK, CL838.Contig8_YK, CL884.Contig1_YK, CL884.Contig3_YK, CL902.Contig1_YK, CL902.Contig2_YK, CL935.Contig1_YK, CL935.Contig2_YK, Unigene10096_YK, Unigene10209_YK, Unigene10210_YK, Unigene10211_YK, Unigene10546_YK, Unigene10547_YK, Unigene10614_YK, Unigene10908_YK, Unigene11140_YK, Unigene11141_YK, Unigene11567_YK, Unigene11568_YK, Unigene11662_YK, Unigene11889_YK, Unigene12021_YK, Unigene12128_YK, Unigene12216_YK, Unigene12219_YK, Unigene1223_YK, Unigene12733_YK, Unigene13180_YK, Unigene13237_YK, Unigene1356_YK, Unigene13670_YK, Unigene13877_YK, Unigene13937_YK, Unigene13945_YK, Unigene13960_YK, Unigene14094_YK, Unigene14243_YK, Unigene14922_YK, Unigene15057_YK, Unigene15348_YK, Unigene15587_YK, Unigene15877_YK, Unigene16000_YK, Unigene16029_YK, Unigene16112_YK, Unigene16638_YK, Unigene17790_YK, Unigene17811_YK, Unigene18574_YK, Unigene18819_YK, Unigene18931_YK, Unigene201_YK, Unigene2080_YK, Unigene2550_YK, Unigene258_YK, Unigene319_YK, Unigene3219_YK, Unigene331_YK, Unigene337_YK, Unigene3439_YK, Unigene3626_YK, Unigene4062_YK, Unigene4419_YK, Unigene4445_YK, Unigene4689_YK, Unigene4690_YK, Unigene4778_YK, Unigene5385_YK, Unigene5386_YK, Unigene5467_YK, Unigene5468_YK, Unigene6006_YK, Unigene6007_YK, Unigene6227_YK, Unigene6367_YK, Unigene6368_YK, Unigene6406_YK, Unigene6432_YK, Unigene6461_YK, Unigene6611_YK, Unigene6980_YK, Unigene6_YK, Unigene7307_YK, Unigene7308_YK, Unigene7313_YK, Unigene7314_YK, Unigene7382_YK, Unigene7390_YK, Unigene7391_YK, Unigene7525_YK, Unigene8101_YK, Unigene838_YK, Unigene8606_YK, Unigene8677_YK, Unigene8678_YK, Unigene8679_YK, Unigene8682_YK, Unigene8683_YK, Unigene904_YK, Unigene905_YK, Unigene9168_YK, Unigene9169_YK, Unigene9459_YK, Unigene9984_YK |
| 60 | [GABAergic synapse](../../../../D:%5C高通量测序结果%5CF14FTSSCKF1242_NEMpnqE%5Cannotation%5CKEGG%5CYK-Unigene.fa_map%5Cmap04727.html) | CL1088.Contig1_YK, CL1088.Contig2_YK, CL149.Contig1_YK, CL149.Contig2_YK, CL1689.Contig2_YK, CL1767.Contig2_YK, CL1838.Contig1_YK, CL1838.Contig2_YK, CL1896.Contig1_YK, CL1896.Contig2_YK, CL1896.Contig3_YK, CL1919.Contig1_YK, CL1919.Contig2_YK, CL2181.Contig1_YK, CL2181.Contig2_YK, CL2265.Contig1_YK, CL2265.Contig2_YK, CL2316.Contig1_YK, CL2316.Contig2_YK, CL2407.Contig1_YK, CL255.Contig1_YK, CL255.Contig2_YK, CL255.Contig3_YK, CL255.Contig4_YK, CL255.Contig5_YK, CL255.Contig6_YK, CL255.Contig7_YK, CL255.Contig8_YK, CL2622.Contig1_YK, CL2622.Contig2_YK, CL2733.Contig2_YK, CL2790.Contig1_YK, CL2790.Contig2_YK, CL2861.Contig1_YK, CL2861.Contig2_YK, CL397.Contig1_YK, CL397.Contig2_YK, CL506.Contig1_YK, CL506.Contig2_YK, CL506.Contig3_YK, CL591.Contig1_YK, CL591.Contig2_YK, CL591.Contig3_YK, CL591.Contig4_YK, CL591.Contig5_YK, CL591.Contig6_YK, CL591.Contig7_YK, CL591.Contig8_YK, CL663.Contig1_YK, CL766.Contig1_YK, CL766.Contig2_YK, CL766.Contig3_YK, CL79.Contig1_YK, CL79.Contig2_YK, CL79.Contig3_YK, CL79.Contig4_YK, CL79.Contig5_YK, CL79.Contig6_YK, CL79.Contig7_YK, CL79.Contig8_YK, CL838.Contig1_YK, CL838.Contig2_YK, CL838.Contig3_YK, CL838.Contig4_YK, CL838.Contig5_YK, CL838.Contig8_YK, CL883.Contig3_YK, CL883.Contig4_YK, CL884.Contig1_YK, CL884.Contig3_YK, CL924.Contig10_YK, CL924.Contig11_YK, CL924.Contig12_YK, CL924.Contig1_YK, CL924.Contig2_YK, CL924.Contig3_YK, CL924.Contig4_YK, CL924.Contig5_YK, CL924.Contig6_YK, CL924.Contig7_YK, CL924.Contig8_YK, CL924.Contig9_YK, CL972.Contig1_YK, CL972.Contig2_YK, CL972.Contig3_YK, CL972.Contig4_YK, CL998.Contig1_YK, CL998.Contig3_YK, Unigene10267_YK, Unigene10506_YK, Unigene10507_YK, Unigene10627_YK, Unigene10760_YK, Unigene10781_YK, Unigene10790_YK, Unigene10908_YK, Unigene11836_YK, Unigene11889_YK, Unigene11890_YK, Unigene12037_YK, Unigene12216_YK, Unigene12219_YK, Unigene12292_YK, Unigene12293_YK, Unigene12622_YK, Unigene13102_YK, Unigene1335_YK, Unigene13969_YK, Unigene14080_YK, Unigene14159_YK, Unigene14507_YK, Unigene1450_YK, Unigene15442_YK, Unigene15450_YK, Unigene15493_YK, Unigene15706_YK, Unigene15784_YK, Unigene157_YK, Unigene15939_YK, Unigene16000_YK, Unigene16094_YK, Unigene16329_YK, Unigene16406_YK, Unigene16715_YK, Unigene18164_YK, Unigene181_YK, Unigene1964_YK, Unigene2121_YK, Unigene2148_YK, Unigene2231_YK, Unigene2289_YK, Unigene2493_YK, Unigene2582_YK, Unigene2583_YK, Unigene258_YK, Unigene2630_YK, Unigene2685_YK, Unigene2860_YK, Unigene3200_YK, Unigene331_YK, Unigene3596_YK, Unigene3727_YK, Unigene3885_YK, Unigene3937_YK, Unigene4291_YK, Unigene4564_YK, Unigene4565_YK, Unigene4772_YK, Unigene5305_YK, Unigene5306_YK, Unigene5438_YK, Unigene5780_YK, Unigene5781_YK, Unigene6407_YK, Unigene6455_YK, Unigene6697_YK, Unigene6766_YK, Unigene6921_YK, Unigene7481_YK, Unigene7525_YK, Unigene7622_YK, Unigene765_YK, Unigene7851_YK, Unigene7853_YK, Unigene8437_YK, Unigene8938_YK, Unigene9702_YK, Unigene9710_YK |
| 61 | [Axon guidance](../../../../D:%5C高通量测序结果%5CF14FTSSCKF1242_NEMpnqE%5Cannotation%5CKEGG%5CYK-Unigene.fa_map%5Cmap04360.html) | CL1136.Contig1_YK, CL1136.Contig2_YK, CL1381.Contig1_YK, CL1381.Contig2_YK, CL1558.Contig1_YK, CL1558.Contig2_YK, CL1577.Contig1_YK, CL1577.Contig2_YK, CL1577.Contig3_YK, CL1577.Contig4_YK, CL172.Contig10_YK, CL172.Contig11_YK, CL172.Contig12_YK, CL172.Contig13_YK, CL172.Contig14_YK, CL172.Contig15_YK, CL172.Contig1_YK, CL172.Contig2_YK, CL172.Contig3_YK, CL172.Contig4_YK, CL172.Contig5_YK, CL172.Contig6_YK, CL172.Contig7_YK, CL172.Contig8_YK, CL172.Contig9_YK, CL1816.Contig1_YK, CL1816.Contig2_YK, CL2006.Contig1_YK, CL2006.Contig2_YK, CL2006.Contig3_YK, CL2047.Contig1_YK, CL2047.Contig2_YK, CL2047.Contig3_YK, CL2125.Contig1_YK, CL2135.Contig1_YK, CL2135.Contig2_YK, CL2140.Contig1_YK, CL2140.Contig2_YK, CL2449.Contig1_YK, CL2449.Contig2_YK, CL2635.Contig1_YK, CL2635.Contig2_YK, CL2668.Contig1_YK, CL2668.Contig2_YK, CL2856.Contig2_YK, CL2887.Contig2_YK, CL326.Contig1_YK, CL326.Contig2_YK, CL431.Contig1_YK, CL431.Contig2_YK, CL431.Contig3_YK, CL512.Contig3_YK, CL741.Contig2_YK, CL741.Contig3_YK, CL774.Contig1_YK, CL774.Contig2_YK, CL810.Contig1_YK, CL810.Contig2_YK, CL810.Contig3_YK, CL810.Contig4_YK, CL832.Contig1_YK, CL856.Contig2_YK, Unigene10037_YK, Unigene10096_YK, Unigene10132_YK, Unigene10165_YK, Unigene10439_YK, Unigene10440_YK, Unigene10441_YK, Unigene10546_YK, Unigene10547_YK, Unigene10645_YK, Unigene10646_YK, Unigene10647_YK, Unigene10659_YK, Unigene11093_YK, Unigene11368_YK, Unigene11369_YK, Unigene11370_YK, Unigene11371_YK, Unigene11647_YK, Unigene11931_YK, Unigene12090_YK, Unigene12974_YK, Unigene12975_YK, Unigene13086_YK, Unigene13203_YK, Unigene13296_YK, Unigene13468_YK, Unigene13560_YK, Unigene13589_YK, Unigene13605_YK, Unigene13674_YK, Unigene13723_YK, Unigene13877_YK, Unigene13969_YK, Unigene13985_YK, Unigene14254_YK, Unigene14735_YK, Unigene15373_YK, Unigene15561_YK, Unigene15910_YK, Unigene16250_YK, Unigene1689_YK, Unigene18164_YK, Unigene182_YK, Unigene18999_YK, Unigene213_YK, Unigene2270_YK, Unigene2320_YK, Unigene236_YK, Unigene2546_YK, Unigene257_YK, Unigene2652_YK, Unigene2697_YK, Unigene2899_YK, Unigene2918_YK, Unigene3012_YK, Unigene317_YK, Unigene3286_YK, Unigene3384_YK, Unigene3712_YK, Unigene3788_YK, Unigene3937_YK, Unigene3947_YK, Unigene4062_YK, Unigene4165_YK, Unigene4166_YK, Unigene4203_YK, Unigene4396_YK, Unigene4494_YK, Unigene4648_YK, Unigene4680_YK, Unigene4769_YK, Unigene4770_YK, Unigene4861_YK, Unigene4924_YK, Unigene4_YK, Unigene5101_YK, Unigene5125_YK, Unigene5191_YK, Unigene5410_YK, Unigene5438_YK, Unigene5524_YK, Unigene5587_YK, Unigene5708_YK, Unigene5819_YK, Unigene6561_YK, Unigene6664_YK, Unigene7098_YK, Unigene7382_YK, Unigene7448_YK, Unigene7458_YK, Unigene7590_YK, Unigene7620_YK, Unigene7675_YK, Unigene7816_YK, Unigene8552_YK, Unigene8719_YK, Unigene9163_YK, Unigene9186_YK, Unigene9323_YK, Unigene9388_YK, Unigene9968_YK, Unigene9969_YK |
| 62 | [Neurotrophin signaling pathway](../../../../D:%5C高通量测序结果%5CF14FTSSCKF1242_NEMpnqE%5Cannotation%5CKEGG%5CYK-Unigene.fa_map%5Cmap04722.html) | CL112.Contig1_YK, CL112.Contig2_YK, CL112.Contig3_YK, CL112.Contig4_YK, CL112.Contig5_YK, CL112.Contig6_YK, CL112.Contig7_YK, CL112.Contig8_YK, CL1308.Contig1_YK, CL1308.Contig2_YK, CL1381.Contig1_YK, CL1381.Contig2_YK, CL1602.Contig1_YK, CL1602.Contig2_YK, CL1653.Contig1_YK, CL1653.Contig2_YK, CL1714.Contig2_YK, CL1758.Contig1_YK, CL1758.Contig2_YK, CL1816.Contig1_YK, CL1816.Contig2_YK, CL1861.Contig1_YK, CL2006.Contig1_YK, CL2006.Contig2_YK, CL2006.Contig3_YK, CL2126.Contig1_YK, CL2126.Contig2_YK, CL2168.Contig1_YK, CL218.Contig1_YK, CL218.Contig2_YK, CL238.Contig1_YK, CL238.Contig2_YK, CL238.Contig3_YK, CL2428.Contig1_YK, CL2428.Contig2_YK, CL2447.Contig1_YK, CL2447.Contig2_YK, CL2593.Contig1_YK, CL2593.Contig2_YK, CL2593.Contig3_YK, CL2624.Contig1_YK, CL2624.Contig2_YK, CL2668.Contig1_YK, CL2668.Contig2_YK, CL2764.Contig1_YK, CL2764.Contig2_YK, CL2792.Contig1_YK, CL2793.Contig1_YK, CL2793.Contig2_YK, CL2800.Contig1_YK, CL2800.Contig2_YK, CL475.Contig1_YK, CL475.Contig2_YK, CL475.Contig3_YK, CL475.Contig4_YK, CL539.Contig1_YK, CL539.Contig2_YK, CL539.Contig3_YK, CL539.Contig4_YK, CL568.Contig1_YK, CL568.Contig2_YK, CL577.Contig1_YK, CL577.Contig2_YK, CL577.Contig5_YK, CL774.Contig1_YK, CL774.Contig2_YK, CL832.Contig1_YK, CL902.Contig1_YK, CL902.Contig2_YK, Unigene10145_YK, Unigene10197_YK, Unigene10198_YK, Unigene10577_YK, Unigene10751_YK, Unigene10752_YK, Unigene10753_YK, Unigene107_YK, Unigene10940_YK, Unigene10972_YK, Unigene10993_YK, Unigene11335_YK, Unigene11546_YK, Unigene11784_YK, Unigene11931_YK, Unigene12218_YK, Unigene12237_YK, Unigene12451_YK, Unigene13436_YK, Unigene13571_YK, Unigene13674_YK, Unigene13723_YK, Unigene13937_YK, Unigene13960_YK, Unigene13985_YK, Unigene14243_YK, Unigene14278_YK, Unigene14345_YK, Unigene14456_YK, Unigene14647_YK, Unigene14707_YK, Unigene14892_YK, Unigene15057_YK, Unigene15080_YK, Unigene15247_YK, Unigene15497_YK, Unigene15522_YK, Unigene15561_YK, Unigene15587_YK, Unigene15910_YK, Unigene16250_YK, Unigene16951_YK, Unigene18574_YK, Unigene18716_YK, Unigene18819_YK, Unigene18931_YK, Unigene19134_YK, Unigene2324_YK, Unigene2550_YK, Unigene2791_YK, Unigene3295_YK, Unigene3384_YK, Unigene3439_YK, Unigene3626_YK, Unigene372_YK, Unigene4003_YK, Unigene4203_YK, Unigene4494_YK, Unigene4680_YK, Unigene4_YK, Unigene5219_YK, Unigene5358_YK, Unigene5642_YK, Unigene6227_YK, Unigene6229_YK, Unigene6230_YK, Unigene6231_YK, Unigene6451_YK, Unigene6461_YK, Unigene6561_YK, Unigene6611_YK, Unigene6664_YK, Unigene6820_YK, Unigene6_YK, Unigene7307_YK, Unigene7308_YK, Unigene7448_YK, Unigene7755_YK, Unigene7816_YK, Unigene7920_YK, Unigene8323_YK, Unigene8324_YK, Unigene8325_YK, Unigene8378_YK, Unigene8892_YK, Unigene8893_YK, Unigene9036_YK, Unigene9071_YK, Unigene9323_YK, Unigene9388_YK, Unigene9459_YK, Unigene9678_YK, Unigene9968_YK, Unigene9969_YK |
| 63 | [Oxidative phosphorylation](../../../../D:%5C高通量测序结果%5CF14FTSSCKF1242_NEMpnqE%5Cannotation%5CKEGG%5CYK-Unigene.fa_map%5Cmap00190.html) | CL1002.Contig1_YK, CL1002.Contig2_YK, CL1103.Contig1_YK, CL1247.Contig1_YK, CL1247.Contig2_YK, CL1286.Contig1_YK, CL1286.Contig2_YK, CL1286.Contig3_YK, CL1286.Contig4_YK, CL1286.Contig5_YK, CL1286.Contig6_YK, CL1286.Contig7_YK, CL1286.Contig8_YK, CL1288.Contig1_YK, CL1611.Contig1_YK, CL1611.Contig2_YK, CL1682.Contig1_YK, CL1826.Contig1_YK, CL1826.Contig2_YK, CL2176.Contig1_YK, CL2176.Contig2_YK, CL2275.Contig1_YK, CL2275.Contig2_YK, CL2322.Contig1_YK, CL2322.Contig2_YK, CL2626.Contig1_YK, CL2626.Contig2_YK, CL564.Contig10_YK, CL564.Contig13_YK, CL564.Contig14_YK, CL564.Contig16_YK, CL564.Contig2_YK, CL564.Contig3_YK, CL564.Contig5_YK, CL564.Contig6_YK, CL564.Contig7_YK, CL564.Contig8_YK, CL564.Contig9_YK, CL707.Contig1_YK, CL707.Contig2_YK, CL801.Contig1_YK, CL801.Contig2_YK, CL801.Contig3_YK, CL801.Contig5_YK, CL809.Contig1_YK, CL809.Contig2_YK, Unigene10224_YK, Unigene10262_YK, Unigene10263_YK, Unigene10600_YK, Unigene10777_YK, Unigene10895_YK, Unigene11281_YK, Unigene11439_YK, Unigene11592_YK, Unigene11740_YK, Unigene12025_YK, Unigene12259_YK, Unigene12376_YK, Unigene12990_YK, Unigene13046_YK, Unigene13063_YK, Unigene13065_YK, Unigene13166_YK, Unigene13210_YK, Unigene13229_YK, Unigene13231_YK, Unigene13232_YK, Unigene13234_YK, Unigene13274_YK, Unigene13365_YK, Unigene13375_YK, Unigene13401_YK, Unigene13405_YK, Unigene13473_YK, Unigene13483_YK, Unigene13507_YK, Unigene13527_YK, Unigene13572_YK, Unigene13584_YK, Unigene13592_YK, Unigene13607_YK, Unigene13608_YK, Unigene13639_YK, Unigene13643_YK, Unigene13708_YK, Unigene13750_YK, Unigene13785_YK, Unigene13801_YK, Unigene13829_YK, Unigene13847_YK, Unigene13857_YK, Unigene13878_YK, Unigene13963_YK, Unigene14019_YK, Unigene14028_YK, Unigene14030_YK, Unigene14052_YK, Unigene14053_YK, Unigene14054_YK, Unigene14165_YK, Unigene14204_YK, Unigene14283_YK, Unigene14309_YK, Unigene14455_YK, Unigene14482_YK, Unigene14510_YK, Unigene14527_YK, Unigene14542_YK, Unigene14932_YK, Unigene14936_YK, Unigene14977_YK, Unigene15040_YK, Unigene15461_YK, Unigene15508_YK, Unigene15523_YK, Unigene15529_YK, Unigene15687_YK, Unigene15764_YK, Unigene15815_YK, Unigene1604_YK, Unigene16262_YK, Unigene16564_YK, Unigene16714_YK, Unigene17182_YK, Unigene1730_YK, Unigene18156_YK, Unigene19067_YK, Unigene19105_YK, Unigene19109_YK, Unigene2366_YK, Unigene2439_YK, Unigene2785_YK, Unigene2927_YK, Unigene3133_YK, Unigene3140_YK, Unigene332_YK, Unigene3381_YK, Unigene3397_YK, Unigene3859_YK, Unigene3868_YK, Unigene4033_YK, Unigene4454_YK, Unigene5018_YK, Unigene5019_YK, Unigene5321_YK, Unigene5322_YK, Unigene5412_YK, Unigene5832_YK, Unigene5975_YK, Unigene6166_YK, Unigene6209_YK, Unigene6789_YK, Unigene69_YK, Unigene7661_YK, Unigene8587_YK, Unigene8588_YK, Unigene9718_YK, Unigene9735_YK, Unigene9736_YK, Unigene9903_YK, Unigene9904_YK |
| 64 | [Pyrimidine metabolism](../../../../D:%5C高通量测序结果%5CF14FTSSCKF1242_NEMpnqE%5Cannotation%5CKEGG%5CYK-Unigene.fa_map%5Cmap00240.html) | CL1058.Contig4_YK, CL1177.Contig1_YK, CL1177.Contig2_YK, CL119.Contig1_YK, CL119.Contig2_YK, CL119.Contig3_YK, CL1560.Contig1_YK, CL1560.Contig2_YK, CL1560.Contig3_YK, CL1785.Contig1_YK, CL1785.Contig2_YK, CL1785.Contig3_YK, CL1786.Contig2_YK, CL1786.Contig3_YK, CL2042.Contig1_YK, CL2153.Contig1_YK, CL2153.Contig2_YK, CL2153.Contig3_YK, CL2153.Contig4_YK, CL2471.Contig1_YK, CL2471.Contig2_YK, CL2471.Contig3_YK, CL2471.Contig4_YK, CL2493.Contig1_YK, CL2594.Contig1_YK, CL2594.Contig2_YK, CL2594.Contig3_YK, CL2594.Contig4_YK, CL2637.Contig1_YK, CL2637.Contig2_YK, CL2815.Contig1_YK, CL2815.Contig2_YK, CL345.Contig1_YK, CL345.Contig2_YK, CL347.Contig1_YK, CL477.Contig3_YK, CL527.Contig10_YK, CL527.Contig1_YK, CL527.Contig4_YK, CL527.Contig5_YK, CL527.Contig6_YK, CL527.Contig7_YK, CL527.Contig8_YK, CL527.Contig9_YK, CL552.Contig1_YK, CL569.Contig1_YK, CL569.Contig2_YK, CL63.Contig1_YK, CL63.Contig2_YK, CL711.Contig1_YK, CL711.Contig2_YK, CL726.Contig1_YK, Unigene10178_YK, Unigene10410_YK, Unigene10665_YK, Unigene10926_YK, Unigene11120_YK, Unigene112_YK, Unigene11701_YK, Unigene11965_YK, Unigene12930_YK, Unigene12982_YK, Unigene13071_YK, Unigene13076_YK, Unigene13282_YK, Unigene13494_YK, Unigene13623_YK, Unigene13696_YK, Unigene13841_YK, Unigene13873_YK, Unigene13979_YK, Unigene14075_YK, Unigene14196_YK, Unigene14199_YK, Unigene14264_YK, Unigene14368_YK, Unigene14497_YK, Unigene14606_YK, Unigene14733_YK, Unigene14744_YK, Unigene14789_YK, Unigene14876_YK, Unigene14885_YK, Unigene14905_YK, Unigene14923_YK, Unigene14925_YK, Unigene14927_YK, Unigene14934_YK, Unigene14991_YK, Unigene15050_YK, Unigene15053_YK, Unigene15054_YK, Unigene15079_YK, Unigene15184_YK, Unigene15220_YK, Unigene15246_YK, Unigene15345_YK, Unigene15478_YK, Unigene15541_YK, Unigene15575_YK, Unigene15718_YK, Unigene15726_YK, Unigene15729_YK, Unigene15916_YK, Unigene15974_YK, Unigene15979_YK, Unigene16066_YK, Unigene16177_YK, Unigene16211_YK, Unigene16273_YK, Unigene16299_YK, Unigene179_YK, Unigene18791_YK, Unigene2189_YK, Unigene2211_YK, Unigene2474_YK, Unigene2628_YK, Unigene2629_YK, Unigene2772_YK, Unigene2794_YK, Unigene2897_YK, Unigene2926_YK, Unigene2931_YK, Unigene3031_YK, Unigene3049_YK, Unigene338_YK, Unigene3793_YK, Unigene3794_YK, Unigene3845_YK, Unigene3846_YK, Unigene3934_YK, Unigene3935_YK, Unigene3959_YK, Unigene4002_YK, Unigene4040_YK, Unigene4041_YK, Unigene4103_YK, Unigene456_YK, Unigene457_YK, Unigene4611_YK, Unigene4775_YK, Unigene5136_YK, Unigene5196_YK, Unigene5365_YK, Unigene548_YK, Unigene5794_YK, Unigene5817_YK, Unigene5818_YK, Unigene5935_YK, Unigene6013_YK, Unigene6143_YK, Unigene6508_YK, Unigene6527_YK, Unigene6592_YK, Unigene6895_YK, Unigene7669_YK, Unigene9411_YK, Unigene9412_YK, Unigene9517_YK, Unigene9985_YK |
| 65 | [Fc gamma R-mediated phagocytosis](../../../../D:%5C高通量测序结果%5CF14FTSSCKF1242_NEMpnqE%5Cannotation%5CKEGG%5CYK-Unigene.fa_map%5Cmap04666.html) | CL1143.Contig1_YK, CL1143.Contig2_YK, CL1179.Contig1_YK, CL1179.Contig2_YK, CL1179.Contig3_YK, CL1179.Contig4_YK, CL1381.Contig1_YK, CL1381.Contig2_YK, CL1456.Contig3_YK, CL1469.Contig1_YK, CL1654.Contig1_YK, CL1814.Contig1_YK, CL1814.Contig2_YK, CL1888.Contig1_YK, CL1969.Contig1_YK, CL1969.Contig2_YK, CL215.Contig1_YK, CL2180.Contig1_YK, CL2180.Contig2_YK, CL2360.Contig1_YK, CL2360.Contig2_YK, CL2428.Contig1_YK, CL2428.Contig2_YK, CL2449.Contig1_YK, CL2449.Contig2_YK, CL2717.Contig2_YK, CL2751.Contig1_YK, CL2751.Contig2_YK, CL2792.Contig1_YK, CL475.Contig1_YK, CL475.Contig2_YK, CL475.Contig3_YK, CL475.Contig4_YK, CL52.Contig1_YK, CL591.Contig1_YK, CL591.Contig2_YK, CL591.Contig3_YK, CL591.Contig4_YK, CL591.Contig5_YK, CL591.Contig6_YK, CL591.Contig7_YK, CL591.Contig8_YK, CL636.Contig1_YK, CL674.Contig10_YK, CL674.Contig3_YK, CL728.Contig1_YK, CL728.Contig2_YK, CL800.Contig1_YK, CL800.Contig2_YK, CL832.Contig1_YK, CL859.Contig10_YK, CL859.Contig1_YK, CL859.Contig2_YK, CL859.Contig3_YK, CL859.Contig4_YK, CL859.Contig5_YK, CL859.Contig6_YK, CL859.Contig7_YK, CL859.Contig8_YK, CL859.Contig9_YK, CL884.Contig1_YK, CL884.Contig3_YK, CL966.Contig1_YK, CL966.Contig2_YK, CL966.Contig3_YK, CL966.Contig4_YK, CL966.Contig5_YK, CL966.Contig6_YK, Unigene10568_YK, Unigene10804_YK, Unigene10972_YK, Unigene11167_YK, Unigene11335_YK, Unigene11435_YK, Unigene11605_YK, Unigene11606_YK, Unigene12131_YK, Unigene12218_YK, Unigene12274_YK, Unigene12784_YK, Unigene12897_YK, Unigene12898_YK, Unigene12899_YK, Unigene13203_YK, Unigene13589_YK, Unigene13605_YK, Unigene13674_YK, Unigene13723_YK, Unigene13896_YK, Unigene13977_YK, Unigene14035_YK, Unigene14345_YK, Unigene14487_YK, Unigene14600_YK, Unigene14682_YK, Unigene14713_YK, Unigene14747_YK, Unigene14856_YK, Unigene15247_YK, Unigene15497_YK, Unigene15795_YK, Unigene15899_YK, Unigene16142_YK, Unigene16164_YK, Unigene16250_YK, Unigene16465_YK, Unigene16580_YK, Unigene16898_YK, Unigene1689_YK, Unigene179_YK, Unigene18183_YK, Unigene18577_YK, Unigene18676_YK, Unigene233_YK, Unigene2612_YK, Unigene2791_YK, Unigene2899_YK, Unigene3056_YK, Unigene3286_YK, Unigene3293_YK, Unigene4190_YK, Unigene4196_YK, Unigene4203_YK, Unigene4494_YK, Unigene4505_YK, Unigene4583_YK, Unigene4761_YK, Unigene4861_YK, Unigene4912_YK, Unigene4939_YK, Unigene5219_YK, Unigene5924_YK, Unigene6066_YK, Unigene6229_YK, Unigene6230_YK, Unigene6231_YK, Unigene6309_YK, Unigene6310_YK, Unigene6553_YK, Unigene6598_YK, Unigene6670_YK, Unigene6920_YK, Unigene6980_YK, Unigene7511_YK, Unigene7525_YK, Unigene7590_YK, Unigene7610_YK, Unigene7611_YK, Unigene801_YK, Unigene8323_YK, Unigene8324_YK, Unigene8325_YK, Unigene84_YK, Unigene8892_YK, Unigene8893_YK, Unigene978_YK, Unigene979_YK |
| 66 | [Endocrine and other factor-regulated calcium reabsorption](../../../../D:%5C高通量测序结果%5CF14FTSSCKF1242_NEMpnqE%5Cannotation%5CKEGG%5CYK-Unigene.fa_map%5Cmap04961.html) | CL1058.Contig1_YK, CL1058.Contig2_YK, CL1058.Contig3_YK, CL1058.Contig4_YK, CL1088.Contig1_YK, CL1088.Contig2_YK, CL1143.Contig1_YK, CL1143.Contig2_YK, CL1260.Contig1_YK, CL1260.Contig2_YK, CL1260.Contig3_YK, CL15.Contig10_YK, CL15.Contig11_YK, CL15.Contig12_YK, CL15.Contig13_YK, CL15.Contig14_YK, CL15.Contig15_YK, CL15.Contig16_YK, CL15.Contig17_YK, CL15.Contig18_YK, CL15.Contig19_YK, CL15.Contig1_YK, CL15.Contig20_YK, CL15.Contig21_YK, CL15.Contig22_YK, CL15.Contig23_YK, CL15.Contig24_YK, CL15.Contig2_YK, CL15.Contig3_YK, CL15.Contig4_YK, CL15.Contig5_YK, CL15.Contig6_YK, CL15.Contig7_YK, CL15.Contig8_YK, CL15.Contig9_YK, CL1630.Contig2_YK, CL2283.Contig1_YK, CL2283.Contig2_YK, CL2283.Contig3_YK, CL2283.Contig4_YK, CL255.Contig1_YK, CL255.Contig2_YK, CL255.Contig3_YK, CL255.Contig4_YK, CL255.Contig5_YK, CL255.Contig6_YK, CL255.Contig7_YK, CL255.Contig8_YK, CL307.Contig10_YK, CL307.Contig11_YK, CL307.Contig12_YK, CL307.Contig1_YK, CL307.Contig2_YK, CL307.Contig3_YK, CL307.Contig4_YK, CL307.Contig5_YK, CL307.Contig6_YK, CL307.Contig7_YK, CL307.Contig8_YK, CL307.Contig9_YK, CL529.Contig1_YK, CL529.Contig2_YK, CL529.Contig3_YK, CL529.Contig4_YK, CL529.Contig5_YK, CL529.Contig6_YK, CL591.Contig1_YK, CL591.Contig2_YK, CL591.Contig3_YK, CL591.Contig4_YK, CL591.Contig5_YK, CL591.Contig6_YK, CL591.Contig7_YK, CL591.Contig8_YK, CL728.Contig1_YK, CL728.Contig2_YK, CL79.Contig1_YK, CL79.Contig2_YK, CL79.Contig3_YK, CL79.Contig4_YK, CL79.Contig5_YK, CL79.Contig6_YK, CL79.Contig7_YK, CL79.Contig8_YK, CL801.Contig1_YK, CL801.Contig2_YK, CL801.Contig3_YK, CL801.Contig4_YK, CL801.Contig5_YK, CL801.Contig6_YK, CL884.Contig1_YK, CL884.Contig3_YK, CL966.Contig1_YK, CL966.Contig2_YK, CL966.Contig3_YK, CL966.Contig4_YK, CL966.Contig5_YK, CL966.Contig6_YK, Unigene10001_YK, Unigene10568_YK, Unigene10887_YK, Unigene10888_YK, Unigene11435_YK, Unigene11605_YK, Unigene11606_YK, Unigene11937_YK, Unigene12216_YK, Unigene12219_YK, Unigene1223_YK, Unigene12292_YK, Unigene12293_YK, Unigene12478_YK, Unigene12636_YK, Unigene12638_YK, Unigene12639_YK, Unigene12768_YK, Unigene13055_YK, Unigene13192_YK, Unigene13237_YK, Unigene13454_YK, Unigene13750_YK, Unigene14589_YK, Unigene158_YK, Unigene16843_YK, Unigene1964_YK, Unigene2231_YK, Unigene2287_YK, Unigene2289_YK, Unigene258_YK, Unigene2770_YK, Unigene331_YK, Unigene3416_YK, Unigene4252_YK, Unigene4435_YK, Unigene5334_YK, Unigene5476_YK, Unigene5477_YK, Unigene5511_YK, Unigene5557_YK, Unigene5857_YK, Unigene6206_YK, Unigene6725_YK, Unigene6980_YK, Unigene7525_YK, Unigene8101_YK, Unigene8587_YK, Unigene8589_YK, Unigene8590_YK, Unigene8933_YK, Unigene8934_YK, Unigene904_YK, Unigene905_YK, Unigene9715_YK, Unigene9716_YK, Unigene9717_YK, Unigene9884_YK |
| 67 | [Peroxisome](../../../../D:%5C高通量测序结果%5CF14FTSSCKF1242_NEMpnqE%5Cannotation%5CKEGG%5CYK-Unigene.fa_map%5Cmap04146.html) | CL100.Contig1_YK, CL1261.Contig1_YK, CL1261.Contig2_YK, CL1569.Contig1_YK, CL1569.Contig2_YK, CL1763.Contig1_YK, CL1763.Contig2_YK, CL1837.Contig1_YK, CL1837.Contig2_YK, CL1880.Contig1_YK, CL1941.Contig1_YK, CL1943.Contig1_YK, CL1943.Contig2_YK, CL1974.Contig1_YK, CL1974.Contig2_YK, CL2024.Contig1_YK, CL2119.Contig1_YK, CL2119.Contig2_YK, CL2166.Contig1_YK, CL2166.Contig2_YK, CL2222.Contig1_YK, CL2222.Contig2_YK, CL2410.Contig1_YK, CL2410.Contig2_YK, CL2410.Contig3_YK, CL2531.Contig1_YK, CL2531.Contig2_YK, CL2717.Contig2_YK, CL401.Contig1_YK, CL401.Contig2_YK, CL413.Contig1_YK, CL413.Contig2_YK, CL560.Contig1_YK, CL560.Contig2_YK, CL560.Contig3_YK, CL751.Contig2_YK, CL751.Contig3_YK, CL751.Contig4_YK, CL757.Contig1_YK, CL773.Contig1_YK, CL773.Contig2_YK, CL819.Contig1_YK, CL819.Contig2_YK, CL919.Contig1_YK, CL919.Contig2_YK, Unigene10082_YK, Unigene10204_YK, Unigene10205_YK, Unigene10275_YK, Unigene10541_YK, Unigene10542_YK, Unigene10594_YK, Unigene10776_YK, Unigene10873_YK, Unigene10874_YK, Unigene11265_YK, Unigene11293_YK, Unigene13064_YK, Unigene13121_YK, Unigene13189_YK, Unigene13190_YK, Unigene13283_YK, Unigene13306_YK, Unigene13444_YK, Unigene13476_YK, Unigene13486_YK, Unigene13866_YK, Unigene13928_YK, Unigene14044_YK, Unigene14172_YK, Unigene14209_YK, Unigene14258_YK, Unigene14284_YK, Unigene14354_YK, Unigene14355_YK, Unigene14358_YK, Unigene14377_YK, Unigene14409_YK, Unigene14489_YK, Unigene14568_YK, Unigene14711_YK, Unigene14863_YK, Unigene15088_YK, Unigene151_YK, Unigene15237_YK, Unigene15238_YK, Unigene15433_YK, Unigene15629_YK, Unigene15646_YK, Unigene15736_YK, Unigene15813_YK, Unigene15963_YK, Unigene16183_YK, Unigene16339_YK, Unigene16790_YK, Unigene17398_YK, Unigene17421_YK, Unigene17549_YK, Unigene18192_YK, Unigene2083_YK, Unigene2102_YK, Unigene2274_YK, Unigene2360_YK, Unigene2363_YK, Unigene2876_YK, Unigene2914_YK, Unigene2932_YK, Unigene2965_YK, Unigene2993_YK, Unigene3243_YK, Unigene3244_YK, Unigene3305_YK, Unigene3361_YK, Unigene3362_YK, Unigene3363_YK, Unigene3528_YK, Unigene3612_YK, Unigene3679_YK, Unigene3682_YK, Unigene3787_YK, Unigene3869_YK, Unigene4004_YK, Unigene4282_YK, Unigene4513_YK, Unigene4568_YK, Unigene4912_YK, Unigene5135_YK, Unigene5222_YK, Unigene5238_YK, Unigene52_YK, Unigene5303_YK, Unigene5382_YK, Unigene6131_YK, Unigene6280_YK, Unigene641_YK, Unigene6605_YK, Unigene6606_YK, Unigene6806_YK, Unigene6878_YK, Unigene7111_YK, Unigene753_YK, Unigene7563_YK, Unigene7668_YK, Unigene7710_YK, Unigene7737_YK, Unigene8035_YK, Unigene8509_YK, Unigene8510_YK, Unigene8806_YK, Unigene8807_YK, Unigene9487_YK, Unigene9488_YK |
| 68 | [Leukocyte transendothelial migration](../../../../D:%5C高通量测序结果%5CF14FTSSCKF1242_NEMpnqE%5Cannotation%5CKEGG%5CYK-Unigene.fa_map%5Cmap04670.html) | CL1221.Contig1_YK, CL1221.Contig2_YK, CL1221.Contig3_YK, CL1223.Contig1_YK, CL1223.Contig3_YK, CL1250.Contig1_YK, CL1250.Contig2_YK, CL1250.Contig3_YK, CL1381.Contig1_YK, CL1381.Contig2_YK, CL1415.Contig1_YK, CL1415.Contig2_YK, CL1488.Contig1_YK, CL1488.Contig2_YK, CL1577.Contig1_YK, CL1577.Contig2_YK, CL1577.Contig3_YK, CL1577.Contig4_YK, CL1816.Contig1_YK, CL1816.Contig2_YK, CL1831.Contig2_YK, CL2002.Contig1_YK, CL2002.Contig2_YK, CL2002.Contig3_YK, CL2002.Contig4_YK, CL2002.Contig5_YK, CL2006.Contig1_YK, CL2006.Contig2_YK, CL2006.Contig3_YK, CL2126.Contig1_YK, CL2126.Contig2_YK, CL215.Contig1_YK, CL2168.Contig1_YK, CL2180.Contig1_YK, CL2180.Contig2_YK, CL2360.Contig1_YK, CL2360.Contig2_YK, CL2612.Contig1_YK, CL2612.Contig2_YK, CL2624.Contig1_YK, CL2624.Contig2_YK, CL2732.Contig1_YK, CL2732.Contig2_YK, CL2792.Contig1_YK, CL2816.Contig1_YK, CL2816.Contig2_YK, CL2887.Contig2_YK, CL2905.Contig1_YK, CL2905.Contig2_YK, CL300.Contig1_YK, CL300.Contig2_YK, CL349.Contig1_YK, CL349.Contig2_YK, CL349.Contig4_YK, CL415.Contig1_YK, CL415.Contig2_YK, CL415.Contig3_YK, CL415.Contig4_YK, CL475.Contig1_YK, CL475.Contig2_YK, CL475.Contig3_YK, CL475.Contig4_YK, CL591.Contig1_YK, CL591.Contig2_YK, CL591.Contig3_YK, CL591.Contig4_YK, CL591.Contig5_YK, CL591.Contig6_YK, CL591.Contig7_YK, CL591.Contig8_YK, CL627.Contig1_YK, CL627.Contig2_YK, CL832.Contig1_YK, CL84.Contig2_YK, CL84.Contig3_YK, CL884.Contig1_YK, CL884.Contig3_YK, Unigene10240_YK, Unigene10384_YK, Unigene10385_YK, Unigene10386_YK, Unigene10387_YK, Unigene10954_YK, Unigene10972_YK, Unigene11093_YK, Unigene12898_YK, Unigene12899_YK, Unigene13296_YK, Unigene13560_YK, Unigene13571_YK, Unigene13670_YK, Unigene13720_YK, Unigene13723_YK, Unigene13743_YK, Unigene13811_YK, Unigene13969_YK, Unigene13977_YK, Unigene13985_YK, Unigene14081_YK, Unigene14097_YK, Unigene14105_YK, Unigene14254_YK, Unigene14581_YK, Unigene14707_YK, Unigene14929_YK, Unigene15166_YK, Unigene15325_YK, Unigene15497_YK, Unigene15629_YK, Unigene15714_YK, Unigene15899_YK, Unigene16061_YK, Unigene16250_YK, Unigene16409_YK, Unigene17370_YK, Unigene18164_YK, Unigene18316_YK, Unigene18999_YK, Unigene19044_YK, Unigene256_YK, Unigene2791_YK, Unigene2849_YK, Unigene2899_YK, Unigene2949_YK, Unigene3067_YK, Unigene3293_YK, Unigene3318_YK, Unigene3349_YK, Unigene3937_YK, Unigene4203_YK, Unigene4396_YK, Unigene4494_YK, Unigene4761_YK, Unigene4924_YK, Unigene5191_YK, Unigene5438_YK, Unigene6066_YK, Unigene6182_YK, Unigene6304_YK, Unigene6514_YK, Unigene7458_YK, Unigene7525_YK, Unigene7590_YK, Unigene8323_YK, Unigene8324_YK, Unigene8325_YK, Unigene8500_YK, Unigene8501_YK, Unigene8569_YK, Unigene8570_YK, Unigene9323_YK, Unigene9388_YK |
| 69 | [Parkinson's disease](../../../../D:%5C高通量测序结果%5CF14FTSSCKF1242_NEMpnqE%5Cannotation%5CKEGG%5CYK-Unigene.fa_map%5Cmap05012.html) | CL1103.Contig1_YK, CL1228.Contig1_YK, CL1247.Contig1_YK, CL1247.Contig2_YK, CL1288.Contig1_YK, CL1416.Contig1_YK, CL1416.Contig2_YK, CL1514.Contig1_YK, CL1514.Contig2_YK, CL1611.Contig1_YK, CL1611.Contig2_YK, CL1682.Contig1_YK, CL1968.Contig1_YK, CL1968.Contig2_YK, CL2122.Contig1_YK, CL2122.Contig2_YK, CL2192.Contig1_YK, CL2192.Contig2_YK, CL2294.Contig1_YK, CL2294.Contig2_YK, CL2626.Contig1_YK, CL2626.Contig2_YK, CL35.Contig1_YK, CL35.Contig2_YK, CL481.Contig1_YK, CL481.Contig2_YK, CL564.Contig10_YK, CL564.Contig13_YK, CL564.Contig14_YK, CL564.Contig16_YK, CL564.Contig2_YK, CL564.Contig3_YK, CL564.Contig5_YK, CL564.Contig6_YK, CL564.Contig7_YK, CL564.Contig8_YK, CL564.Contig9_YK, Unigene10409_YK, Unigene10600_YK, Unigene10777_YK, Unigene10895_YK, Unigene11072_YK, Unigene11281_YK, Unigene11554_YK, Unigene11555_YK, Unigene11592_YK, Unigene11740_YK, Unigene12025_YK, Unigene12133_YK, Unigene12259_YK, Unigene12376_YK, Unigene12743_YK, Unigene12990_YK, Unigene13063_YK, Unigene13065_YK, Unigene13073_YK, Unigene13166_YK, Unigene13229_YK, Unigene13231_YK, Unigene13234_YK, Unigene13375_YK, Unigene13387_YK, Unigene13401_YK, Unigene13473_YK, Unigene13483_YK, Unigene13500_YK, Unigene13507_YK, Unigene13584_YK, Unigene13592_YK, Unigene13607_YK, Unigene13608_YK, Unigene13639_YK, Unigene13643_YK, Unigene13706_YK, Unigene13708_YK, Unigene13785_YK, Unigene13801_YK, Unigene13829_YK, Unigene13847_YK, Unigene13878_YK, Unigene14028_YK, Unigene14030_YK, Unigene14052_YK, Unigene14053_YK, Unigene14054_YK, Unigene14165_YK, Unigene14204_YK, Unigene14283_YK, Unigene14309_YK, Unigene14455_YK, Unigene14482_YK, Unigene14510_YK, Unigene14527_YK, Unigene14542_YK, Unigene14672_YK, Unigene14977_YK, Unigene15028_YK, Unigene15040_YK, Unigene15214_YK, Unigene15277_YK, Unigene15508_YK, Unigene15523_YK, Unigene15529_YK, Unigene15764_YK, Unigene15775_YK, Unigene15815_YK, Unigene1604_YK, Unigene16564_YK, Unigene16638_YK, Unigene16714_YK, Unigene17182_YK, Unigene1730_YK, Unigene18156_YK, Unigene18679_YK, Unigene19067_YK, Unigene19105_YK, Unigene19109_YK, Unigene2329_YK, Unigene2439_YK, Unigene2785_YK, Unigene3133_YK, Unigene3140_YK, Unigene3397_YK, Unigene3868_YK, Unigene4033_YK, Unigene4419_YK, Unigene4454_YK, Unigene4994_YK, Unigene5018_YK, Unigene5019_YK, Unigene5322_YK, Unigene5412_YK, Unigene5595_YK, Unigene5832_YK, Unigene5916_YK, Unigene5975_YK, Unigene5995_YK, Unigene6789_YK, Unigene7589_YK, Unigene7661_YK, Unigene8606_YK, Unigene8677_YK, Unigene8678_YK, Unigene8679_YK, Unigene8682_YK, Unigene8683_YK, Unigene9735_YK, Unigene9736_YK, Unigene9903_YK, Unigene9904_YK |
| 70 | [Retinol metabolism](../../../../D:%5C高通量测序结果%5CF14FTSSCKF1242_NEMpnqE%5Cannotation%5CKEGG%5CYK-Unigene.fa_map%5Cmap00830.html) | CL1059.Contig1_YK, CL1059.Contig2_YK, CL1149.Contig1_YK, CL1149.Contig2_YK, CL1175.Contig1_YK, CL1175.Contig2_YK, CL1263.Contig1_YK, CL1263.Contig3_YK, CL1353.Contig1_YK, CL1353.Contig2_YK, CL1401.Contig1_YK, CL1401.Contig2_YK, CL1652.Contig1_YK, CL1652.Contig2_YK, CL1681.Contig1_YK, CL1681.Contig2_YK, CL1681.Contig3_YK, CL1813.Contig1_YK, CL1813.Contig2_YK, CL1837.Contig1_YK, CL1837.Contig2_YK, CL1941.Contig1_YK, CL1961.Contig2_YK, CL1994.Contig1_YK, CL1994.Contig2_YK, CL2092.Contig1_YK, CL2092.Contig2_YK, CL2119.Contig1_YK, CL2119.Contig2_YK, CL2188.Contig1_YK, CL2188.Contig2_YK, CL231.Contig1_YK, CL2419.Contig1_YK, CL243.Contig1_YK, CL243.Contig2_YK, CL2455.Contig1_YK, CL2455.Contig2_YK, CL2459.Contig1_YK, CL2459.Contig2_YK, CL2878.Contig1_YK, CL417.Contig1_YK, CL417.Contig2_YK, CL417.Contig3_YK, CL417.Contig4_YK, CL461.Contig1_YK, CL461.Contig2_YK, CL757.Contig1_YK, CL765.Contig1_YK, CL765.Contig2_YK, CL769.Contig1_YK, CL769.Contig2_YK, CL769.Contig3_YK, CL773.Contig1_YK, CL773.Contig2_YK, CL803.Contig1_YK, CL803.Contig2_YK, CL871.Contig1_YK, CL919.Contig1_YK, CL919.Contig2_YK, Unigene10028_YK, Unigene10031_YK, Unigene10501_YK, Unigene10650_YK, Unigene10719_YK, Unigene10755_YK, Unigene10835_YK, Unigene11200_YK, Unigene11222_YK, Unigene11253_YK, Unigene11459_YK, Unigene11460_YK, Unigene11461_YK, Unigene11466_YK, Unigene11467_YK, Unigene12113_YK, Unigene12132_YK, Unigene12522_YK, Unigene12699_YK, Unigene12764_YK, Unigene12797_YK, Unigene13019_YK, Unigene13283_YK, Unigene133_YK, Unigene13928_YK, Unigene14049_YK, Unigene14219_YK, Unigene14355_YK, Unigene14736_YK, Unigene14860_YK, Unigene15177_YK, Unigene15299_YK, Unigene1560_YK, Unigene15735_YK, Unigene15785_YK, Unigene15813_YK, Unigene15830_YK, Unigene16095_YK, Unigene16183_YK, Unigene16317_YK, Unigene16366_YK, Unigene17726_YK, Unigene18192_YK, Unigene1997_YK, Unigene2065_YK, Unigene2120_YK, Unigene2268_YK, Unigene2274_YK, Unigene2932_YK, Unigene2993_YK, Unigene3003_YK, Unigene3059_YK, Unigene3305_YK, Unigene3475_YK, Unigene3685_YK, Unigene3823_YK, Unigene3857_YK, Unigene4852_YK, Unigene5036_YK, Unigene5069_YK, Unigene5135_YK, Unigene5140_YK, Unigene5184_YK, Unigene5222_YK, Unigene5259_YK, Unigene5281_YK, Unigene5282_YK, Unigene52_YK, Unigene5303_YK, Unigene5382_YK, Unigene5635_YK, Unigene5723_YK, Unigene6642_YK, Unigene6643_YK, Unigene6804_YK, Unigene7111_YK, Unigene753_YK, Unigene7558_YK, Unigene7942_YK, Unigene7943_YK, Unigene828_YK, Unigene8393_YK, Unigene8394_YK, Unigene9315_YK, Unigene9854_YK, Unigene9856_YK, Unigene9857_YK, Unigene9976_YK, Unigene9977_YK |
[truncated: 232,067 more chars]
